# Supplementary material for: Risk factors for overtaking, rear-end, and door crashes involving bicycles in the United Kingdom: Revisited and reanalysed
Source: PLoS One. 2025 Jan 3;20(1):e0315692. doi: 10.1371/journal.pone.0315692 (PMC11698528; doi:10.1371/journal.pone.0315692)
Supplement: Attachment — Submitted filename: PONE-D-24-17126_Reviewed.pdf [file pone.0315692.s001.pdf]

PLOS ONE

Risk Factors for Overtaking, Rear-End, and Door Crashes Involving Bicycles in the United Kingdom: Revisited and Reanalysed

--Manuscript Draft--

**Manuscript Number:**	PONE-D-24-17126
**Article Type:**	Research Article
**Full Title:**	Risk Factors for Overtaking, Rear-End, and Door Crashes Involving Bicycles in the United Kingdom: Revisited and Reanalysed
**Short Title:**	Risk Factors for Overtaking, Rear-End, and Door Crashes Involving Bicycles in the United Kingdom
**Corresponding Author:**	Chih-Wei Pai<br>Taipei Medical University<br>Taipei, TAIWAN
**Keywords:**	Bicycle crash; Road segment; Overtaking crash; Rear-end crash; Door crash
**Abstract:**	Objectives<br><br>Relevant research has provided valuable insights into risk factors for bicycle crashes at intersections. However, few studies have focused explicitly on three common types of bicycle crashes on road segments: overtaking, rear-end, and door crashes.<br><br>Material and methods<br><br>The present study investigated risk factors for these three crash types on road segments. We analysed British STATS19 accident records from 1991 to 2020. Using multivariate logistic regression models, we estimated adjusted odds ratios (AORs) with 95% confidence intervals (CIs) for multiple risk factors. The analysis included 127,637 bicycle crashes, categorised into 18,350 overtaking, 44,962 rear-end, 6,363 door, and 57,962 other crashes.<br><br>Results<br><br>Significant risk factors for overtaking crashes included speed limits of ≥40 miles per hour (mph) (AOR = 2.238, 95% CI = 2.159–2.320), heavy goods vehicles (HGVs) as crash partners (AOR = 2.867, 95% CI 2.473–3.323), and elderly crash partners (AOR = 2.013, 95% CI = 1.937–2.092). For rear-end crashes, noteworthy risk factors included unlit darkness (AOR = 1.486, 95% CI = 1.404–1.573) and midnight hours (AOR = 1.269, 95% CI = 1.190–1.354). Factors associated with door crashes included speed limits of 20–30 mph (AOR = 16.185, 95% CI = 13.514–19.382) and taxi and private hire cars (AOR = 2.695, 95% CI = 2.310–3.145). Our joint-effect analysis revealed additional interesting results; for example, there were elevated risks for overtaking crashes in rural areas with elderly drivers as crash partners (AOR = 2.93, 95% CI = 2.79–3.08) and with HGVs as crash partners (AOR = 2.62, 95% CI = 2.46–2.78).<br><br>Conclusions<br><br>The aforementioned risk factors remained largely unchanged since 2011, when we conducted our previous study. However, the present study concluded that the detrimental effects of certain variables became more pronounced in certain situations. For example, cyclists in rural settings exhibited an elevated risk of overtaking crashes involving HGVs as crash partners.
**Order of Authors:**	Chun-Chieh Chao
	Hon-Ping Ma
	Li Wei
	Yen-Nung Lin
	Chenyi Chen

Powered by Editorial Manager® and ProduXion Manager® from Aries Systems Corporation

	Wafaa Saleh
	Bayu Satria Wiratama
	Akhmad Fajri Widodo
	Shou-Chien Hsu
	Shih Yu Ko
	Hui-An Lin
	Cheng-Wei Chan
	Chih-Wei Pai
**Additional Information:**	
**Question**	**Response**
**Financial Disclosure**<br><br>Enter a financial disclosure statement that describes the sources of funding for the work included in this submission. Review the submission guidelines for detailed requirements. View published research articles from *PLOS ONE* for specific examples.<br><br>This statement is required for submission and **will appear in the published article** if the submission is accepted. Please make sure it is accurate.<br><br>**Funded studies**<br>Enter a statement with the following details:<br>• Initials of the authors who received each award<br>• Grant numbers awarded to each author<br>• The full name of each funder<br>• URL of each funder website<br>• Did the sponsors or funders play any role in the study design, data collection and analysis, decision to publish, or preparation of the manuscript?<br><br>Did you receive funding for this work?	Yes
Please add funding details.<br>as follow-up to "**Financial Disclosure**<br><br>Enter a financial disclosure statement that describes the sources of funding for the work included in this submission. Review the submission guidelines for detailed	This study received financial support from the Ministry of Science and Technology, Taiwan (MOST 110–2410-H-038-016-MY2 and MOST 109–2314-B-038-066-); New Taipei City Hospital (NTPC113–002); and the National Science and Technology Council, Taiwan (NSTC 112-2410-H-038-016-MY2). The funders played no role in the design of the study, data collection and analysis, interpretation of data, or preparation of the manuscript.

requirements. View published research articles from *PLOS ONE* for specific examples.<br><br>This statement is required for submission and **will appear in the published article** if the submission is accepted. Please make sure it is accurate.<br><br>**Funded studies**<br>Enter a statement with the following details:<br>• Initials of the authors who received each award<br>• Grant numbers awarded to each author<br>• The full name of each funder<br>• URL of each funder website<br>• Did the sponsors or funders play any role in the study design, data collection and analysis, decision to publish, or preparation of the manuscript?<br><br>Did you receive funding for this work?"	
Please select the country of your main research funder (please select carefully as in some cases this is used in fee calculation).<br>as follow-up to "**Financial Disclosure**<br><br>Enter a financial disclosure statement that describes the sources of funding for the work included in this submission. Review the submission guidelines for detailed requirements. View published research articles from *PLOS ONE* for specific examples.<br><br>This statement is required for submission and **will appear in the published article** if the submission is accepted. Please make sure it is accurate.	TAIWAN - TW

**Funded studies**<br>Enter a statement with the following details:<br>• Initials of the authors who received each award<br>• Grant numbers awarded to each author<br>• The full name of each funder<br>• URL of each funder website<br>• Did the sponsors or funders play any role in the study design, data collection and analysis, decision to publish, or preparation of the manuscript?<br><br>Did you receive funding for this work?"	
**Competing Interests**<br><br>Use the instructions below to enter a competing interest statement for this submission. On behalf of all authors, disclose any competing interests that could be perceived to bias this work—acknowledging all financial support and any other relevant financial or non-financial competing interests.<br><br>This statement is required for submission and **will appear in the published article** if the submission is accepted. Please make sure it is accurate and that any funding sources listed in your Funding Information later in the submission form are also declared in your Financial Disclosure statement.<br><br>View published research articles from *PLOS ONE* for specific examples.	The authors have declared that no competing interests exist.

Powered by Editorial Manager® and ProduXion Manager® from Aries Systems Corporation

**NO authors have competing interests**<br><br>Enter: *The authors have declared that no competing interests exist.*<br><br>**Authors with competing interests**<br><br>Enter competing interest details beginning with this statement:<br><br>*I have read the journal's policy and the authors of this manuscript have the following competing interests: [insert competing interests here]*<br><br>* typeset	
**Ethics Statement**<br><br>Enter an ethics statement for this submission. This statement is required if the study involved:<br><br>• Human participants<br>• Human specimens or tissue<br>• Vertebrate animals or cephalopods<br>• Vertebrate embryos or tissues<br>• Field research<br><br>Write "N/A" if the submission does not require an ethics statement.<br><br>General guidance is provided below. Consult the submission guidelines for detailed instructions. **Make sure that all information entered here is included in the Methods section of the manuscript.**	This study was approved by the Joint Institutional Review Board of Taipei Medical University (N202011030). The Joint Institutional Review Board of Taipei Medical University has waived the requirement of informed consent. All methods were performed in accordance with the relevant guidelines and regulations of the Declaration of Helsinki.

Powered by Editorial Manager® and ProduXion Manager® from Aries Systems Corporation

**Format for specific study types**<br><br>**Human Subject Research (involving human participants and/or tissue)**<br>• Give the name of the institutional review board or ethics committee that approved the study<br>• Include the approval number and/or a statement indicating approval of this research<br>• Indicate the form of consent obtained (written/oral) or the reason that consent was not obtained (e.g. the data were analyzed anonymously)<br><br>**Animal Research (involving vertebrate animals, embryos or tissues)**<br>• Provide the name of the Institutional Animal Care and Use Committee (IACUC) or other relevant ethics board that reviewed the study protocol, and indicate whether they approved this research or granted a formal waiver of ethical approval<br>• Include an approval number if one was obtained<br>• If the study involved *non-human primates*, add *additional details* about animal welfare and steps taken to ameliorate suffering<br>• If anesthesia, euthanasia, or any kind of animal sacrifice is part of the study, include briefly which substances and/or methods were applied<br><br>**Field Research**<br><br>Include the following details if this study involves the collection of plant, animal, or other materials from a natural setting:<br>• Field permit number<br>• Name of the institution or relevant body that granted permission	
**Data Availability**<br><br>Authors are required to make all data underlying the findings described fully available, without restriction, and from the time of publication. PLOS allows rare exceptions to address legal and ethical concerns. See the PLOS Data Policy and FAQ for detailed information.	Yes - all data are fully available without restriction

A Data Availability Statement describing where the data can be found is required at submission. Your answers to this question constitute the Data Availability Statement and **will be published in the article**, if accepted.<br><br>**Important:** Stating 'data available on request from the author' is not sufficient. If your data are only available upon request, select 'No' for the first question and explain your exceptional situation in the text box.<br><br>Do the authors confirm that all data underlying the findings described in their manuscript are fully available without restriction?	
**Describe where the data may be found in full sentences. If you are copying our sample text, replace any instances of XXX with the appropriate details.**<br><br>• If the data are **held or will be held in a public repository**, include URLs, accession numbers or DOIs. If this information will only be available after acceptance, indicate this by ticking the box below. For example: *All XXX files are available from the XXX database (accession number(s) XXX, XXX.*).<br>• If the data are all contained **within the manuscript and/or Supporting Information files**, enter the following: *All relevant data are within the manuscript and its Supporting Information files.*<br>• If neither of these applies but you are able to provide **details of access elsewhere**, with or without limitations, please do so. For example:<br><br>*Data cannot be shared publicly because of [XXX]. Data are available from the XXX Institutional Data Access / Ethics Committee (contact via XXX) for researchers who meet the criteria for access to confidential data.*<br><br>*The data underlying the results presented in the study are available from (include the name of the third party*	This study utilised the British STATS19 database, which contains data on all road traffic accidents in the United Kingdom. The data that support the findings of this study are openly available at https://www.data.gov.uk/dataset/cb7ae6f0-4be6-4935-9277-47e5ce24a11f/road-safety-data.

and contact information or URL).<br>• This text is appropriate if the data are owned by a third party and authors do not have permission to share the data.<br><br>* typeset	
Additional data availability information:	

Manuscript

Click here to access/download;Manuscript;4-28 Main Risk Factors for Overtaking.docx

Risk Factors for Overtaking, Rear-End, and Door Crashes Involving Bicycles in the United Kingdom: Revisited and Reanalysed

Chun-Chieh Chao[1,2,3†], Hon-Ping Ma[1,3,7], Li Wei[1,8,9], Yen-Nung Lin[1,10], Chenyi Chen[1], Wafaa Saleh[11], Bayu Satria Wiratama[12], Akhmad Fajri Widodo[1], Shou-Chien Hsu[6,13], Shih Yu Ko[7], Hui-An Lin[1,2,3†], Cheng-Wei Chan[1,4,5,6†], Chih-Wei Pai[1*]

[1] Graduate Institute of Injury Prevention and Control, College of Public Health, Taipei Medical University, Taipei City, Taiwan, [2] Department of Emergency Medicine, Taipei Medical University Hospital, Taipei City, Taiwan, [3] Department of Emergency Medicine, School of Medicine, College of Medicine, Taipei Medical University, Taipei, Taiwan, [4] Department of Emergency Medicine, New Taipei City Hospital, New Taipei City, Taiwan, [5] College of Medicine, Chang Gung University, Taoyuan City, Taiwan, [6] Department of Emergency Medicine, Chang Gung Memorial Hospital, Linkou branch, Taoyuan, Taiwan, [7] Department of Emergency Medicine, Taipei Medical University-Shuang Ho Hospital, New Taipei City, Taiwan, [8] Taipei Neuroscience Institute, Taipei Medical University, Taipei, Taiwan, [9] Division of Neurosurgery, Department of Surgery, Wan Fang Hospital, Taipei Medical University, Taipei, Taiwan, [10] Department of Physical Medicine and Rehabilitation, Wan Fang Hospital, Taipei Medical University, Taipei, Taiwan, [11] Transport Research Institute, Edinburgh Napier University, Edinburgh, Scotland, [12] Department of Epidemiology, Biostatistics, and Population Health, Faculty of Medicine, Public Health and Nursing, Universitas Gadjah Mada, Yogyakarta City, Indonesia, [13] Department of Occupational Medicine, Chang Gung Memorial Hospital, Linkou branch, Taoyuan, Taiwan.

* Correspondence: cpai@tmu.edu.tw

† Contributed equally to this work

Abstract

Objectives

Relevant research has provided valuable insights into risk factors for bicycle crashes at intersections. However, few studies have focused explicitly on three common types of bicycle crashes on road segments: overtaking, rear-end, and door crashes.

Material and methods

The present study investigated risk factors for these three crash types on road segments. We analysed British STATS19 accident records from 1991 to 2020. Using multivariate logistic regression models, we estimated adjusted odds ratios (AORs) with 95% confidence intervals (CIs) for multiple risk factors. The analysis included 127,637 bicycle crashes, categorised into 18,350 overtaking, 44,962 rear-end, 6,363 door, and 57,962 other crashes.

Results

Significant risk factors for overtaking crashes included speed limits of ≥40 miles per hour (mph) (AOR = 2.238, 95% CI = 2.159–2.320), heavy goods vehicles (HGVs) as crash partners (AOR = 2.867, 95% CI 2.473–3.323), and elderly crash partners (AOR = 2.013, 95% CI = 1.937–2.092). For rear-end crashes, noteworthy risk factors included unlit darkness (AOR = 1.486, 95% CI = 1.404–1.573) and midnight hours (AOR = 1.269, 95% CI = 1.190–1.354). Factors associated with door crashes included speed limits of 20–30 mph (AOR = 16.185, 95% CI = 13.514–19.382) and taxi and private hire cars (AOR = 2.695, 95% CI = 2.310–3.145). Our joint-effect analysis revealed additional interesting results; for example, there were elevated risks for overtaking crashes in rural areas with elderly drivers as crash partners (AOR = 2.93, 95% CI = 2.79–3.08) and with HGVs as crash partners (AOR = 2.62, 95% CI = 2.46–2.78).

Conclusions

The aforementioned risk factors remained largely unchanged since 2011, when we conducted our previous study. However, the present study concluded that the detrimental effects of certain variables became more pronounced in certain situations. For example, cyclists in rural settings exhibited an elevated risk of overtaking crashes involving HGVs as crash partners.

Keywords: Bicycle crash; Road segment; Overtaking crash; Rear-end crash; Door crash

Introduction

In recent years, urban bicycling has become increasingly popular in many countries, offering

benefits such as reduced traffic congestion, diminished parking pressure, and a reduction in greenhouse gas emissions [1, 2]. The World Health Organization has highlighted numerous health advantages of moderate-intensity physical activities such as bicycling, including improvements in life expectancy, quality of life, cognitive function, mental health, sleep quality, muscular and cardiorespiratory fitness, and bone and functional health [2].

However, despite such health benefits, the risk of injury remains a considerable safety concern for cyclists, who are regarded as vulnerable road users [2, 3]. Traffic crash data indicate that the risk of accidents for cyclists, measured per distance travelled, is approximately 20 times higher than that for vehicle drivers [2]. To address this problem, researchers in the United States developed a comprehensive bicycle route safety rating model with a focus on injury severity [4]. This model evaluates multiple operational and physical aspects such as traffic volume, population density, highway classification, lane width, and the presence of one-way streets. In addition, it is capable of predicting the severity of injuries due to motor vehicle–related crashes at specific locations [4]. Another finding was that a route is considered adequately safe if it includes geometric factors that enhance safety [4]. This model can aid urban planners and public officials in creating infrastructure such as bike lanes and implementing strict lane policies to improve cyclist safety [4]. Implementing bike lanes has been demonstrated to reduce crash rates by up to 40% among adult cyclists [5]. One study regarding roundabouts indicated that roundabouts with cycle tracks significantly reduced injury risk for cyclists compared with those lacking bicycle infrastructure [6]. Furthermore, adequate night-time lighting on rural roads has the potential to prevent over half of all cyclist injuries [7].

Although intersectional crashes are generally more frequent than nonintersectional ones, in

2020, 64% of fatal crashes involving cyclists occurred on road segments, defined as areas 20 m away from intersections, whereas only 26% of such fatalities occurred at intersections [8]. Bil et al. demonstrated that car drivers, when at fault for crashes, often cause more serious consequences for cyclists on straight road sections [9]. In crashes occurring on road segments, several factors contribute to high injury severity, including being in a rural region with an elevated speed limit, male gender, and cyclist age of >55 years [10]. Another identified risk factor is bicycling on roads against oncoming traffic [11].

Although relevant research has shed light on risk factors for bicycle crashes at intersections, few studies have explicitly investigated crashes on road segments. Studies that have examined bicycle crashes relatively broadly, without distinguishing crash types, have identified several key factors—including vehicle volume [13], traffic density [12], number of lanes [12], access points along road segments [13], shoulder and median widths [13], parking space availability [12, 13], length of continuous two-way left-turn lanes [13], and pavement type [14]—all of which contribute to crashes on road segments. Several studies have specifically explored overtaking, rear-end, and door crashes involving bicycles. A pioneering contribution in this area was made by Pai, who focused on these three types of crashes on road segments [15]. Specifically, Pai identified buses and coaches as common crash partners in overtaking crashes; poor visibility, traversing manoeuvres, and teenage cyclists as risk factors for rear-end crashes; and built-up areas as a risk factor for door crashes [15]. In addition, another study linked the speed of a passing vehicle to increased severity of cyclist injury in overtaking crashes [16].

The primary objective of the present study, an extension of our previous study [15], was to analyse police-reported crash data from additional years to determine whether the risk factors

for these three crash types remained unchanged. Furthermore, we aimed to untangle the joint associations of several factors—including light conditions, urban versus rural settings, vehicle types, and rider and driver characteristics—with these three crash types.

Material and Methods

Crash data source

The present investigation utilised data from 01/01/1991 to 31/12/2020, obtained from the United Kingdom's official road traffic casualty database, STATS19. Police record such data either at crash scenes or within 30 days of each crash. The UK's Department for Transport compiles the data, which the United Kingdom Data Archive then maintains and distributes. The dataset encompasses a variety of variables, including crash circumstances (e.g., time and date, weather conditions, road and light conditions, posted speed limit, road type), vehicle and driver characteristics, demographic details of the drivers, precrash manoeuvres of the vehicles, and the initial impact point of the vehicle. Additionally, the dataset contains demographic information and details regarding injury severity for each casualty. This study adhered to the STROBE (strengthening the reporting of observational studies in epidemiology) reporting guidelines.

Injury severity in the aforementioned dataset is divided into three categories, namely slight, serious, and fatal. Fatal injuries refer to those leading to death within 30 days of the accident. Serious injuries include conditions such as fractures, internal injuries, severe cuts and lacerations, concussions, and any injury requiring hospitalisation. Slight injuries include sprains, bruises, and minor cuts, as well as mild shock requiring roadside attention. The exclusive focus of this study was crashes leading to cyclist casualties.

As shown in Figure 1, this study analysed 1,366,196 crashes involving bicycles and other vehicles. Initially, 1,235,032 junction cases were excluded. From the remaining 131,164 bicycle segment crashes, 3,527 were further excluded because of incomplete demographic data for the cyclist and missing speed limit information, leaving a valid cohort of 127,637 bicycle segment crashes for analysis. Within this cohort, this study identified 18,350 overtaking crashes, 44,962 rear-end crashes, 6,363 door crashes, and 57,962 other types of crashes.

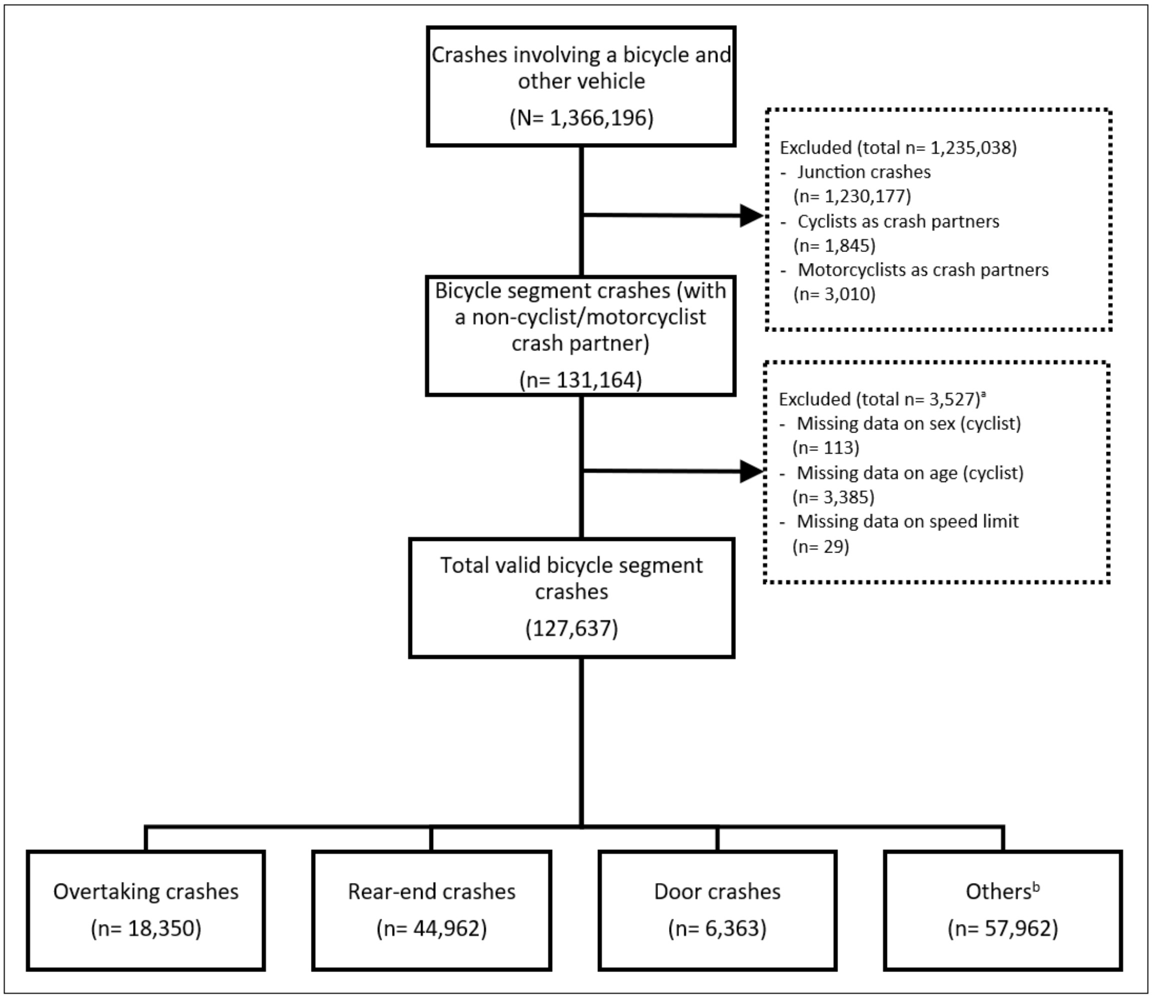

Fig. 1. Flowchart of the study sample selection process. [a]Listed excluded criteria are nonexclusive; thus, the sum of the total may exceed 3,527. [b]Other crashes include reversing crashes and head-on crashes.

Classification of crash types

An overtaking crash is defined as a crash where a motorised vehicle overtakes and collides with

a bicycle, which may be travelling straight, overtaking another vehicle, changing lanes, or turning. A rear-end crash occurs when a following vehicle collides with the rear of a bicycle. A door crash involves a bicycle either being struck by or striking the opening door of an automobile. These three crash types were described using schematics in our previous study [15].

Data collection

For the present study, the three crash types of focus (overtaking, rear-end, and door crashes) were the binary-dependent variables. The collected data encompassed the following factors: lighting conditions on the roadway at the time of the crash (daylight, darkness-lit, darkness-unlit), the speed limit at the crash scene (rural: ≥40 miles per hour [mph]; urban: 20–30 mph), the time of day categorised into four periods according to traffic volume (midnight: 00:00–06:00; rush hours: 07:00–08:00 and 17:00–18:00; nonrush hours: 09:00–16:00; and evening: 19:00–23:00), and the day of the week (weekday or weekend day). The demographic details of cyclist casualties encompassed age (≤18, 19–40, 41–64, or ≥65 years) and sex (male or female). Finally, the demographic details of the crash partner included the type of vehicle (identified as a taxi, private hire car, car, bus, or heavy goods vehicle [HGV]), age (≤18, 19–40, 41–64, or ≥65 years) and sex (male or female).

Statistical analysis

This study employed the chi-squared test to examine the associations between crash type and other factors, including cyclist or motorist characteristics, vehicle features, roadway conditions, and temporal variables. Variables with a p value lower than 0.2 in the univariate analysis were

subsequently incorporated into the multivariate logistic regression analysis [17]. All statistical analyses were conducted using SPSS Statistics version 25 for Windows (IBM Corp., Armonk, New York, USA). A p value lower than 0.05 in two-tailed tests was considered statistically significant.

Results

Population characteristics

Tables 1, 2, and 3 present the distributions of overtaking, rear-end, and door crashes, respectively, in relation to multiple independent variables. These data revealed that a significant proportion of bicycle crashes occurred in daylight (82.31%), occurred in urban settings (78.54%), occurred during nonrush hours (48.34%), occurred on weekdays (77.49%), involved cyclists aged under 18 years (40.11%), and involved male cyclists (81.30%). Additionally, most crashes involved cars as crash partners (83.57%), and crash partners were predominately aged 19–40 years (38.47%) and were male (76.35%). Table 1 highlights an overrepresentation in bicycle overtaking crashes for certain variables, namely unlit darkness (19.50%), rural areas (24.84%), midnight hours (17.71%), buses or HGVs as crash partners (24.72%), and elderly crash partners (21.47%) and male crash partners (15.99%). These results were revealed to be statistically significant by the chi-squared test ($p < 0.01$).

Table 1. Distribution of overtaking crashes according to a set of independent variables

Variable	Total (n=127,637)	Overtaking crashes (n=18,350)	Non-overtaking crashes (n=109,287)	*χ2 test p value*
**Light conditions**, n (%)				<0.001
Daylight	105,053 (82.31%)	15,283 (14.55%)	89,770 (85.45%)	
Darkness-lit	16,543 (12.96%)	1,889 (11,42%)	14,654 (88.58%)	
Darkness-unlit	6,041 (4.73%)	1,178 (19.50%)	4,863 (80.50%)	

Table 1. Distribution of overtaking crashes according to a set of independent variables *(continued)*

Variable	Total (n=127,637)	Overtaking crashes (n=18,350)	Non-overtaking crashes (n=109,287)	*χ2 test p value*
**Speed limit**, n (%)				<0.001
Rural (≥ 40 mph)	27,395 (21.46%)	6,805 (24.84%)	20,590 (75.61%)	
Urban (20–30 mph)	100,242 (78.54%)	11,545 (11.52%)	88,697 (88.48%)	
**Crash time (h)**, n (%)				<0.001
Midnight (00:00–06:00)	4,810 (3.77%)	852 (17.71%)	3,958 (82.29%)	
Rush hours (07:00–08:00/17:00–18:00)	41,619 (32.61%)	5,685 (13.66%)	35,934 (86.34%)	
Nonrush hours (09:00–16:00)	61,696 (48.34%)	9,386 (15.21%)	52,310 (84.79%)	
Evening (19:00–23:00)	19,512 (15.29%)	2,427 (12.44%)	17,085 (87.56%)	
**Crash day**, n (%)				0.094
Weekend	28,730 (22.51%)	4,218 (14.68%)	24,512 (85.21%)	
Weekday	98,907 (77.49%)	14,132 (14.29%)	84,775 (85.71%)	
**Cyclist's age (years)**, n (%)				<0.001
≤18	51,193 (40.11%)	5,220 (10.20%)	45,973 (89.80%)	
19–40	45,760 (35.85%)	7,108 (15.53%)	38,652 (84.47%)	
41–64	26,052 (20.41%)	5,012 (19.24%)	21,040 (80.76%)	
≥65	4,632 (3.63%)	1,010 (21.80%)	3,622 (78.20%)	
**Cyclist's sex**, n (%)				<0.001
Male	103,766 (81.30%)	14,746 (14.21%)	89,020 (85.79%)	
Female	23,871 (18.70%)	3,604 (15.10%)	20,267 (84.90%)	
**Crash partner**, n (%)				<0.001
Taxi/Private hire car	2,588 (2.03%)	208 (8.04%)	2,380 (91.96%)	
Car	106,668 (83.57%)	13,599 (12.75%)	93,069 (87.25%)	
Bus/Heavy goods vehicle	18,381 (14.40%)	4,543 (24.72%)	13,838 (75.28%)	
**Crash partner's age (years)**, n (%)				<0.001
≤18	2,415 (1.89%)	281 (11.64%)	2,134 (88.36%)	
19–40	49,103 (38.47%)	5,398 (10.99%)	43,705 (89.01%)	
41–64	35,598 (27.89%)	3,973 (11.16%)	31,625 (88.84%)	
≥65	40,521 (31.75%)	8,698 (21.47%)	31,823 (78.53%)	
**Crash partner's sex**, n (%)				<0.001
Male	97,447 (76.35)	15,584 (15.99%)	81,863 (84.01%)	
Female	30,190 (23.765%)	2,766 (9.16%)	27,424 (90.84%)	

As reported in Table 2, several variables, for instance, unlit darkness (50.19%), rural areas (43.03%), in midnight hours (47.59%), taxis as crash partners (42.35%), and elderly (39.67%) or male crash partners (36.77%) appeared to be disproportionately represented in bicycle rear-end crashes. These results were also revealed to be statistically significant by the chi-squared test ($p < 0.01$).

Table 2. Distribution of rear-end crashes according to a set of independent variables

Variable	Total (n=127,637)	Rear-end crashes (n=44,962)	Non-rear-end crashes (n=82,675)	*χ2 test p value*
**Light conditions**, n (%)				<0.001
Daylight	105,053 (82.31%)	35,726 (34.10%)	69,333 (66.00%)	
Darkness-lit	16,543 (12.96%)	6,204 (37.50%)	10,339 (63.50%)	
Darkness-unlit	6,041 (4.73%)	3,032 (50.19%)	3,003 (49.71%)	

Table 2. Distribution of rear-end crashes according to a set of independent variables *(continued)*

Variable	Total (n=127,637)	Rear-end crashes (n=44,962)	Non-rear-end crashes (n=82,675)	*χ2 test p value*
**Speed limit**, n (%)				<0.001
Rural (≥ 40 mph)	27,395 (21.46%)	11,788 (43.03%)	15,607 (56.97%)	
Urban (20–30 mph)	100,242 (78.54%)	33,174 (33.09%)	67,068 (66.91%)	
**Crash time (h)**, n (%)				<0.001
Midnight (00:00–06:00)	4,810 (3.77%)	2,289 (47.59%)	2,521 (52.41%)	
Rush hours (07:00–08:00/17:00–18:00)	41,619 (32.61%)	15,089 (36.26%)	26,530 (63.74%)	
Nonrush hours (09:00–16:00)	61,696 (48.34%)	20,723 (33.59%)	40,973 (66.41%)	
Evening (19:00–23:00)	19,512 (15.29%)	6,861 (36.16%)	12,651 (64.85%)	
**Crash day**, n (%)				<0.001
Weekend	28,730 (22.51%)	9,485 (33.01%)	19,245 (66.99%)	
Weekday	98,907 (77.49%)	35,477 (35.87%)	63,430 (64.13%)	
**Cyclist's age (years)**, n (%)				<0.001
≤18	51,193 (40.11%)	13,446 (26.27%)	37,747 (73.73%)	
19–40	45,760 (35.85%)	19,102 (41.74%)	26,658 (58.26%)	
41–64	26,052 (20.41%)	10,619 (40.76%)	15,433 (59.24%)	
≥65	4,632 (3.63%)	1,795 (38.75%)	2,837 (61.25%)	
**Cyclist's sex**, n (%)				<0.001
Male	103,766 (81.30%)	37,175 (35.83%)	66,591 (64.17%)	
Female	23,871 (18.70%)	7,787 (32.62%)	16,084 (67.38%)	
**Crash partner**, n (%)				<0.001
Taxi/Private hire car	2,588 (2.03%)	1,096 (42.35%)	1,492 (57.65%)	
Car	106,668 (83.57%)	37,202 (34.88%)	71,342 (66.88%)	
Bus/Heavy goods vehicle	18,381 (14.40%)	6,664 (36.25%)	9,841 (53.54%)	
**Crash partner's age (years)**, n (%)				<0.001
≤18	2,415 (1.89%)	870 (36.02%)	1,545 (63.98%)	
19–40	49,103 (38.47%)	16,282 (33.16%)	32,821 (66.84%)	
41–64	35,598 (27.89%)	11,736 (32.97%)	23,862 (67.03%)	
≥65	40,521 (31.75%)	16,074 (39.67%)	24,447 (60.33%)	
**Crash partner's sex**, n (%)				<0.001
Male	97,447 (76.35%)	35,828 (36.77%)	61,619 (63.23%)	
Female	30,190 (23.65%)	9,134 (30.26%)	21,056 (69.74%)	

Table 3 demonstrates that cyclists in several conditions, such as in unlit darkness (6.23%), in urban areas (6.22%), when they were female (8.21%), when taxi/private hire car were crash partners (10.55%), and when crash partners were female (7.42%), exhibited a higher risk of door crashes. These results were revealed to be statistically significant by the chi-squared test ($p < 0.01$).

Table 3. Distribution of door crashes according to a set of independent variables

Variable	Total (n=127,637)	Door crashes (n=6,363)	Non-door crashes (n=121,274)	*χ2 test p value*
**Light conditions**, n (%)				<0.001
Daylight	105,053 (82.31%)	5,192 (4.94%)	99,861 (95.06%)	
Darkness-lit	16,543 (12.96%)	1,031 (6.23%)	15,512 (93.77%)	
Darkness-unlit	6,041 (4.73%)	140 (2.32%)	5,901 (97.68%)	
**Speed limit**, n (%)				<0.001
Rural (≥ 40 mph)	27,395 (21.46%)	123 (0.45%)	27,272 (99.55%)	
Urban (20–30 mph)	100,242 (78.54%)	6,240 (6.22%)	94,002 (93.78%)	
**Crash time (h)**, n (%)				<0.001
Midnight (00:00–06:00)	4,810 (3.77%)	113 (2.35%)	4,697 (97.65%)	
Rush hours (07:00–08:00/17:00–18:00)	41,619 (32.61%)	2,056 (4.94%)	39,563 (95.06%)	
Nonrush hours (09:00–16:00)	61,696 (48.34%)	3,363 (5.54%)	58,333 (94.55%)	
Evening (19:00–23:00)	19,512 (15.29%)	831 (4.26%)	18,681 (95.74%)	
**Crash day**, n (%)				<0.001
Weekend	28,730 (22.51%)	1,072 (3.73%)	27,658 (96.27%)	
Weekday	98,907 (77.49%)	5,291 (5.35%)	93,616 (94.65%)	
**Cyclist's age (years)**, n (%)				<0.001
≤18	51,193 (40.11%)	802 (1.57%)	50,391 (98.43%)	
19–40	45,760 (35.85%)	3,474 (7.59%)	42,286 (93.41%)	
41–64	26,052 (20.41%)	1,773 (6.81%)	24,279 (93.19%)	
≥65	4,632 (3.63%)	314 (6.78%)	4,318 (93.22%)	
**Cyclist's sex**, n (%)				<0.001
Male	103,766 (81.30%)	4,404 (4.24%)	99,362 (95.76%)	
Female	23,871 (18.70%)	1,959 (8.21%)	21,912 (91.79%)	
**Crash partner**, n (%)				<0.001
Taxi/Private hire car	2,588 (2.03%)	273 (10.55%)	2,315 (89.45%)	
Car	106,668 (83.57%)	5,514 (5.17%)	101,154 (94.83%)	
Bus/Heavy goods vehicle	18,381 (14.40%)	576 (3.13%)	17,805 (96.87%)	
**Crash partner's age (years)**, n (%)				<0.001
≤18	2,415 (1.89%)	1,62 (5.22%)	2,253 (93.29%)	
19–40	49,103 (38.47%)	2,585 (5.26%)	46,518 (94.74%)	
41–64	35,598 (27.89%)	1,887 (5.30%)	33,711 (94.70%)	
≥65	40,521 (31.75%)	1,729 (4.27%)	38,792 (95.73%)	
**Crash partner's sex**, n (%)				<0.001
Male	97,447 (76.35%)	4,123 (4.23%)	93,324 (95.77%)	
Female	30,190 (23.65%)	2,240 (7.42%)	27,950 (92.58%)	

Risk factors for the three crash types

Table 4 presents the logistic regression model results. Regarding overtaking crashes, the identified risk factors included daylight conditions (adjusted odds ratio [AOR] = 1.233, 95% confidence interval [CI] = 1.162–1.309), speed limits of ≥40 mph (AOR = 2.238, 95% CI = 2.159–2.320), nonrush hours (AOR = 1.091, 95% CI 1.031–1.154), cyclists aged ≥65 years (AOR = 1.785,

95% CI = 1.649–1.931), female cyclists (AOR = 1.106, 95% CI = 1.062–1.153), HGVs as crash partners (AOR = 2.867, 95% CI = 2.473–3.323), elderly crash partners (AOR = 2.013, 95% CI = 1.937–2.092), and male crash partners (AOR = 1.353, 95% CI = 1.292–1.416).

For rear-end crashes, noteworthy risk factors included unlit darkness (AOR = 1.486, 95% CI = 1.404–1.573), speed limits of ≥40 mph (AOR = 1.315, 95% CI = 1.277–1.354), weekdays (AOR = 1.090, 95% CI = 1.059–1.122), midnight hours (AOR = 1.269, 95% CI = 1.190–1.354), and taxis as crash partners (AOR = 1.286, 95% CI = 1.186–1.394).

Regarding door crashes, significant risk factors included lit darkness (AOR = 1.373, 95% CI = 1.141–1.651), speed limits of 20–30 mph (AOR = 16.185, 95% CI = 13.514–19.382), weekdays (AOR = 1.246, 95% CI = 1.162–1.336), and nonrush hours (AOR = 2.912, 95% CI = 2.384–3.556). Additionally, female cyclists (AOR = 1.675, 95% CI = 1.582–1.774), taxis or private hire cars as crash partners (AOR = 2.695, 95% CI = 2.310–3.145), male crash partners (AOR = 1.373, 95% CI = 1.296–1.455), and crash partners aged 41–64 years (AOR = 1.855, 95% CI = 1.625–2.117) were associated with door crashes.

Table 4. Multivariate logistic regression results

Variable	Overtaking crashes		Rear-end crashes		Door crashes	
	AOR (95% CI)	*p* value	AOR (95% CI)	*p* value	AOR (95% CI)	*p* value
**Light condition**						
Daylight	1.233 (1.162, 1.309)	<0.001	Ref		1.146 (0.958, 1.370)	0.137
Darkness-lit	Ref		1.042 (1.002, 1.085)	0.041	1.373 (1.141, 1.651)	0.001
Darkness-unlit	1.152 (1.059, 1.253)	0.001	1.486 (1.404, 1.573)	<0.001	Ref	
**Speed limit**						
Rural (≥40 mph)	2.238 (2.159, 2.320)	<0.001	1.315 (1.277, 1.354)	<0.001	Ref	
Urban (20–30 mph)	Ref		Ref		16.185 (13.514, 19.382)	<0.001
**Crash time**						
Midnight	1.073 (0.982, 1.173)	0.119	1.269 (1.190, 1.354)	<0.001	Ref	
Rush hours	1.059 (1.002, 1.120)	0.043	1.108 (1.078, 1.139)	<0.001	2.502 (2.051, 3.052)	<0.001
Nonrush hours	1.091 (1.031, 1.154)	0.003	Ref		2.912 (2.384, 3.556)	<0.001
Evening	Ref		0.992 (0.953, 1.032)	0.686	2.014 (1.646, 2.465)	<0.001

Table 4. Multivariate logistic regression results *(continued)*

Variable	Overtaking crashes		Rear-end crashes		Door crashes	
	AOR (95% CI)	*p* value	AOR (95% CI)	*p* value	AOR (95% CI)	*p* value
**Crash day**						
Weekend	1.031 (0.991, 1.072)	0.132	Ref		Ref	
Weekday	Ref		1.090 (1.059, 1.122)	<0.001	1.246 (1.162, 1.336)	<0.001
**Cyclist's age (years)**						
≤18	Ref		Ref		Ref	
19–40	1.292 (1.242, 1.345)	<0.001	1.839 (1.788, 1.891)	<0.001	5.943 (5.489, 6.435)	<0.001
41–64	1.509 (1.444, 1.578)	<0.001	1.731 (1.676, 1.789)	<0.001	6.129 (5.621, 6.684)	<0.001
≥65	1.785 (1.649, 1.931)	<0.001	1.671 (1.568, 1.780)	<0.001	5.988 (5.217, 6.874)	<0.001
**Cyclist's sex**						
Male	Ref		1.172 (1.137, 1.208)	<0.001	Ref	
Female	1.106 (1.062, 1.153)	<0.001	Ref		1.675 (1.582, 1.774)	<0.001
**Crash partner**						
Taxi/Private hire car	Ref		1.286 (1.186, 1.394)	<0.001	2.695 (2.310, 3.145)	<0.001
Car	1.571 (1.359, 1.816)	<0.001	Ref		2.089 (1.908, 2.286)	<0.001
Bus/Heavy goods vehicle	2.867 (2.473, 3.323)	<0.001	1.099 (1.061, 1.139)	<0.001	Ref	
**Crash partner's age (years)**						
≤18	1.097 (0.963, 1.249)	0.162	1.225 (1.188, 1.263)	<0.001	1.507 (1.313, 1.731)	<0.001
19–40	Ref		1.038 (1.008, 1.069)	0.013	1.855 (1.625, 2.117)	<0.001
41–64	0.950 (0.909, 0.994)	0.025	Ref		1.801 (1.574, 2.060)	<0.001
≥65	2.013 (1.937, 2.092)	<0.001	1.241 (1.137, 1.355)	<0.001	Ref	
**Crash partner's sex**						
Male	1.353 (1.292, 1.416)	<0.001	1.150 (1.117, 1.185)	<0.001	1.373 (1.296, 1.455)	<0.001
Female	Ref		Ref		Ref	

Figure 2 presents a forest plot demonstrating the joint effects of several variables on the three crash types when other variables were controlled for. An elevated risk of overtaking crashes was evident in rural areas with elderly drivers as crash partners (AOR = 2.93, 95% CI = 2.79–3.08), HGVs as crash partners (AOR = 2.62, 95% CI = 2.46–2.78), and elderly cyclists involved in accidents during weekends (AOR = 1.56, 95% CI = 1.34-1.81). The risk of rear-end crashes was increased by the synergistic interaction of unlit darkness with midnight (AOR = 1.68, 95% CI = 1.48–1.90) and by rural areas (AOR = 2.15, 95% CI = 2.01–2.31). Furthermore, bicycling at midnight in rural areas was associated with an increased risk of rear-end crashes (AOR = 1.68; 95% CI = 1.51–1.86). In urban settings, the risk of door crashes was higher for female cyclists (AOR = 2.29; 95% CI = 2.17–

2.43) and for elderly cyclists (AOR = 2.06; 95% CI = 1.82–2.34). Finally, female cyclists exhibited a 112% higher likelihood of door crashes when the crash partner was a taxi (AOR = 2.12; 95% CI = 1.68–2.69).

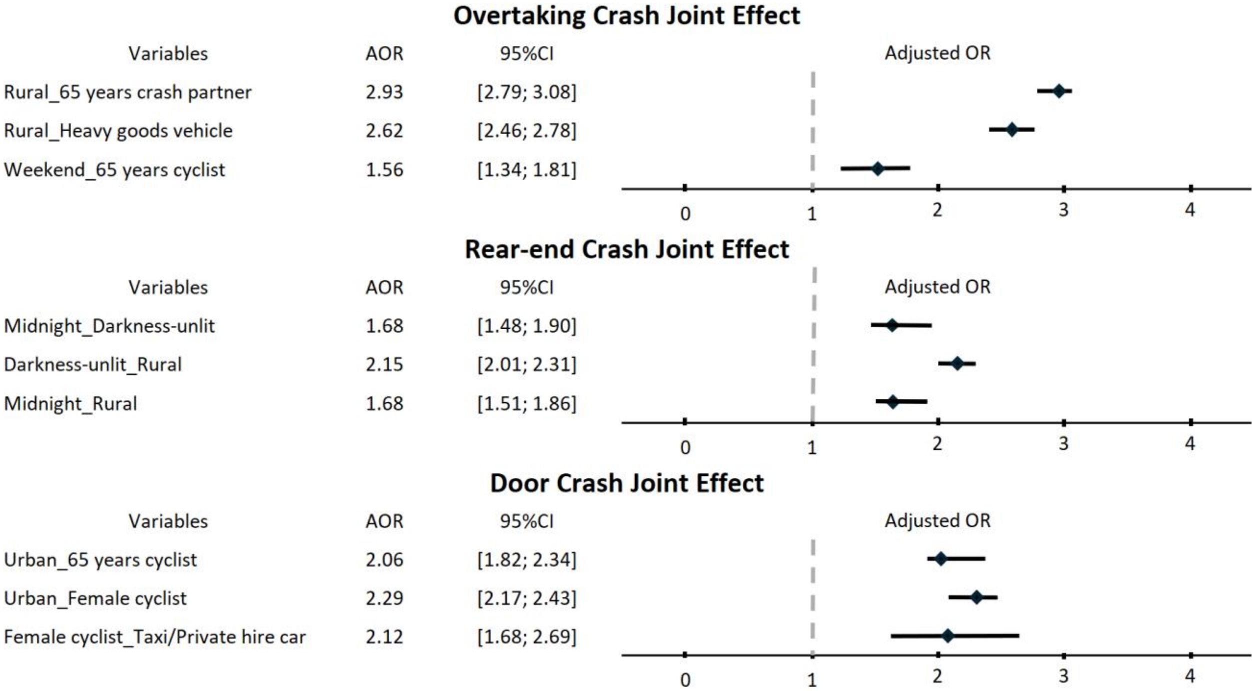

Fig. 2. Joint effects of several variables on the three crash types.

Discussion

This study explored the relationships among individual and environmental factors in relation to three common bicycle crash types (overtaking, rear-end, and door crashes) on roads in the United Kingdom from 1991 to 2020. The findings revealed several significant factors. First, for overtaking crashes, HGVs as crash partners, rural areas, and the involvement of elderly crash partners emerged as key contributing factors. Second, unlit darkness, midnight hours, and rural areas were the factors most closely associated with rear-end crashes. Third, urban areas and taxis as crash partners significantly increased the likelihood of door crashes. Moreover, male crash partners were found to be a consistent risk factor across all three crash types. These findings

warrant further discussion and thus are elaborated on in this section of this paper.

Our research findings identified specific risk factors for overtaking crashes, namely rural areas, HGVs as crash partners, and elderly crash partners. These findings align with those of a previous study [18], which similarly observed that elderly drivers, driving speeds surpassing 10 mph, and the presence of pick-up trucks increased the overtaking crash risk. We further found that the detrimental effect of HGVs on overtaking crashes was more pronounced in rural areas and when the crash partner was elderly. A behavioural study suggested that compared with cars, HGVs tended to maintain a narrower clearance zone when overtaking bicycles [19]. Pai et al. [15] speculated that the time pressures on HGV drivers for timely loading and unloading might lead to reckless driving. Our findings underscore the necessity of implementing measures such as 'Share the Road' warning signs [20], particularly in rural settings, where HGVs are likely to execute overtaking manoeuvres at high speed. Such measures could prompt motor vehicles to maintain safer distances from the edges of travel lanes, especially in areas with a notable presence of both HGVs and bicycles.

We also identified elderly drivers as a factor contributing to overtaking crashes—a finding consistent with relevant research [18]. As individuals age, their risk of being involved in road accidents is influenced by declines in their cognitive capabilities [21], their health [22], and their driving performance [23]. Notably, crashes involving elderly individuals often occur in scenarios with challenging conditions, including at intersections without traffic control measures, on high-speed roads, during adverse weather conditions, in poorly lit areas, and in head-on accidents [24–26]. The heightened level of risk under such conditions may be attributed to cognitive and perceptual decline in older drivers, which could affect their capacity to execute actions such as

overtaking manoeuvres safely. Accordingly, developing specialised cognitive training programmes as interventions to enhance road safety for elderly drivers is evidently necessary [27].

In the present study, several factors were found to increase the risk of rear-end crashes on road segments, including darkness with unlit surroundings, midnight hours, and rural settings (speed limit > 40 mph). Although few studies have specifically addressed rear-end crashes involving bicycles on road segments, available data suggest that the low conspicuity of bicycles, especially at night, is a recurrent factor in rear-end crashes [15, 28]. Moreover, a lack of adequate street lighting, which is common in rural settings, predisposes cyclists to rear-end crashes [15]. Our joint-effects analysis further indicated that the detrimental effect of unlit darkness is more pronounced in rural areas and during midnight hours. Potential intervention strategies to mitigate rear-end crashes include enhancing illumination and executing speed control management on rural road segments with heavy bicycle traffic.

Next, our analysis successfully identified associations of urban areas and taxis and private hire cars as crash partners with door crashes on road segments. Although research specifically focusing on door crashes on road segments is limited, similar findings were documented by Pai, indicating that urban roadways and taxis contributed to door crashes [15]. However, determining the factors influencing this trend poses a challenge. One possible explanation could be the increased presence of taxis or private hire cars in such areas, where passengers often disembark. Additionally, our analysis further revealed an elevated risk of door crashes involving crashes with taxis in urban areas. To reduce door crashes on road segments, educating taxi drivers, as well as passengers, about the importance of vigilance when opening doors near traffic is essential [15].

In addition, cyclists should be advised to maintain at least a door's width distance from all parked cars to improve the sight triangles of drivers and increase the visibility of cyclists [29]. Implementing a two-stage door opening mechanism for vehicles, which would enable drivers to verify the presence of bicycles to the rear, could also be beneficial [30].

This study had several limitations that warrant acknowledgement. First, the substantial underreporting of nonfatal casualties to the police, particularly casualties involving cyclists not obligated to report accidents, is a critical factor to consider. Such underreporting, as highlighted by the U.K. Government's Department for Transport [31], likely results in the incomplete representation of nonfatal and 'slight' casualties in road casualty data. Second, the STATS19 data utilised in this study lack critical variables, including precrash speeds, specific geometric characteristics of roadways, data regarding alcohol and illicit substance use, and cyclist speed at the time of an accident. Moreover, critical exposure data—such as those related to traffic flow, rider or driver experience, and other elements of risk exposure—are absent, and the absence of such details limits our ability to fully account for potential variations resulting from unobserved factors in the analyses. Finally, this study did not explore annual trends in each type of bicycle crash over the 30-year study period; investigating such trends could provide insights regarding changing behaviours among cyclists and motor vehicle drivers as well as the effects of legislative changes for road speed limits.

Conclusions

This study identified several significant risk factors for the three predominate types of crashes involving cyclists on road segments: HGVs as crash partners, elderly crash partners, and rural

areas for overtaking crashes; unlit darkness, midnight hours, and rural areas for rear-end crashes; and urban areas and taxis as crash partners for door crashes. These risk factors remained unchanged since our previous study conducted in 2011 [15]. The present research enhances the field of bicycle safety research by concluding that the detrimental effects of certain variables become more pronounced under certain conditions. For example, first, cyclists in rural settings exhibited an elevated risk of overtaking crashes involving HGVs. Second, the rear-end crash risk increases in the combined presence of unlit darkness, midnight hours, and rural areas. Finally, in urban settings, the likelihood of door crashes increases when a taxi is the crash partner.

Abbreviations

WHO: World Health Organization; HGVs: heavy goods vehicles; AOR: adjusted odds ratio; CI: confidence interval.

Acknowledgments

This manuscript was edited by Wallace Academic Editing.

Author contributions

Literature review: Chun-Chieh Chao.

Methodology: Chun-Chieh Chao, Chih-Wei Pai.

Data merging and analysis: Akhmad Fajri Widodo , Wafaa Saleh, Bayu Satria Wiratama.

Writing - original draft: Chun-Chieh Chao.

Writing – review and editing: Hui-An Lin, Chenyi Chen, Hon-Ping Ma, Akhmad Fajri Widodo.

Validation: Chun-Chieh Chao, Hui-An Lin, Chenyi Chen, Hon-Ping Ma, Shih Yu Ko.

Supervision: Li Wei, Yen-Nung Lin, Shou-Chien Hsu, Chih-Wei Pai.

Funding: Cheng-Wei Chan, Chih-Wei Pai.

Funding

This study received financial support from the Ministry of Science and Technology, Taiwan (MOST 110–2410-H-038-016-MY2 and MOST 109–2314-B-038-066-); New Taipei City Hospital (NTPC113–002); and the National Science and Technology Council, Taiwan (NSTC 112-2410-H-038-016-MY2). The funders played no role in the design of the study, data collection and analysis, interpretation of data, or preparation of the manuscript.

Availability of data and materials

This study utilised the British STATS19 database, which contains data on all road traffic accidents in the United Kingdom. The data that support the findings of this study are openly available at https://www.data.gov.uk/dataset/cb7ae6f0-4be6-4935-9277-47e5ce24a11f/road-safety-data.

Declarations

Ethical approval and consent to participate

This study was conducted in accordance with the Declaration of Helsinki and approved by the Joint Institutional Review Board of Taipei Medical University (N202011030).

Consent for publication

This study was approved by the Joint Institutional Review Board of Taipei Medical University (N202011030). The Joint Institutional Review Board of Taipei Medical University has waived the requirement of informed consent. All methods were performed in accordance with the relevant guidelines and regulations of the Declaration of Helsinki.

Competing interests

The authors declare that they have no competing interests in relation to this work.

Author information

[1]Graduate Institute of Injury Prevention and Control, College of Public Health, Taipei Medical University, Taipei City, Taiwan. [2]Department of Emergency Medicine, Taipei Medical University Hospital, Taipei City, Taiwan. [3]Department of Emergency Medicine, School of Medicine, College of Medicine, Taipei Medical University, Taipei City, Taiwan. [4]Department of Emergency Medicine, New Taipei City Hospital, New Taipei City, Taiwan. [5]College of Medicine, Chang Gung University, Taoyuan City, Taiwan. [6]Department of Emergency Medicine, Chang Gung Memorial Hospital, Linkou branch, Taoyuan, Taiwan.[7] Department of Emergency Medicine, Taipei Medical University-Shuang Ho Hospital, New Taipei City, Taiwan. [8]Taipei Neuroscience Institute, Taipei Medical University, Taipei City, Taiwan. [9]Division of Neurosurgery, Department of Surgery, Wan Fang Hospital, Taipei Medical University, Taipei City, Taiwan. [10]Department of Physical Medicine and Rehabilitation, Wan Fang Hospital, Taipei Medical University, Taipei City, Taiwan. [11]Transport Research Institute, Edinburgh Napier University, Edinburgh, Scotland. [12]Department of

Epidemiology, Biostatistics, and Population Health, Faculty of Medicine, Public Health and Nursing, Universitas Gadjah Mada, Yogyakarta City, Indonesia, [13]Department of Occupational Medicine, Chang Gung Memorial Hospital, Linkou branch, Taoyuan, Taiwan.

References:

1. Kjeldgard L, Ohlin M, Elrud R, Stigson H, Alexanderson K, Friberg E: Bicycle crashes and sickness absence - a population-based Swedish register study of all individuals of working ages. *BMC Public Health* 2019, *19*(1):943.
2. World Health Organization. Regional Office for E: Walking and cycling: latest evidence to support policy-making and practice. Copenhagen: World Health Organization. Regional Office for Europe; 2022,*1*.
3. Venkatraman V, Richard CM, Magee K, Johnson K: Countermeasures That Work: A Highway Safety Countermeasure Guide for State Highway Safety Offices, 10th Edition, 2020. 2021(DOT HS 813 097).
4. Allen-Munley C, Daniel J, Dhar S: Logistic Model for Rating Urban Bicycle Route Safety. *Transportation Research Record* 2004, 1878,*1*,:107-115.
5. Kaplan JA: Characteristics of the Regular Adult Bicycle User. Final Report; 1975.
6. Rivara FP, Thompson DC, Thompson RS: Epidemiology of bicycle injuries and risk factors for serious injury. *Inj Prev* 1997, 3,*2,*:110-114.
7. Wanvik PO: Effects of road lighting: an analysis based on Dutch accident statistics 1987-2006. *Accid Anal Prev* 2009, 41,*1*,:123-128.
8. Bicyclists and other cyclists: 2020 data (Traffic Safety Facts. Report No. DOT HS 813 322). In. https://crashstats.nhtsa.dot.gov/Api/Public/ViewPublication/813322: National Highway Traffic Safety Administration; 2022.
9. Bil M, Bilova M, Muller I: Critical factors in fatal collisions of adult cyclists with automobiles. *Accid Anal Prev* 2010, 42,*6,*:1632-1636.
10. Moore DN, Schneider WHt, Savolainen PT, Farzaneh M: Mixed logit analysis of bicyclist injury severity resulting from motor vehicle crashes at intersection and non-intersection locations. *Accid Anal Prev* 2011, 43,*3,*:621-630.
11. Wachtel A, Lewiston D: Risk factors for bicycle-motor vehicle collisions at intersections. *ITE journal* 1994, 64,*9,*:30-35.
12. Meuleners LB, Fraser M, Johnson M, Stevenson M, Rose G, Oxley J. Characteristics of the road infrastructure and injurious cyclist crashes resulting in a hospitalisation. *Accid Anal Prev.* 2020;136:105407.
13. Ugan J, Abdel-Aty M, Cai Q, Mahmoud N, Al-Omari Me: Effect of Various Speed Management Strategies on Bicycle Crashes for Urban Roads in Central Florida. *Transportation Research Record: Journal of the Transportation Research Board* 2021, 2676:036119812110366.
14. Robartes E, Chen TD: The effect of crash characteristics on cyclist injuries: An analysis of Virginia automobile-bicycle crash data. *Accid Anal Prev* 2017, 104,*1,*:165-173.
15. Pai CW: Overtaking, rear-end, and door crashes involving bicycles: an empirical investigation. *Accid Anal Prev* 2011, 43,*1,*:1228-1235.
16. Debnath AK, Haworth N, Schramm A, Heesch KC, Somoray K: Factors influencing noncompliance with bicycle passing distance laws. *Accid Anal Prev* 2018, 115,*1,*:137-142.
17. Maldonado G, Greenland S: Simulation study of confounder-selection strategies. *Am J Epidemiol* 1993, 138,*11,*:923-936.
18. Liu J, Jones S, Adanu EK, Li X: Behavioral pathways in bicycle-motor vehicle crashes: From

contributing factors, pre-crash actions, to injury severities. *J Safety Res* 2021, 77*,1,*:229-240.

19. Walker I: Signals are informative but slow down responses when drivers meet bicyclists at road junctions. *Accid Anal Prev* 2005, 37*,6,*:1074-1085.
20. Kay JJ, Savolainen PT, Gates TJ, Datta TK: Driver behavior during bicycle passing maneuvers in response to a Share the Road sign treatment. *Accid Anal Prev* 2014, *70*:92-99.
21. Anstey KJ, Horswill MS, Wood JM, Hatherly C: The role of cognitive and visual abilities as predictors in the Multifactorial Model of Driving Safety. *Accid Anal Prev* 2012, *45*:766-774.
22. Kandasamy D, Betz ME, DiGuiseppi C, Mielenz TJ, Eby DW, Molnar LJ, Hill L, Strogatz D, Li G: Self-reported health conditions and related driving reduction in older drivers. *Occup Ther Health Care* 2018, 32*,4,*:363-379.
23. Laosee O, Rattanapan C, Somrongthong R: Physical and cognitive functions affecting road traffic injuries among senior drivers. *Arch Gerontol Geriatr* 2018, *78*:160-164.
24. Cicchino JB, Wells JK, McCartt AT: Survey about pedestrian safety and attitudes toward automated traffic enforcement in Washington, D.C. *Traffic Inj Prev* 2014, 15(4):414-423.
25. Kostyniuk LP, Molnar LJ: Self-regulatory driving practices among older adults: health, age and sex effects. *Accid Anal Prev* 2008, 40*,4,*:1576-1580.
26. Zhang J, Lindsay J, Clarke K, Robbins G, Mao Y: Factors affecting the severity of motor vehicle traffic crashes involving elderly drivers in Ontario. *Accid Anal Prev* 2000, 32*,1,*:117-125.
27. Edwards JD, Ross LA, Wadley VG, Clay OJ, Crowe M, Roenker DL, Ball KK: The useful field of view test: normative data for older adults. *Arch Clin Neuropsychol* 2006, 21*,4,*:275-286.
28. Wood JM, Lacherez PF, Marszalek RP, King MJ: Drivers' and cyclists' experiences of sharing the road: incidents, attitudes and perceptions of visibility. *Accid Anal Prev* 2009, 41*,4,*:772-776.
29. W.~Hunter W, Stewart JR: An Evaluation of Bike Lanes Adjacent to Motor Vehicle Parking. 1999.
30. Huang C-Y: Observations of Drivers' Behavior when Opening Car Doors. *Procedia Manufacturing* 2015, *3*:2753-2760.
31. Traffic Safety Facts - Bicyclists and Other Cyclists. In. https://crashstats.nhtsa.dot.gov/Api/Public/ViewPublication/813322: National Highway Traffic Safety Administration; 2020.

 Click here to access/download;Figure;Figure 1.docx

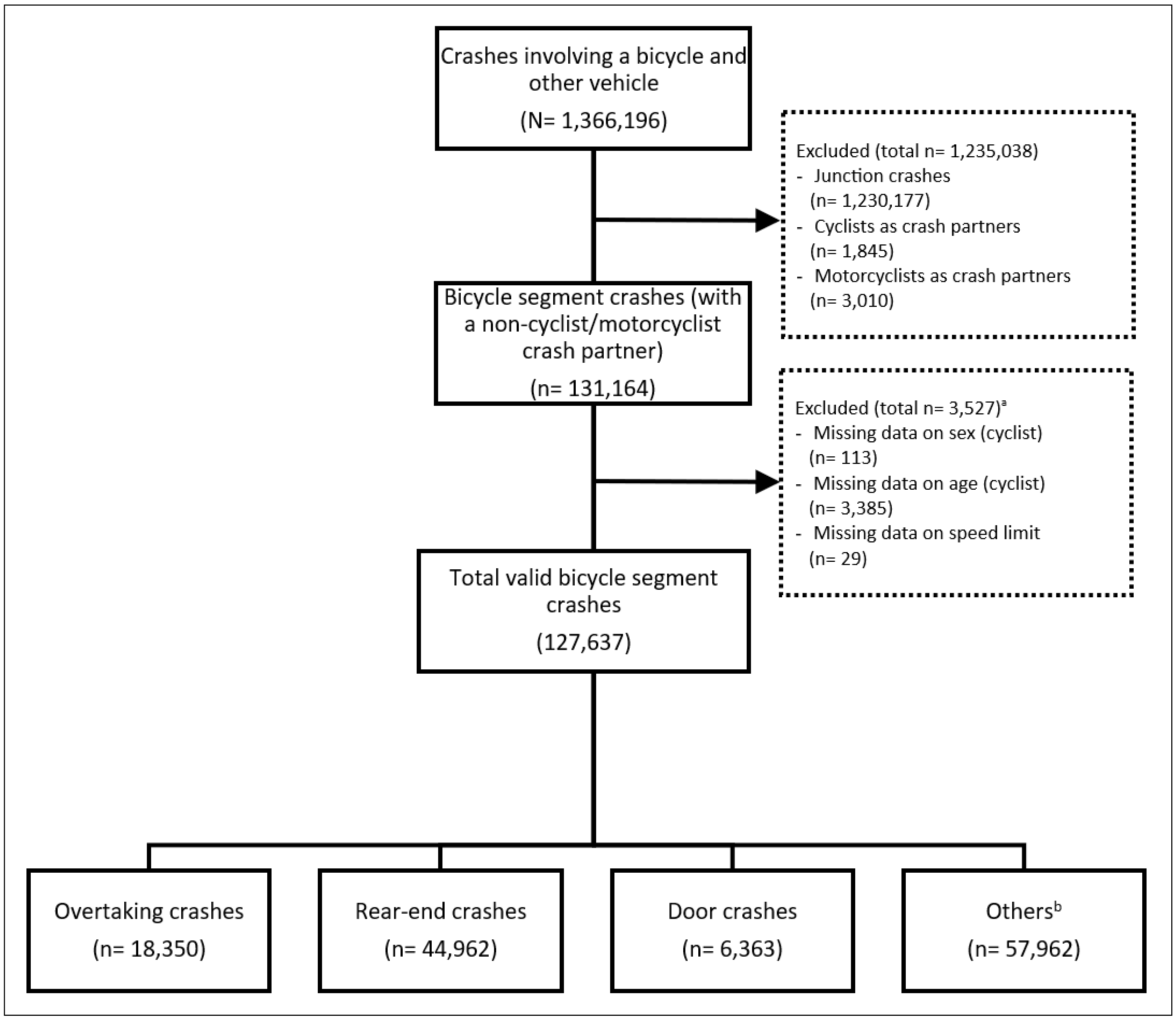

Fig. 1. Flowchart of the study sample selection process. [a]Listed excluded criteria are nonexclusive; thus, the sum of the total may exceed 3,527. [b]Other crashes include reversing crashes and head-on crashes.

Figure 2 Click here to access/download;Figure;Figure 2.docx

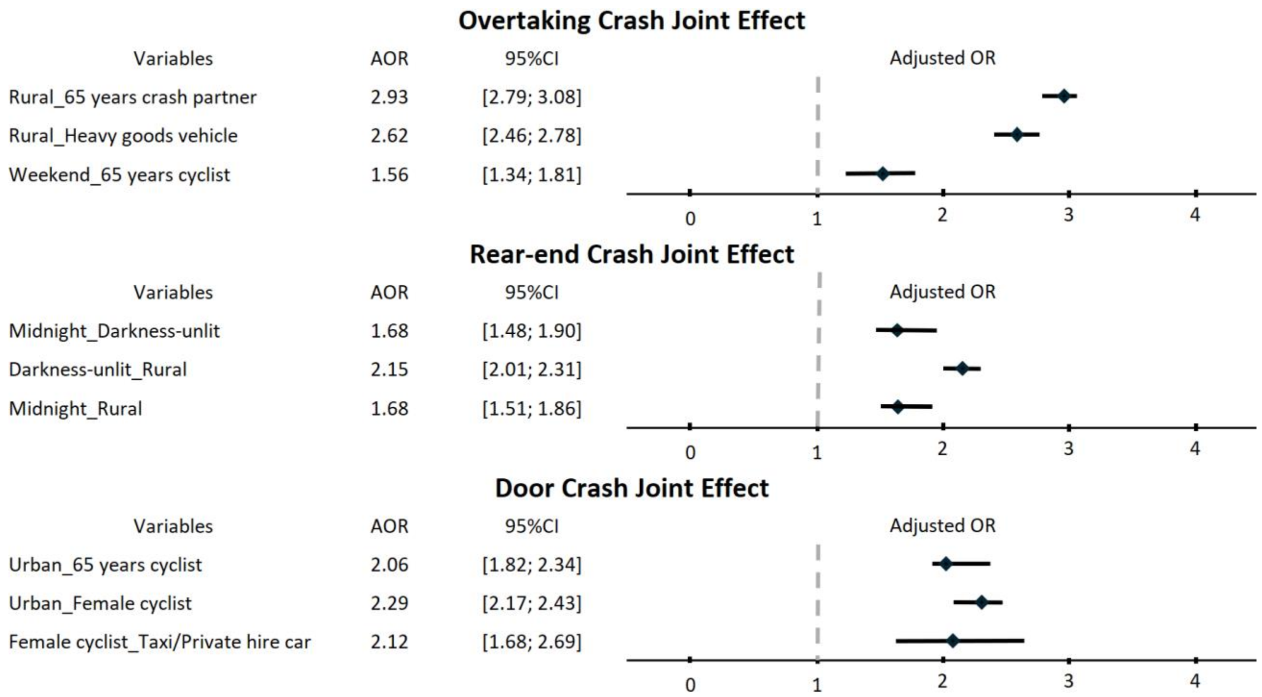

Fig. 2. Joint effects of several variables on the three crash types.
