# Supplementary material for: Risk factors for overtaking, rear-end, and door crashes involving bicycles in the United Kingdom: Revisited and reanalysed
Source: PLoS One. 2025 Jan 3;20(1):e0315692. doi: 10.1371/journal.pone.0315692 (PMC11698528; doi:10.1371/journal.pone.0315692)
Supplement: Attachment — Submitted filename: Reviewer_comment1.pdf [file pone.0315692.s002.pdf]

Reviewer's Responses to Questions

Abstract

The abstract is lacking the background section, please see the comment on the pdf

Introduction

The authors did not explain the context of the previous study, where this current study was based, but only cited it. For my comments also see the pdf with my comments on this section

Methodology

The method section was described well and is adequate, although we need to know whether normality checks were conducted.

Results

This area still requires more work. The way the results were presented was hasty, and we need to redo some of the highlighted sections. For example, a separate Univariate table is needed as Table 1.

Furthermore, the authors need to interpret the Bivariate results for all the significant variables, rather than summarizing them.

Discussion

The section also needs serious work, especially on the way the findings were discussed. The authors should consider discussing their own findings rather than those of other studies.

There is also a need to have a section for recommendations rather than merging it within result discussions.

They need to tell us how they tried to minimize the biases that could have been introduced by the many study limitations identified for this study.

Overall comments

Other than that, the article has the potential to contribute to knowledge on overtaking crashes and bicycle safety around the globe.
