# Supplementary material for: Risk factors for overtaking, rear-end, and door crashes involving bicycles in the United Kingdom: Revisited and reanalysed
Source: PLoS One. 2025 Jan 3;20(1):e0315692. doi: 10.1371/journal.pone.0315692 (PMC11698528; doi:10.1371/journal.pone.0315692)
Supplement: Attachment — Submitted filename: 7-31 PLOS one Reviewer all 07312024 PAI.pdf [file pone.0315692.s003.pdf]

Dear Editors and Reviewers,

We greatly appreciate the valuable comments and suggestions raised by reviewers. Please very kindly see our responses below, as well as the revised manuscript. We would be glad if you could have our manuscript reviewed again.

Best regards,

Chih-Wei Pai (Prof)

Graduate Institute of Injury Prevention and Control College of Public Health

Taipei Medical University

Reviewer comments:

Reviewer 1: Regarding the statistical analysis, I would like to ask the authors to explain:
1. the reason(s) for ignoring any probable interaction between independent variables in the multivariate logistic regression.

Author's response: We appreciate the reviewer's comment and question. By examining variables independently, we gain a clearer understanding of their individual impacts on the outcome (specifically, crash type in this study). This approach allows us to assess each variable's direct influence without the added complexity of interactions or modifications between variables. It provides insights into which variables independently affect the outcome, directly addressing our research questions. Initially, we used the chi-squared test to explore associations between a set of independent variables and the three crash types. To minimize type II errors in variable selection and ensure unbiased inferences, we included variables with a p-value less than 0.2 from the univariate analysis into the multivariate logistic regression models, a common practice in past studies of traffic injuries (e.g., a, b) and methodology (c). Subsequently, we examined interaction effects among several variables of interest, as depicted in Figure 2 of the manuscript. While acknowledging the potential for other interactions among variables, our study focused on assessing the joint effects of specific variables of interest. To take overtaking crashes as an example, these variables included rural areas, crash partners aged 65 years or older, heavy goods vehicles, weekends, and cyclists aged 65 years or older. Future research could delve into untangling the complexities of additional interaction effects among variables, as suggested by the reviewer.

References:

a: Chen, P-L, Pai, C-W. Evaluation of injuries sustained by motorcyclists in approach-turn crashes in Taiwan. Accident Analysis and Prevention, 2019, 124, 33-39.

b: Chien, D-K., Hwang, HF, Lin, MR. Injury severity measures for predicting return-to-work after a traumatic brain injury. Accident Analysis and Prevention, 2017, 98, 101-107.

c: Maldonado G, Greenland S. Simulation study of confounder-selection strategies. Am J Epidemiol 1993, 138, 11, 923-936.

2. Why did they consider different reference categories for the same individual variables among different outcomes in logistic regression modeling? This will make it difficult to interpret the comparison of the effect of an independent variable on different types of crashes. for example, in table 4, the ref category for Light condition is

Darkness-lit, Daylight and Darkness-unlit for Overtaking, Rear-end and Door crashes respectively.

Author's response: We appreciate the reviewer's comment and question. In our analysis, we chose various reference categories for variables based on the lowest Adjusted Odds Ratios (AORs) observed. This approach allowed us to highlight different risk factors associated with higher AORs for specific types of crashes. For example, urban roads with speed limits of 20-30 mph were identified as protective factors for overtaking and rear-end crashes. However, for door crashes, these urban roads appeared to pose a higher risk compared to rural roads, as indicated by their higher AOR. It is important to note that selecting a reference category does not change the estimation results of our models. Instead, assigning reference case with the lowest AOR helps readers identify risk factors with higher AORs among the three crash types.

3. I suggest authors provide identical indicators for figures both in the main text and in the figure's caption. Reading "Fig. 1" below a figure, one will look for the same word in the main text while it is recalled as "Figure 1".

Author's response: We appreciate this reviewer's comments, and we have revised the manuscript in the main text and figure's caption (please refer to lines 145 to 146; page 8 in the manuscript).

Reviewer 2:
1 General comments:
1.1 None of the authors was from the UK???

Author's response: We appreciate this reviewer's comments. One of our authors, Prof. Wafaa Saleh, is from Edinburgh Napier University, UK.

1.2 The authors should emphasize the significance of including these three types of crashes????

Author's response: We appreciate the reviewer's comments. We have incorporated the following statements into the introduction to underscore the significance of including the three crash types (please refer to lines 110 to 115; pages 5-6 in the manuscript):

> "The study addresses a critical gap in current research, focusing on crashes specifically occurring on road segments. Existing literature offers limited insights into this specific type of crash, highlighting a crucial need for targeted investigation. These crashes have the potential for severe impact, involving complex dynamics that demand a nuanced understanding for effective mitigation strategies. By exploring these factors, our research aims to significantly enhance cyclist safety within this particular context."

1.3 What novelty this study adds compared to the previous one in 2011???

Author's response:

We appreciate this reviewer's comment. One inherent problem with police-reported crash data is the variables not readily available, hereby causing unobserved heterogeneity across the observations. To overcome such a limitation, we estimated separate regression models, as suggested by Kim et al. (e.g., d), for the three crash types; such an approach provides greater explanatory power compared to single overall models. Further, we conducted joint-effect analyses of several variables of interest that capture heterogeneity. In our previous studies, we adopted the above-mentioned approaches to overcome the inherent problem with a success (e.g., e, f).

To clarify this, the following statements have been added to the Discussion section of the manuscript (please refer to lines 391 to 397; page 23 in the manuscript):

> "One inherent problem with police-reported crash data is the variables not readily available, hereby causing unobserved heterogeneity across the observations. To overcome such a limitation, we estimated separate regression models, as

suggested by Kim et al. (e.g., d), for the three crash types; such an approach provides greater explanatory power compared to single overall models. Further, we conducted joint-effect analyses of several variables of interest that capture heterogeneity. In our previous studies, we adopted the above-mentioned approaches to overcome the inherent problem with a success (e.g., e, f)."

d: Kim, D., Washington, S., Oh, J., 2006. Modelling crash outcomes: new insights into the effects of covariates on crashes at rural intersections. Journal of Transportation Engineering. 132 (4), 282-292.

e: Pai CW, Jou RC, 2014. Cyclists' red-light running behaviours: An examination of risk-taking, opportunistic, and law-obeying behaviours. Accident Analysis and Prevention. 62,191-198.

f: Pai CW, Saleh W., 2008. Modelling motorcyclist injury severity by various crash types at T-junctions in the UK. Safety Science. 13, 98-98.

1.4 The rationale for conducting the current study as well as the practical implications should be emphasized??

<u>Author's response:</u> We appreciate this reviewer's comments. First, regarding the rationale for conducting the current study, we have added the following statements (please kindly refer to lines 91-95 on page 5 of the manuscript):

> "Bicycle crashes on road segments remain a substantial issue for public health concern. Existing research primarily emphasizes intersection-related crashes. This study aims to fill a critical gap by conducting a thorough examination of the risk factors associated with three distinct bicycle crash types: overtaking, rear-end, and door crashes that occur on road segments."

Secondly, to highlight the practical implications, we have included the following statements in the Discussion section (please refer to lines 404-412 on pages 23-24 of the manuscript):

> "Recommendations
>
> For overtaking crashes, we recommend implementing 'Share the Road' warning signs, especially in rural areas, and developing specialized cognitive training programs for elderly drivers. Regarding rear-end crashes, our suggestions include improving illumination during night time and implementing speed control measures on rural road segments. For door crashes involving parked cars, we propose enhancing driver sight triangles and increasing cyclist visibility. Moreover, implementing a two-stage door opening mechanism and an automatic

> detection device in vehicles to alert drivers of bicycles approaching from behind could potentially be beneficial.”

1.5 For the introduction section, burden in terms of mortality, morbidity, and DALYs should be mentioned as well the economic and health care costs should be mentioned (globally and UK)

Author’s response: We appreciate the reviewer’s comments. Our original literature review has included several past studies that have reported the accident/injury outcomes resulting from these three crash types. For example, road segments with elevated speed limits, male cyclists, and cyclists aged over 55 years contribute significantly to high injury severity crashes. Additionally, built-up areas increase the risk of door crashes involving cyclists and parked cars.

It is important to note that there is limited research specifically examining the impact of overtaking, rear-end, and door crashes on Disability-Adjusted Life Years DALYs, economic costs, and healthcare expenses. Notable exceptions include studies by Elvik and Sundfør (e.g., d), who examined the inclusion of cyclist injuries in health impact economic assessments. Aertsens et al. (e.g., h) and Scholten et al. (e.g., i) also provided comprehensive analyses of the total and average costs associated with bicycle injuries. Although the three crash types were not explicitly examined in the above-mentioned studies, we have followed this reviewer’s suggestion by incorporating these studies into the 'Introduction' section (please refer to lines 77-81; page 4 of the manuscript):

> “Bicycle crashes can also impose a significant burden on healthcare expenses. Elvik and Sundfør (e.g., g) have discussed the economic implications and healthcare expenditures associated with bicycle accidents. For instance, in Belgium, the average cost of bicycle accidents per case is estimated at 841 euros (e.g., h). In the Netherlands, the total annual cost has been reported as €410.7 million (e.g., i).”

References:

g: Elvik, R., & Sundfør, H. B. (2017). How can cyclist injuries be included in health impact economic assessments? Journal of Transport & Health, 6, 29-39.
h: Aertsens, J., de Geus, B., Vandenbulcke, G., Degraeuwe, B., Broekx, S., De Nocker, L., ... & Panis, L. I. (2010). Commuting by bike in Belgium, the costs of minor accidents. Accident Analysis & Prevention, 42(6), 2149-2157.

i: Scholten, A. C., Polinder, S., Panneman, M. J., Van Beeck, E. F., & Haagsma, J. A. (2015). Incidence and costs of bicycle-related traumatic brain injuries in the Netherlands. Accident Analysis & Prevention, 81, 51-60.

1.6 The number of cyclists in UK or those using bicycles for their mobility??

Author's response: We appreciate the reviewer's comment. In our study, we analyzed national police-reported crash data involving cyclists. Unfortunately, exposure data, such as the number of cyclists and miles traveled, were not available in the STATS19 dataset. While such data may be available from the UK National Travel Survey, it often reflects outdated information and may not be fully representative of the entire population.

2. Specific comments:
2.1 Instead of data collection, data used for analysis is appropriate??

Author's response: We appreciate the reviewer's comment. The dataset, UK Stats19 covering all traffic accidents in the UK, should be appropriate, as numerous studies in the field of traffic injury and medicine have analysed such data (e.g., references j, k, l).

j: Haghpanahan, Houra, et al. "An evaluation of the effects of lowering blood alcohol concentration limits for drivers on the rates of road traffic accidents and alcohol consumption: a natural experiment." *The Lancet* 393.10169 (2019): 321-329.

k: Pai, C. W., Hwang, K. P., & Saleh, W. (2009). A mixed logit analysis of motorists' right-of-way violation in motorcycle accidents at priority T-junctions. Accident Analysis & Prevention, 41(3), 565-573.

l: Fountas, G., Fonzone, A., Gharavi, N., & Rye, T. (2020). The joint effect of weather and lighting conditions on injury severities of single-vehicle accidents. *Analytic methods in accident research*, *27*, 100124.

2.2 Of the used crashes data, how many were fatal???

Author's response: We appreciate the reviewer's comment. As reported in the table below, as many as 0.8% of those in overtaking crashes sustained fatal injuries, which was the highest compared to those in the other two crash types.

	Slight	Serious	Fatal	Total
Overtaking crashes	14240(77.6%)	3,964(21.6%)	147(0.8%)	18350

Rear-end crashes	39821(89.1%)	4782(10.7%)	89(0.2%)	44692
Door crashes	5561(87.4%)	770(12.1%)	32(0.5%)	6363

2.3 For analysis of data, use the Odds ratios and 95% confidence intervals (univariate and bivariate)

<u>Author's response:</u> We appreciate this reviewer's comment. We analyzed the distribution of crash types across a set of independent variables. Chi-square tests were used to explore relationships between these variables and crash types. Variables with a significance level below 0.2 were identified to minimize type II errors and were considered significantly associated with the outcome variables ($p < 0.05$). Subsequently, these variables were included in multiple logistic regression models. Stepwise logistic regression was then employed to estimate the odds of various variables after controlling for specific factors. This methodology has been widely used in past studies of traffic injuries (e.g., a, b) and methodology (e.g., c).

a: Chen, P-L, Pai, C-W. Evaluation of injuries sustained by motorcyclists in approach-turn crashes in Taiwan. Accident Analysis and Prevention, 2019, 124, 33-39;

b: Chien, D-K., Hwang, HF, Lin, MR. Injury severity measures for predicting return-to-work after a traumatic brain injury. Accident Analysis and Prevention, 2017, 98, 101-107;

c: Maldonado G, Greenland S. Simulation study of confounder-selection strategies. Am J Epidemiol 1993, 138, 11, 923-936).

2.4 Details about the multivariate logistic regression model should be mentioned???
Use the Odds ratios for interpreting and displaying the results in tables 1, 2, and 3???

<u>Author's response:</u> We appreciate the reviewer's comment. Firstly, if we understand this reviewer correctly, we have incorporated additional details (such as formulation and derivation) of the multivariate logistic regression model into the "Methods" section (please refer to lines 179-194 on pages 10-11 of the manuscript):

> "Initially, we examined the distribution of three crash types across various variables to explore their relationships with a binary outcome. These variables included lighting conditions, speed limit, time of day, and day of the week. Demographic details concerning cyclist casualties encompassed age and sex, while information about the crash partner included vehicle type, age, and sex. We set a significance level of p <

0.2 to include risk factors in our multivariate analysis. Adjusted odds ratios (AORs) were computed using multivariate logistic regression with backward selection.

The multivariate logistic regression model equation was specified as:

$$log\left(\frac{P(Y = 1)}{1 - P(Y = 1)}\right) = \beta_0 + \beta_1 X_1 + \beta_2 X_2$$

where $P(Y = 1)$ denotes the probability of the outcome, $\beta_0, \beta_1, \beta_2, \ldots, \beta_p$ are the coefficients to be estimated, and $X_1, X_2, \ldots, X_p$ represent the predictor variables.

Before estimating the model, assumptions of logistic regression, such as linearity of the logit, absence of multicollinearity, and independence of observations, were evaluated.

An odds ratio (OR) greater than 1 indicated a positive association between the independent variable and the occurrence rate, while an OR less than 1 indicated a negative association. An OR of 1 suggested no association between the variables of interest and the outcomes."

Secondly, this reviewer suggested that we should use the Odds ratios for interpreting and displaying the results in tables 1, 2, and 3. While we acknowledge this suggestion, we would like to clarify here that we adopted the commonly-used Chi-square tests to identify the distribution of three crash types across several independent variables. Instead of the univariate logistic regression, such a method has been proved as an efficient way to minimize type II errors, and has been widely employed in past studies of traffic injuries (e.g., a, b) and methodology (e.g., c).

a: Chen, P-L, Pai, C-W. Evaluation of injuries sustained by motorcyclists in approach-turn crashes in Taiwan. Accident Analysis and Prevention, 2019, 124, 33-39;

b: Chien, D-K., Hwang, HF, Lin, MR. Injury severity measures for predicting return-to-work after a traumatic brain injury. Accident Analysis and Prevention, 2017, 98, 101-107;

c: Maldonado G, Greenland S. Simulation study of confounder-selection strategies. Am J Epidemiol 1993, 138, 11, 923-936).

2.5 Chi square is not enough test to identify the direction and which segment of the given variable is significantly different???

Author's response: We appreciate this reviewer's comment. The reviewer is correct. Chi-square tests can be used for ascertaining the association of the dependent and independent variables. However, the direction of the independent variables can be untangled in the subsequent multivariate logistic regression models.

2.6 What was the adjustment made for??? And how???

Author's response: We appreciate this reviewer's comment. Each variable was adjusted for in the multivariate analysis. For instance, in Table 4, adjustments were made for crash day after accounting for other variables such as cyclist's sex, crash partner, and crash partner's age and sex.

2.7 The joint-crash effect: how it was measured statistically???

Author's response: Thank you for your valuable comment. We do apologize for not making our analysis clear. To clarify how joint-effect analysis was structured, we drew several figures below that help us respond to this reviewer.
As Figure A1 (X axis: speed limit; Y axis: percentage) and A2 report (X axis: Crash partner's age; Y axis: percentage), the joint effects of speed limit (two categories: rural (≥ 40 mph) /urban (20–30 mph)] and crash partner's age (four categories: ≤18, 19–40, 41–64, and ≥65) on overtaking crashes were examined, yielding eight combinations of interaction effects (i.e., 1. Rural x ≤18; 2. Rural x 19-40; 3. Rural x 41-64; 4. Rural x ≥65; 5. Urban x ≤18; 6. Urban x 19-40; 7. Urban x 41-64; 8. Urban x ≥65). All percentages of overtaking crashes among these eight combinations were compared, and the combination with the highest percentages for overtaking crashes is taken as the indicator variable. In this joint-effect analysis, the indicator variable "rural areas x crash partner's ≥65 years old" has the highest percentage of overtaking crashes. These results elucidated that overtaking crashes were more likely to occur when the cyclists were in rural areas and when involving ≥65-year-old crash partners.

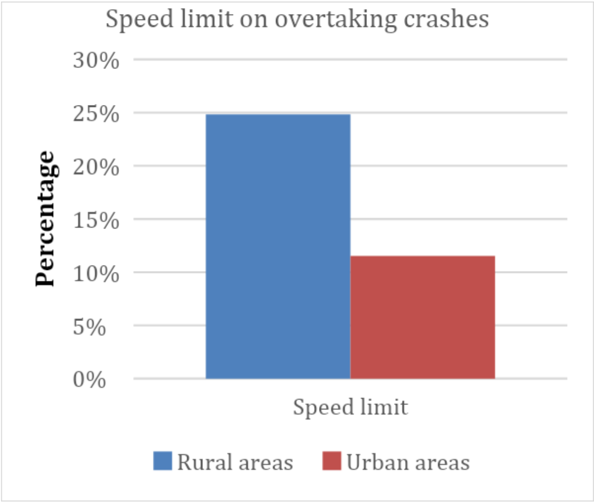

Figure A1

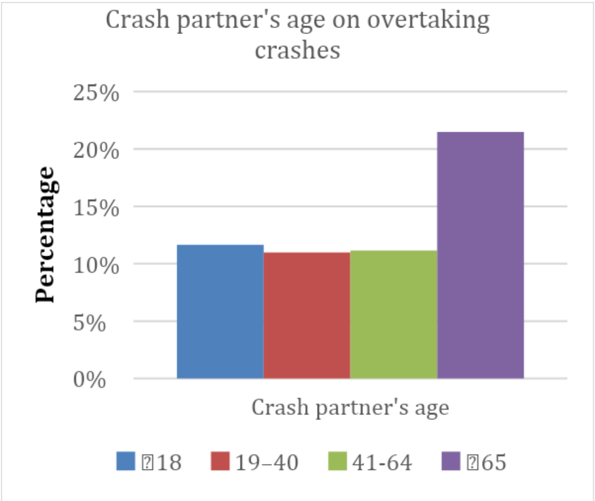

Figure A2

In practice, such a joint-effect analysis has been widely employed in medicine or traffic injury literature. One well-known paper by Weinstein et al. (i.e., m) was published in JAMA which examined the joint effect of physical activity and body mass index on diabetes in women. In this paper, Weinstein et al. pointed out that the beneficial effect of active lifestyle on type 2 diabetes was consistent across women with three BMI levels.

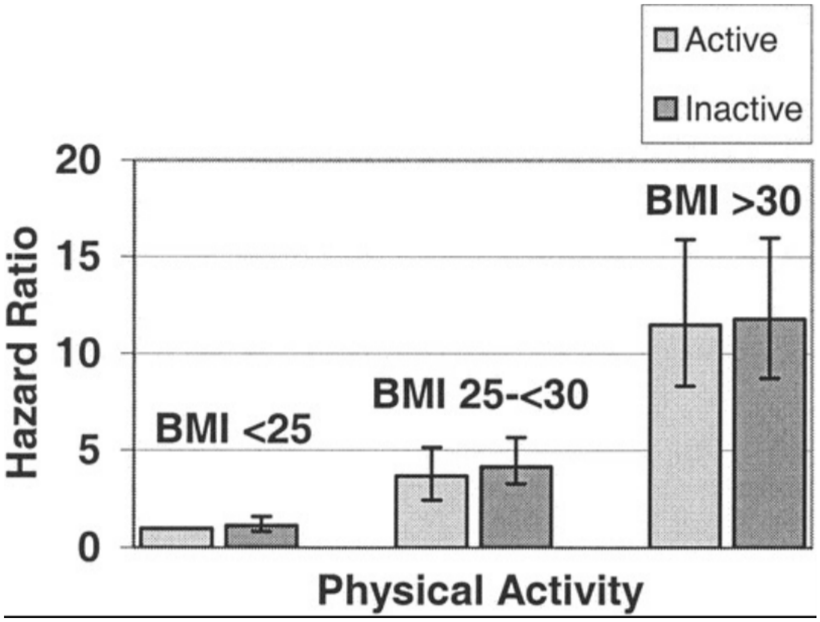

Another example is our previous paper published in Accident Analysis and Prevention in 2020 titled: Evaluating the combined effect of alcohol-involved and un-helmeted riding on motorcyclist fatalities in Taiwan. In this study, we specifically analysed the joint effect of alcohol use and helmet use on motorcyclist fatalities (i.e., n).

Interaction results for motorcyclist's alcohol use with motorcyclist's helmet use.

Interaction term	Odds ratio	p value	95 % CI
Blood alcohol level with helmet use[a]			
1 Blood alcohol positive and not using helmet	18.1	< 0.001	15.9 – 20.4
2 Blood alcohol positive and using helmet	10.1	< 0.001	9.3 – 11.1
3 Blood alcohol negative and not using helmet	2.3	< 0.001	2.1 – 2.5
4 Blood alcohol negative and using helmet	1	–	1

In addition, our previous paper published in BMC Public Health in 2023 titled: Walking against traffic and pedestrian injuries in the United Kingdom: new insights (i.e., o). In this study, we specifically analysed the joint effect to examine whether the beneficial effect of walking against traffic on injury severity may apply to different situations. By doing so, we were able to compare injury outcomes in walking against-traffic crashes against those in walking with-traffic crashes.

Variables	AOR	95% CI
With traffic_Darkness unlit	1.48	1.29-1.70
With traffic_Midnight	1.60	1.37-1.87
With traffic_Rural area	2.20	1.92-2.51
With traffic_Elderly pedestrian	2.65	2.16-3.26
With traffic_HGVs	1.51	1.28-1.78

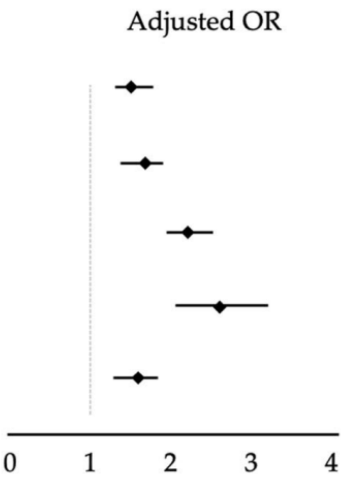

Fig. 2 Joint effects of walking with traffic and other variables on pedestrian fatalities

We believe this detailed explanation clarifies our methodology.

Reference:

m: Weinstein A., Sesso, H., Lee, I., Cook, N., Manson, J., Buring, J., Gaziano, J., 2004. The relationship of physical activity vs body mass index with type 2 diabetes in women. JAMA 290: 1188-1194.

n: Wiratama, B., Chen, P., Ma, S., Chen, Y., Saleh, W., Lin, H., Pai, C., 2020. Evaluating the combined effect of alcohol-involved and un-helmeted riding on motorcyclist fatalities in Taiwan. Accident Analysis and Prevention, 143, 105594.

o: Widodo, A. F., Chen, C., Chan, C. W., Saleh, W., Wiratama, B. S., & Pai, C. W. (2023). Walking against traffic and pedestrian injuries in the United Kingdom: new insights. BMC public health, 23(1), 2205.

Reviewer #3: Areas for Improvement:
3.1 Clarity and Conciseness:

Some sections of the text are verbose and could benefit from more concise language. For instance, the detailed descriptions of statistical methods and results could be streamlined without losing essential information.
Simplifying the language and structure would enhance readability and accessibility, particularly for readers who are not specialists in the field.

Author's response: We appreciate the reviewer's valuable suggestions. Concerning two reviewers who recommended extending several sections (i.e., reviewer #2 asked us to explain more on multivariate regression models and reviewer #4 requested for further discussions), we maintained a neutral stance for the time being. Nonetheless, we have revised the introduction to provide a clearer context and expanded our descriptions in the discussion section to provide broader insights into the implications of our findings. Additionally, detailed descriptions of the statistical methods have been included in the methods section, aimed at enhancing readability and accessibility for our readers.

3.2 Detailed Interpretation of Results:

While the results section provides extensive data, there is limited interpretation of what these results mean in practical terms. Adding more context about how these findings could influence policy or infrastructure design would be valuable. Discussing potential interventions based on the identified risk factors, such as specific infrastructure improvements or policy changes, would strengthen the practical implications of the study.
Author's response: We appreciate the reviewer's comment and suggestion. We have revised the discussion section of the manuscript and added one recommendation section to address findings that could potentially influence policy or infrastructure as follows (please refer to lines 404-412 on pages 23-24 of the manuscript):

> "Recommendations
>
> For overtaking crashes, we recommend implementing 'Share the Road' warning signs, especially in rural areas, and developing specialized cognitive training programs for elderly drivers. Regarding rear-end crashes, our suggestions include improving illumination during night time and implementing speed control measures on rural road segments. For door crashes involving parked cars, we propose enhancing driver sight triangles and increasing cyclist visibility. Moreover, implementing a two-stage door opening mechanism and an automatic

detection device in vehicles to alert drivers of bicycles approaching from behind could potentially be beneficial.”

3.3 Comparative Analysis:
Including a comparative analysis with similar studies from other countries could provide a broader context for the findings and highlight whether these risk factors are unique to the UK or consistent globally.
Discussing how the UK’s findings compare with those from the United States or other European countries, especially concerning the impact of infrastructure and vehicle types, could offer valuable insights:

Author’s response: We appreciate the reviewer’s comment. To our knowledge, no comparative analysis from other countries has been conducted for the three crash types (overtaking, rear-end, and door crashes). In addition, it is out of the scope of the current research to obtain crash data from other countries and conduct a large scale of comparative analysis. However, in our introduction sections, we have reviewed previous studies that focused on risk factors for these crash types individually or collectively on road segments (such as vehicle volume, traffic density, and number of lanes).

In the discussion section, we have discussed our findings with those of other studies in the US or elsewhere. For instance, previous analyses of overtaking crashes highlighted risk factors such as speeds exceeding 10 mph and the presence of pick-up trucks. Rear-end crashes were associated with conditions such as darkness, unlit surroundings, midnight hours, and reduced cognitive capabilities. Door crashes were found to be influenced by factors including urban roadways and the presence of taxis.

3.4 Providing more detailed information about the methodology, particularly the criteria for excluding certain data points, would enhance transparency. For example, explaining why specific demographic data were incomplete and how this might affect the results would be useful.
A discussion on the limitations of the data and the potential biases introduced by police reporting practices could provide a more nuanced understanding of the findings.

Author’s response: We appreciate the reviewer’s comment. To clarify the reasons for excluding junction cases and cyclists/motorcyclists as crash partners, we have added the following statements in the Methods section (please also kindly see lines 168 to 174; pages 9-10 in the manuscript):

“On a cautionary note, we removed junction cases to avoid the variability introduced when exogenous factors, such as junction geometry and control measures, are present at junctions. Furthermore, the cases involving other cyclists and motorcyclists were removed as we focused on vehicle-cycle crashes only. Missing data on sex, age, or speed limits were also excluded in the analysis. Excluding these data may impact our results in a marginal scale, as these data are likely to be single-bicycle crashes that in nature be underreported in police crash dataset [e.g., p]. “

Regarding the limitation of police reported crash data, the following statements have been added to the manuscript (please also kindly see lines 378 to 391; pages 22-23 in the manuscript):

“This study had several limitations that warrant acknowledgement. First, the substantial underreporting of nonfatal casualties to the police, particularly casualties involving cyclists not obligated to report accidents, is a critical factor to consider. Such underreporting, as highlighted by the U.K. Government’s Department for Transport, likely results in the incomplete representation of nonfatal and ‘slight’ casualties in road casualty data. Second, the STATS19 data utilised in this study lack critical variables, including precrash speeds, specific geometric characteristics of roadways, data regarding alcohol and illicit substance use, and cyclist speed at the time of an accident. Moreover, critical exposure data—such as those related to traffic flow, rider or driver experience, and other elements of risk exposure—are absent, and the absence of such details limits our ability to fully account for potential variations resulting from unobserved factors in the analyses. Finally, this study did not explore annual trends in each type of bicycle crash over the 30-year study period; investigating such trends could provide insights regarding changing behaviours among cyclists and motor vehicle drivers as well as the effects of legislative changes for road speed limits.”

p. Watson, Angela, Barry Watson, and Kirsten Vallmuur. "Estimating under-reporting of road crash injuries to police using multiple linked data collections." *Accident Analysis & Prevention* 83 (2015): 18-25.

3.4 Visual Aids:

Adding more visual aids, such as graphs or charts, could help in visualizing the key findings and making the data more accessible to readers.
A geographic distribution map showing where different types of crashes are more prevalent could add an interesting dimension to the analysis.

<u>Author's response:</u> We appreciate the reviewer's suggestions. We firstly reported our sampling by using a flowchart that helps readers understand what data were excluded and included in the analyses. Although we presented our statistical analyses in a traditional way (Tables 1 to 4), we illustrated a forest plot demonstrating the joint effects of several variables on the three crash types when other variables were controlled for (please refer to lines 213 to 276; pages 12-17 in the manuscript).

Regarding the geographic distribution map illustrating where these crash types were more prevalent, our research objective does not primarily emphasize the geographic effects of these three crash types. Rather, we focused on identifying risk factors for these crash types. While we appreciate this reviewer's valuable comment on this, we have identified this as an important research area as follows (please refer to lines 398 to 402; page 23 in the manuscript):

> "Future research directions could involve integrating GPS (Global Positioning System) data and weather conditions to analyse both the injury frequency and fatalities of bicycle crashes on road segments. Additionally, exploring the potential of autonomous vehicles for detecting approaching bicycles for door-crashes and implementing AI-controlled lighting systems in rural areas for cyclist detection could be promising areas for further study."

3.5 Future Directions:
Including a section on future research directions would be beneficial. Identifying gaps in the current research and suggesting areas for further investigation could guide subsequent studies.
Discussing the potential impact of emerging technologies, such as autonomous vehicles and advanced cyclist detection systems, on these crash types could provide a forward-looking perspective.

<u>Author's response:</u> We appreciate the reviewer's comment and suggestion. We have revised the discussion section of the manuscript and added one future research section (please refer to lines 398 to 402; page 23 in the manuscript). Furthermore, we have added one new section "Recommendation" that reports potential intervention points (please refer to lines 404-412 on pages 23-24 of the manuscript):

> "Recommendations
>
> For overtaking crashes, we recommend implementing 'Share the Road' warning signs, especially in rural areas, and developing specialized cognitive training programs for elderly drivers. Regarding rear-end crashes, our suggestions include improving illumination during night time and implementing speed control

measures on rural road segments. For door crashes involving parked cars, we propose enhancing driver sight triangles and increasing cyclist visibility. Moreover, implementing a two-stage door opening mechanism and an automatic detection device in vehicles to alert drivers of bicycles approaching from behind could potentially be beneficial."

Future research directions:

"Future research directions could involve integrating GPS (Global Positioning System) data and weather conditions to analyse both the injury frequency and fatalities of bicycle crashes on road segments. Additionally, exploring the potential of autonomous vehicles for detecting approaching bicycles for door-crashes and implementing AI-controlled lighting systems in rural areas for cyclist detection could be promising areas for further study."

Reviewer #4: This Study is technically sound and has potential to add to the body of knowledge involving bicycle riding safety in the UK and everywhere across the globe. It has adhered to the research and publication ethics, however, the study still need revision on some of the key identified areas which i have pointed out, starting from abstract, background, results and discussions.

4.1 Abstract

The abstract is lacking the background section, please see the comment on the pdf

This abstract is lacking the background section, which must start when presenting structured abstract. Also there is no objective put here, but rather the research problem investigated.

Author's response: We appreciate the reviewer's comment and suggestion. We have revised the abstract to add background and objects as follows (please refer to lines 23 to 27; page 2 in the manuscript):

> "Background: Relevant research has provided valuable insights into risk factors for bicycle crashes at intersections. However, few studies have focused explicitly on three common types of bicycle crashes on road segments: overtaking, rear-end, and door crashes.
>
> Objective: This study aims to identify risk factors for overtaking, rear-end, and door crashes that occur on road segment."

4.1.1 Abbreviations should be defined when they are first mentioned

Author's response: We appreciate the reviewer's suggestion. We have revised the abstract to include the full definitions of abbreviations upon their first appearance as follows (please refer to lines 31; page 2 in the manuscript).

> "Abstract: AOR (adjusted odds ratio)"

4.2 Introduction

4.2.1 The authors did not explain the context of the previous study, where this current study was based, but only cited it. For my comments also see the pdf with my comments on this section

4.2.2 See the comments above on the abstract to enhance this one

4.2.3 Highlight some key findings of this previous study here to avoid making the readers look for the findings on their own. The point of scientific writing is to make the work easy to understand

<u>Author's response:</u> We appreciate the reviewer's comment and suggestion. We have revised our introduction section to include the reviewer's suggestion, providing an explanation of the previous study and emphasizing our key findings accordingly as follows (please refer to lines 101 to 106; page 5 in the manuscript):

> "The primary objective of this study, building on our previous research into risk factors related to overtaking, rear-end, and door crashes, is to conduct a more comprehensive investigation. Specifically, Pai identified buses and coaches as common crash partners in overtaking crashes; poor visibility, traversing manoeuvres, and teenage cyclists as risk factors for rear-end crashes; and built-up areas as a risk factor for door crashes."

4.3 Methodology

The method section was described well and is adequate, although we need to know whether normality checks were conducted.

<u>Author's response:</u> Thank you for your positive feedback on the method section and for your valuable suggestion regarding normality checks. We employed multivariate logistic regression models in our investigation, which do not require assuming the normality of the predictor variables. Logistic regression is resilient to deviations from normality as it estimates the likelihood of a binary outcome instead of assuming a normal distribution of the variables. Consequently, we refrained from performing formal normality assessments for the predictor variables.

4.4 Results

This area still requires more work. The way the results were presented was hasty, and we need to redo some of the highlighted sections. For example, a separate Univariate table is needed as Table 1.

<u>4.4,1 I think you need a joint univariate table of all factors studied that combining it all in the Bivariate table. It is a lazy way of reporting that require a reader to tease out proportions on their own. address this</u>

<u>Author's response:</u> We appreciate the reviewer's comment. In response to the suggestion for a joint univariate table of all studied factors, we acknowledge the importance of presenting comprehensive data that is readily interpretable. Our analysis

included an examination of crash type distributions across multiple independent variables. To explore these relationships, we employed Chi-square tests. Variables with significance levels below 0.2 were identified to minimize type II errors and were considered significantly associated with the outcome variables ($p < 0.05$). These variables were subsequently included in multiple logistic regression models.

We utilized stepwise logistic regression to estimate odds ratios while controlling for specific factors, following a methodological approach well-established in traffic injury studies (e.g., references a and b) and detailed in previous research (e.g., reference c). This approach allows for a nuanced understanding of how various factors interact to influence crash types, ensuring our findings are robust and informative. This methodological approach is well-established in the study of traffic injuries (e.g., references a and b) and has been detailed in previous studies (e.g., reference c).

a: Chen, P-L, Pai, C-W. Evaluation of injuries sustained by motorcyclists in approach-turn crashes in Taiwan. Accident Analysis and Prevention, 2019, 124, 33-39;

b: Chien, D-K., Hwang, HF, Lin, MR. Injury severity measures for predicting return-to-work after a traumatic brain injury. Accident Analysis and Prevention, 2017, 98, 101-107;

c: Maldonado G, Greenland S. Simulation study of confounder-selection strategies. Am J Epidemiol 1993, 138, 11, 923-936).

<u>4.4.2 Use one decimal place and not two</u>

<u>Author's response:</u> We appreciate this reviewer's comment. We have updated our tables (Tables 1-4) to display data with one decimal place instead of two (please refer to lines 213 to 276; pages 12-17 in the manuscript).

<u>4.4.3 After inserting a combined univariate table, please remove these percentages, as they are very misleading</u>

<u>Author's response:</u> We appreciate this reviewer's comment. However, presenting percentages is crucial for demonstrating the distribution among each crash type and others. Therefore, we have decided to continue using percentages as presentation in our manuscript.

<u>4.4.5 Tables: Here put frequencies/percenatgase and removed all the percentages from the table. the same applies to all other tables</u>

<u>Author's response:</u> We appreciate this reviewer's comment. Nevertheless, it is essential to use percentages to clearly demonstrate the distribution of each crash type across a set of variables. By reporting these percentages, we are able to identify whether one certain variable was over-involved in one crash type. Therefore, we have opted to maintain the use of percentages in our presentation.

<u>4.4.6 All most all the bivariate table has not been interpreted. but summarize using phrases like serveral variables as shown in table 2.</u>

<u>Author's response:</u> We appreciate this reviewer's comment. We have revised our results section to incorporate the reviewer's suggestion and rephrase the sentence accordingly (please refer to lines 215 to 229; page 13 in the manuscript):

> "Several variables in Table 2 reveal significant differences between rear-end crashes and non-rear-end crashes. Specifically, a higher proportion of rear-end crashes occurred under darkness-unlit conditions (50.2%) compared to darkness-lit conditions (37.5%). Additionally, rear-end crashes were more prevalent in rural areas with speed limits of ≥ 40 mph (43.0%) compared to urban areas with speed limits of 20–30 mph(33.1%). Crashes involving crash partners aged ≥ 65 accounted for 39.7% of rear-end crashes, which was higher compared to other age groups (age 41–64: 33.0% and ≤18: 36.0%). Furthermore, rear-end crashes were more likely to occur during midnight (47.6%) compared to rush hours (36.3%). Taxis were frequently involved in rear-end crashes (42.4%), as were male crash partners (36.8%). These findings highlight the significant influence of various factors on the likelihood of rear-end crashes. Variables such as darkness-unlit conditions, higher speed limits in rural areas, crash time, and characteristics of the crash partner all emerged as significant determinants. Specifically, rear-end crashes were notably more prevalent under darkness-unlit conditions, in rural areas with higher speed limits, during midnight hours, and involving certain characteristics of crash partners. Importantly, these associations were statistically significant, as indicated by the Chi-squared test ($p < 0.001$)."

<u>4,4,7 Do inteprete the results individually for all the significant factors.</u>

<u>Author's response:</u> We appreciate this reviewer's comment. We have revised our discussion section to incorporate the reviewer's suggestion and rephrase the sentence accordingly (please refer to lines 232 to 248; pages 14-15 in the manuscript):

> "As shown in Table 3, several variables can contribute to door crashes involving bicycles. Door crashes predominantly occurred in urban areas with speed limits of 20-30 mph (6.22%), while a significantly lower proportion occurred in rural

areas with speed limits ≥ 40 mph (0.45%). These crashes were overrepresented during non-rush hours (5.54%) and rush hours (4.94%) compared to evening (4.26%) and midnight (2.35%). Cyclists were more frequently involved in door crashes on weekdays (5.35%) than weekends (3.73%). As many as 8.21% of all female cyclists were involved in door crashes, which is higher than the involvement rate among males (4.24%).Taxi and private hire cars were overinvolved in door crashes (10.55%) compared to cars (5.17%) and buses/heavy goods vehicles (3.13%). Crash partners aged ≤18 years (5.22%) and 19-40 years (5.26%) were disproportionately involved in door crashes compared to older age groups, and female crash partners were overrepresented in door crashes (7.42%) compared to males (4.23%). These results were statistically significant, as indicated by the Chi-squared test (p < 0.001). They suggest that various factors including traffic conditions (rural areas, crash time), cyclist demographics (younger age, gender), and characteristics of the crash partner (taxi/private hire cars)—significantly contribute to the likelihood of door crashes involving cyclists."

4.4.8 where are the corresponding p-values. include them for all the significant risk factors

Author's response: We appreciate this reviewer's comment. We have revised our Results section to include the reviewer's suggestion and have added the corresponding p-values accordingly (please refer to lines 253 to 272; page 16 in the manuscript):

For example: "(AOR = 2.912, 95% CI = 2.384–3.556; p<0.001)."

4.4.9 Here, present both the crude and adjusted odd ratios

Author's response: We appreciate the reviewer's comment. In response, we have focused on presenting the adjusted odds ratios (AOR) and their corresponding 95% confidence intervals in our manuscript.

To address the analysis of crash types across various independent variables, we conducted Chi-square tests to assess the association between dependent and independent variables. The direction of the independent variables will be clarified in the subsequent multivariate logistic regression models.

Significant variables identified through stepwise selection were included in the multiple logistic regression models. The adjusted odds ratios (AOR) and their 95% confidence intervals were then calculated from these final models. This approach, widely used in traffic injury studies (e.g., a, b), ensures robust methodology by controlling for other variables (e.g., c).

a: Chen, P-L, Pai, C-W. Evaluation of injuries sustained by motorcyclists in approach-turn crashes in Taiwan. Accident Analysis and Prevention, 2019, 124, 33-39;

b: Chien, D-K., Hwang, HF, Lin, MR. Injury severity measures for predicting return-to-work after a traumatic brain injury. Accident Analysis and Prevention, 2017, 98, 101-107;

c: Maldonado G, Greenland S. Simulation study of confounder-selection strategies. Am J Epidemiol 1993, 138, 11, 923-936).

<u>4.4.10 Also do interpret these results. For instance what does the odd ratio of 2.93 mean in this case?</u>

<u>Author's response:</u> We appreciate the reviewer's comment and suggestion. We have revised our results section to incorporate the reviewer's suggestion and have interpreted the meaning of odds ratios in our findings accordingly (please refer to lines 279 to 286; pages17-18 in the manuscript):

> "The results identified several key risk factors for both overtaking and rear-end crashes. The risk of overtaking crashes showed a significant increase of 193% in rural areas when elderly drivers were involved (AOR = 2.93, 95% CI = 2.79–3.08), and similarly when heavy goods vehicles (HGVs) were the crash partner (AOR = 2.62, 95% CI = 2.46–2.78). Elderly cyclists also faced a higher risk of overtaking crashes on weekends (AOR = 1.56, 95% CI = 1.34–1.81).
>
> Regarding rear-end crashes, the risk increased notably with unlit darkness during midnight (AOR = 1.68, 95% CI = 1.48–1.90) and was significantly higher in rural areas (AOR = 2.15, 95% CI = 2.01–2.31)."

<u>4.4.11 you look at risk factors and not only environment factors, what about factors like sex, age. are they from the environment too, and yet you included them.</u>

<u>Author's response:</u> We appreciate the reviewer's comment. In our multivariate logistic regression results in Table 4, we analyzed and presented such factors such as cyclist's sex and age for each crash type (please refer to lines 275 to 276; page 17 in the manuscript). Moreover, in our joint-effect analysis, cyclist's age (≥65-year-old cyclist) was combined and analyzed with other variables.

4.5 Discussion

The section also needs serious work, especially on the way the findings were discussed. The authors should consider discussing their own findings rather than those of other studies. There is also a need to have a section for recommendations rather than merging it within result

4.5.1 You dont need this type of writing, just discuss the findings

<u>Author's response:</u> We appreciate the reviewer's comment. We have revised our discussion section to delete the paragraph as the reviewer's suggestion as follows (please refer to lines 305; page 19 in the manuscript):

> "Delete: These findings warrant further discussion and thus are elaborated on in this section of this paper."

You have not discussed the findings. Yes you found HGVs a risk for overtaking crash, so tell us why you think that is a risk factor. in other word explain your findings and then place it in the context of other study

<u>Author's response:</u> We appreciate the reviewer's comment and suggestion. We have revised our discussion section to integrate the reviewer's suggestion and provide a discussion on how heavy goods vehicles (HGVs) pose a risk for overtaking crashes accordingly as follows (please refer to lines 308 to 317; page 19 in the manuscript):

> "These findings align with previous research that identified elderly drivers, speeds exceeding 10 mph, and the presence of pick-up trucks as factors contributing to increased risk for overtaking crash. Specifically, HGVs possess several characteristics that amplify this danger. Their large blind spots make it difficult for drivers to see cyclists, increasing the likelihood of crashes during overtaking. Additionally, HGVs are less maneuverable compared to passenger cars, which reduces their ability to avoid crashes if cyclists suddenly enter their path. The speed and distance perception issues between HGVs and cyclists further complicate the judgment of safe overtaking gaps. Furthermore, HGVs require longer stopping distances due to their size and weight, which can lead to severe consequences if a sudden need to brake arises."

4.5.3 Do not discuss other people's findings, just discuss your findings and only state whether it agrees of disagrees with what Pai et al found for example

<u>Author's response:</u> We appreciate the reviewer's comment and suggestion. We have revised our Discussion section to incorporate the reviewer's suggestion and provide a

discussion on our findings, comparing them with previous studies accordingly (please refer to lines 318 to 322; pages 19-20 in the manuscript):

> "Regarding the association with buses or HGVs, our findings are consistent with existing research suggesting that time pressures on HGV drivers for timely loading and unloading might lead to more reckless driving. Specifically, our results align with the observations made by Pai et al., who also mentioned higher crash rates involving buses or HGVs, supporting the idea that these time pressures contribute to increased crash risks."

4.5.4 Take this to the recommendation section

Author's response: We appreciate the reviewer's comment and suggestion. We have added a recommendations section (please refer to lines 404-412; pages 23-24 of the manuscript):

> "Recommendations
>
> For overtaking crashes, we recommend implementing 'Share the Road' warning signs, especially in rural areas, and developing specialized cognitive training programs for elderly drivers. Regarding rear-end crashes, our suggestions include improving illumination during night time and implementing speed control measures on rural road segments. For door crashes involving parked cars, we propose enhancing driver sight triangles and increasing cyclist visibility. Moreover, implementing a two-stage door opening mechanism and an automatic detection device in vehicles to alert drivers of bicycles approaching from behind could potentially be beneficial."

4.5.5 Good use of references but first tells why you found what you found. And again your study was looking at comparing the risk factors for overtaking crashes with what was previously found in your study and the findings of that study needed to be described well in this study too

Author's response: We appreciate the reviewer's comment and suggestion. We have revised our Discussion section to incorporate the reviewer's suggestion and provide a discussion on our findings, comparing them with previous studies accordingly (please refer to lines 328 to 332; page 20 in the manuscript):

> "We found that as individuals age, their risk of being involved in road accidents increases, primarily due to declines in cognitive capabilities. Our study corroborates these findings by showing that older cyclists are more susceptible to accidents during overtaking maneuvers, which can be attributed to diminished reaction times and impaired decision-making abilities, their health, and their driving performance."

4.5.6 I think you need to also link this to delays in reacting as compared to the younger cyclist or driver.

Author's response: We appreciate this reviewer's comment. We have revised our Discussion section to integrate the reviewer's suggestion and provide a discussion on delays in reaction among elderly cyclists or drivers accordingly (please refer to lines 328 to 332; page 20 in the manuscript):

> "We found that as individuals age, their risk of being involved in road accidents increases, primarily due to declines in cognitive capabilities. Our study corroborates these findings by showing that older cyclists are more susceptible to accidents during overtaking maneuvers, which can be attributed to diminished reaction times and impaired decision-making abilities, their health, and their driving performance."

4.5.7 This is supposed to be a recommendation but first of all it is not right. it is not what you found but what you think is making more elder drivers to get into overspreading crashes. So recommend only based on what you found and not based on what you think.

Author's response: We appreciate the reviewer's comment and suggestion. We have revised our discussion section to integrate the reviewer's suggestion as follows (please refer to lines 339 to 346; pages 20-21 in the manuscript):

> "Based on our study's findings, we recommend the development of specialized interventions to improve road safety for elderly cyclists. Our analysis reveals that older cyclists are at a higher risk of being involved in overtaking crashes, with this increased risk being strongly linked to declines in cognitive capabilities associated with aging. To address this issue, we advocate for the implementation of targeted cognitive training programs specifically designed for elderly cyclists. These programs should focus on enhancing critical skills such as reaction time, situational awareness, and decision-making abilities, which are crucial for reducing crash risk and improving overall road safety. "

4.5.7 Now this is a good statement that should have followed your first sentence, starting from the full stop after segments. then you can now show us how similar it is with what Pai and others found.

Author's response: We appreciate this reviewer's comment. If we understand this reviewer correctly, this reviewer makes a valid argument that Advanced Stop Lines (ASLs), also called bike boxes that had been implemented in the UK for decades, would be beneficial in reducing conflicts between cars and cyclists. However, our study focuses on cyclist crashes that occurred on road segments only (i.e., 20 metres away from junctions); as a result, we remain reserved with discussing this finding with this engineering measure (i.e., ASLs).

In addition to this, we routinely discussed our current findings with those of Pai; for instance, HGVs, unlit streets and midnight hours, and taxis have been similarly identified as a risk factor for overtaking crashes, rear-end crashes, and door crashes, respectively.

4.6 discussions.

4.6.1 They need to tell us how they tried to minimize the biases that could have been introduced by the many study limitations identified for this study.

Author's response: We appreciate this reviewer's comment. One inherent problem with police-reported crash data is the variables not readily available, hereby causing unobserved heterogeneity across the observations. To overcome such a limitation, we estimated separate regression models, as suggested by Kim et al. (2006), for the three crash types; such an approach provides greater explanatory power compared to single overall models. Further, we conducted joint-effect analyses of several variables of interest that capture heterogeneity. In our previous studies, we adopted the above-mentioned approaches to overcome the inherent problem with a success (see, for example, Pai and Saleh, 2008; Pai and Jou, 2014).

To clarify this, the following statements have been added to the Discussion section of the manuscript (please refer to lines 391 to 397; page 23 in the manuscript):

> "One inherent problem with police-reported crash data is the variables not readily available, hereby causing unobserved heterogeneity across the observations. To overcome such a limitation, we estimated separate regression models, as suggested by Kim et al. (e.g., d), for the three crash types; such an approach provides greater explanatory power compared to single overall models. Further, we conducted joint-effect analyses of several variables of interest that capture heterogeneity. In our previous studies, we adopted the above-mentioned approaches to overcome the inherent problem with a success (e.g., e, f)."

d: Kim, D., Washington, S., Oh, J., 2006. Modelling crash outcomes: new insights into the effects of covariates on crashes at rural intersections. Journal of Transportation Engineering. 132 (4), 282-292.

e: Pai CW, Jou RC, 2014. Cyclists' red-light running behaviours: An examination of risk-taking, opportunistic, and law-obeying behaviours. Accident Analysis and Prevention. 62,191-198.

f: Pai CW, Saleh W., 2008. Modelling motorcyclist injury severity by various crash types at T-junctions in the UK. Safety Science. 13, 98-98.

4.6.2 Present both the strength and limitations of the study. And you have really brought the limitation well, but my question would be, despite knowing all these why did you decided to carry on to utilised this dataset as opposed to others. Please tell us how you catered for these limitations as away of reducing bias that might have been introduced by them

Author's response: We appreciate this reviewer's comment. We have added a section discussing the strengths of our study before addressing its limitations as follows (please refer to lines 370 to 377; page 22 in the manuscript):

> "The strengths of this study include the use of STATS19 datasets spanning from 1991 to 2020, which provides a robust statistical foundation and a broad perspective on trends in bicycle crashes. By focusing specifically on three crash types on road segments—overtaking, rear-end, and door crashes—the study provides a comprehensive and focused analysis, which can yield more actionable insights and more effective recommendations. The UK-based dataset ensures that the findings are particularly relevant for local policy and safety interventions. Additionally, the application of statistical techniques and the consideration of various factors, such as crash partner and time of day, enhance the validity and depth of the analysis."
