# Supplementary material for: Risk factors for overtaking, rear-end, and door crashes involving bicycles in the United Kingdom: Revisited and reanalysed
Source: PLoS One. 2025 Jan 3;20(1):e0315692. doi: 10.1371/journal.pone.0315692 (PMC11698528; doi:10.1371/journal.pone.0315692)
Supplement: Attachment — Submitted filename: PONE-D-24-17126_R1_Reviewed.pdf [file pone.0315692.s004.pdf]

PLOS ONE

Risk Factors for Overtaking, Rear-End, and Door Crashes Involving Bicycles in the United Kingdom: Revisited and Reanalysed

--Manuscript Draft--

**Manuscript Number:**	PONE-D-24-17126R1
**Article Type:**	Research Article
**Full Title:**	Risk Factors for Overtaking, Rear-End, and Door Crashes Involving Bicycles in the United Kingdom: Revisited and Reanalysed
**Short Title:**	Risk Factors for Overtaking, Rear-End, and Door Crashes Involving Bicycles in the United Kingdom
**Corresponding Author:**	Chih-Wei Pai<br>Taipei Medical University<br>Taipei, TAIWAN
**Keywords:**	Bicycle crash; Road segment; Overtaking crash; Rear-end crash; Door crash
**Abstract:**	Objectives<br><br>Relevant research has provided valuable insights into risk factors for bicycle crashes at intersections. However, few studies have focused explicitly on three common types of bicycle crashes on road segments: overtaking, rear-end, and door crashes. This study aims to identify risk factors for overtaking, rear-end, and door crashes that occur on road segment.<br><br>Material and methods<br><br>We analysed British STATS19 accident records from 1991 to 2020. Using multivariate logistic regression models, we estimated adjusted odds ratios (AORs) with 95% confidence intervals (CIs) for multiple risk factors. The analysis included 127,637 bicycle crashes, categorised into 18,350 overtaking, 44,962 rear-end, 6,363 door, and 57,962 other crashes.overtaking, 44,962 rear-end, 6,363 door, and 57,962 other crashes.<br><br>Results<br><br>Significant risk factors for overtaking crashes included speed limits of ≥40 miles per hour (mph) (AOR = 2.238, 95% CI = 2.159–2.320), heavy goods vehicles (HGVs) as crash partners (AOR = 2.867, 95% CI 2.473–3.323), and elderly crash partners (AOR = 2.013, 95% CI = 1.937–2.092). For rear-end crashes, noteworthy risk factors included unlit darkness (AOR = 1.486, 95% CI = 1.404–1.573) and midnight hours (AOR = 1.269, 95% CI = 1.190–1.354). Factors associated with door crashes included speed limits of 20–30 mph (AOR = 16.185, 95% CI = 13.514–19.382) and taxi and private hire cars (AOR = 2.695, 95% CI = 2.310–3.145). Our joint-effect analysis revealed additional interesting results; for example, there were elevated risks for overtaking crashes in rural areas with elderly drivers as crash partners (AOR = 2.93, 95% CI = 2.79–3.08) and with HGVs as crash partners (AOR = 2.62, 95% CI = 2.46–2.78).<br><br>Conclusions<br><br>The aforementioned risk factors remained largely unchanged since 2011, when we conducted our previous study. However, the present study concluded that the detrimental effects of certain variables became more pronounced in certain situations. For example, cyclists in rural settings exhibited an elevated risk of overtaking crashes involving HGVs as crash partners.
**Order of Authors:**	Chun-Chieh Chao
	Hon-Ping Ma
	Li Wei

Powered by Editorial Manager® and ProduXion Manager® from Aries Systems Corporation

	Yen-Nung Lin
	Chenyi Chen
	Wafaa Saleh
	Bayu Satria Wiratama
	Akhmad Fajri Widodo
	Shou-Chien Hsu
	Shih Yu Ko
	Hui-An Lin
	Cheng-Wei Chan
	Chih-Wei Pai
**Response to Reviewers:**	Reviewer comments:<br>Reviewer 1: Regarding the statistical analysis, I would like to ask the authors to explain:<br>1. the reason(s) for ignoring any probable interaction between independent variables in the multivariate logistic regression.<br>Author's response: We appreciate the reviewer's comment and question. By examining variables independently, we gain a clearer understanding of their individual impacts on the outcome (specifically, crash type in this study). This approach allows us to assess each variable's direct influence without the added complexity of interactions or modifications between variables. It provides insights into which variables independently affect the outcome, directly addressing our research questions. Initially, we used the chi-squared test to explore associations between a set of independent variables and the three crash types. To minimize type II errors in variable selection and ensure unbiased inferences, we included variables with a p-value less than 0.2 from the univariate analysis into the multivariate logistic regression models, a common practice in past studies of traffic injuries (e.g., a, b) and methodology (c). Subsequently, we examined interaction effects among several variables of interest, as depicted in Figure 2 of the manuscript. While acknowledging the potential for other interactions among variables, our study focused on assessing the joint effects of specific variables of interest. To take overtaking crashes as an example, these variables included rural areas, crash partners aged 65 years or older, heavy goods vehicles, weekends, and cyclists aged 65 years or older. Future research could delve into untangling the complexities of additional interaction effects among variables, as suggested by the reviewer.<br>References:<br>a: Chen, P-L, Pai, C-W. Evaluation of injuries sustained by motorcyclists in approach-turn crashes in Taiwan. Accident Analysis and Prevention, 2019, 124, 33-39.<br>b: Chien, D-K., Hwang, HF, Lin, MR. Injury severity measures for predicting return-to-work after a traumatic brain injury. Accident Analysis and Prevention, 2017, 98, 101-107.<br>c: Maldonado G, Greenland S. Simulation study of confounder-selection strategies. Am J Epidemiol 1993, 138, 11, 923-936.<br><br>2. Why did they consider different reference categories for the same individual variables among different outcomes in logistic regression modeling? This will make it difficult to interpret the comparison of the effect of an independent variable on different types of crashes. for example, in table 4, the ref category for Light condition is Darkness-lit, Daylight and Darkness-unlit for Overtaking, Rear-end and Door crashes respectively.<br>Author's response: We appreciate the reviewer's comment and question. In our analysis, we chose various reference categories for variables based on the lowest Adjusted Odds Ratios (AORs) observed. This approach allowed us to highlight different risk factors associated with higher AORs for specific types of crashes. For example, urban roads with speed limits of 20-30 mph were identified as protective factors for overtaking and rear-end crashes. However, for door crashes, these urban roads appeared to pose a higher risk compared to rural roads, as indicated by their higher AOR. It is important to note that selecting a reference category does not change the estimation results of our models. Instead, assigning reference case with the lowest

Powered by Editorial Manager® and ProduXion Manager® from Aries Systems Corporation

AOR helps readers identify risk factors with higher AORs among the three crash types.

3. I suggest authors provide identical indicators for figures both in the main text and in the figure's caption. Reading "Fig. 1" below a figure, one will look for the same word in the main text while it is recalled as "Figure 1".
Author's response: We appreciate this reviewer's comments, and we have revised the manuscript in the main text and figure's caption (please refer to lines 145 to 146; page 8 in the manuscript).
Reviewer 2:
1 General comments:
1.1 None of the authors was from the UK???
Author's response: We appreciate this reviewer's comments. One of our authors, Prof. Wafaa Saleh, is from Edinburgh Napier University, UK.

1.2 The authors should emphasize the significance of including these three types of crashes????
Author's response: We appreciate the reviewer's comments. We have incorporated the following statements into the introduction to underscore the significance of including the three crash types (please refer to lines 110 to 115; pages 5-6 in the manuscript):
"The study addresses a critical gap in current research, focusing on crashes specifically occurring on road segments. Existing literature offers limited insights into this specific type of crash, highlighting a crucial need for targeted investigation. These crashes have the potential for severe impact, involving complex dynamics that demand a nuanced understanding for effective mitigation strategies. By exploring these factors, our research aims to significantly enhance cyclist safety within this particular context."

1.3 What novelty this study adds compared to the previous one in 2011???
Author's response:
We appreciate this reviewer's comment. One inherent problem with police-reported crash data is the variables not readily available, hereby causing unobserved heterogeneity across the observations. To overcome such a limitation, we estimated separate regression models, as suggested by Kim et al. (e.g., d), for the three crash types; such an approach provides greater explanatory power compared to single overall models. Further, we conducted joint-effect analyses of several variables of interest that capture heterogeneity. In our previous studies, we adopted the above-mentioned approaches to overcome the inherent problem with a success (e.g., e, f). To clarify this, the following statements have been added to the Discussion section of the manuscript (please refer to lines 391 to 397; page 23 in the manuscript):
"One inherent problem with police-reported crash data is the variables not readily available, hereby causing unobserved heterogeneity across the observations. To overcome such a limitation, we estimated separate regression models, as suggested by Kim et al. (e.g., d), for the three crash types; such an approach provides greater explanatory power compared to single overall models. Further, we conducted joint-effect analyses of several variables of interest that capture heterogeneity. In our previous studies, we adopted the above-mentioned approaches to overcome the inherent problem with a success (e.g., e, f)."
d: Kim, D., Washington, S., Oh, J., 2006. Modelling crash outcomes: new insights into the effects of covariates on crashes at rural intersections. Journal of Transportation Engineering. 132 (4), 282-292.
e: Pai CW, Jou RC, 2014. Cyclists' red-light running behaviours: An examination of risk-taking, opportunistic, and law-obeying behaviours. Accident Analysis and Prevention. 62,191-198.
f: Pai CW, Saleh W., 2008. Modelling motorcyclist injury severity by various crash types at T-junctions in the UK. Safety Science. 13, 98-98.

1.4 The rationale for conducting the current study as well as the practical implications should be emphasized??
Author's response: We appreciate this reviewer's comments. First, regarding the rationale for conducting the current study, we have added the following statements (please kindly refer to lines 91-95 on page 5 of the manuscript):
"Bicycle crashes on road segments remain a substantial issue for public health concern. Existing research primarily emphasizes intersection-related crashes. This study aims to fill a critical gap by conducting a thorough examination of the risk factors associated with three distinct bicycle crash types: overtaking, rear-end, and door

crashes that occur on road segments."
Secondly, to highlight the practical implications, we have included the following statements in the Discussion section (please refer to lines 404-412 on pages 23-24 of the manuscript):
"Recommendations
For overtaking crashes, we recommend implementing 'Share the Road' warning signs, especially in rural areas, and developing specialized cognitive training programs for elderly drivers. Regarding rear-end crashes, our suggestions include improving illumination during night time and implementing speed control measures on rural road segments. For door crashes involving parked cars, we propose enhancing driver sight triangles and increasing cyclist visibility. Moreover, implementing a two-stage door opening mechanism and an automatic detection device in vehicles to alert drivers of bicycles approaching from behind could potentially be beneficial."

1.5 For the introduction section, burden in terms of mortality, morbidity, and DALYs should be mentioned as well the economic and health care costs should be mentioned (globally and UK)
Author's response: We appreciate the reviewer's comments. Our original literature review has included several past studies that have reported the accident/injury outcomes resulting from these three crash types. For example, road segments with elevated speed limits, male cyclists, and cyclists aged over 55 years contribute significantly to high injury severity crashes. Additionally, built-up areas increase the risk of door crashes involving cyclists and parked cars.
It is important to note that there is limited research specifically examining the impact of overtaking, rear-end, and door crashes on Disability-Adjusted Life Years DALYs, economic costs, and healthcare expenses. Notable exceptions include studies by Elvik and Sundfør (e.g., d), who examined the inclusion of cyclist injuries in health impact economic assessments. Aertsens et al. (e.g., h) and Scholten et al. (e.g., i) also provided comprehensive analyses of the total and average costs associated with bicycle injuries. Although the three crash types were not explicitly examined in the above-mentioned studies, we have followed this reviewer's suggestion by incorporating these studies into the 'Introduction' section (please refer to lines 77-81; page 4 of the manuscript):
"Bicycle crashes can also impose a significant burden on healthcare expenses. Elvik and Sundfør (e.g., g) have discussed the economic implications and healthcare expenditures associated with bicycle accidents. For instance, in Belgium, the average cost of bicycle accidents per case is estimated at 841 euros (e.g., h). In the Netherlands, the total annual cost has been reported as €410.7 million (e.g., i)."
References:
g: Elvik, R., & Sundfør, H. B. (2017). How can cyclist injuries be included in health impact economic assessments? Journal of Transport & Health, 6, 29-39.
h: Aertsens, J., de Geus, B., Vandenbulcke, G., Degraeuwe, B., Broekx, S., De Nocker, L., ... & Panis, L. I. (2010). Commuting by bike in Belgium, the costs of minor accidents. Accident Analysis & Prevention, 42(6), 2149-2157.
i: Scholten, A. C., Polinder, S., Panneman, M. J., Van Beeck, E. F., & Haagsma, J. A. (2015). Incidence and costs of bicycle-related traumatic brain injuries in the Netherlands. Accident Analysis & Prevention, 81, 51-60.
1.6 The number of cyclists in UK or those using bicycles for their mobility??
Author's response: We appreciate the reviewer's comment. In our study, we analyzed national police-reported crash data involving cyclists. Unfortunately, exposure data, such as the number of cyclists and miles traveled, were not available in the STATS19 dataset. While such data may be available from the UK National Travel Survey, it often reflects outdated information and may not be fully representative of the entire population.
2. Specific comments:
2.1 Instead of data collection, data used for analysis is appropriate??
Author's response: We appreciate the reviewer's comment. The dataset, UK Stats19 covering all traffic accidents in the UK, should be appropriate, as numerous studies in the field of traffic injury and medicine have analysed such data (e.g., references j, k, l).
j: Haghpanahan, Houra, et al. "An evaluation of the effects of lowering blood alcohol concentration limits for drivers on the rates of road traffic accidents and alcohol consumption: a natural experiment." The Lancet 393.10169 (2019): 321-329.
k: Pai, C. W., Hwang, K. P., & Saleh, W. (2009). A mixed logit analysis of motorists' right-of-way violation in motorcycle accidents at priority T-junctions. Accident Analysis

Powered by Editorial Manager® and ProduXion Manager® from Aries Systems Corporation

& Prevention, 41(3), 565-573.
l: Fountas, G., Fonzone, A., Gharavi, N., & Rye, T. (2020). The joint effect of weather and lighting conditions on injury severities of single-vehicle accidents. Analytic methods in accident research, 27, 100124.

2.2 Of the used crashes data, how many were fatal???
Author's response: We appreciate the reviewer's comment. As reported in the table below, as many as 0.8% of those in overtaking crashes sustained fatal injuries, which was the highest compared to those in the other two crash types.
SlightSeriousFatalTotal
Overtaking crashes14240(77.6%)3,964(21.6%)147(0.8%)18350
Rear-end crashes39821(89.1%)4782(10.7%)89(0.2%)44692
Door crashes5561(87.4%)770(12.1%)32(0.5%)6363
2.3 For analysis of data, use the Odds ratios and 95% confidence intervals (univariate and bivariate)
Author's response: We appreciate this reviewer's comment. We analyzed the distribution of crash types across a set of independent variables. Chi-square tests were used to explore relationships between these variables and crash types. Variables with a significance level below 0.2 were identified to minimize type II errors and were considered significantly associated with the outcome variables ($p<0.05$).
Subsequently, these variables were included in multiple logistic regression models. Stepwise logistic regression was then employed to estimate the odds of various variables after controlling for specific factors. This methodology has been widely used in past studies of traffic injuries (e.g., a, b) and methodology (e.g., c).
a: Chen, P-L, Pai, C-W. Evaluation of injuries sustained by motorcyclists in approach-turn crashes in Taiwan. Accident Analysis and Prevention, 2019, 124, 33-39;
b: Chien, D-K., Hwang, HF, Lin, MR. Injury severity measures for predicting return-to-work after a traumatic brain injury. Accident Analysis and Prevention, 2017, 98, 101-107;
c: Maldonado G, Greenland S. Simulation study of confounder-selection strategies. Am J Epidemiol 1993, 138, 11, 923-936).

2.4 Details about the multivariate logistic regression model should be mentioned???
Use the Odds ratios for interpreting and displaying the results in tables 1, 2, and 3???
Author's response: We appreciate the reviewer's comment. Firstly, if we understand this reviewer correctly, we have incorporated additional details (such as formulation and derivation) of the multivariate logistic regression model into the "Methods" section (please refer to lines 179-194 on pages 10-11 of the manuscript):
"Initially, we examined the distribution of three crash types across various variables to explore their relationships with a binary outcome. These variables included lighting conditions, speed limit, time of day, and day of the week. Demographic details concerning cyclist casualties encompassed age and sex, while information about the crash partner included vehicle type, age, and sex. We set a significance level of $p < 0.2$ to include risk factors in our multivariate analysis. Adjusted odds ratios (AORs) were computed using multivariate logistic regression with backward selection.
The multivariate logistic regression model equation was specified as:
log(P(Y=1)/(1 - P(Y=1))) =β_0+β_1 X_1+β_2 X_2
where P(Y=1) denotes the probability of the outcome, $\beta 0,\beta 1,\beta 2,\dots,\beta p$ are the coefficients to be estimated, and $X1,X2,\dots,Xp$ represent the predictor variables. Before estimating the model, assumptions of logistic regression, such as linearity of the logit, absence of multicollinearity, and independence of observations, were evaluated. An odds ratio (OR) greater than 1 indicated a positive association between the independent variable and the occurrence rate, while an OR less than 1 indicated a negative association. An OR of 1 suggested no association between the variables of interest and the outcomes."
Secondly, this reviewer suggested that we should use the Odds ratios for interpreting and displaying the results in tables 1, 2, and 3. While we acknowledge this suggestion, we would like to clarify here that we adopted the commonly-used Chi-square tests to identify the distribution of three crash types across several independent variables. Instead of the univariate logistic regression, such a method has been proved as an efficient way to minimize type II errors, and has been widely employed in past studies of traffic injuries (e.g., a, b) and methodology (e.g., c).
a: Chen, P-L, Pai, C-W. Evaluation of injuries sustained by motorcyclists in approach-turn crashes in Taiwan. Accident Analysis and Prevention, 2019, 124, 33-39;

b: Chien, D-K., Hwang, HF, Lin, MR. Injury severity measures for predicting return-to-work after a traumatic brain injury. Accident Analysis and Prevention, 2017, 98, 101-107;
c: Maldonado G, Greenland S. Simulation study of confounder-selection strategies. Am J Epidemiol 1993, 138, 11, 923-936).

2.5 Chi square is not enough test to identify the direction and which segment of the given variable is significantly different???
Author's response: We appreciate this reviewer's comment. The reviewer is correct. Chi-square tests can be used for ascertaining the association of the dependent and independent variables. However, the direction of the independent variables can be untangled in the subsequent multivariate logistic regression models.

2.6 What was the adjustment made for??? And how???
Author's response: We appreciate this reviewer's comment. Each variable was adjusted for in the multivariate analysis. For instance, in Table 4, adjustments were made for crash day after accounting for other variables such as cyclist's sex, crash partner, and crash partner's age and sex.

2.7 The joint-crash effect: how it was measured statistically???
Author's response: Thank you for your valuable comment. We do apologize for not making our analysis clear. To clarify how joint-effect analysis was structured, we drew several figures below that help us respond to this reviewer.
As Figure A1 (X axis: speed limit; Y axis: percentage) and A2 report (X axis: Crash partner's age; Y axis: percentage), the joint effects of speed limit (two categories: rural (≥ 40 mph) /urban (20–30 mph)] and crash partner's age (four categories: ≤18, 19–40, 41–64, and ≥65) on overtaking crashes were examined, yielding eight combinations of interaction effects (i.e., 1. Rural x ≤18; 2. Rural x 19-40; 3. Rural x 41-64; 4. Rural x ≥65; 5. Urban x ≤18; 6. Urban x 19-40; 7. Urban x 41-64; 8. Urban x ≥65). All percentages of overtaking crashes among these eight combinations were compared, and the combination with the highest percentages for overtaking crashes is taken as the indicator variable. In this joint-effect analysis, the indicator variable "rural areas x crash partner's ≥65 years old" has the highest percentage of overtaking crashes. These results elucidated that overtaking crashes were more likely to occur when the cyclists were in rural areas and when involving ≥65-year-old crash partners.

Figure A1 Figure A2
In practice, such a joint-effect analysis has been widely employed in medicine or traffic injury literature. One well-known paper by Weinstein et al. (i.e., m) was published in JAMA which examined the joint effect of physical activity and body mass index on diabetes in women. In this paper, Weinstein et al. pointed out that the beneficial effect of active lifestyle on type 2 diabetes was consistent across women with three BMI levels.

Another example is our previous paper published in Accident Analysis and Prevention in 2020 titled: Evaluating the combined effect of alcohol-involved and un-helmeted riding on motorcyclist fatalities in Taiwan. In this study, we specifically analysed the joint effect of alcohol use and helmet use on motorcyclist fatalities (i.e., n).

In addition, our previous paper published in BMC Public Health in 2023 titled: Walking against traffic and pedestrian injuries in the United Kingdom: new insights (i.e., o). In this study, we specifically analysed the joint effect to examine whether the beneficial effect of walking against traffic on injury severity may apply to different situations. By doing so, we were able to compare injury outcomes in walking against-traffic crashes against those in walking with-traffic crashes.

We believe this detailed explanation clarifies our methodology.

Powered by Editorial Manager® and ProduXion Manager® from Aries Systems Corporation

Reference:
m: Weinstein A., Sesso, H., Lee, I., Cook, N., Manson, J., Buring, J., Gaziano, J., 2004. The relationship of physical activity vs body mass index with type 2 diabetes in women. JAMA 290: 1188-1194.
n: Wiratama, B., Chen, P., Ma, S., Chen, Y., Saleh, W., Lin, H., Pai, C., 2020. Evaluating the combined effect of alcohol-involved and un-helmeted riding on motorcyclist fatalities in Taiwan. Accident Analysis and Prevention, 143, 105594.
o: Widodo, A. F., Chen, C., Chan, C. W., Saleh, W., Wiratama, B. S., & Pai, C. W. (2023). Walking against traffic and pedestrian injuries in the United Kingdom: new insights. BMC public health, 23(1), 2205.

Reviewer #3: Areas for Improvement:
3.1 Clarity and Conciseness:

Some sections of the text are verbose and could benefit from more concise language. For instance, the detailed descriptions of statistical methods and results could be streamlined without losing essential information.
Simplifying the language and structure would enhance readability and accessibility, particularly for readers who are not specialists in the field.
Author’s response: We appreciate the reviewer’s valuable suggestions. Concerning two reviewers who recommended extending several sections (i.e., reviewer #2 asked us to explain more on multivariate regression models and reviewer #4 requested for further discussions), we maintained a neutral stance for the time being. Nonetheless, we have revised the introduction to provide a clearer context and expanded our descriptions in the discussion section to provide broader insights into the implications of our findings. Additionally, detailed descriptions of the statistical methods have been included in the methods section, aimed at enhancing readability and accessibility for our readers.

3.2 Detailed Interpretation of Results:

While the results section provides extensive data, there is limited interpretation of what these results mean in practical terms. Adding more context about how these findings could influence policy or infrastructure design would be valuable. Discussing potential interventions based on the identified risk factors, such as specific infrastructure improvements or policy changes, would strengthen the practical implications of the study.
Author’s response: We appreciate the reviewer’s comment and suggestion. We have revised the discussion section of the manuscript and added one recommendation section to address findings that could potentially influence policy or infrastructure as follows (please refer to lines 404-412 on pages 23-24 of the manuscript):
“Recommendations
For overtaking crashes, we recommend implementing 'Share the Road' warning signs, especially in rural areas, and developing specialized cognitive training programs for elderly drivers. Regarding rear-end crashes, our suggestions include improving illumination during night time and implementing speed control measures on rural road segments. For door crashes involving parked cars, we propose enhancing driver sight triangles and increasing cyclist visibility. Moreover, implementing a two-stage door opening mechanism and an automatic detection device in vehicles to alert drivers of bicycles approaching from behind could potentially be beneficial.”

3.3 Comparative Analysis:
Including a comparative analysis with similar studies from other countries could provide a broader context for the findings and highlight whether these risk factors are unique to the UK or consistent globally.
Discussing how the UK’s findings compare with those from the United States or other European countries, especially concerning the impact of infrastructure and vehicle types, could offer valuable insights:
Author’s response: We appreciate the reviewer’s comment. To our knowledge, no comparative analysis from other countries has been conducted for the three crash types (overtaking, rear-end, and door crashes). In addition, it is out of the scope of the current research to obtain crash data from other countries and conduct a large scale of comparative analysis. However, in our introduction sections, we have reviewed previous studies that focused on risk factors for these crash types individually or

Powered by Editorial Manager® and ProduXion Manager® from Aries Systems Corporation

collectively on road segments (such as vehicle volume, traffic density, and number of lanes).
In the discussion section, we have discussed our findings with those of other studies in the US or elsewhere. For instance, previous analyses of overtaking crashes highlighted risk factors such as speeds exceeding 10 mph and the presence of pick-up trucks. Rear-end crashes were associated with conditions such as darkness, unlit surroundings, midnight hours, and reduced cognitive capabilities. Door crashes were found to be influenced by factors including urban roadways and the presence of taxis.

3.4 Providing more detailed information about the methodology, particularly the criteria for excluding certain data points, would enhance transparency. For example, explaining why specific demographic data were incomplete and how this might affect the results would be useful.
A discussion on the limitations of the data and the potential biases introduced by police reporting practices could provide a more nuanced understanding of the findings.
Author's response: We appreciate the reviewer's comment. To clarify the reasons for excluding junction cases and cyclists/motorcyclists as crash partners, we have added the following statements in the Methods section (please also kindly see lines 168 to 174; pages 9-10 in the manuscript):
"On a cautionary note, we removed junction cases to avoid the variability introduced when exogenous factors, such as junction geometry and control measures, are present at junctions. Furthermore, the cases involving other cyclists and motorcyclists were removed as we focused on vehicle-cycle crashes only. Missing data on sex, age, or speed limits were also excluded in the analysis. Excluding these data may impact our results in a marginal scale, as these data are likely to be single-bicycle crashes that in nature be underreported in police crash dataset [e.g., p]. "
Regarding the limitation of police reported crash data, the following statements have been added to the manuscript (please also kindly see lines 378 to 391; pages 22-23 in the manuscript):
"This study had several limitations that warrant acknowledgement. First, the substantial underreporting of nonfatal casualties to the police, particularly casualties involving cyclists not obligated to report accidents, is a critical factor to consider. Such underreporting, as highlighted by the U.K. Government's Department for Transport, likely results in the incomplete representation of nonfatal and 'slight' casualties in road casualty data. Second, the STATS19 data utilised in this study lack critical variables, including precrash speeds, specific geometric characteristics of roadways, data regarding alcohol and illicit substance use, and cyclist speed at the time of an accident. Moreover, critical exposure data—such as those related to traffic flow, rider or driver experience, and other elements of risk exposure—are absent, and the absence of such details limits our ability to fully account for potential variations resulting from unobserved factors in the analyses. Finally, this study did not explore annual trends in each type of bicycle crash over the 30-year study period; investigating such trends could provide insights regarding changing behaviours among cyclists and motor vehicle drivers as well as the effects of legislative changes for road speed limits."
p. Watson, Angela, Barry Watson, and Kirsten Vallmuur. "Estimating under-reporting of road crash injuries to police using multiple linked data collections." Accident Analysis & Prevention 83 (2015): 18-25.

3.4 Visual Aids:

Adding more visual aids, such as graphs or charts, could help in visualizing the key findings and making the data more accessible to readers.
A geographic distribution map showing where different types of crashes are more prevalent could add an interesting dimension to the analysis.
Author's response: We appreciate the reviewer's suggestions. We firstly reported our sampling by using a flowchart that helps readers understand what data were excluded and included in the analyses. Although we presented our statistical analyses in a traditional way (Tables 1 to 4), we illustrated a forest plot demonstrating the joint effects of several variables on the three crash types when other variables were controlled for (please refer to lines 213 to 276; pages 12-17 in the manuscript).
Regarding the geographic distribution map illustrating where these crash types were more prevalent, our research objective does not primarily emphasize the geographic effects of these three crash types. Rather, we focused on identifying risk factors for these crash types. While we appreciate this reviewer's valuable comment on this, we

Powered by Editorial Manager® and ProduXion Manager® from Aries Systems Corporation

	have identified this as an important research area as follows (please refer to lines 398 to 402; page 23 in the manuscript):<br>“Future research directions could involve integrating GPS (Global Positioning System) data and weather conditions to analyse both the injury frequency and fatalities of bicycle crashes on road segments. Additionally, exploring the potential of autonomous vehicles for detecting approaching bicycles for door-crashes and implementing AI-controlled lighting systems in rural areas for cyclist detection could be promising areas for further study.”<br><br>3.5 Future Directions:<br>Including a section on future research directions would be beneficial. Identifying gaps in the current research and suggesting areas for further investigation could guide subsequent studies.<br>Discussing the potential impact of emerging technologies, such as autonomous vehicles and advanced cyclist detection systems, on these crash types could provide a forward-looking perspective.<br>Author’s response: We appreciate the reviewer’s comment and suggestion. We have revised the discussion section of the manuscript and added one future research section (please refer to lines 398 to 402; page 23 in the manuscript). Furthermore, we have added one new section “Recommendation” that reports potential intervention points (please refer to lines 404-412 on pages 23-24 of the manuscript):<br>“Recommendations<br>For overtaking crashes, we recommend implementing 'Share the Road' warning signs, especially in rural areas, and developing specialized cognitive training programs for elderly drivers. Regarding rear-end crashes, our suggestions include improving illumination during night time and implementing speed control measures on rural road segments. For door crashes involving parked cars, we propose enhancing driver sight triangles and increasing cyclist visibility. Moreover, implementing a two-stage door opening mechanism and an automatic detection device in vehicles to alert drivers of bicycles approaching from behind could potentially be beneficial.”<br>Future research directions:<br>“Future research directions could involve integrating GPS (Global Positioning System) data and weather conditions to analyse both the injury frequency and fatalities of bicycle crashes on road segments. Additionally, exploring the potential of autonomous vehicles for detecting approaching bicycles for door-crashes and implementing AI-controlled lighting systems in rural areas for cyclist detection could be promising areas for further study.”<br><br>Reviewer #4: This Study is technically sound and has potential to add to the body of knowledge involving bicycle riding safety in the UK and everywhere across the globe. It has adhered to the research and publication ethics, however, the study still need revision on some of the key identified areas which i have pointed out, starting from abstract, background, results and discussions.<br>4.1 Abstract<br>The abstract is lacking the background section, please see the comment on the pdf<br>This abstract is lacking the background section, which must start when presenting structured abstract. Also there is no objective put here, but rather the research problem investigated.<br>Author’s response: We appreciate the reviewer’s comment and suggestion. We have revised the abstract to add background and objects as follows (please refer to lines 23 to 27; page 2 in the manuscript):<br>“Background: Relevant research has provided valuable insights into risk factors for bicycle crashes at intersections. However, few studies have focused explicitly on three common types of bicycle crashes on road segments: overtaking, rear-end, and door crashes.<br>Objective: This study aims to identify risk factors for overtaking, rear-end, and door crashes that occur on road segment."<br><br>4.1.1 Abbreviations should be defined when they are first mentioned<br>Author’s response: We appreciate the reviewer’s suggestion. We have revised the abstract to include the full definitions of abbreviations upon their first appearance as follows (please refer to lines 31; page 2 in the manuscript).<br>“Abstract: AOR (adjusted odds ratio)”

Powered by Editorial Manager® and ProduXion Manager® from Aries Systems Corporation

	4.2 Introduction<br>4.2.1 The authors did not explain the context of the previous study, where this current study was based, but only cited it. For my comments also see the pdf with my comments on this section<br>4.2.2 See the comments above on the abstract to enhance this one<br>4.2.3 Highlight some key findings of this previous study here to avoid making the readers look for the findings on their own. The point of scientific writing is to make the work easy to understand<br>Author's response: We appreciate the reviewer's comment and suggestion. We have revised our introduction section to include the reviewer's suggestion, providing an explanation of the previous study and emphasizing our key findings accordingly as follows (please refer to lines 101 to 106; page 5 in the manuscript):<br>"The primary objective of this study, building on our previous research into risk factors related to overtaking, rear-end, and door crashes, is to conduct a more comprehensive investigation. Specifically, Pai identified buses and coaches as common crash partners in overtaking crashes; poor visibility, traversing manoeuvres, and teenage cyclists as risk factors for rear-end crashes; and built-up areas as a risk factor for door crashes."<br><br>4.3 Methodology<br>The method section was described well and is adequate, although we need to know whether normality checks were conducted.<br>Author's response: Thank you for your positive feedback on the method section and for your valuable suggestion regarding normality checks. We employed multivariate logistic regression models in our investigation, which do not require assuming the normality of the predictor variables. Logistic regression is resilient to deviations from normality as it estimates the likelihood of a binary outcome instead of assuming a normal distribution of the variables. Consequently, we refrained from performing formal normality assessments for the predictor variables.<br>4.4 Results<br>This area still requires more work. The way the results were presented was hasty, and we need to redo some of the highlighted sections. For example, a separate Univariate table is needed as Table 1.<br>4.4,1 I think you need a joint univariate table of all factors studied that combining it all in the Bivariate table. It is a lazy way of reporting that require a reader to tease out proportions on their own. address this<br>Author's response: We appreciate the reviewer's comment. In response to the suggestion for a joint univariate table of all studied factors, we acknowledge the importance of presenting comprehensive data that is readily interpretable. Our analysis included an examination of crash type distributions across multiple independent variables. To explore these relationships, we employed Chi-square tests. Variables with significance levels below 0.2 were identified to minimize type II errors and were considered significantly associated with the outcome variables ($p<0.05$). These variables were subsequently included in multiple logistic regression models.<br>We utilized stepwise logistic regression to estimate odds ratios while controlling for specific factors, following a methodological approach well-established in traffic injury studies (e.g., references a and b) and detailed in previous research (e.g., reference c). This approach allows for a nuanced understanding of how various factors interact to influence crash types, ensuring our findings are robust and informative. This methodological approach is well-established in the study of traffic injuries (e.g., references a and b) and has been detailed in previous studies (e.g., reference c).<br>a: Chen, P-L, Pai, C-W. Evaluation of injuries sustained by motorcyclists in approach-turn crashes in Taiwan. Accident Analysis and Prevention, 2019, 124, 33-39;<br>b: Chien, D-K., Hwang, HF, Lin, MR. Injury severity measures for predicting return-to-work after a traumatic brain injury. Accident Analysis and Prevention, 2017, 98, 101-107;<br>c: Maldonado G, Greenland S. Simulation study of confounder-selection strategies. Am J Epidemiol 1993, 138, 11, 923-936).<br><br>4.4.2 Use one decimal place and not two<br>Author's response: We appreciate this reviewer's comment. We have updated our tables (Tables 1-4) to display data with one decimal place instead of two (please refer to lines 213 to 276; pages 12-17 in the manuscript).<br>4.4.3 After inserting a combined univariate table, please remove these percentages, as they are very misleading

Powered by Editorial Manager® and ProduXion Manager® from Aries Systems Corporation

	Author’s response: We appreciate this reviewer’s comment. However, presenting percentages is crucial for demonstrating the distribution among each crash type and others. Therefore, we have decided to continue using percentages as presentation in our manuscript.<br>4.4.5 Tables: Here put frequencies/percenatgase and removed all the percentages from the table. the same applies to all other tables<br>Author’s response: We appreciate this reviewer’s comment. Nevertheless, it is essential to use percentages to clearly demonstrate the distribution of each crash type across a set of variables. By reporting these percentages, we are able to identify whether one certain variable was over-involved in one crash type. Therefore, we have opted to maintain the use of percentages in our presentation.<br>4.4.6 All most all the bivariate table has not been interpreted. but summarize using phrases like serveral variables as shown in table 2.<br>Author’s response: We appreciate this reviewer’s comment. We have revised our results section to incorporate the reviewer's suggestion and rephrase the sentence accordingly (please refer to lines 215 to 229; page 13 in the manuscript):<br>"Several variables in Table 2 reveal significant differences between rear-end crashes and non-rear-end crashes. Specifically, a higher proportion of rear-end crashes occurred under darkness-unlit conditions (50.2%) compared to darkness-lit conditions (37.5%). Additionally, rear-end crashes were more prevalent in rural areas with speed limits of ≥ 40 mph (43.0%) compared to urban areas with speed limits of 20–30 mph(33.1%). Crashes involving crash partners aged ≥ 65 accounted for 39.7% of rear-end crashes, which was higher compared to other age groups (age 41–64: 33.0% and ≤18: 36.0%). Furthermore, rear-end crashes were more likely to occur during midnight (47.6%) compared to rush hours (36.3%). Taxis were frequently involved in rear-end crashes (42.4%), as were male crash partners (36.8%). These findings highlight the significant influence of various factors on the likelihood of rear-end crashes. Variables such as darkness-unlit conditions, higher speed limits in rural areas, crash time, and characteristics of the crash partner all emerged as significant determinants. Specifically, rear-end crashes were notably more prevalent under darkness-unlit conditions, in rural areas with higher speed limits, during midnight hours, and involving certain characteristics of crash partners. Importantly, these associations were statistically significant, as indicated by the Chi-squared test (p < 0.001)."<br><br>4,4,7 Do inteprete the results individually for all the significant factors.<br>Author’s response: We appreciate this reviewer’s comment. We have revised our discussion section to incorporate the reviewer's suggestion and rephrase the sentence accordingly (please refer to lines 232 to 248; pages 14-15 in the manuscript):<br>“As shown in Table 3, several variables can contribute to door crashes involving bicycles. Door crashes predominantly occurred in urban areas with speed limits of 20-30 mph (6.22%), while a significantly lower proportion occurred in rural areas with speed limits ≥ 40 mph (0.45%). These crashes were overrepresented during non-rush hours (5.54%) and rush hours (4.94%) compared to evening (4.26%) and midnight (2.35%). Cyclists were more frequently involved in door crashes on weekdays (5.35%) than weekends (3.73%). As many as 8.21% of all female cyclists were involved in door crashes, which is higher than the involvement rate among males (4.24%).Taxi and private hire cars were overinvolved in door crashes (10.55%) compared to cars (5.17%) and buses/heavy goods vehicles (3.13%). Crash partners aged ≤18 years (5.22%) and 19-40 years (5.26%) were disproportionately involved in door crashes compared to older age groups, and female crash partners were overrepresented in door crashes (7.42%) compared to males (4.23%). These results were statistically significant, as indicated by the Chi-squared test (p < 0.001). They suggest that various factors including traffic conditions (rural areas, crash time), cyclist demographics (younger age, gender), and characteristics of the crash partner (taxi/private hire cars)—significantly contribute to the likelihood of door crashes involving cyclists.”<br>4.4.8 where are the corresponding p-values. include them for all the significant risk factors<br>Author’s response: We appreciate this reviewer’s comment. We have revised our Results section to include the reviewer's suggestion and have added the corresponding p-values accordingly (please refer to lines 253 to 272; page 16 in the manuscript):<br>For example: “(AOR = 2.912, 95% CI = 2.384–3.556; p<0.001).”<br>4.4.9 Here, present both the crude and adjusted odd ratios<br>Author’s response: We appreciate the reviewer’s comment. In response, we have

Powered by Editorial Manager® and ProduXion Manager® from Aries Systems Corporation

focused on presenting the adjusted odds ratios (AOR) and their corresponding 95% confidence intervals in our manuscript.
To address the analysis of crash types across various independent variables, we conducted Chi-square tests to assess the association between dependent and independent variables. The direction of the independent variables will be clarified in the subsequent multivariate logistic regression models.
Significant variables identified through stepwise selection were included in the multiple logistic regression models. The adjusted odds ratios (AOR) and their 95% confidence intervals were then calculated from these final models. This approach, widely used in traffic injury studies (e.g., a, b), ensures robust methodology by controlling for other variables (e.g., c).
a: Chen, P-L, Pai, C-W. Evaluation of injuries sustained by motorcyclists in approach-turn crashes in Taiwan. Accident Analysis and Prevention, 2019, 124, 33-39;
b: Chien, D-K., Hwang, HF, Lin, MR. Injury severity measures for predicting return-to-work after a traumatic brain injury. Accident Analysis and Prevention, 2017, 98, 101-107;
c: Maldonado G, Greenland S. Simulation study of confounder-selection strategies. Am J Epidemiol 1993, 138, 11, 923-936).

4.4.10 Also do interpret these results. For instance what does the odd ratio of 2.93 mean in this case?
Author's response: We appreciate the reviewer's comment and suggestion. We have revised our results section to incorporate the reviewer's suggestion and have interpreted the meaning of odds ratios in our findings accordingly (please refer to lines 279 to 286; pages17-18 in the manuscript):
"The results identified several key risk factors for both overtaking and rear-end crashes. The risk of overtaking crashes showed a significant increase of 193% in rural areas when elderly drivers were involved (AOR = 2.93, 95% CI = 2.79–3.08), and similarly when heavy goods vehicles (HGVs) were the crash partner (AOR = 2.62, 95% CI = 2.46–2.78). Elderly cyclists also faced a higher risk of overtaking crashes on weekends (AOR = 1.56, 95% CI = 1.34–1.81).
Regarding rear-end crashes, the risk increased notably with unlit darkness during midnight (AOR = 1.68, 95% CI = 1.48–1.90) and was significantly higher in rural areas (AOR = 2.15, 95% CI = 2.01–2.31)."
4.4.11 you look at risk factors and not only environment factors, what about factors like sex, age. are they from the environment too, and yet you included them.
Author's response: We appreciate the reviewer's comment. In our multivariate logistic regression results in Table 4, we analyzed and presented such factors such as cyclist's sex and age for each crash type (please refer to lines 275 to 276; page 17 in the manuscript). Moreover, in our joint-effect analysis, cyclist's age (≥65-year-old cyclist) was combined and analyzed with other variables.

4.5 Discussion
The section also needs serious work, especially on the way the findings were discussed. The authors should consider discussing their own findings rather than those of other studies. There is also a need to have a section for recommendations rather than merging it within result
4.5.1 You dont need this type of writing, just discuss the findings
Author's response: We appreciate the reviewer's comment. We have revised our discussion section to delete the paragraph as the reviewer's suggestion as follows (please refer to lines 305; page 19 in the manuscript):
"Delete: These findings warrant further discussion and thus are elaborated on in this section of this paper."

You have not discussed the findings. Yes you found HGVs a risk for overtaking crash, so tell us why you think that is a risk factor. in other word explain your findings and then place it in the context of other study
Author's response: We appreciate the reviewer's comment and suggestion. We have revised our discussion section to integrate the reviewer's suggestion and provide a discussion on how heavy goods vehicles (HGVs) pose a risk for overtaking crashes accordingly as follows (please refer to lines 308 to 317; page 19 in the manuscript):
"These findings align with previous research that identified elderly drivers, speeds exceeding 10 mph, and the presence of pick-up trucks as factors contributing to increased risk for overtaking crash. Specifically, HGVs possess several characteristics

	that amplify this danger. Their large blind spots make it difficult for drivers to see cyclists, increasing the likelihood of crashes during overtaking. Additionally, HGVs are less maneuverable compared to passenger cars, which reduces their ability to avoid crashes if cyclists suddenly enter their path. The speed and distance perception issues between HGVs and cyclists further complicate the judgment of safe overtaking gaps. Furthermore, HGVs require longer stopping distances due to their size and weight, which can lead to severe consequences if a sudden need to brake arises."<br><br>4.5.3 Do not discuss other people's findings, just discuss your findings and only state whether it agrees of disagrees with what Pai et al found for example<br>Author's response: We appreciate the reviewer's comment and suggestion. We have revised our Discussion section to incorporate the reviewer's suggestion and provide a discussion on our findings, comparing them with previous studies accordingly (please refer to lines 318 to 322; pages 19-20 in the manuscript):<br>"Regarding the association with buses or HGVs, our findings are consistent with existing research suggesting that time pressures on HGV drivers for timely loading and unloading might lead to more reckless driving. Specifically, our results align with the observations made by Pai et al., who also mentioned higher crash rates involving buses or HGVs, supporting the idea that these time pressures contribute to increased crash risks."<br>4.5.4 Take this to the recommendation section<br>Author's response: We appreciate the reviewer's comment and suggestion. We have added a recommendations section (please refer to lines 404-412; pages 23-24 of the manuscript):<br>"Recommendations<br>For overtaking crashes, we recommend implementing 'Share the Road' warning signs, especially in rural areas, and developing specialized cognitive training programs for elderly drivers. Regarding rear-end crashes, our suggestions include improving illumination during night time and implementing speed control measures on rural road segments. For door crashes involving parked cars, we propose enhancing driver sight triangles and increasing cyclist visibility. Moreover, implementing a two-stage door opening mechanism and an automatic detection device in vehicles to alert drivers of bic...
**Additional Information:**	
**Question**	**Response**
**Financial Disclosure**<br><br>Enter a financial disclosure statement that describes the sources of funding for the work included in this submission. Review the submission guidelines for detailed requirements. View published research articles from *PLOS ONE* for specific examples.<br><br>This statement is required for submission and **will appear in the published article** if the submission is accepted. Please make sure it is accurate.	Yes

**Funded studies**<br>Enter a statement with the following details:<br>• Initials of the authors who received each award<br>• Grant numbers awarded to each author<br>• The full name of each funder<br>• URL of each funder website<br>• Did the sponsors or funders play any role in the study design, data collection and analysis, decision to publish, or preparation of the manuscript?<br><br>Did you receive funding for this work?	
Please add funding details.<br>as follow-up to "**Financial Disclosure**<br><br>Enter a financial disclosure statement that describes the sources of funding for the work included in this submission. Review the submission guidelines for detailed requirements. View published research articles from *PLOS ONE* for specific examples.<br><br>This statement is required for submission and **will appear in the published article** if the submission is accepted. Please make sure it is accurate.<br><br>**Funded studies**<br>Enter a statement with the following details:<br>• Initials of the authors who received each award<br>• Grant numbers awarded to each author<br>• The full name of each funder<br>• URL of each funder website<br>• Did the sponsors or funders play any role in the study design, data collection and analysis, decision to publish, or preparation of the manuscript?<br><br>Did you receive funding for this work?"	This study received financial support from the Ministry of Science and Technology, Taiwan (MOST 110–2410-H-038-016-MY2 and MOST 109–2314-B-038-066-); New Taipei City Hospital (NTPC113–002); and the National Science and Technology Council, Taiwan (NSTC 112-2410-H-038-016-MY2). The funders played no role in the design of the study, data collection and analysis, interpretation of data, or preparation of the manuscript.
Please select the country of your main research funder (please select carefully as in some cases this is used in fee calculation).	TAIWAN - TW

as follow-up to "**Financial Disclosure**<br><br>Enter a financial disclosure statement that describes the sources of funding for the work included in this submission. Review the submission guidelines for detailed requirements. View published research articles from *PLOS ONE* for specific examples.<br><br>This statement is required for submission and **will appear in the published article** if the submission is accepted. Please make sure it is accurate.<br><br>**Funded studies**<br>Enter a statement with the following details:<br>• Initials of the authors who received each award<br>• Grant numbers awarded to each author<br>• The full name of each funder<br>• URL of each funder website<br>• Did the sponsors or funders play any role in the study design, data collection and analysis, decision to publish, or preparation of the manuscript?<br><br>Did you receive funding for this work?"	
**Competing Interests**<br><br>Use the instructions below to enter a competing interest statement for this submission. On behalf of all authors, disclose any competing interests that could be perceived to bias this work—acknowledging all financial support and any other relevant financial or non-financial competing interests.<br><br>This statement is required for submission and **will appear in the published article** if the submission is accepted. Please make sure it is accurate and that any funding sources listed in your Funding Information later in the submission form are also declared in your Financial Disclosure statement.	The authors have declared that no competing interests exist.

View published research articles from *PLOS ONE* for specific examples.<br><br>**NO authors have competing interests**<br><br>Enter: *The authors have declared that no competing interests exist.*<br><br>**Authors with competing interests**<br><br>Enter competing interest details beginning with this statement:<br><br>*I have read the journal's policy and the authors of this manuscript have the following competing interests: [insert competing interests here]*<br><br>* typeset	
**Ethics Statement**<br><br>Enter an ethics statement for this submission. This statement is required if the study involved:<br><br>• Human participants<br>• Human specimens or tissue<br>• Vertebrate animals or cephalopods<br>• Vertebrate embryos or tissues<br>• Field research<br><br>Write "N/A" if the submission does not require an ethics statement.<br><br>General guidance is provided below. Consult the submission guidelines for detailed instructions. **Make sure that all information entered here is included in the Methods section of the manuscript.**	This study was approved by the Joint Institutional Review Board of Taipei Medical University (N202011030). The Joint Institutional Review Board of Taipei Medical University has waived the requirement of informed consent. All methods were performed in accordance with the relevant guidelines and regulations of the Declaration of Helsinki.

**Format for specific study types**<br><br>**Human Subject Research (involving human participants and/or tissue)**<br>• Give the name of the institutional review board or ethics committee that approved the study<br>• Include the approval number and/or a statement indicating approval of this research<br>• Indicate the form of consent obtained (written/oral) or the reason that consent was not obtained (e.g. the data were analyzed anonymously)<br><br>**Animal Research (involving vertebrate animals, embryos or tissues)**<br>• Provide the name of the Institutional Animal Care and Use Committee (IACUC) or other relevant ethics board that reviewed the study protocol, and indicate whether they approved this research or granted a formal waiver of ethical approval<br>• Include an approval number if one was obtained<br>• If the study involved *non-human primates*, add *additional details* about animal welfare and steps taken to ameliorate suffering<br>• If anesthesia, euthanasia, or any kind of animal sacrifice is part of the study, include briefly which substances and/or methods were applied<br><br>**Field Research**<br><br>Include the following details if this study involves the collection of plant, animal, or other materials from a natural setting:<br>• Field permit number<br>• Name of the institution or relevant body that granted permission	
**Data Availability**<br><br>Authors are required to make all data underlying the findings described fully available, without restriction, and from the time of publication. PLOS allows rare exceptions to address legal and ethical concerns. See the PLOS Data Policy and FAQ for detailed information.	Yes - all data are fully available without restriction

A Data Availability Statement describing where the data can be found is required at submission. Your answers to this question constitute the Data Availability Statement and **will be published in the article**, if accepted.<br><br>**Important:** Stating 'data available on request from the author' is not sufficient. If your data are only available upon request, select 'No' for the first question and explain your exceptional situation in the text box.<br><br>Do the authors confirm that all data underlying the findings described in their manuscript are fully available without restriction?	
**Describe where the data may be found in full sentences. If you are copying our sample text, replace any instances of XXX with the appropriate details.**<br><br>• If the data are **held or will be held in a public repository**, include URLs, accession numbers or DOIs. If this information will only be available after acceptance, indicate this by ticking the box below. For example: *All XXX files are available from the XXX database (accession number(s) XXX, XXX.*).<br>• If the data are all contained **within the manuscript and/or Supporting Information files**, enter the following: *All relevant data are within the manuscript and its Supporting Information files.*<br>• If neither of these applies but you are able to provide **details of access elsewhere**, with or without limitations, please do so. For example:<br><br>*Data cannot be shared publicly because of [XXX]. Data are available from the XXX Institutional Data Access / Ethics Committee (contact via XXX) for researchers who meet the criteria for access to confidential data.*<br><br>*The data underlying the results presented in the study are available from (include the name of the third party*	This study utilised the British STATS19 database, which contains data on all road traffic accidents in the United Kingdom. The data that support the findings of this study are openly available at https://figshare.com/ndownloader/files/48173452.

Powered by Editorial Manager® and ProduXion Manager® from Aries Systems Corporation

and contact information or URL).<br>• This text is appropriate if the data are owned by a third party and authors do not have permission to share the data.<br><br>* typeset	
Additional data availability information:	Tick here if the URLs/accession numbers/DOIs will be available only after acceptance of the manuscript for publication so that we can ensure their inclusion before publication.

Powered by Editorial Manager® and ProduXion Manager® from Aries Systems Corporation

Manuscript

Click here to access/download;Manuscript;4-28 Main Risk Factors for Overtaking.docx

Risk Factors for Overtaking, Rear-End, and Door Crashes Involving Bicycles in the United Kingdom: Revisited and Reanalysed

Chun-Chieh Chao[1,2,3†], Hon-Ping Ma[1,3,7], Li Wei[1,8,9], Yen-Nung Lin[1,10], Chenyi Chen[1], Wafaa Saleh[11], Bayu Satria Wiratama[12], Akhmad Fajri Widodo[1], Shou-Chien Hsu[6,13], Shih Yu Ko[7], Hui-An Lin[1,2,3†], Cheng-Wei Chan[1,4,5,6†], Chih-Wei Pai[1*]

[1] Graduate Institute of Injury Prevention and Control, College of Public Health, Taipei Medical University, Taipei City, Taiwan, [2] Department of Emergency Medicine, Taipei Medical University Hospital, Taipei City, Taiwan, [3] Department of Emergency Medicine, School of Medicine, College of Medicine, Taipei Medical University, Taipei, Taiwan, [4] Department of Emergency Medicine, New Taipei City Hospital, New Taipei City, Taiwan, [5] College of Medicine, Chang Gung University, Taoyuan City, Taiwan, [6] Department of Emergency Medicine, Chang Gung Memorial Hospital, Linkou branch, Taoyuan, Taiwan, [7] Department of Emergency Medicine, Taipei Medical University-Shuang Ho Hospital, New Taipei City, Taiwan, [8] Taipei Neuroscience Institute, Taipei Medical University, Taipei, Taiwan, [9] Division of Neurosurgery, Department of Surgery, Wan Fang Hospital, Taipei Medical University, Taipei, Taiwan, [10] Department of Physical Medicine and Rehabilitation, Wan Fang Hospital, Taipei Medical University, Taipei, Taiwan, [11] Transport Research Institute, Edinburgh Napier University, Edinburgh, Scotland, [12] Department of Epidemiology, Biostatistics, and Population Health, Faculty of Medicine, Public Health and Nursing, Universitas Gadjah Mada, Yogyakarta City, Indonesia, [13] Department of Occupational Medicine, Chang Gung Memorial Hospital, Linkou branch, Taoyuan, Taiwan.

* Correspondence: cpai@tmu.edu.tw

† Contributed equally to this work

Abstract

Objectives

Relevant research has provided valuable insights into risk factors for bicycle crashes at intersections. However, few studies have focused explicitly on three common types of bicycle crashes on road segments: overtaking, rear-end, and door crashes.

Material and methods

The present study investigated risk factors for these three crash types on road segments. We analysed British STATS19 accident records from 1991 to 2020. Using multivariate logistic regression models, we estimated adjusted odds ratios (AORs) with 95% confidence intervals (CIs) for multiple risk factors. The analysis included 127,637 bicycle crashes, categorised into 18,350 overtaking, 44,962 rear-end, 6,363 door, and 57,962 other crashes.

Results

Significant risk factors for overtaking crashes included speed limits of ≥40 miles per hour (mph) (AOR = 2.238, 95% CI = 2.159–2.320), heavy goods vehicles (HGVs) as crash partners (AOR = 2.867, 95% CI 2.473–3.323), and elderly crash partners (AOR = 2.013, 95% CI = 1.937–2.092). For rear-end crashes, noteworthy risk factors included unlit darkness (AOR = 1.486, 95% CI = 1.404–1.573) and midnight hours (AOR = 1.269, 95% CI = 1.190–1.354). Factors associated with door crashes included speed limits of 20–30 mph (AOR = 16.185, 95% CI = 13.514–19.382) and taxi and private hire cars (AOR = 2.695, 95% CI = 2.310–3.145). Our joint-effect analysis revealed additional interesting results; for example, there were elevated risks for overtaking crashes in rural areas with elderly drivers as crash partners (AOR = 2.93, 95% CI = 2.79–3.08) and with HGVs as crash partners (AOR = 2.62, 95% CI = 2.46–2.78).

Conclusions

The aforementioned risk factors remained largely unchanged since 2011, when we conducted our previous study. However, the present study concluded that the detrimental effects of certain variables became more pronounced in certain situations. For example, cyclists in rural settings exhibited an elevated risk of overtaking crashes involving HGVs as crash partners.

Keywords: Bicycle crash; Road segment; Overtaking crash; Rear-end crash; Door crash

Introduction

In recent years, urban bicycling has become increasingly popular in many countries, offering

benefits such as reduced traffic congestion, diminished parking pressure, and a reduction in greenhouse gas emissions [1, 2]. The World Health Organization has highlighted numerous health advantages of moderate-intensity physical activities such as bicycling, including improvements in life expectancy, quality of life, cognitive function, mental health, sleep quality, muscular and cardiorespiratory fitness, and bone and functional health [2].

However, despite such health benefits, the risk of injury remains a considerable safety concern for cyclists, who are regarded as vulnerable road users [2, 3]. Traffic crash data indicate that the risk of accidents for cyclists, measured per distance travelled, is approximately 20 times higher than that for vehicle drivers [2]. To address this problem, researchers in the United States developed a comprehensive bicycle route safety rating model with a focus on injury severity [4]. This model evaluates multiple operational and physical aspects such as traffic volume, population density, highway classification, lane width, and the presence of one-way streets. In addition, it is capable of predicting the severity of injuries due to motor vehicle–related crashes at specific locations [4]. Another finding was that a route is considered adequately safe if it includes geometric factors that enhance safety [4]. This model can aid urban planners and public officials in creating infrastructure such as bike lanes and implementing strict lane policies to improve cyclist safety [4]. Implementing bike lanes has been demonstrated to reduce crash rates by up to 40% among adult cyclists [5]. One study regarding roundabouts indicated that roundabouts with cycle tracks significantly reduced injury risk for cyclists compared with those lacking bicycle infrastructure [6]. Furthermore, adequate night-time lighting on rural roads has the potential to prevent over half of all cyclist injuries [7].

Although intersectional crashes are generally more frequent than nonintersectional ones, in

2020, 64% of fatal crashes involving cyclists occurred on road segments, defined as areas 20 m away from intersections, whereas only 26% of such fatalities occurred at intersections [8]. Bil et al. demonstrated that car drivers, when at fault for crashes, often cause more serious consequences for cyclists on straight road sections [9]. In crashes occurring on road segments, several factors contribute to high injury severity, including being in a rural region with an elevated speed limit, male gender, and cyclist age of >55 years [10]. Another identified risk factor is bicycling on roads against oncoming traffic [11].

Although relevant research has shed light on risk factors for bicycle crashes at intersections, few studies have explicitly investigated crashes on road segments. Studies that have examined bicycle crashes relatively broadly, without distinguishing crash types, have identified several key factors—including vehicle volume [13], traffic density [12], number of lanes [12], access points along road segments [13], shoulder and median widths [13], parking space availability [12, 13], length of continuous two-way left-turn lanes [13], and pavement type [14]—all of which contribute to crashes on road segments. Several studies have specifically explored overtaking, rear-end, and door crashes involving bicycles. A pioneering contribution in this area was made by Pai, who focused on these three types of crashes on road segments [15]. Specifically, Pai identified buses and coaches as common crash partners in overtaking crashes; poor visibility, traversing manoeuvres, and teenage cyclists as risk factors for rear-end crashes; and built-up areas as a risk factor for door crashes [15]. In addition, another study linked the speed of a passing vehicle to increased severity of cyclist injury in overtaking crashes [16].

The primary objective of the present study, an extension of our previous study [15], was to analyse police-reported crash data from additional years to determine whether the risk factors

for these three crash types remained unchanged. Furthermore, we aimed to untangle the joint associations of several factors—including light conditions, urban versus rural settings, vehicle types, and rider and driver characteristics—with these three crash types.

Material and Methods

Crash data source

The present investigation utilised data from 01/01/1991 to 31/12/2020, obtained from the United Kingdom's official road traffic casualty database, STATS19. Police record such data either at crash scenes or within 30 days of each crash. The UK's Department for Transport compiles the data, which the United Kingdom Data Archive then maintains and distributes. The dataset encompasses a variety of variables, including crash circumstances (e.g., time and date, weather conditions, road and light conditions, posted speed limit, road type), vehicle and driver characteristics, demographic details of the drivers, precrash manoeuvres of the vehicles, and the initial impact point of the vehicle. Additionally, the dataset contains demographic information and details regarding injury severity for each casualty. This study adhered to the STROBE (strengthening the reporting of observational studies in epidemiology) reporting guidelines.

Injury severity in the aforementioned dataset is divided into three categories, namely slight, serious, and fatal. Fatal injuries refer to those leading to death within 30 days of the accident. Serious injuries include conditions such as fractures, internal injuries, severe cuts and lacerations, concussions, and any injury requiring hospitalisation. Slight injuries include sprains, bruises, and minor cuts, as well as mild shock requiring roadside attention. The exclusive focus of this study was crashes leading to cyclist casualties.

As shown in Figure 1, this study analysed 1,366,196 crashes involving bicycles and other vehicles. Initially, 1,235,032 junction cases were excluded. From the remaining 131,164 bicycle segment crashes, 3,527 were further excluded because of incomplete demographic data for the cyclist and missing speed limit information, leaving a valid cohort of 127,637 bicycle segment crashes for analysis. Within this cohort, this study identified 18,350 overtaking crashes, 44,962 rear-end crashes, 6,363 door crashes, and 57,962 other types of crashes.

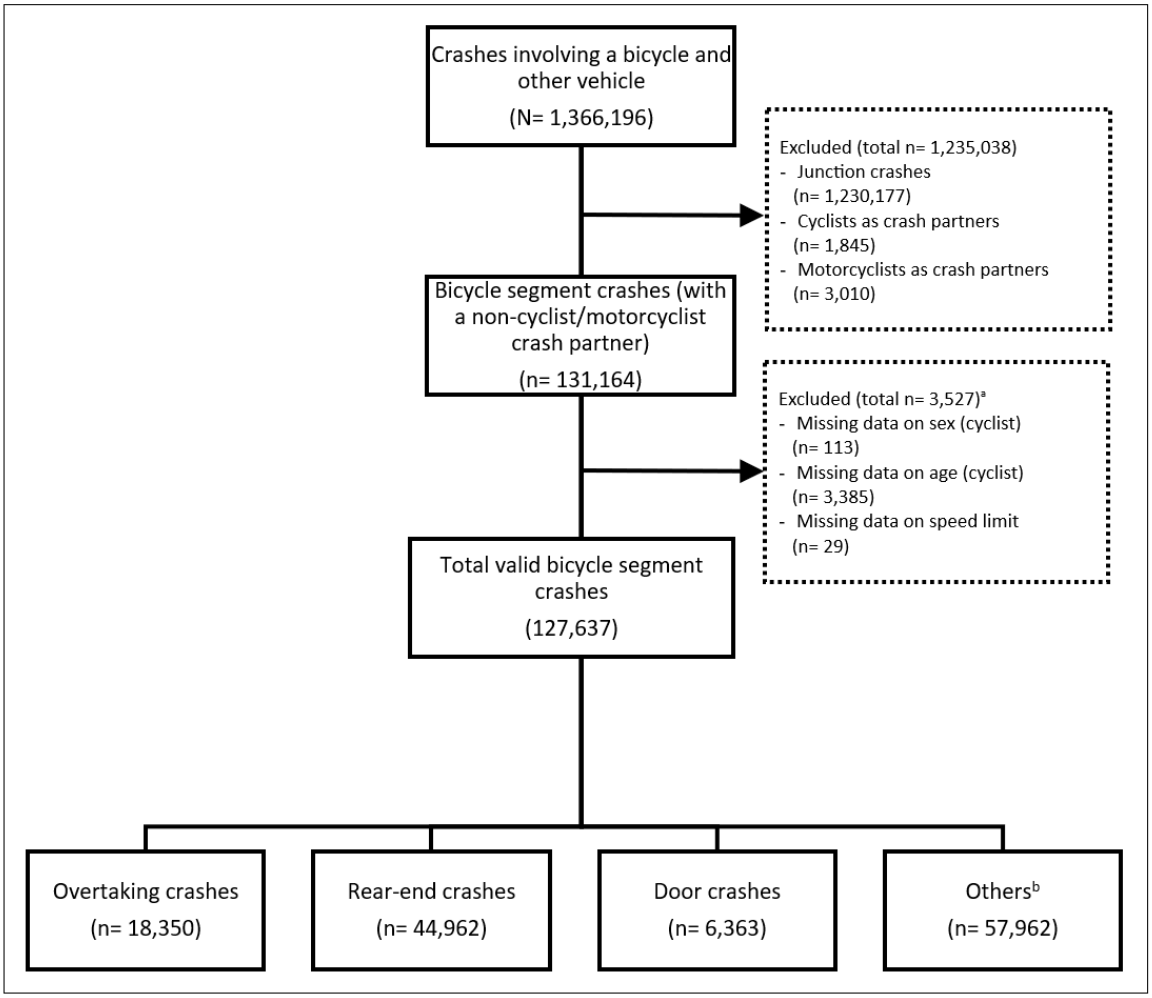

Fig. 1. Flowchart of the study sample selection process. [a]Listed excluded criteria are nonexclusive; thus, the sum of the total may exceed 3,527. [b]Other crashes include reversing crashes and head-on crashes.

Classification of crash types

An overtaking crash is defined as a crash where a motorised vehicle overtakes and collides with

a bicycle, which may be travelling straight, overtaking another vehicle, changing lanes, or turning. A rear-end crash occurs when a following vehicle collides with the rear of a bicycle. A door crash involves a bicycle either being struck by or striking the opening door of an automobile. These three crash types were described using schematics in our previous study [15].

Data collection

For the present study, the three crash types of focus (overtaking, rear-end, and door crashes) were the binary-dependent variables. The collected data encompassed the following factors: lighting conditions on the roadway at the time of the crash (daylight, darkness-lit, darkness-unlit), the speed limit at the crash scene (rural: ≥40 miles per hour [mph]; urban: 20–30 mph), the time of day categorised into four periods according to traffic volume (midnight: 00:00–06:00; rush hours: 07:00–08:00 and 17:00–18:00; nonrush hours: 09:00–16:00; and evening: 19:00–23:00), and the day of the week (weekday or weekend day). The demographic details of cyclist casualties encompassed age (≤18, 19–40, 41–64, or ≥65 years) and sex (male or female). Finally, the demographic details of the crash partner included the type of vehicle (identified as a taxi, private hire car, car, bus, or heavy goods vehicle [HGV]), age (≤18, 19–40, 41–64, or ≥65 years) and sex (male or female).

Statistical analysis

This study employed the chi-squared test to examine the associations between crash type and other factors, including cyclist or motorist characteristics, vehicle features, roadway conditions, and temporal variables. Variables with a *p* value lower than 0.2 in the univariate analysis were

subsequently incorporated into the multivariate logistic regression analysis [17]. All statistical analyses were conducted using SPSS Statistics version 25 for Windows (IBM Corp., Armonk, New York, USA). A p value lower than 0.05 in two-tailed tests was considered statistically significant.

Results

Population characteristics

Tables 1, 2, and 3 present the distributions of overtaking, rear-end, and door crashes, respectively, in relation to multiple independent variables. These data revealed that a significant proportion of bicycle crashes occurred in daylight (82.31%), occurred in urban settings (78.54%), occurred during nonrush hours (48.34%), occurred on weekdays (77.49%), involved cyclists aged under 18 years (40.11%), and involved male cyclists (81.30%). Additionally, most crashes involved cars as crash partners (83.57%), and crash partners were predominately aged 19–40 years (38.47%) and were male (76.35%). Table 1 highlights an overrepresentation in bicycle overtaking crashes for certain variables, namely unlit darkness (19.50%), rural areas (24.84%), midnight hours (17.71%), buses or HGVs as crash partners (24.72%), and elderly crash partners (21.47%) and male crash partners (15.99%). These results were revealed to be statistically significant by the chi-squared test ($p < 0.01$).

Table 1. Distribution of overtaking crashes according to a set of independent variables

**Variable**	**Total (n=127,637)**	**Overtaking crashes (n=18,350)**	**Non-overtaking crashes (n=109,287)**	***χ2 test p value***
**Light conditions**, n (%)				<0.001
Daylight	105,053 (82.31%)	15,283 (14.55%)	89,770 (85.45%)	
Darkness-lit	16,543 (12.96%)	1,889 (11,42%)	14,654 (88.58%)	
Darkness-unlit	6,041 (4.73%)	1,178 (19.50%)	4,863 (80.50%)	

Table 1. Distribution of overtaking crashes according to a set of independent variables *(continued)*

Variable	Total (n=127,637)	Overtaking crashes (n=18,350)	Non-overtaking crashes (n=109,287)	*χ2 test p value*
**Speed limit**, n (%)				<0.001
Rural (≥ 40 mph)	27,395 (21.46%)	6,805 (24.84%)	20,590 (75.61%)	
Urban (20–30 mph)	100,242 (78.54%)	11,545 (11.52%)	88,697 (88.48%)	
**Crash time (h)**, n (%)				<0.001
Midnight (00:00–06:00)	4,810 (3.77%)	852 (17.71%)	3,958 (82.29%)	
Rush hours (07:00–08:00/17:00–18:00)	41,619 (32.61%)	5,685 (13.66%)	35,934 (86.34%)	
Nonrush hours (09:00–16:00)	61,696 (48.34%)	9,386 (15.21%)	52,310 (84.79%)	
Evening (19:00–23:00)	19,512 (15.29%)	2,427 (12.44%)	17,085 (87.56%)	
**Crash day**, n (%)				0.094
Weekend	28,730 (22.51%)	4,218 (14.68%)	24,512 (85.21%)	
Weekday	98,907 (77.49%)	14,132 (14.29%)	84,775 (85.71%)	
**Cyclist's age (years)**, n (%)				<0.001
≤18	51,193 (40.11%)	5,220 (10.20%)	45,973 (89.80%)	
19–40	45,760 (35.85%)	7,108 (15.53%)	38,652 (84.47%)	
41–64	26,052 (20.41%)	5,012 (19.24%)	21,040 (80.76%)	
≥65	4,632 (3.63%)	1,010 (21.80%)	3,622 (78.20%)	
**Cyclist's sex**, n (%)				<0.001
Male	103,766 (81.30%)	14,746 (14.21%)	89,020 (85.79%)	
Female	23,871 (18.70%)	3,604 (15.10%)	20,267 (84.90%)	
**Crash partner**, n (%)				<0.001
Taxi/Private hire car	2,588 (2.03%)	208 (8.04%)	2,380 (91.96%)	
Car	106,668 (83.57%)	13,599 (12.75%)	93,069 (87.25%)	
Bus/Heavy goods vehicle	18,381 (14.40%)	4,543 (24.72%)	13,838 (75.28%)	
**Crash partner's age (years)**, n (%)				<0.001
≤18	2,415 (1.89%)	281 (11.64%)	2,134 (88.36%)	
19–40	49,103 (38.47%)	5,398 (10.99%)	43,705 (89.01%)	
41–64	35,598 (27.89%)	3,973 (11.16%)	31,625 (88.84%)	
≥65	40,521 (31.75%)	8,698 (21.47%)	31,823 (78.53%)	
**Crash partner's sex**, n (%)				<0.001
Male	97,447 (76.35)	15,584 (15.99%)	81,863 (84.01%)	
Female	30,190 (23.765%)	2,766 (9.16%)	27,424 (90.84%)	

As reported in Table 2, several variables, for instance, unlit darkness (50.19%), rural areas (43.03%), in midnight hours (47.59%), taxis as crash partners (42.35%), and elderly (39.67%) or male crash partners (36.77%) appeared to be disproportionately represented in bicycle rear-end crashes. These results were also revealed to be statistically significant by the chi-squared test ($p < 0.01$).

Table 2. Distribution of rear-end crashes according to a set of independent variables

Variable	Total (n=127,637)	Rear-end crashes (n=44,962)	Non-rear-end crashes (n=82,675)	*χ2 test p value*
**Light conditions**, n (%)				<0.001
Daylight	105,053 (82.31%)	35,726 (34.10%)	69,333 (66.00%)	
Darkness-lit	16,543 (12.96%)	6,204 (37.50%)	10,339 (63.50%)	
Darkness-unlit	6,041 (4.73%)	3,032 (50.19%)	3,003 (49.71%)	

Table 2. Distribution of rear-end crashes according to a set of independent variables *(continued)*

**Variable**	**Total (n=127,637)**	**Rear-end crashes (n=44,962)**	**Non-rear-end crashes (n=82,675)**	***χ2 test p value***
**Speed limit**, n (%)				<0.001
Rural (≥ 40 mph)	27,395 (21.46%)	11,788 (43.03%)	15,607 (56.97%)	
Urban (20–30 mph)	100,242 (78.54%)	33,174 (33.09%)	67,068 (66.91%)	
**Crash time (h)**, n (%)				<0.001
Midnight (00:00–06:00)	4,810 (3.77%)	2,289 (47.59%)	2,521 (52.41%)	
Rush hours (07:00–08:00/17:00–18:00)	41,619 (32.61%)	15,089 (36.26%)	26,530 (63.74%)	
Nonrush hours (09:00–16:00)	61,696 (48.34%)	20,723 (33.59%)	40,973 (66.41%)	
Evening (19:00–23:00)	19,512 (15.29%)	6,861 (36.16%)	12,651 (64.85%)	
**Crash day**, n (%)				<0.001
Weekend	28,730 (22.51%)	9,485 (33.01%)	19,245 (66.99%)	
Weekday	98,907 (77.49%)	35,477 (35.87%)	63,430 (64.13%)	
**Cyclist's age (years)**, n (%)				<0.001
≤18	51,193 (40.11%)	13,446 (26.27%)	37,747 (73.73%)	
19–40	45,760 (35.85%)	19,102 (41.74%)	26,658 (58.26%)	
41–64	26,052 (20.41%)	10,619 (40.76%)	15,433 (59.24%)	
≥65	4,632 (3.63%)	1,795 (38.75%)	2,837 (61.25%)	
**Cyclist's sex**, n (%)				<0.001
Male	103,766 (81.30%)	37,175 (35.83%)	66,591 (64.17%)	
Female	23,871 (18.70%)	7,787 (32.62%)	16,084 (67.38%)	
**Crash partner**, n (%)				<0.001
Taxi/Private hire car	2,588 (2.03%)	1,096 (42.35%)	1,492 (57.65%)	
Car	106,668 (83.57%)	37,202 (34.88%)	71,342 (66.88%)	
Bus/Heavy goods vehicle	18,381 (14.40%)	6,664 (36.25%)	9,841 (53.54%)	
**Crash partner's age (years)**, n (%)				<0.001
≤18	2,415 (1.89%)	870 (36.02%)	1,545 (63.98%)	
19–40	49,103 (38.47%)	16,282 (33.16%)	32,821 (66.84%)	
41–64	35,598 (27.89%)	11,736 (32.97%)	23,862 (67.03%)	
≥65	40,521 (31.75%)	16,074 (39.67%)	24,447 (60.33%)	
**Crash partner's sex**, n (%)				<0.001
Male	97,447 (76.35%)	35,828 (36.77%)	61,619 (63.23%)	
Female	30,190 (23.65%)	9,134 (30.26%)	21,056 (69.74%)	

Table 3 demonstrates that cyclists in several conditions, such as in unlit darkness (6.23%), in urban areas (6.22%), when they were female (8.21%), when taxi/private hire car were crash partners (10.55%), and when crash partners were female (7.42%), exhibited a higher risk of door crashes. These results were revealed to be statistically significant by the chi-squared test ($p < 0.01$).

Table 3. Distribution of door crashes according to a set of independent variables

Variable	Total (n=127,637)	Door crashes (n=6,363)	Non-door crashes (n=121,274)	*χ2 test p value*
**Light conditions**, n (%)				<0.001
Daylight	105,053 (82.31%)	5,192 (4.94%)	99,861 (95.06%)	
Darkness-lit	16,543 (12.96%)	1,031 (6.23%)	15,512 (93.77%)	
Darkness-unlit	6,041 (4.73%)	140 (2.32%)	5,901 (97.68%)	
**Speed limit**, n (%)				<0.001
Rural (≥ 40 mph)	27,395 (21.46%)	123 (0.45%)	27,272 (99.55%)	
Urban (20–30 mph)	100,242 (78.54%)	6,240 (6.22%)	94,002 (93.78%)	
**Crash time (h)**, n (%)				<0.001
Midnight (00:00–06:00)	4,810 (3.77%)	113 (2.35%)	4,697 (97.65%)	
Rush hours (07:00–08:00/17:00–18:00)	41,619 (32.61%)	2,056 (4.94%)	39,563 (95.06%)	
Nonrush hours (09:00–16:00)	61,696 (48.34%)	3,363 (5.54%)	58,333 (94.55%)	
Evening (19:00–23:00)	19,512 (15.29%)	831 (4.26%)	18,681 (95.74%)	
**Crash day**, n (%)				<0.001
Weekend	28,730 (22.51%)	1,072 (3.73%)	27,658 (96.27%)	
Weekday	98,907 (77.49%)	5,291 (5.35%)	93,616 (94.65%)	
**Cyclist's age (years)**, n (%)				<0.001
≤18	51,193 (40.11%)	802 (1.57%)	50,391 (98.43%)	
19–40	45,760 (35.85%)	3,474 (7.59%)	42,286 (93.41%)	
41–64	26,052 (20.41%)	1,773 (6.81%)	24,279 (93.19%)	
≥65	4,632 (3.63%)	314 (6.78%)	4,318 (93.22%)	
**Cyclist's sex**, n (%)				<0.001
Male	103,766 (81.30%)	4,404 (4.24%)	99,362 (95.76%)	
Female	23,871 (18.70%)	1,959 (8.21%)	21,912 (91.79%)	
**Crash partner**, n (%)				<0.001
Taxi/Private hire car	2,588 (2.03%)	273 (10.55%)	2,315 (89.45%)	
Car	106,668 (83.57%)	5,514 (5.17%)	101,154 (94.83%)	
Bus/Heavy goods vehicle	18,381 (14.40%)	576 (3.13%)	17,805 (96.87%)	
**Crash partner's age (years)**, n (%)				<0.001
≤18	2,415 (1.89%)	1,62 (5.22%)	2,253 (93.29%)	
19–40	49,103 (38.47%)	2,585 (5.26%)	46,518 (94.74%)	
41–64	35,598 (27.89%)	1,887 (5.30%)	33,711 (94.70%)	
≥65	40,521 (31.75%)	1,729 (4.27%)	38,792 (95.73%)	
**Crash partner's sex**, n (%)				<0.001
Male	97,447 (76.35%)	4,123 (4.23%)	93,324 (95.77%)	
Female	30,190 (23.65%)	2,240 (7.42%)	27,950 (92.58%)	

Risk factors for the three crash types

Table 4 presents the logistic regression model results. Regarding overtaking crashes, the identified risk factors included daylight conditions (adjusted odds ratio [AOR] = 1.233, 95% confidence interval [CI] = 1.162–1.309), speed limits of ≥40 mph (AOR = 2.238, 95% CI = 2.159–2.320), nonrush hours (AOR = 1.091, 95% CI 1.031–1.154), cyclists aged ≥65 years (AOR = 1.785,

95% CI = 1.649–1.931), female cyclists (AOR = 1.106, 95% CI = 1.062–1.153), HGVs as crash partners (AOR = 2.867, 95% CI = 2.473–3.323), elderly crash partners (AOR = 2.013, 95% CI = 1.937–2.092), and male crash partners (AOR = 1.353, 95% CI = 1.292–1.416).

For rear-end crashes, noteworthy risk factors included unlit darkness (AOR = 1.486, 95% CI = 1.404–1.573), speed limits of ≥40 mph (AOR = 1.315, 95% CI = 1.277–1.354), weekdays (AOR = 1.090, 95% CI = 1.059–1.122), midnight hours (AOR = 1.269, 95% CI = 1.190–1.354), and taxis as crash partners (AOR = 1.286, 95% CI = 1.186–1.394).

Regarding door crashes, significant risk factors included lit darkness (AOR = 1.373, 95% CI = 1.141–1.651), speed limits of 20–30 mph (AOR = 16.185, 95% CI = 13.514–19.382), weekdays (AOR = 1.246, 95% CI = 1.162–1.336), and nonrush hours (AOR = 2.912, 95% CI = 2.384–3.556). Additionally, female cyclists (AOR = 1.675, 95% CI = 1.582–1.774), taxis or private hire cars as crash partners (AOR = 2.695, 95% CI = 2.310–3.145), male crash partners (AOR = 1.373, 95% CI = 1.296–1.455), and crash partners aged 41–64 years (AOR = 1.855, 95% CI = 1.625–2.117) were associated with door crashes.

Table 4. Multivariate logistic regression results

Variable	Overtaking crashes		Rear-end crashes		Door crashes	
	AOR (95% CI)	*p* value	AOR (95% CI)	*p* value	AOR (95% CI)	*p* value
**Light condition**						
Daylight	1.233 (1.162, 1.309)	<0.001	Ref		1.146 (0.958, 1.370)	0.137
Darkness-lit	Ref		1.042 (1.002, 1.085)	0.041	1.373 (1.141, 1.651)	0.001
Darkness-unlit	1.152 (1.059, 1.253)	0.001	1.486 (1.404, 1.573)	<0.001	Ref	
**Speed limit**						
Rural (≥40 mph)	2.238 (2.159, 2.320)	<0.001	1.315 (1.277, 1.354)	<0.001	Ref	
Urban (20–30 mph)	Ref		Ref		16.185 (13.514, 19.382)	<0.001
**Crash time**						
Midnight	1.073 (0.982, 1.173)	0.119	1.269 (1.190, 1.354)	<0.001	Ref	
Rush hours	1.059 (1.002, 1.120)	0.043	1.108 (1.078, 1.139)	<0.001	2.502 (2.051, 3.052)	<0.001
Nonrush hours	1.091 (1.031, 1.154)	0.003	Ref		2.912 (2.384, 3.556)	<0.001
Evening	Ref		0.992 (0.953, 1.032)	0.686	2.014 (1.646, 2.465)	<0.001

Table 4. Multivariate logistic regression results *(continued)*

Variable	Overtaking crashes		Rear-end crashes		Door crashes	
	**AOR (95% CI)**	***p* value**	**AOR (95% CI)**	***p* value**	**AOR (95% CI)**	***p* value**
**Crash day**						
Weekend	1.031 (0.991, 1.072)	0.132	Ref		Ref	
Weekday	Ref		1.090 (1.059, 1.122)	<0.001	1.246 (1.162, 1.336)	<0.001
**Cyclist's age (years)**						
≤18	Ref		Ref		Ref	
19–40	1.292 (1.242, 1.345)	<0.001	1.839 (1.788, 1.891)	<0.001	5.943 (5.489, 6.435)	<0.001
41–64	1.509 (1.444, 1.578)	<0.001	1.731 (1.676, 1.789)	<0.001	6.129 (5.621, 6.684)	<0.001
≥65	1.785 (1.649, 1.931)	<0.001	1.671 (1.568, 1.780)	<0.001	5.988 (5.217, 6.874)	<0.001
**Cyclist's sex**						
Male	Ref		1.172 (1.137, 1.208)	<0.001	Ref	
Female	1.106 (1.062, 1.153)	<0.001	Ref		1.675 (1.582, 1.774)	<0.001
**Crash partner**						
Taxi/Private hire car	Ref		1.286 (1.186, 1.394)	<0.001	2.695 (2.310, 3.145)	<0.001
Car	1.571 (1.359, 1.816)	<0.001	Ref		2.089 (1.908, 2.286)	<0.001
Bus/Heavy goods vehicle	2.867 (2.473, 3.323)	<0.001	1.099 (1.061, 1.139)	<0.001	Ref	
**Crash partner's age (years)**						
≤18	1.097 (0.963, 1.249)	0.162	1.225 (1.188, 1.263)	<0.001	1.507 (1.313, 1.731)	<0.001
19–40	Ref		1.038 (1.008, 1.069)	0.013	1.855 (1.625, 2.117)	<0.001
41–64	0.950 (0.909, 0.994)	0.025	Ref		1.801 (1.574, 2.060)	<0.001
≥65	2.013 (1.937, 2.092)	<0.001	1.241 (1.137, 1.355)	<0.001	Ref	
**Crash partner's sex**						
Male	1.353 (1.292, 1.416)	<0.001	1.150 (1.117, 1.185)	<0.001	1.373 (1.296, 1.455)	<0.001
Female	Ref		Ref		Ref	

Figure 2 presents a forest plot demonstrating the joint effects of several variables on the three crash types when other variables were controlled for. An elevated risk of overtaking crashes was evident in rural areas with elderly drivers as crash partners (AOR = 2.93, 95% CI = 2.79–3.08), HGVs as crash partners (AOR = 2.62, 95% CI = 2.46–2.78), and elderly cyclists involved in accidents during weekends (AOR = 1.56, 95% CI = 1.34-1.81). The risk of rear-end crashes was increased by the synergistic interaction of unlit darkness with midnight (AOR = 1.68, 95% CI = 1.48–1.90) and by rural areas (AOR = 2.15, 95% CI = 2.01–2.31). Furthermore, bicycling at midnight in rural areas was associated with an increased risk of rear-end crashes (AOR = 1.68; 95% CI = 1.51–1.86). In urban settings, the risk of door crashes was higher for female cyclists (AOR = 2.29; 95% CI = 2.17–

2.43) and for elderly cyclists (AOR = 2.06; 95% CI = 1.82–2.34). Finally, female cyclists exhibited a 112% higher likelihood of door crashes when the crash partner was a taxi (AOR = 2.12; 95% CI = 1.68–2.69).

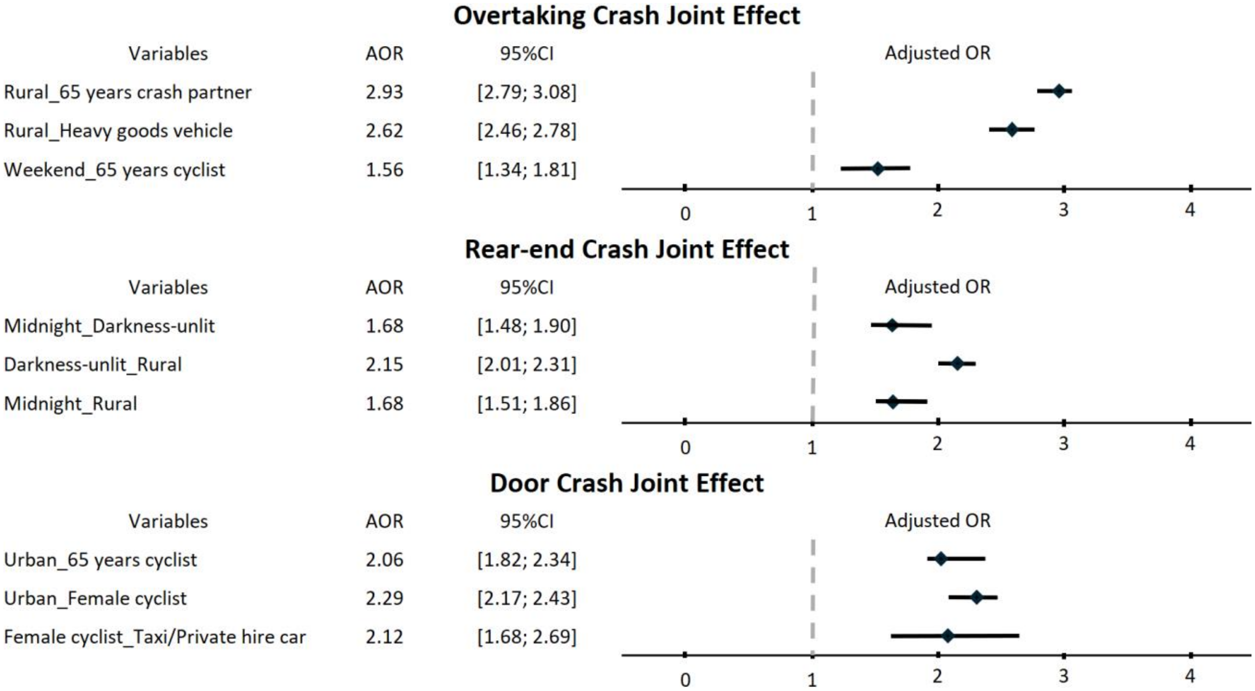

Fig. 2. Joint effects of several variables on the three crash types.

Discussion

This study explored the relationships among individual and environmental factors in relation to three common bicycle crash types (overtaking, rear-end, and door crashes) on roads in the United Kingdom from 1991 to 2020. The findings revealed several significant factors. First, for overtaking crashes, HGVs as crash partners, rural areas, and the involvement of elderly crash partners emerged as key contributing factors. Second, unlit darkness, midnight hours, and rural areas were the factors most closely associated with rear-end crashes. Third, urban areas and taxis as crash partners significantly increased the likelihood of door crashes. Moreover, male crash partners were found to be a consistent risk factor across all three crash types. These findings

warrant further discussion and thus are elaborated on in this section of this paper.

Our research findings identified specific risk factors for overtaking crashes, namely rural areas, HGVs as crash partners, and elderly crash partners. These findings align with those of a previous study [18], which similarly observed that elderly drivers, driving speeds surpassing 10 mph, and the presence of pick-up trucks increased the overtaking crash risk. We further found that the detrimental effect of HGVs on overtaking crashes was more pronounced in rural areas and when the crash partner was elderly. A behavioural study suggested that compared with cars, HGVs tended to maintain a narrower clearance zone when overtaking bicycles [19]. Pai et al. [15] speculated that the time pressures on HGV drivers for timely loading and unloading might lead to reckless driving. Our findings underscore the necessity of implementing measures such as 'Share the Road' warning signs [20], particularly in rural settings, where HGVs are likely to execute overtaking manoeuvres at high speed. Such measures could prompt motor vehicles to maintain safer distances from the edges of travel lanes, especially in areas with a notable presence of both HGVs and bicycles.

We also identified elderly drivers as a factor contributing to overtaking crashes—a finding consistent with relevant research [18]. As individuals age, their risk of being involved in road accidents is influenced by declines in their cognitive capabilities [21], their health [22], and their driving performance [23]. Notably, crashes involving elderly individuals often occur in scenarios with challenging conditions, including at intersections without traffic control measures, on high-speed roads, during adverse weather conditions, in poorly lit areas, and in head-on accidents [24–26]. The heightened level of risk under such conditions may be attributed to cognitive and perceptual decline in older drivers, which could affect their capacity to execute actions such as

overtaking manoeuvres safely. Accordingly, developing specialised cognitive training programmes as interventions to enhance road safety for elderly drivers is evidently necessary [27].

In the present study, several factors were found to increase the risk of rear-end crashes on road segments, including darkness with unlit surroundings, midnight hours, and rural settings (speed limit > 40 mph). Although few studies have specifically addressed rear-end crashes involving bicycles on road segments, available data suggest that the low conspicuity of bicycles, especially at night, is a recurrent factor in rear-end crashes [15, 28]. Moreover, a lack of adequate street lighting, which is common in rural settings, predisposes cyclists to rear-end crashes [15]. Our joint-effects analysis further indicated that the detrimental effect of unlit darkness is more pronounced in rural areas and during midnight hours. Potential intervention strategies to mitigate rear-end crashes include enhancing illumination and executing speed control management on rural road segments with heavy bicycle traffic.

Next, our analysis successfully identified associations of urban areas and taxis and private hire cars as crash partners with door crashes on road segments. Although research specifically focusing on door crashes on road segments is limited, similar findings were documented by Pai, indicating that urban roadways and taxis contributed to door crashes [15]. However, determining the factors influencing this trend poses a challenge. One possible explanation could be the increased presence of taxis or private hire cars in such areas, where passengers often disembark. Additionally, our analysis further revealed an elevated risk of door crashes involving crashes with taxis in urban areas. To reduce door crashes on road segments, educating taxi drivers, as well as passengers, about the importance of vigilance when opening doors near traffic is essential [15].

In addition, cyclists should be advised to maintain at least a door's width distance from all parked cars to improve the sight triangles of drivers and increase the visibility of cyclists [29]. Implementing a two-stage door opening mechanism for vehicles, which would enable drivers to verify the presence of bicycles to the rear, could also be beneficial [30].

This study had several limitations that warrant acknowledgement. First, the substantial underreporting of nonfatal casualties to the police, particularly casualties involving cyclists not obligated to report accidents, is a critical factor to consider. Such underreporting, as highlighted by the U.K. Government's Department for Transport [31], likely results in the incomplete representation of nonfatal and 'slight' casualties in road casualty data. Second, the STATS19 data utilised in this study lack critical variables, including precrash speeds, specific geometric characteristics of roadways, data regarding alcohol and illicit substance use, and cyclist speed at the time of an accident. Moreover, critical exposure data—such as those related to traffic flow, rider or driver experience, and other elements of risk exposure—are absent, and the absence of such details limits our ability to fully account for potential variations resulting from unobserved factors in the analyses. Finally, this study did not explore annual trends in each type of bicycle crash over the 30-year study period; investigating such trends could provide insights regarding changing behaviours among cyclists and motor vehicle drivers as well as the effects of legislative changes for road speed limits.

Conclusions

This study identified several significant risk factors for the three predominate types of crashes involving cyclists on road segments: HGVs as crash partners, elderly crash partners, and rural

areas for overtaking crashes; unlit darkness, midnight hours, and rural areas for rear-end crashes; and urban areas and taxis as crash partners for door crashes. These risk factors remained unchanged since our previous study conducted in 2011 [15]. The present research enhances the field of bicycle safety research by concluding that the detrimental effects of certain variables become more pronounced under certain conditions. For example, first, cyclists in rural settings exhibited an elevated risk of overtaking crashes involving HGVs. Second, the rear-end crash risk increases in the combined presence of unlit darkness, midnight hours, and rural areas. Finally, in urban settings, the likelihood of door crashes increases when a taxi is the crash partner.

Abbreviations

WHO: World Health Organization; HGVs: heavy goods vehicles; AOR: adjusted odds ratio; CI: confidence interval.

Acknowledgments

This manuscript was edited by Wallace Academic Editing.

Author contributions

Literature review: Chun-Chieh Chao.

Methodology: Chun-Chieh Chao, Chih-Wei Pai.

Data merging and analysis: Akhmad Fajri Widodo , Wafaa Saleh, Bayu Satria Wiratama.

Writing - original draft: Chun-Chieh Chao.

Writing – review and editing: Hui-An Lin, Chenyi Chen, Hon-Ping Ma, Akhmad Fajri Widodo.

Validation: Chun-Chieh Chao, Hui-An Lin, Chenyi Chen, Hon-Ping Ma, Shih Yu Ko.

Supervision: Li Wei, Yen-Nung Lin, Shou-Chien Hsu, Chih-Wei Pai.

Funding: Cheng-Wei Chan, Chih-Wei Pai.

Funding

This study received financial support from the Ministry of Science and Technology, Taiwan (MOST 110–2410-H-038-016-MY2 and MOST 109–2314-B-038-066-); New Taipei City Hospital (NTPC113–002); and the National Science and Technology Council, Taiwan (NSTC 112-2410-H-038-016-MY2). The funders played no role in the design of the study, data collection and analysis, interpretation of data, or preparation of the manuscript.

Availability of data and materials

This study utilised the British STATS19 database, which contains data on all road traffic accidents in the United Kingdom. The data that support the findings of this study are openly available at https://www.data.gov.uk/dataset/cb7ae6f0-4be6-4935-9277-47e5ce24a11f/road-safety-data.

Declarations

Ethical approval and consent to participate

This study was conducted in accordance with the Declaration of Helsinki and approved by the Joint Institutional Review Board of Taipei Medical University (N202011030).

Consent for publication

This study was approved by the Joint Institutional Review Board of Taipei Medical University (N202011030). The Joint Institutional Review Board of Taipei Medical University has waived the requirement of informed consent. All methods were performed in accordance with the relevant guidelines and regulations of the Declaration of Helsinki.

Competing interests

The authors declare that they have no competing interests in relation to this work.

Author information

[1]Graduate Institute of Injury Prevention and Control, College of Public Health, Taipei Medical University, Taipei City, Taiwan. [2]Department of Emergency Medicine, Taipei Medical University Hospital, Taipei City, Taiwan. [3]Department of Emergency Medicine, School of Medicine, College of Medicine, Taipei Medical University, Taipei City, Taiwan. [4]Department of Emergency Medicine, New Taipei City Hospital, New Taipei City, Taiwan. [5]College of Medicine, Chang Gung University, Taoyuan City, Taiwan. [6]Department of Emergency Medicine, Chang Gung Memorial Hospital, Linkou branch, Taoyuan, Taiwan.[7] Department of Emergency Medicine, Taipei Medical University-Shuang Ho Hospital, New Taipei City, Taiwan. [8]Taipei Neuroscience Institute, Taipei Medical University, Taipei City, Taiwan. [9]Division of Neurosurgery, Department of Surgery, Wan Fang Hospital, Taipei Medical University, Taipei City, Taiwan. [10]Department of Physical Medicine and Rehabilitation, Wan Fang Hospital, Taipei Medical University, Taipei City, Taiwan. [11]Transport Research Institute, Edinburgh Napier University, Edinburgh, Scotland. [12]Department of

Epidemiology, Biostatistics, and Population Health, Faculty of Medicine, Public Health and Nursing, Universitas Gadjah Mada, Yogyakarta City, Indonesia, [13]Department of Occupational Medicine, Chang Gung Memorial Hospital, Linkou branch, Taoyuan, Taiwan.

References:

1. Kjeldgard L, Ohlin M, Elrud R, Stigson H, Alexanderson K, Friberg E: Bicycle crashes and sickness absence - a population-based Swedish register study of all individuals of working ages. *BMC Public Health* 2019, *19*(1):943.
2. World Health Organization. Regional Office for E: Walking and cycling: latest evidence to support policy-making and practice. Copenhagen: World Health Organization. Regional Office for Europe; 2022,*1*.
3. Venkatraman V, Richard CM, Magee K, Johnson K: Countermeasures That Work: A Highway Safety Countermeasure Guide for State Highway Safety Offices, 10th Edition, 2020. 2021(DOT HS 813 097).
4. Allen-Munley C, Daniel J, Dhar S: Logistic Model for Rating Urban Bicycle Route Safety. *Transportation Research Record* 2004, 1878,*1*,:107-115.
5. Kaplan JA: Characteristics of the Regular Adult Bicycle User. Final Report; 1975.
6. Rivara FP, Thompson DC, Thompson RS: Epidemiology of bicycle injuries and risk factors for serious injury. *Inj Prev* 1997, 3*,2,*:110-114.
7. Wanvik PO: Effects of road lighting: an analysis based on Dutch accident statistics 1987-2006. *Accid Anal Prev* 2009, 41*,1,*:123-128.
8. Bicyclists and other cyclists: 2020 data (Traffic Safety Facts. Report No. DOT HS 813 322). In. https://crashstats.nhtsa.dot.gov/Api/Public/ViewPublication/813322: National Highway Traffic Safety Administration; 2022.
9. Bil M, Bilova M, Muller I: Critical factors in fatal collisions of adult cyclists with automobiles. *Accid Anal Prev* 2010, 42*,6,*:1632-1636.
10. Moore DN, Schneider WHt, Savolainen PT, Farzaneh M: Mixed logit analysis of bicyclist injury severity resulting from motor vehicle crashes at intersection and non-intersection locations. *Accid Anal Prev* 2011, 43*,3,*:621-630.
11. Wachtel A, Lewiston D: Risk factors for bicycle-motor vehicle collisions at intersections. *ITE journal* 1994, 64*,9,*:30-35.
12. Meuleners LB, Fraser M, Johnson M, Stevenson M, Rose G, Oxley J. Characteristics of the road infrastructure and injurious cyclist crashes resulting in a hospitalisation. *Accid Anal Prev.* 2020;136:105407.
13. Ugan J, Abdel-Aty M, Cai Q, Mahmoud N, Al-Omari Me: Effect of Various Speed Management Strategies on Bicycle Crashes for Urban Roads in Central Florida. *Transportation Research Record: Journal of the Transportation Research Board* 2021, 2676:036119812110366.
14. Robartes E, Chen TD: The effect of crash characteristics on cyclist injuries: An analysis of Virginia automobile-bicycle crash data. *Accid Anal Prev* 2017, 104*,1,*:165-173.
15. Pai CW: Overtaking, rear-end, and door crashes involving bicycles: an empirical investigation. *Accid Anal Prev* 2011, 43*,1,*:1228-1235.
16. Debnath AK, Haworth N, Schramm A, Heesch KC, Somoray K: Factors influencing noncompliance with bicycle passing distance laws. *Accid Anal Prev* 2018, 115*,1,*:137-142.
17. Maldonado G, Greenland S: Simulation study of confounder-selection strategies. *Am J Epidemiol* 1993, 138*,11,*:923-936.
18. Liu J, Jones S, Adanu EK, Li X: Behavioral pathways in bicycle-motor vehicle crashes: From

contributing factors, pre-crash actions, to injury severities. *J Safety Res* 2021, 77*,1,*:229-240.

19. Walker I: Signals are informative but slow down responses when drivers meet bicyclists at road junctions. *Accid Anal Prev* 2005, 37*,6,*:1074-1085.
20. Kay JJ, Savolainen PT, Gates TJ, Datta TK: Driver behavior during bicycle passing maneuvers in response to a Share the Road sign treatment. *Accid Anal Prev* 2014, *70*:92-99.
21. Anstey KJ, Horswill MS, Wood JM, Hatherly C: The role of cognitive and visual abilities as predictors in the Multifactorial Model of Driving Safety. *Accid Anal Prev* 2012, *45*:766-774.
22. Kandasamy D, Betz ME, DiGuiseppi C, Mielenz TJ, Eby DW, Molnar LJ, Hill L, Strogatz D, Li G: Self-reported health conditions and related driving reduction in older drivers. *Occup Ther Health Care* 2018, 32*,4,*:363-379.
23. Laosee O, Rattanapan C, Somrongthong R: Physical and cognitive functions affecting road traffic injuries among senior drivers. *Arch Gerontol Geriatr* 2018, *78*:160-164.
24. Cicchino JB, Wells JK, McCartt AT: Survey about pedestrian safety and attitudes toward automated traffic enforcement in Washington, D.C. *Traffic Inj Prev* 2014, 15(4):414-423.
25. Kostyniuk LP, Molnar LJ: Self-regulatory driving practices among older adults: health, age and sex effects. *Accid Anal Prev* 2008, 40*,4,*:1576-1580.
26. Zhang J, Lindsay J, Clarke K, Robbins G, Mao Y: Factors affecting the severity of motor vehicle traffic crashes involving elderly drivers in Ontario. *Accid Anal Prev* 2000, 32*,1,*:117-125.
27. Edwards JD, Ross LA, Wadley VG, Clay OJ, Crowe M, Roenker DL, Ball KK: The useful field of view test: normative data for older adults. *Arch Clin Neuropsychol* 2006, 21*,4,*:275-286.
28. Wood JM, Lacherez PF, Marszalek RP, King MJ: Drivers' and cyclists' experiences of sharing the road: incidents, attitudes and perceptions of visibility. *Accid Anal Prev* 2009, 41*,4,*:772-776.
29. W.~Hunter W, Stewart JR: An Evaluation of Bike Lanes Adjacent to Motor Vehicle Parking. 1999.
30. Huang C-Y: Observations of Drivers' Behavior when Opening Car Doors. *Procedia Manufacturing* 2015, *3*:2753-2760.
31. Traffic Safety Facts - Bicyclists and Other Cyclists. In. https://crashstats.nhtsa.dot.gov/Api/Public/ViewPublication/813322: National Highway Traffic Safety Administration; 2020.

8-5 Manuscript clean version
Click here to access/download;Manuscript;8-5 Clean_Manuscript for PLOS One copy.docx

Risk Factors for Overtaking, Rear-End, and Door Crashes Involving Bicycles in the United Kingdom: Revisited and Reanalysed

Chun-Chieh Chao[1,2,3†], Hon-Ping Ma[1,3,7], Li Wei[1,8,9], Yen-Nung Lin[1,10], Chenyi Chen[1], Wafaa Saleh[11], Bayu Satria Wiratama[12], Akhmad Fajri Widodo[1], Shou-Chien Hsu[6,13], Shih Yu Ko[7], Hui-An Lin[1,2,3†], Cheng-Wei Chan[1,4,5,6†], Chih-Wei Pai[1*]

[1] Graduate Institute of Injury Prevention and Control, College of Public Health, Taipei Medical University, Taipei City, Taiwan, [2] Department of Emergency Medicine, Taipei Medical University Hospital, Taipei City, Taiwan, [3] Department of Emergency Medicine, School of Medicine, College of Medicine, Taipei Medical University, Taipei, Taiwan, [4] Department of Emergency Medicine, New Taipei City Hospital, New Taipei City, Taiwan, [5] College of Medicine, Chang Gung University, Taoyuan City, Taiwan, [6] Department of Emergency Medicine, Chang Gung Memorial Hospital, Linkou branch, Taoyuan, Taiwan, [7] Department of Emergency Medicine, Taipei Medical University-Shuang Ho Hospital, New Taipei City, Taiwan, [8] Taipei Neuroscience Institute, Taipei Medical University, Taipei, Taiwan, [9] Division of Neurosurgery, Department of Surgery, Wan Fang Hospital, Taipei Medical University, Taipei, Taiwan, [10] Department of Physical Medicine and Rehabilitation, Wan Fang Hospital, Taipei Medical University, Taipei, Taiwan, [11] Transport Research Institute, Edinburgh Napier University, Edinburgh, Scotland, [12] Department of Epidemiology, Biostatistics, and Population Health, Faculty of Medicine, Public Health and Nursing, Universitas Gadjah Mada, Yogyakarta City, Indonesia, [13] Department of Occupational Medicine, Chang Gung Memorial Hospital, Linkou branch, Taoyuan, Taiwan.

* Correspondence: cpai@tmu.edu.tw

† Contributed equally to this work

Abstract

Background: Relevant research has provided valuable insights into risk factors for bicycle crashes at intersections. However, few studies have focused explicitly on three common types of bicycle crashes on road segments: overtaking, rear-end, and door crashes.

Objective: This study aims to identify risk factors for overtaking, rear-end, and door crashes that occur on road segment.

Material and methods

We analysed British STATS19 accident records from 1991 to 2020. Using multivariate logistic regression models, we estimated adjusted odds ratios (AORs) with 95% confidence intervals (CIs) for multiple risk factors. The analysis included 127,637 bicycle crashes, categorised into 18,350 overtaking, 44,962 rear-end, 6,363 door, and 57,962 other crashes.

Results

Significant risk factors for overtaking crashes included speed limits of ≥40 miles per hour (mph) (AOR = 2.238, 95% CI = 2.159–2.320), heavy goods vehicles (HGVs) as crash partners (AOR = 2.867, 95% CI 2.473–3.323), and elderly crash partners (AOR = 2.013, 95% CI = 1.937–2.092). For rear-end crashes, noteworthy risk factors included unlit darkness (AOR = 1.486, 95% CI = 1.404–1.573) and midnight hours (AOR = 1.269, 95% CI = 1.190–1.354). Factors associated with door crashes included speed limits of 20–30 mph (AOR = 16.185, 95% CI = 13.514–19.382) and taxi and private hire cars (AOR = 2.695, 95% CI = 2.310–3.145). Our joint-effect analysis revealed additional interesting results; for example, there were elevated risks for overtaking crashes in rural areas with elderly drivers as crash partners (AOR = 2.93, 95% CI = 2.79–3.08) and with HGVs as crash partners (AOR = 2.62, 95% CI = 2.46–2.78).

Conclusions

The aforementioned risk factors remained largely unchanged since 2011, when we conducted our previous study. However, the present study concluded that the detrimental effects of certain variables became more pronounced in certain situations. For example, cyclists in rural settings exhibited an elevated risk of overtaking crashes involving HGVs as crash partners.

Keywords: Bicycle crash; Road segment; Overtaking crash; Rear-end crash; Door crash

Introduction

In recent years, urban bicycling has become increasingly popular in many countries, offering benefits such as reduced traffic congestion, diminished parking pressure, and a reduction in greenhouse gas emissions [1, 2]. The World Health Organization has highlighted numerous health advantages of moderate-intensity physical activities such as bicycling, including improvements in life expectancy, quality of life, cognitive function, mental health, sleep quality, muscular and cardiorespiratory fitness, and bone and functional health [2].

However, despite such health benefits, the risk of injury remains a considerable safety concern for cyclists, who are regarded as vulnerable road users [2, 3]. Traffic crash data indicate that the risk of accidents for cyclists, measured per distance travelled, is approximately 20 times higher than that for vehicle drivers[2]. To address this problem, researchers in the United States developed a comprehensive bicycle route safety rating model with a focus on injury severity [4].

This model evaluates multiple operational and physical aspects such as traffic volume, population density, highway classification, lane width, and the presence of one-way streets. In addition, it is capable of predicting the severity of injuries due to motor vehicle–related crashes at specific locations [4]. Another finding was that a route is considered adequately safe if it includes geometric factors that enhance safety [4]. This model can aid urban planners and public officials in creating infrastructure such as bike lanes and implementing strict lane policies to improve cyclist safety [4]. Implementing bike lanes has been demonstrated to reduce crash rates by up to 40% among adult cyclists [5]. One study regarding roundabouts indicated that roundabouts with cycle tracks significantly reduced injury risk for cyclists compared with those lacking bicycle infrastructure [6]. Furthermore, adequate night-time lighting on rural roads has the potential to prevent over half of all cyclist injuries [7]. Bicycle crashes can also impose a significant burden on healthcare expenses. Elvik and Sundfør [8] have discussed the economic implications and healthcare expenditures associated with bicycle accidents. For instance, in Belgium, the average cost of bicycle accidents per case is estimated at 841 euros [9]. In the Netherlands, the total annual cost has been reported as €410.7 million [10].

Although intersectional crashes are generally more frequent than nonintersectional ones, in 2020, 64% of fatal crashes involving cyclists occurred on road segments, defined as areas 20 m away from intersections, whereas only 26% of such fatalities occurred at intersections [11]. Bil et al. demonstrated that car drivers, when at fault for crashes, often cause more serious consequences for cyclists on straight road sections [12]. In crashes occurring on road segments, several factors contribute to high injury severity, including being in a rural region with an elevated speed limit, male gender, and cyclist age of >55 years [13]. Another identified risk factor is

bicycling on roads against oncoming traffic [14].

Although relevant research has shed light on risk factors for bicycle crashes at intersections, few studies have explicitly investigated crashes on road segments. Bicycle crashes on road segments remain a substantial issue for public health concern. This study aims to fill a critical gap by conducting a thorough examination of the risk factors associated with three distinct bicycle crash types: overtaking, rear-end, and door crashes that occur on road segments. Studies that have examined bicycle crashes relatively broadly, without distinguishing crash types, have identified several key factors—including vehicle volume [15], traffic density [16], number of lanes [16], access points along road segments [15], shoulder and median widths [15], parking space availability [15, 16] , length of continuous two-way left-turn lanes [15], and pavement type [17]—all of which contribute to bicycle crashes on road segments. Two exceptional work have examined risk factors for overtaking, rear-end, and door crashes [18, 19]. Specifically, Pai identified buses and coaches as common crash partners in overtaking crashes, poor visibility, traversing manoeuvres, and teenage cyclists as risk factors for rear-end crashes, and built-up areas as a risk factor for door crashes [18]. In addition, another study linked the speed of a passing vehicle to increased severity of cyclist injury in overtaking crashes [19].

The primary objective of the present study, an extension of our previous study [18], was to analyse police-reported crash data from additional years to determine whether the risk factors for these three crash types remained unchanged. The study addresses a critical gap in current research, focusing on crashes specifically occurring on road segments. Existing literature offers limited insights into these crash types, highlighting a crucial need for targeted investigations. These crashes have the potential for severe impacts, involving complex dynamics that demand a

nuanced understanding for effective mitigation strategies. By exploring these factors, our research aims to significantly enhance cyclist safety within this particular context. Furthermore, we aimed to untangle the joint associations of several factors—including light conditions, urban versus rural settings, vehicle types, and rider and driver characteristics—with these three crash types.

Material and Methods

Crash data source

The present investigation utilised data from 01/01/1991 to 31/12/2020, obtained from the United Kingdom's official road traffic casualty database, STATS19. Police record such data either at crash scenes or within 30 days of each crash. The UK's Department for Transport compiles the data, which the United Kingdom Data Archive then maintains and distributes. The dataset encompasses a variety of variables, including crash circumstances (e.g., time and date, weather conditions, road and light conditions, posted speed limit, road type), vehicle and driver characteristics, demographic details of the drivers, precrash manoeuvres of the vehicles, and the initial impact point of the vehicle. Additionally, the dataset contains demographic information and details regarding injury severity for each casualty. This study adhered to the STROBE (strengthening the reporting of observational studies in epidemiology) reporting guidelines.[20]

Injury severity in the aforementioned dataset is divided into three categories, namely slight, serious, and fatal. Fatal injuries refer to those leading to death within 30 days of the accident. Serious injuries include conditions such as fractures, internal injuries, severe cuts and lacerations, concussions, and any injury requiring hospitalisation. Slight injuries include sprains, bruises, and

minor cuts, as well as mild shock requiring roadside attention. The exclusive focus of this study was crashes leading to cyclist casualties.

As shown in Figure 1, this study analysed 1,366,196 crashes involving bicycles and other vehicles. Initially, 1,235,032 junction cases were excluded. From the remaining 131,164 bicycle segment crashes, 3,527 were further excluded because of incomplete demographic data for the cyclist and missing speed limit information, leaving a valid cohort of 127,637 bicycle segment crashes for analysis. Within this cohort, this study identified 18,350 overtaking crashes, 44,962 rear-end crashes, 6,363 door crashes, and 57,962 other types of crashes.

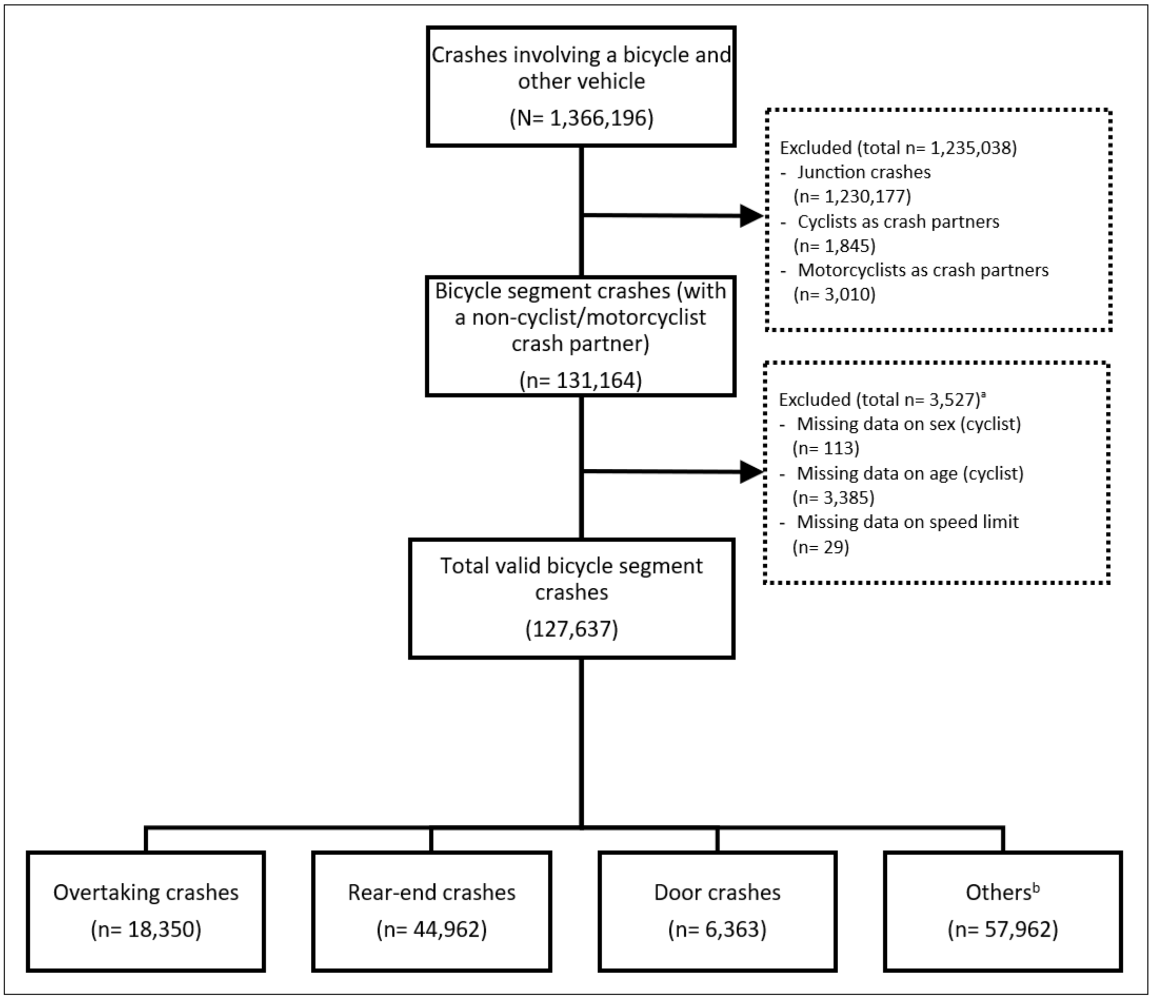

Figure. 1. Flowchart of the study sample selection process. [a]Listed excluded criteria are nonexclusive; thus, the sum of the total may exceed 3,527. [b]Other crashes include reversing crashes and head-on crashes.

Classification of crash types

As shown in Figure 2, an overtaking crash is defined as a crash where a motorised vehicle overtakes and impacts with a bicycle, which may be travelling straight, overtaking another vehicle, changing lanes, or turning. A rear-end crash occurs when a following vehicle impacts with the rear of a bicycle. A door crash involves a bicycle either being struck by or striking the opening door of an automobile. These three crash types were described using schematics in our previous study [18].

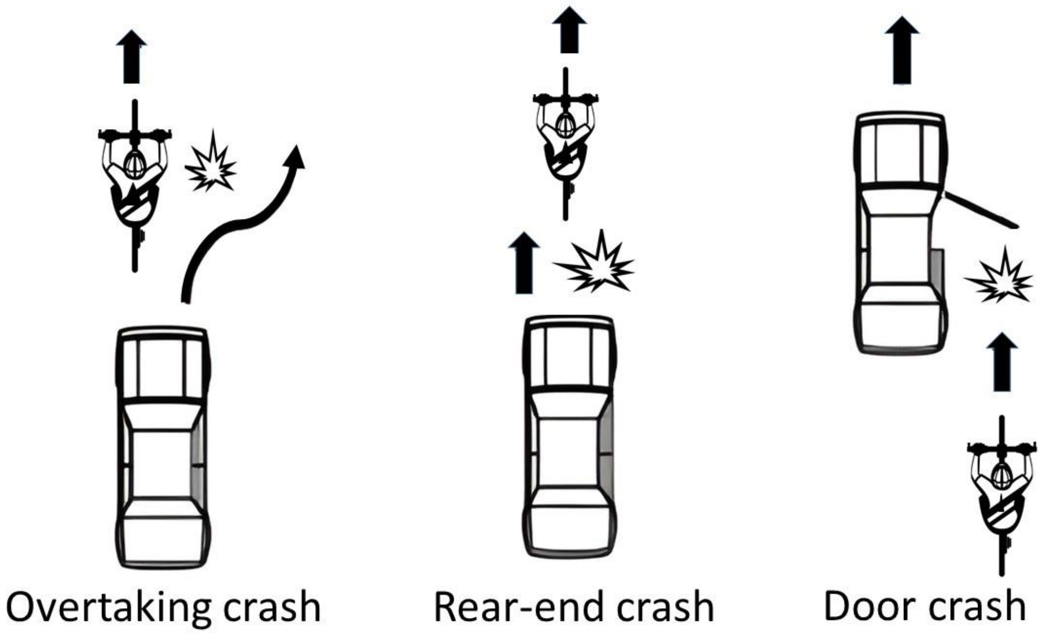

Figure 2. Illustrative diagram of the three crash types

Data collection

For the present study, the three crash types of focus (overtaking, rear-end, and door crashes) were the binary-dependent variables. The collected data encompassed the following factors: lighting conditions on the roadway at the time of the crash (daylight, darkness-lit, darkness-unlit),

the speed limit at the crash scene (rural: ≥40 miles per hour [mph]; urban: 20–30 mph), the time of day categorised into four periods according to traffic volume (midnight: 00:00–06:00; rush hours: 07:00–08:00 and 17:00–18:00; nonrush hours: 09:00–16:00; and evening: 19:00–23:00), and the day of the week (weekday or weekend day). The demographic details of cyclist casualties encompassed age (≤18, 19–40, 41–64, or ≥65 years) and sex (male or female). Finally, the demographic details of the crash partner included the type of vehicle (identified as a taxi, private hire car, car, bus, or heavy goods vehicle [HGV]), age (≤18, 19–40, 41–64, or ≥65 years) and sex (male or female). On a cautionary note, we removed junction cases to avoid the variability introduced when exogenous factors, such as junction geometry and control measures, are present at junctions. Furthermore, the cases involving other cyclists and motorcyclists were removed as we focused on vehicle-cycle crashes only. Missing data on sex, age, or speed limits were also excluded in the analysis. Excluding these data may impact our results in a marginal scale, as these data are likely to be single-bicycle crashes that in nature be underreported in police crash dataset [21].

Statistical analysis

This study employed the chi-squared test to examine the associations between crash type and other factors, including cyclist or motorist characteristics, vehicle features, roadway conditions, and temporal variables. Initially, we examined the distribution of three crash types across various variables to explore their relationships with a binary outcome. These variables included lighting conditions, speed limit, time of day, and day of the week. Demographic details concerning cyclist casualties encompassed age and sex, while information about the crash partner included vehicle

type, age, and sex. We set a significance level of $p < 0.2$ to include risk factors in our multivariate analysis [23]. Adjusted odds ratios (AORs) were computed using multivariate logistic regression with backward selection.[22, 23]

The multivariate logistic regression model equation was specified as:

$$log\left(\frac{P(Y=1)}{1 - P(Y=1)}\right) = \beta_0 + \beta_1 X_1 + \beta_2 X_2$$

where $P(Y = 1)$ denotes the probability of the outcome, $\beta_0, \beta_1, \beta_2, \ldots, \beta_p$ are the coefficients to be estimated, and $X_1, X_2, \ldots, X_p$ represent the predictor variables.

Before estimating the model, assumptions of logistic regression, such as linearity of the logit, absence of multicollinearity, and independence of observations, were evaluated. An odds ratio (OR) greater than 1 indicated a positive association between the independent variable and the occurrence rate, while an OR less than 1 indicated a negative association. An OR of 1 suggested no association between the variables of interest and the outcomes. All statistical analyses were conducted using SPSS Statistics version 25 for Windows (IBM Corp., Armonk, New York, USA). A *p* value lower than 0.05 in two-tailed tests was considered statistically significant.

Results

Population characteristics

Tables 1, 2, and 3 present the distributions of overtaking, rear-end, and door crashes, respectively, in relation to multiple independent variables. These data revealed that a significant proportion

of bicycle crashes occurred in daylight (82.3%), occurred in urban settings (78.5%), occurred during nonrush hours (48.3%), occurred on weekdays (77.5%), involved cyclists aged under 18 years (40.1%), and involved male cyclists (81.3%). Additionally, most crashes involved cars as crash partners (83.6%), and crash partners were predominately aged 19–40 years (38.5%) and were male (76.4%). Table 1 highlights an overrepresentation in bicycle overtaking crashes for certain variables, namely unlit darkness (19.5%), rural areas (24.8%), midnight hours (17.7%), buses or HGVs as crash partners (24.7%), and elderly crash partners (21.5%) and male crash partners (16.0%). These results were revealed to be statistically significant by the chi-squared test ($p < 0.01$).

Table 1. Distribution of overtaking crashes according to a set of independent variables

Variable	Total (n=127,637)	Overtaking crashes (n=18,350)	Non-overtaking crashes (n=109,287)	*χ2 test p value*
**Light conditions**, n (%)				<0.001
Daylight	105,053 (82.3%)	15,283 (14.55%)	89,770 (85.5%)	
Darkness-lit	16,543 (13.0%)	1,889 (11,42%)	14,654 (88.6%)	
Darkness-unlit	6,041 (4.7%)	1,178 (19.50%)	4,863 (80.5%)	
**Speed limit**, n (%)				<0.001
Rural (≥ 40 mph)	27,395 (21.5%)	6,805 (24.8%)	20,590 (75.6%)	
Urban (20–30 mph)	100,242 (78.5%)	11,545 (11.5%)	88,697 (88.5%)	
**Crash time (h)**, n (%)				<0.001
Midnight (00:00–06:00)	4,810 (3.8%)	852 (17.7%)	3,958 (82.3%)	
Rush hours (07:00–08:00/17:00–18:00)	41,619 (32.6%)	5,685 (13.7%)	35,934 (86.3%)	
Nonrush hours (09:00–16:00)	61,696 (48.3%)	9,386 (15.2%)	52,310 (84.8%)	
Evening (19:00–23:00)	19,512 (15.3%)	2,427 (12.4%)	17,085 (87.6%)	
**Crash day**, n (%)				0.094
Weekend	28,730 (22.5%)	4,218 (14.7%)	24,512 (85.2%)	
Weekday	98,907 (77.5%)	14,132 (14.3%)	84,775 (85.7%)	
**Cyclist's age (years)**, n (%)				<0.001
≤18	51,193 (40.1%)	5,220 (10.2%)	45,973 (89.8%)	
19–40	45,760 (35.9%)	7,108 (15.5%)	38,652 (84.5%)	
41–64	26,052 (20.4%)	5,012 (19.2%)	21,040 (80.8%)	
≥65	4,632 (3.6%)	1,010 (21.8%)	3,622 (78.2%)	
**Cyclist's sex**, n (%)				<0.001
Male	103,766 (81.3%)	14,746 (14.2%)	89,020 (85.8%)	
Female	23,871 (18.7%)	3,604 (15.1%)	20,267 (84.9%)	
**Crash partner**, n (%)				<0.001

Taxi/Private hire car	2,588 (2.0%)	208 (8.0%)	2,380 (92.0%)	
Car	106,668 (83.6%)	13,599 (12.8%)	93,069 (87.3%)	
Bus/Heavy goods vehicle	18,381 (14.4%)	4,543 (24.7%)	13,838 (75.3%)	
**Crash partner's age (years)**, n (%)				<0.001
≤18	2,415 (1.9%)	281 (11.6%)	2,134 (88.4%)	
19–40	49,103 (38.5%)	5,398 (11.0%)	43,705 (89.0%)	
41–64	35,598 (27.9%)	3,973 (11.2%)	31,625 (88.8%)	
≥65	40,521 (31.8%)	8,698 (21.5%)	31,823 (78.5%)	
**Crash partner's sex**, n (%)				<0.001
Male	97,447 (76.4%)	15,584 (16.0%)	81,863 (84.0%)	
Female	30,190 (23.8%)	2,766 (9.2%)	27,424 (90.8%)	

Several variables in Table 2 reveal significant differences between rear-end crashes and non-rear-end crashes. Specifically, a higher proportion of rear-end crashes occurred under darkness-unlit conditions (50.2%) compared to darkness-lit conditions (37.5%). Additionally, rear-end crashes were more prevalent in rural areas with speed limits of ≥ 40 mph (43.0%) compared to urban areas with speed limits of 20–30 mph (33.1%). Crashes involving crash partners aged ≥ 65 accounted for 39.7% of rear-end crashes, which was higher compared to other age groups (age 41–64: 33.0% and ≤18: 36.0%). Furthermore, rear-end crashes were more likely to occur during midnight (47.6%) compared to rush hours (36.3%). Taxis were frequently involved in rear-end crashes (42.4%), as were male crash partners (36.8%). These findings highlight the significant influence of various factors on the likelihood of rear-end crashes. Variables such as darkness-unlit conditions, higher speed limits in rural areas, crash time, and characteristics of the crash partner all emerged as significant determinants. Specifically, rear-end crashes were notably more prevalent under darkness-unlit conditions, in rural areas with higher speed limits, during midnight hours, and involving certain characteristics of crash partners. Importantly, these associations were statistically significant, as indicated by the Chi-squared test ($p < 0.001$).

Table 2. Distribution of rear-end crashes according to a set of independent variables

Variable	Total (n=127,637)	Rear-end crashes (n=44,962)	Non-rear-end crashes (n=82,675)	*χ2 test p value*
**Light conditions**, n (%)				<0.001
Daylight	105,053 (82.3%)	35,726 (34.1%)	69,333 (66.0%)	
Darkness-lit	16,543 (13.0%)	6,204 (37.5%)	10,339 (63.5%)	
Darkness-unlit	6,041 (4.73%)	3,032 (50.19%)	3,003 (49.71%)	
**Speed limit**, n (%)				<0.001
Rural (≥ 40 mph)	27,395 (21.5%)	11,788 (43.0%)	15,607 (57.0%)	
Urban (20–30 mph)	100,242 (78.5%)	33,174 (33.1%)	67,068 (66.9%)	
**Crash time (h)**, n (%)				<0.001
Midnight (00:00–06:00)	4,810 (3.8%)	2,289 (47.6%)	2,521 (52.4%)	
Rush hours (07:00–08:00/17:00–18:00)	41,619 (32.6%)	15,089 (36.3%)	26,530 (63.7%)	
Nonrush hours (09:00–16:00)	61,696 (48.3%)	20,723 (33.6%)	40,973 (66.4%)	
Evening (19:00–23:00)	19,512 (15.3%)	6,861 (36.2%)	12,651 (64.9%)	
**Crash day**, n (%)				<0.001
Weekend	28,730 (22.5%)	9,485 (33.0%)	19,245 (67.0%)	

Weekday	98,907 (77.5%)	35,477 (35.9%)	63,430 (64.1%)	
**Cyclist's age (years)**, n (%)				<0.001
≤18	51,193 (40.1%)	13,446 (26.3%)	37,747 (73.7%)	
19–40	45,760 (35.9%)	19,102 (41.7%)	26,658 (58.3%)	
41–64	26,052 (20.4%)	10,619 (40.8%)	15,433 (59.2%)	
≥65	4,632 (3.6%)	1,795 (38.8%)	2,837 (61.3%)	
**Cyclist's sex**, n (%)				<0.001
Male	103,766 (81.3%)	37,175 (35.8%)	66,591 (64.2%)	
Female	23,871 (18.7%)	7,787 (32.6%)	16,084 (67.4%)	
**Crash partner**, n (%)				<0.001
Taxi/Private hire car	2,588 (2.0%)	1,096 (42.4%)	1,492 (57.7%)	
Car	106,668 (83.6%)	37,202 (34.9%)	71,342 (66.9%)	
Bus/Heavy goods vehicle	18,381 (14.4%)	6,664 (36.3%)	9,841 (53.5%)	
**Crash partner's age (years)**, n (%)				<0.001
≤18	2,415 (1.9%)	870 (36.0%)	1,545 (64.0%)	
19–40	49,103 (38.5%)	16,282 (33.2%)	32,821 (66.8%)	
41–64	35,598 (27.9%)	11,736 (33.0%)	23,862 (67.0%)	
≥65	40,521 (31.8%)	16,074 (40.0%)	24,447 (60.3%)	
**Crash partner's sex**, n (%)				<0.001
Male	97,447 (76.6%)	35,828 (36.8%)	61,619 (63.2%)	
Female	30,190 (23.7%)	9,134 (30.3%)	21,056 (69.7%)	

As shown in Table 3, several variables can contribute to door crashes involving bicycles. Door crashes predominantly occurred in urban areas with speed limits of 20-30 mph (6.2%), while a significantly lower proportion occurred in rural areas with speed limits ≥ 40 mph (0.5%). These crashes were overrepresented during non-rush hours (5.5%) and rush hours (4.9%) compared to evening (4.3%) and midnight (2.4%). Cyclists were more frequently involved in door crashes on weekdays (5.4%) than weekends (3.7%). As many as 8.2% of all female cyclists were involved in door crashes, which is higher than the involvement rate among males (4.2%). Taxi and private hire cars were overinvolved in door crashes (10.6%) compared to cars (5.2%) and buses/heavy goods vehicles (3.1%). Crash partners aged ≤18 years (5.2%) and 19-40 years (5.3%) were disproportionately involved in door crashes compared to older age groups, and female crash partners were overrepresented in door crashes (7.4%) compared to males (4.2%). These results were statistically significant, as indicated by the Chi-squared test ($p < 0.001$). They suggest that various factors—including traffic conditions (rural areas, crash time), cyclist demographics

(younger age, gender), and characteristics of the crash partner (taxi/private hire cars)—significantly contribute to the likelihood of door crashes involving cyclists.

Table 3. Distribution of door crashes according to a set of independent variables

Variable	Total (n=127,637)	Door crashes (n=6,363)	Non-door crashes (n=121,274)	*χ2 test p value*
**Light conditions**, n (%)				<0.001
Daylight	105,053 (82.3%)	5,192 (4.9%)	99,861 (95.1%)	
Darkness-lit	16,543 (13.0%)	1,031 (6.2%)	15,512 (93.8%)	
Darkness-unlit	6,041 (4.7%)	140 (2.3%)	5,901 (97.7%)	
**Speed limit**, n (%)				<0.001
Rural (≥ 40 mph)	27,395 (21.5%)	123 (0.5%)	27,272 (99.6%)	
Urban (20–30 mph)	100,242 (78.5%)	6,240 (6.2%)	94,002 (93.8%)	
**Crash time (h)**, n (%)				<0.001
Midnight (00:00–06:00)	4,810 (3.8%)	113 (2.4%)	4,697 (97.7%)	
Rush hours (07:00–08:00/17:00–18:00)	41,619 (32.6%)	2,056 (4.9%)	39,563 (95.1%)	
Nonrush hours (09:00–16:00)	61,696 (48.3%)	3,363 (5.5%)	58,333 (94.6%)	
Evening (19:00–23:00)	19,512 (15.3%)	831 (4.3%)	18,681 (95.7%)	
**Crash day**, n (%)				<0.001
Weekend	28,730 (22.5%)	1,072 (3.7%)	27,658 (96.3%)	
Weekday	98,907 (77.5%)	5,291 (5.4%)	93,616 (94.7%)	
**Cyclist's age (years)**, n (%)				<0.001
≤18	51,193 (40.1%)	802 (1.6%)	50,391 (98.4%)	
19–40	45,760 (35.9%)	3,474 (7.6%)	42,286 (93.4%)	
41–64	26,052 (20.4%)	1,773 (6.8%)	24,279 (93.2%)	
≥65	4,632 (3.6%)	314 (6.8%)	4,318 (93.2%)	
**Cyclist's sex**, n (%)				<0.001
Male	103,766 (81.3%)	4,404 (4.2%)	99,362 (95.8%)	
Female	23,871 (18.7%)	1,959 (8.2%)	21,912 (91.8%)	
**Crash partner**, n (%)				<0.001
Taxi/Private hire car	2,588 (2.0%)	273 (10.6%)	2,315 (89.5%)	
Car	106,668 (83.6%)	5,514 (5.2%)	101,154 (94.8%)	
Bus/Heavy goods vehicle	18,381 (14.4%)	576 (3.1%)	17,805 (96.9%)	
**Crash partner's age (years)**, n (%)				<0.001
≤18	2,415 (1.9%)	1,62 (5.2%)	2,253 (93.3%)	
19–40	49,103 (38.5%)	2,585 (5.3%)	46,518 (94.7%)	
41–64	35,598 (27.9%)	1,887 (5.3%)	33,711 (94.7%)	
≥65	40,521 (31.8%)	1,729 (4.3%)	38,792 (95.7%)	
**Crash partner's sex**, n (%)				<0.001
Male	97,447 (76.6%)	4,123 (4.2%)	93,324 (95.8%)	
Female	30,190 (23.7%)	2,240 (7.4%)	27,950 (92.6%)	

Risk factors for the three crash types

Table 4 presents the logistic regression model results. Regarding overtaking crashes, the identified risk factors included daylight conditions (adjusted odds ratio [AOR] = 1.233, 95%

confidence interval [CI] = 1.162–1.309; *p*<0.001), speed limits of ≥40 mph (AOR = 2.238, 95% CI = 2.159–2.320; *p*<0.001), nonrush hours (AOR = 1.091, 95% CI 1.031–1.154; *p*=0.003), cyclists aged ≥65 years (AOR = 1.785, 95% CI = 1.649–1.931; *p*<0.001), female cyclists (AOR = 1.106, 95% CI = 1.062–1.153), HGVs as crash partners (AOR = 2.867, 95% CI = 2.473–3.323; *p*<0.001), elderly crash partners (AOR = 2.013, 95% CI = 1.937–2.092; *p*<0.001), and male crash partners (AOR = 1.353, 95% CI = 1.292–1.416; *p*<0.001).

For rear-end crashes, noteworthy risk factors included unlit darkness (AOR = 1.486, 95% CI = 1.404–1.573; *p*<0.001), speed limits of ≥40 mph (AOR = 1.315, 95% CI = 1.277–1.354; *p*<0.001), weekdays (AOR = 1.090, 95% CI = 1.059–1.122; *p*<0.001), midnight hours (AOR = 1.269, 95% CI = 1.190–1.354; *p*<0.001), and taxis as crash partners (AOR = 1.286, 95% CI = 1.186–1.394; *p*<0.001).

Regarding door crashes, significant risk factors included lit darkness (AOR = 1.373, 95% CI = 1.141–1.651; *p*<0.001), speed limits of 20–30 mph (AOR = 16.185, 95% CI = 13.514–19.382; *p*<0.001), weekdays (AOR = 1.246, 95% CI = 1.162–1.336; *p*<0.001), and nonrush hours (AOR = 2.912, 95% CI = 2.384–3.556; *p*<0.001). Additionally, female cyclists (AOR = 1.675, 95% CI = 1.582–1.774; *p*<0.001), taxis or private hire cars as crash partners (AOR = 2.695, 95% CI = 2.310–3.145; *p*<0.001), male crash partners (AOR = 1.373, 95% CI = 1.296–1.455; *p*<0.001), and crash partners aged 41–64 years (AOR = 1.855, 95% CI = 1.625–2.117; *p*<0.001) were associated with door crashes.

Table 4. Multivariate logistic regression results

Variable	Overtaking crashes		Rear-end crashes		Door crashes	
	AOR (95% CI)	*p* value	AOR (95% CI)	*p* value	AOR (95% CI)	*p* value
**Light condition**						
Daylight	1.233 (1.162, 1.309)	<0.001	Ref		1.146 (0.958, 1.370)	0.137
Darkness-lit	Ref		1.042 (1.002, 1.085)	0.041	1.373 (1.141, 1.651)	0.001
Darkness-unlit	1.152 (1.059, 1.253)	0.001	1.486 (1.404, 1.573)	<0.001	Ref	
**Speed limit**						
Rural (≥40 mph)	2.238 (2.159, 2.320)	<0.001	1.315 (1.277, 1.354)	<0.001	Ref	
Urban (20–30 mph)	Ref		Ref		16.185 (13.514, 19.382)	<0.001
**Crash time**						
Midnight	1.073 (0.982, 1.173)	0.119	1.269 (1.190, 1.354)	<0.001	Ref	
Rush hours	1.059 (1.002, 1.120)	0.043	1.108 (1.078, 1.139)	<0.001	2.502 (2.051, 3.052)	<0.001
Nonrush hours	1.091 (1.031, 1.154)	0.003	Ref		2.912 (2.384, 3.556)	<0.001
Evening	Ref		0.992 (0.953, 1.032)	0.686	2.014 (1.646, 2.465)	<0.001

Table 4. Multivariate logistic regression results *(continued)*

Variable	Overtaking crashes		Rear-end crashes		Door crashes	
	AOR (95% CI)	*p* value	AOR (95% CI)	*p* value	AOR (95% CI)	*p* value
**Crash day**						
Weekend	1.031 (0.991, 1.072)	0.132	Ref		Ref	
Weekday	Ref		1.090 (1.059, 1.122)	<0.001	1.246 (1.162, 1.336)	<0.001
**Cyclist's age (years)**						
≤18	Ref		Ref		Ref	
19–40	1.292 (1.242, 1.345)	<0.001	1.839 (1.788, 1.891)	<0.001	5.943 (5.489, 6.435)	<0.001
41–64	1.509 (1.444, 1.578)	<0.001	1.731 (1.676, 1.789)	<0.001	6.129 (5.621, 6.684)	<0.001
≥65	1.785 (1.649, 1.931)	<0.001	1.671 (1.568, 1.780)	<0.001	5.988 (5.217, 6.874)	<0.001
**Cyclist's sex**						
Male	Ref		1.172 (1.137, 1.208)	<0.001	Ref	
Female	1.106 (1.062, 1.153)	<0.001	Ref		1.675 (1.582, 1.774)	<0.001
**Crash partner**						
Taxi/Private hire car	Ref		1.286 (1.186, 1.394)	<0.001	2.695 (2.310, 3.145)	<0.001
Car	1.571 (1.359, 1.816)	<0.001	Ref		2.089 (1.908, 2.286)	<0.001
Bus/Heavy goods vehicle	2.867 (2.473, 3.323)	<0.001	1.099 (1.061, 1.139)	<0.001	Ref	
**Crash partner's age (years)**						
≤18	1.097 (0.963, 1.249)	0.162	1.225 (1.188, 1.263)	<0.001	1.507 (1.313, 1.731)	<0.001
19–40	Ref		1.038 (1.008, 1.069)	0.013	1.855 (1.625, 2.117)	<0.001
41–64	0.950 (0.909, 0.994)	0.025	Ref		1.801 (1.574, 2.060)	<0.001
≥65	2.013 (1.937, 2.092)	<0.001	1.241 (1.137, 1.355)	<0.001	Ref	
**Crash partner's sex**						
Male	1.353 (1.292, 1.416)	<0.001	1.150 (1.117, 1.185)	<0.001	1.373 (1.296, 1.455)	<0.001
Female	Ref		Ref		Ref	

Figure 2 presents a forest plot demonstrating the joint effects of several variables on the three crash types when other variables were controlled for. The results identified several key risk

factors for both overtaking and rear-end crashes. The risk of overtaking crashes showed a significant increase of 193% in rural areas when elderly drivers were involved (AOR = 2.93, 95% CI = 2.79–3.08), and similarly when heavy goods vehicles (HGVs) were the crash partner (AOR = 2.62, 95% CI = 2.46–2.78). Elderly cyclists also faced a higher risk of overtaking crashes on weekends (AOR = 1.56, 95% CI = 1.34–1.81).

Regarding rear-end crashes, the risk increased notably with unlit darkness during midnight (AOR = 1.68, 95% CI = 1.48–1.90) and was significantly higher in rural areas (AOR = 2.15, 95% CI = 2.01–2.31). Furthermore, bicycling at midnight in rural areas was associated with an increased risk of rear-end crashes (AOR = 1.68; 95% CI = 1.51–1.86). In urban settings, the risk of door crashes was higher for female cyclists (AOR = 2.29; 95% CI = 2.17–2.43) and for elderly cyclists (AOR = 2.06; 95% CI = 1.82–2.34). Finally, female cyclists exhibited a 112% higher likelihood of door crashes when the crash partner was a taxi (AOR = 2.12; 95% CI = 1.68–2.69).

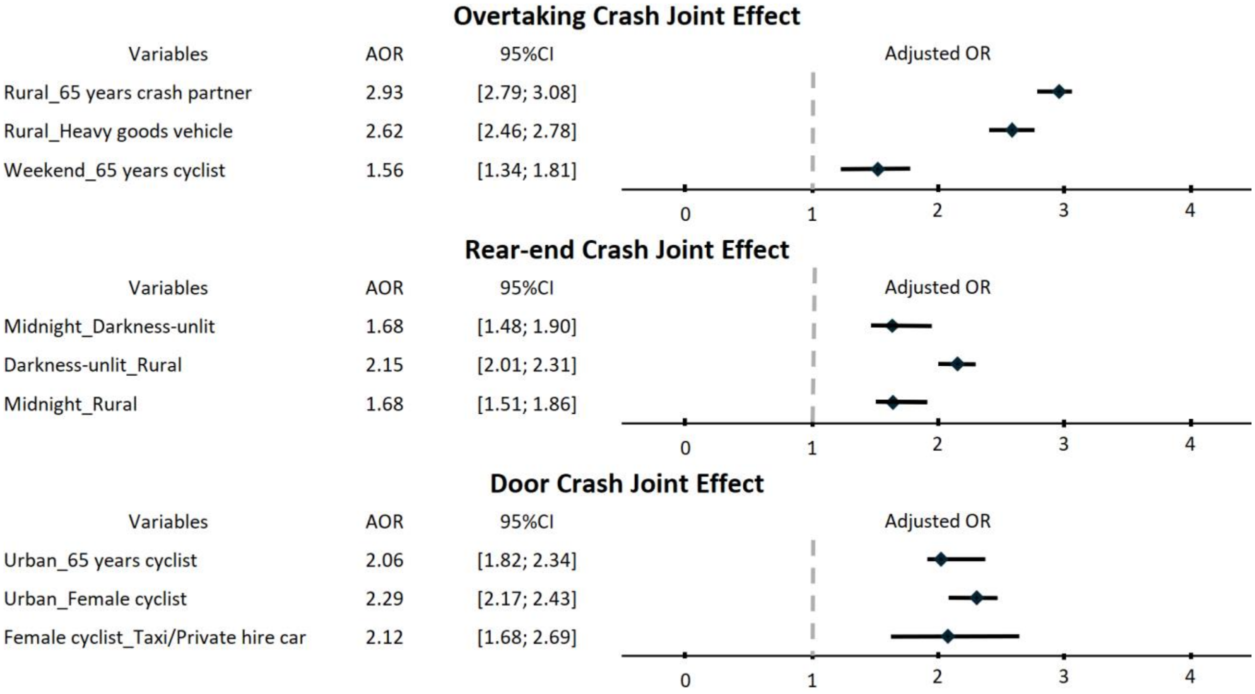

Figure. 3. Joint effects of several variables on the three crash types.

Discussion

This study explored the relationships among individual and environmental factors in relation to three common bicycle crash types (overtaking, rear-end, and door crashes) on roads in the United Kingdom from 1991 to 2020. The findings revealed several significant factors. First, for overtaking crashes, HGVs as crash partners, rural areas, and the involvement of elderly crash partners emerged as key contributing factors. Second, unlit darkness, midnight hours, and rural areas were the factors most closely associated with rear-end crashes. Third, urban areas and taxis as crash partners significantly increased the likelihood of door crashes. Moreover, male crash partners were found to be a consistent risk factor across all three crash types.

Our research findings identified specific risk factors for overtaking crashes, namely rural areas, HGVs as crash partners, and elderly crash partners. These findings align with previous research that identified elderly drivers [24], speeds exceeding 10 mph, and the presence of pick-up trucks as factors contributing to increased risk for overtaking crash. Specifically, HGVs possess several characteristics that amplify this danger. Their large blind spots make it difficult for drivers to see cyclists, increasing the likelihood of crashes during overtaking. Additionally, HGVs are less manoeuvrable compared to passenger cars, which reduces their ability to avoid crashes if cyclists suddenly enter their path. The speed and distance perception issues between HGVs and cyclists further complicate the judgment of safe overtaking gaps. Furthermore, HGVs require longer stopping distances due to their size and weight, which can lead to severe consequences if a sudden need to brake arises. A behavioural study suggested that compared with cars, HGVs

tended to maintain a narrower clearance zone when overtaking bicycles [25]. Regarding the association with buses or HGVs, Pai et al. [18] suggested that time pressures on HGV drivers for timely loading and unloading might lead to more reckless driving. Specifically, our results align with the observations made by Pai et al., who also mentioned higher crash rates involving buses or HGVs, supporting the idea that these time pressures contribute to increased crash risks. Our findings underscore the necessity of implementing measures such as ‘Share the Road’ warning signs [26], particularly in rural settings, where HGVs are likely to execute overtaking manoeuvres at high speed. Such measures could prompt motor vehicles to maintain safer distances from the edges of travel lanes, especially in areas with a notable presence of both HGVs and bicycles.

We also identified elderly drivers as a factor contributing to overtaking crashes—a finding consistent with relevant research [24]. We found that as individuals age, their risk of being involved in road accidents increases, primarily due to declines in cognitive capabilities. Our study corroborates these findings by showing that older cyclists are more susceptible to accidents during overtaking manoeuvres, which can be attributed to diminished reaction times and impaired decision-making abilities [27], their health [28], and their driving performance [29]. Notably, crashes involving elderly individuals often occur in scenarios with challenging conditions, including at intersections without traffic control measures, on high-speed roads, during adverse weather conditions, in poorly lit areas, and in head-on accidents [30-32]. The heightened level of risk under such conditions may be attributed to cognitive and perceptual decline in older drivers, which could affect their capacity to execute actions such as overtaking manoeuvres safely. Accordingly, developing specialised cognitive training programmes as interventions to enhance road safety for elderly drivers is evidently necessary [33]. Based on our study's findings, we

recommend the development of specialised interventions to improve road safety for elderly cyclists. Our analysis reveals that older cyclists are at a higher risk of being involved in overtaking crashes, with this increased risk being strongly linked to declines in cognitive capabilities associated with aging. To address this issue, we advocate for the implementation of targeted cognitive training programs specifically designed for elderly cyclists. These programs should focus on enhancing critical skills such as reaction time, situational awareness, and decision-making abilities, which are crucial for reducing crash risk and improving overall road safety.

In the present study, several factors were found to increase the risk of rear-end crashes on road segments, including darkness with unlit surroundings, midnight hours, and rural settings (speed limit > 40 mph). Although few studies have specifically addressed rear-end crashes involving bicycles on road segments, available data suggest that the low conspicuity of bicycles, especially at night, is a recurrent factor in rear-end crashes [18, 34]. Moreover, a lack of adequate street lighting, which is common in rural settings, predisposes cyclists to rear-end crashes [18]. Our joint-effects analysis further indicated that the detrimental effect of unlit darkness is more pronounced in rural areas and during midnight hours. Potential intervention strategies to mitigate rear-end crashes include enhancing illumination and executing speed control management on rural road segments with heavy bicycle traffic.

Next, our analysis successfully identified associations of urban areas and taxis and private hire cars as crash partners with door crashes on road segments. Although research specifically focusing on door crashes on road segments is limited, similar findings were documented by Pai, indicating that urban roadways and taxis contributed to door crashes [18]. However, determining the factors influencing this trend poses a challenge. One possible explanation could be the

increased presence of taxis or private hire cars in such areas, where passengers often disembark. Additionally, our analysis further revealed an elevated risk of door crashes involving crashes with taxis in urban areas. To reduce door crashes on road segments, educating taxi drivers, as well as passengers, about the importance of vigilance when opening doors near traffic is essential [18]. In addition, cyclists should be advised to maintain at least a door's width distance from all parked cars to improve the sight triangles of drivers and increase the visibility of cyclists [35]. Implementing a two-stage door opening mechanism for vehicles, which would enable drivers to verify the presence of bicycles to the rear, could also be beneficial [36].

The strengths of this study include the use of STATS19 datasets spanning from 1991 to 2020, which provides a robust statistical foundation and a broad perspective on trends in bicycle crashes. By focusing specifically on three crash types on road segments—overtaking, rear-end, and door crashes—the study provides a comprehensive and focused analysis, which can yield more actionable insights and more effective recommendations. The UK-based dataset ensures that the findings are particularly relevant for local policy and safety interventions. Additionally, the application of statistical techniques and the consideration of various factors, such as crash partner and time of day, enhance the validity and depth of the analysis.

This study had several limitations that warrant acknowledgement. First, the substantial underreporting of nonfatal casualties to the police, particularly casualties involving cyclists not obligated to report accidents, is a critical factor to consider. Such underreporting, as highlighted by the U.K. Government's Department for Transport [11], likely results in the incomplete representation of nonfatal and 'slight' casualties in road casualty data. Second, the STATS19 data utilised in this study lack critical variables, including precrash speeds, specific geometric

characteristics of roadways, data regarding alcohol and illicit substance use, and cyclist speed at the time of an accident. Moreover, critical exposure data—such as those related to traffic flow, rider or driver experience, and other elements of risk exposure—are absent, and the absence of such details limits our ability to fully account for potential variations resulting from unobserved factors in the analyses. Finally, this study did not explore annual trends in each type of bicycle crash over the 30-year study period; investigating such trends could provide insights regarding changing behaviours among cyclists and motor vehicle drivers as well as the effects of legislative changes for road speed limits.

One inherent problem with police-reported crash data is the variables not readily available, hereby causing unobserved heterogeneity across the observations. To overcome such a limitation, we estimated separate regression models, as suggested by Kim et al.[37], for the three crash types; such an approach provides greater explanatory power compared to single overall models. Further, we conducted joint-effect analyses of several variables of interest that capture heterogeneity. In our previous studies, we adopted the above-mentioned approaches to overcome the inherent problem with a success [38, 39].

Future research directions could involve integrating GPS (Global Positioning System) data and weather conditions to analyse both injury frequency and fatalities of bicycle crashes on road segments. Additionally, exploring the potential of autonomous vehicles for detecting approaching bicycles for door-crashes and implementing AI-controlled lighting systems in rural areas for cyclist detection could be promising areas for further study.

Recommendations

For overtaking crashes, we recommend implementing 'Share the Road' warning signs, especially in rural areas, and developing specialized cognitive training programs for elderly drivers. Regarding rear-end crashes, our suggestions include improving illumination during night time and implementing speed control measures on rural road segments. For door crashes involving parked cars, we propose enhancing driver sight triangles and increasing cyclist visibility. Moreover, implementing a two-stage door opening mechanism and an automatic detection device in vehicles to alert drivers of bicycles approaching from behind could potentially be beneficial.

Conclusions

This study identified several significant risk factors for the three predominate types of crashes involving cyclists on road segments: HGVs as crash partners, elderly crash partners, and rural areas for overtaking crashes; unlit darkness, midnight hours, and rural areas for rear-end crashes; and urban areas and taxis as crash partners for door crashes. These risk factors remained unchanged since our previous study conducted in 2011 [15]. The present research enhances the field of bicycle safety research by concluding that the detrimental effects of certain variables become more pronounced under certain conditions. For example, first, cyclists in rural settings exhibited an elevated risk of overtaking crashes involving HGVs. Second, the rear-end crash risk increases in the combined presence of unlit darkness, midnight hours, and rural areas. Finally, in urban settings, the likelihood of door crashes increases when a taxi is the crash partner.

Abbreviations

WHO: World Health Organization; HGVs: heavy goods vehicles; AOR: adjusted odds ratio; CI: confidence interval.

Acknowledgments

This manuscript was edited by Wallace Academic Editing.

Author contributions

Literature review: Chun-Chieh Chao.

Methodology: Chun-Chieh Chao, Chih-Wei Pai.

Data merging and analysis: Akhmad Fajri Widodo, Wafaa Saleh, Bayu Satria Wiratama.

Writing - original draft: Chun-Chieh Chao.

Writing – review and editing: Hui-An Lin, Chenyi Chen, Hon-Ping Ma, Akhmad Fajri Widodo.

Validation: Chun-Chieh Chao, Hui-An Lin, Chenyi Chen, Hon-Ping Ma, Shih Yu Ko.

Supervision: Li Wei, Yen-Nung Lin, Shou-Chien Hsu, Chih-Wei Pai.

Funding: Cheng-Wei Chan, Chih-Wei Pai.

Funding

This study received financial support from the Ministry of Science and Technology, Taiwan (MOST 110–2410-H-038-016-MY2 and MOST 109–2314-B-038-066-); New Taipei City Hospital (NTPC113–002); and the National Science and Technology Council, Taiwan (NSTC 112-2410-H-

038-016-MY2). The funders played no role in the design of the study, data collection and analysis, interpretation of data, or preparation of the manuscript.

Availability of data and materials

This study utilised the British STATS19 database, which contains data on all road traffic accidents in the United Kingdom. The data that support the findings of this study are openly available at https://figshare.com/ndownloader/files/48173452.

Declarations

Ethical approval and consent to participate

This study was conducted in accordance with the Declaration of Helsinki and approved by the Joint Institutional Review Board of Taipei Medical University (N202011030).

Consent for publication

This study was approved by the Joint Institutional Review Board of Taipei Medical University (N202011030). The Joint Institutional Review Board of Taipei Medical University has waived the requirement of informed consent. All methods were performed in accordance with the relevant guidelines and regulations of the Declaration of Helsinki.

Competing interests

The authors declare that they have no competing interests in relation to this work.

Author information

[1]Graduate Institute of Injury Prevention and Control, College of Public Health, Taipei Medical University, Taipei City, Taiwan. [2]Department of Emergency Medicine, Taipei Medical University Hospital, Taipei City, Taiwan. [3]Department of Emergency Medicine, School of Medicine, College of Medicine, Taipei Medical University, Taipei City, Taiwan. [4]Department of Emergency Medicine, New Taipei City Hospital, New Taipei City, Taiwan. [5]College of Medicine, Chang Gung University, Taoyuan City, Taiwan. [6]Department of Emergency Medicine, Chang Gung Memorial Hospital, Linkou branch, Taoyuan, Taiwan.[7] Department of Emergency Medicine, Taipei Medical University-Shuang Ho Hospital, New Taipei City, Taiwan. [8]Taipei Neuroscience Institute, Taipei Medical University, Taipei City, Taiwan. [9]Division of Neurosurgery, Department of Surgery, Wan Fang Hospital, Taipei Medical University, Taipei City, Taiwan. [10]Department of Physical Medicine and Rehabilitation, Wan Fang Hospital, Taipei Medical University, Taipei City, Taiwan. [11]Transport Research Institute, Edinburgh Napier University, Edinburgh, Scotland. [12]Department of Epidemiology, Biostatistics, and Population Health, Faculty of Medicine, Public Health and Nursing, Universitas Gadjah Mada, Yogyakarta City, Indonesia, [13]Department of Occupational Medicine, Chang Gung Memorial Hospital, Linkou branch, Taoyuan, Taiwan.

References:

1. Kjeldgard L, Ohlin M, Elrud R, Stigson H, Alexanderson K, Friberg E. Bicycle crashes and sickness absence - a population-based Swedish register study of all individuals of working ages. BMC Public Health. 2019;19(1):943.
2. World Health Organization. Regional Office for E. Walking and cycling: latest evidence to support policy-making and practice. Copenhagen: World Health Organization. Regional Office for Europe; 2022.
3. Venkatraman V, Richard CM, Magee K, Johnson K. Countermeasures that work: A highway safety countermeasure guide for state highway safety offices, 10th Edition, 2020. (DOT HS 813 097).
4. Allen-Munley C, Daniel J, Dhar S. Logistic model for rating urban bicycle route safety. Transportation Research Record. 2004;1878(1):107-15.
5. Kaplan JA. Characteristics of the Regular Adult Bicycle User. Final Report 1975.
6. Rivara FP, Thompson DC, Thompson RS. Epidemiology of bicycle injuries and risk factors for serious injury. Inj Prev. 1997;3(2):110-4.
7. Wanvik PO. Effects of road lighting: an analysis based on Dutch accident statistics 1987-2006. Accid Anal Prev. 2009;41(1):123-8.
8. Elvik R, Sundfør HB. How can cyclist injuries be included in health impact economic assessments? Journal of Transport & Health. 2017;6:29-39.
9. Aertsens J, de Geus B, Vandenbulcke G, Degraeuwe B, Broekx S, De Nocker L, et al. Commuting by bike in Belgium, the costs of minor accidents. Accident Analysis & Prevention. 2010;42(6):2149-57.
10. Scholten AC, Polinder S, Panneman MJ, Van Beeck EF, Haagsma JA. Incidence and costs of bicycle-related traumatic brain injuries in the Netherlands. Accident Analysis & Prevention. 2015;81:51-60.
11. Traffic Safety Facts - Bicyclists and Other Cyclists. https://crashstats.nhtsa.dot.gov/Api/Public/ViewPublication/813322: National Highway Traffic Safety Administration; 2020.
12. Bil M, Bilova M, Muller I. Critical factors in fatal collisions of adult cyclists with automobiles. Accid Anal Prev. 2010;42(6):1632-6.
13. Moore DN, Schneider WHt, Savolainen PT, Farzaneh M. Mixed logit analysis of bicyclist injury severity resulting from motor vehicle crashes at intersection and non-intersection locations. Accid Anal Prev. 2011;43(3):621-30.
14. Wachtel A, Lewiston D. Risk factors for bicycle-motor vehicle collisions at intersections. ITE journal. 1994;64(9):30-5.
15. Ugan J, Abdel-Aty M, Cai Q, Mahmoud N, Al-Omari Me. Effect of various speed management strategies on bicycle crashes for urban roads in Central Florida. Transportation Research Record: Journal of the Transportation Research Board. 2021;2676:036119812110366.
16. Meuleners LB, Fraser M, Johnson M, Stevenson M, Rose G, Oxley J. Characteristics of the road infrastructure and injurious cyclist crashes resulting in a hospitalisation. Accident Analysis & Prevention. 2020;136:105407.
17. Robartes E, Chen T-D. The effect of crash characteristics on cyclist injuries: An analysis of Virginia automobile-bicycle crash data. Accid Anal Prev. 2017;104:165-73.

18. Pai C-W. Overtaking, rear-end, and door crashes involving bicycles: an empirical investigation. Accid Anal Prev. 2011;43(3):1228-35.
19. Debnath AK, Haworth N, Schramm A, Heesch KC, Somoray K. Factors influencing noncompliance with bicycle passing distance laws. Accid Anal Prev. 2018;115:137-42.
20. Vandenbrouckel JP, von Elm E, Altman DG, Gotzsche PC, Mulrow CD, Pocock SJ, et al. Strengthening the Reporting of Observational Studies in Epidemiology (STROBE): explanation and elaboration. PLoS Medicine. 2007;4(10):1628-55.
21. Watson A, Watson B, Vallmuur K. Estimating under-reporting of road crash injuries to police using multiple linked data collections. Accident Analysis & Prevention. 2015;83:18-25.
22. Maldonado G, Greenland S. Simulation study of confounder-selection strategies. American Journal of Epidemiology. 1993;138(11):923-36.
23. Chen P-L, Pai C-W. Evaluation of injuries sustained by motorcyclists in approach-turn crashes in Taiwan. Accident Analysis & Prevention. 2019;124:33-9.
24. Liu J, Jones S, Adanu EK, Li X. Behavioral pathways in bicycle-motor vehicle crashes: From contributing factors, pre-crash actions, to injury severities. J Safety Res. 2021;77:229-40.
25. Walker I. Signals are informative but slow down responses when drivers meet bicyclists at road junctions. Accid Anal Prev. 2005;37(6):1074-85.
26. Kay JJ, Savolainen PT, Gates TJ, Datta TK. Driver behavior during bicycle passing maneuvers in response to a Share the Road sign treatment. Accid Anal Prev. 2014;70:92-9.
27. Anstey KJ, Horswill MS, Wood JM, Hatherly C. The role of cognitive and visual abilities as predictors in the Multifactorial Model of Driving Safety. Accid Anal Prev. 2012;45:766-74.
28. Kandasamy D, Betz ME, DiGuiseppi C, Mielenz TJ, Eby DW, Molnar LJ, et al. Self-reported health conditions and related driving reduction in older drivers. Occup Ther Health Care. 2018;32(4):363-79.
29. Laosee O, Rattanapan C, Somrongthong R. Physical and cognitive functions affecting road traffic injuries among senior drivers. Arch Gerontol Geriatr. 2018;78:160-4.
30. Cicchino JB, Wells JK, McCartt AT. Survey about pedestrian safety and attitudes toward automated traffic enforcement in Washington, D.C. Traffic Inj Prev. 2014;15(4):414-23.
31. Kostyniuk LP, Molnar LJ. Self-regulatory driving practices among older adults: health, age and sex effects. Accid Anal Prev. 2008;40(4):1576-80.
32. Zhang J, Lindsay J, Clarke K, Robbins G, Mao Y. Factors affecting the severity of motor vehicle traffic crashes involving elderly drivers in Ontario. Accid Anal Prev. 2000;32(1):117-25.
33. Edwards JD, Ross LA, Wadley VG, Clay OJ, Crowe M, Roenker DL, et al. The useful field of view test: normative data for older adults. Arch Clin Neuropsychol. 2006;21(4):275-86.
34. Wood JM, Lacherez PF, Marszalek RP, King MJ. Drivers' and cyclists' experiences of sharing the road: incidents, attitudes and perceptions of visibility. Accid Anal Prev. 2009;41(4):772-6.
35. Hunter W, Stewart JR. An evaluation of bike lanes adjacent to motor vehicle parking. 1999.
36. Huang C-Y. Observations of drivers' behavior when opening car doors. Procedia Manufacturing. 2015;3:2753-60.
37. Kim D-G, Washington S, Oh J. Modeling crash types: New insights into the effects of covariates on crashes at rural intersections. Journal of Transportation Engineering. 2006;132(4):282-92.
38. Pai C-W, Saleh W. Modelling motorcyclist injury severity by various crash types at T-junctions in the UK. Safety Science. 2008; 13:89-98.

39. Pai C-W, Jou R-C. Cyclists' red-light running behaviours: An examination of risk-taking, opportunistic, and law-obeying behaviours. Accident Analysis & Prevention. 2014;62:191-8.

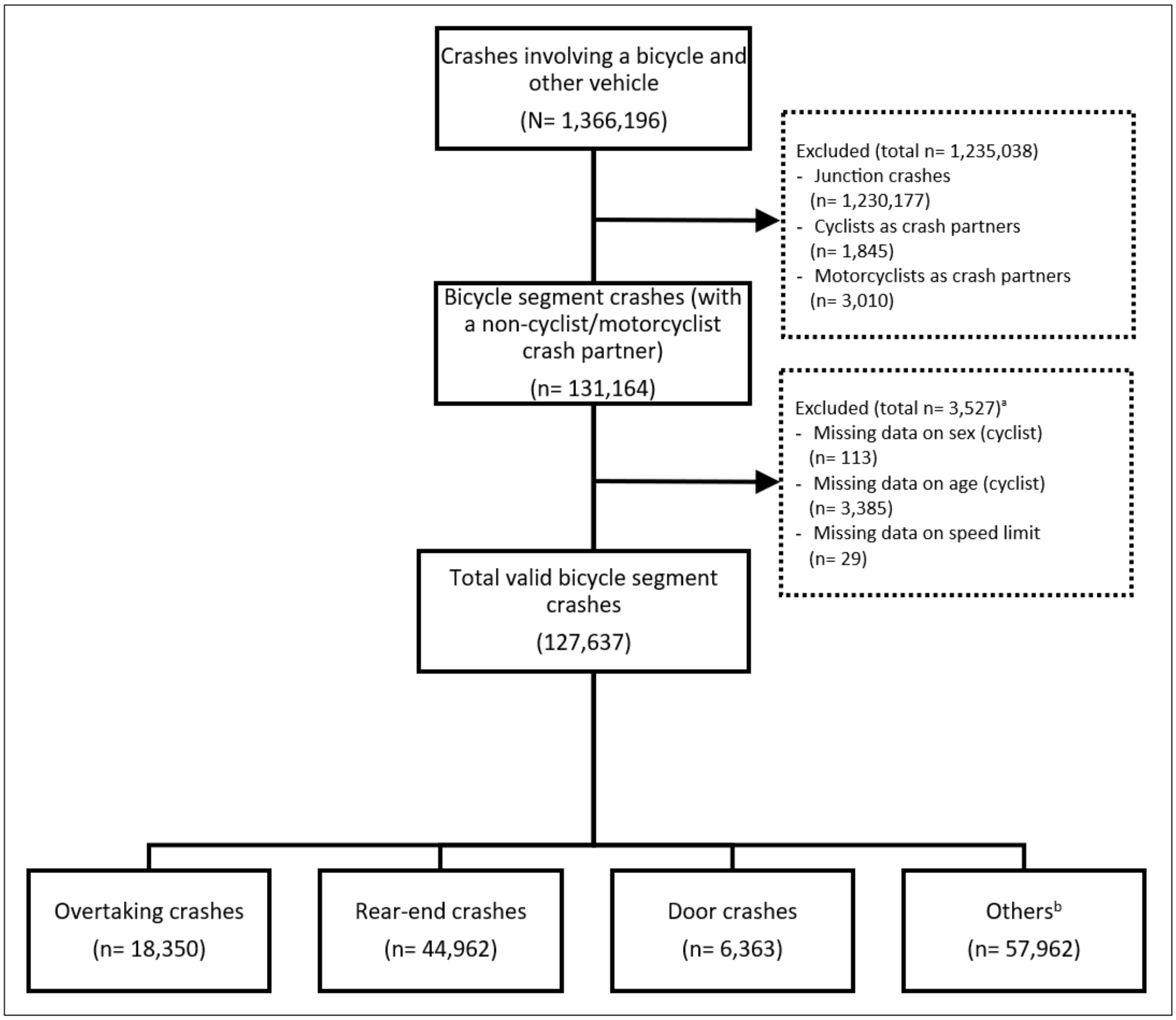

Fig. 1. Flowchart of the study sample selection process. [a]Listed excluded criteria are nonexclusive; thus, the sum of the total may exceed 3,527. [b]Other crashes include reversing crashes and head-on crashes.

Figure 2 Click here to access/download;Figure;Figure 2.docx

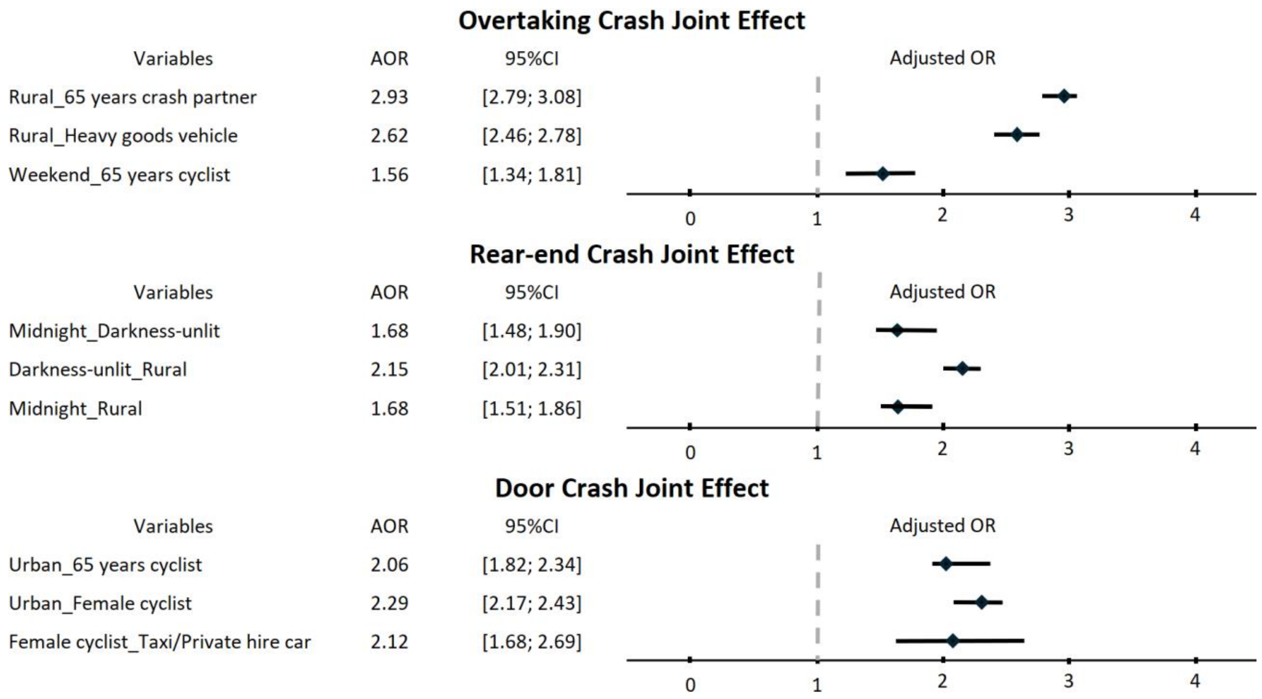

Fig. 2. Joint effects of several variables on the three crash types.

Click here to access/download;Figure;7-31 Figure 2.docx

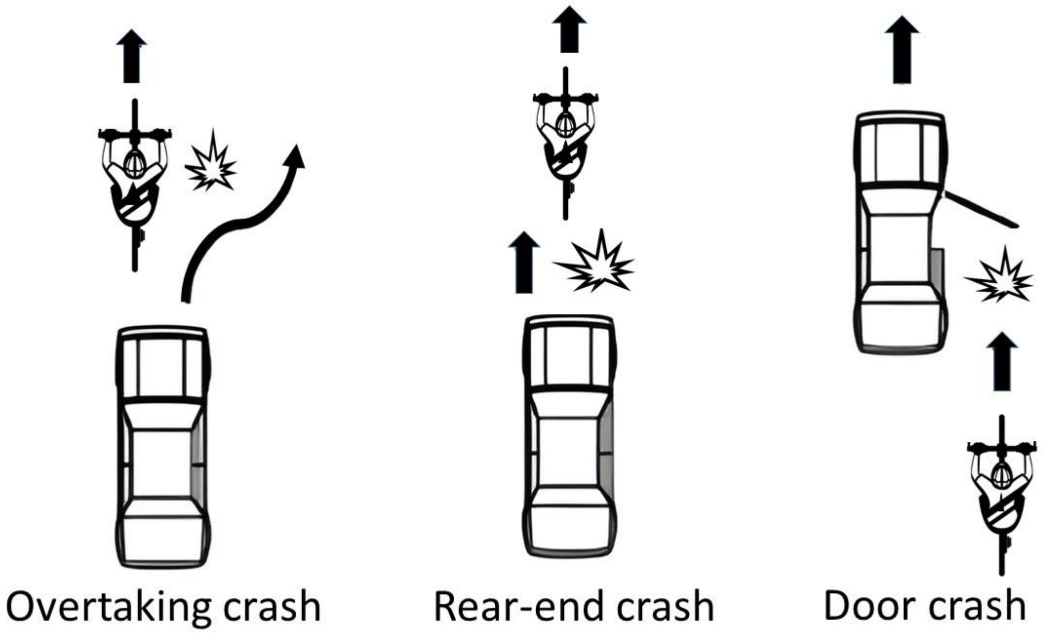

Figure 2. Illustrative diagram of the three crash types

 Click here to access/download;Figure;8-5 Figure 3.docx

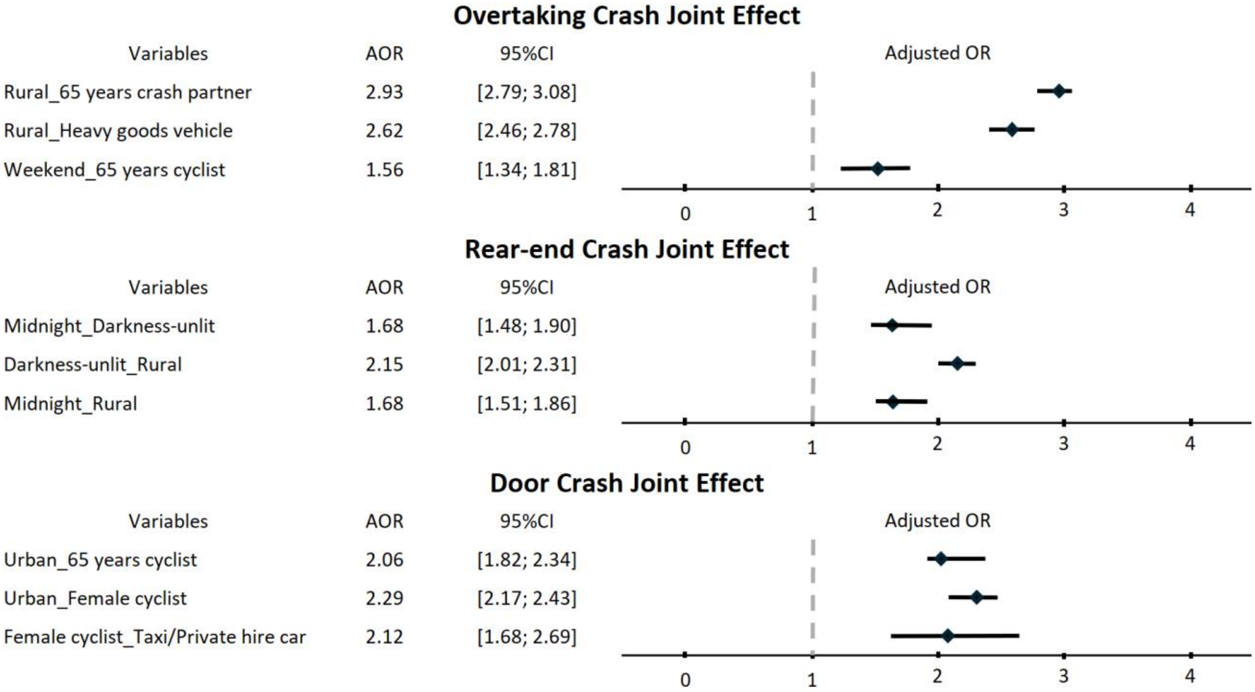

Fig. 3. Joint effects of several variables on the three crash types.

Risk Factors for Overtaking, Rear-End, and Door Crashes Involving Bicycles in the United Kingdom: Revisited and Reanalysed

Chun-Chieh Chao[1,2,3†], Hon-Ping Ma[1,3,7], Li Wei[1,8,9], Yen-Nung Lin[1,10], Chenyi Chen[1], Wafaa Saleh[11], Bayu Satria Wiratama[12], Akhmad Fajri Widodo[1], Shou-Chien Hsu[6,13], Shih Yu Ko[7], Hui-An Lin[1,2,3†], Cheng-Wei Chan[1,4,5,6†], Chih-Wei Pai[1*]

[1] Graduate Institute of Injury Prevention and Control, College of Public Health, Taipei Medical University, Taipei City, Taiwan, [2] Department of Emergency Medicine, Taipei Medical University Hospital, Taipei City, Taiwan, [3] Department of Emergency Medicine, School of Medicine, College of Medicine, Taipei Medical University, Taipei, Taiwan, [4] Department of Emergency Medicine, New Taipei City Hospital, New Taipei City, Taiwan, [5] College of Medicine, Chang Gung University, Taoyuan City, Taiwan, [6] Department of Emergency Medicine, Chang Gung Memorial Hospital, Linkou branch, Taoyuan, Taiwan, [7] Department of Emergency Medicine, Taipei Medical University-Shuang Ho Hospital, New Taipei City, Taiwan, [8] Taipei Neuroscience Institute, Taipei Medical University, Taipei, Taiwan, [9] Division of Neurosurgery, Department of Surgery, Wan Fang Hospital, Taipei Medical University, Taipei, Taiwan, [10] Department of Physical Medicine and Rehabilitation, Wan Fang Hospital, Taipei Medical University, Taipei, Taiwan, [11] Transport Research Institute, Edinburgh Napier University, Edinburgh, Scotland, [12] Department of Epidemiology, Biostatistics, and Population Health, Faculty of Medicine, Public Health and Nursing, Universitas Gadjah Mada, Yogyakarta City, Indonesia, [13] Department of Occupational Medicine, Chang Gung Memorial Hospital, Linkou branch, Taoyuan, Taiwan.

* Correspondence: cpai@tmu.edu.tw

† Contributed equally to this work

Formatted: Right: 0.25"

Abstract

Background: Relevant research has provided valuable insights into risk factors for bicycle crashes at intersections. However, few studies have focused explicitly on three common types of bicycle crashes on road segments: overtaking, rear-end, and door crashes.

Objective: This study aims to identify risk factors for overtaking, rear-end, and door crashes that occur on road segment.

Material and methods

~~The current study examined the risk factors associated with these three types of crashes occurring on road segments.~~ We analysed British STATS19 accident records from 1991 to 2020. Using multivariate logistic regression models, we estimated adjusted odds ratios (AORs) with 95% confidence intervals (CIs) for multiple risk factors. The analysis included 127,637 bicycle crashes, categorised into 18,350 overtaking, 44,962 rear-end, 6,363 door, and 57,962 other crashes.

Results

Significant risk factors for overtaking crashes included speed limits of ≥40 miles per hour (mph) (AOR = 2.238, 95% CI = 2.159–2.320), heavy goods vehicles (HGVs) as crash partners (AOR = 2.867, 95% CI 2.473–3.323), and elderly crash partners (AOR = 2.013, 95% CI = 1.937–2.092). For rear-end crashes, noteworthy risk factors included unlit darkness (AOR = 1.486, 95% CI = 1.404–1.573) and midnight hours (AOR = 1.269, 95% CI = 1.190–1.354). Factors associated with door crashes included speed limits of 20–30 mph (AOR = 16.185, 95% CI = 13.514–19.382) and taxi and private hire cars (AOR = 2.695, 95% CI = 2.310–3.145). Our joint-effect analysis revealed additional interesting results; for example, there were elevated risks for overtaking crashes in rural areas with elderly drivers as crash partners (AOR = 2.93, 95% CI = 2.79–3.08) and with HGVs as crash

Formatted: Font color: Text 1

Formatted: Font color: Text 1

Formatted: Right: 0.25"

partners (AOR = 2.62, 95% CI = 2.46–2.78).

Conclusions

The aforementioned risk factors remained largely unchanged since 2011, when we conducted our previous study. However, the present study concluded that the detrimental effects of certain variables became more pronounced in certain situations. For example, cyclists in rural settings exhibited an elevated risk of overtaking crashes involving HGVs as crash partners.

Keywords: Bicycle crash; Road segment; Overtaking crash; Rear-end crash; Door crash

Introduction

In recent years, urban bicycling has become increasingly popular in many countries, offering benefits such as reduced traffic congestion, diminished parking pressure, and a reduction in greenhouse gas emissions [1, 2]. The World Health Organization has highlighted numerous health advantages of moderate-intensity physical activities such as bicycling, including improvements in life expectancy, quality of life, cognitive function, mental health, sleep quality, muscular and cardiorespiratory fitness, and bone and functional health [2].

However, despite such health benefits, the risk of injury remains a considerable safety concern for cyclists, who are regarded as vulnerable road users [2, 3]. Traffic crash data indicate that the risk of accidents for cyclists, measured per distance travelled, is approximately 20 times higher than that for vehicle drivers[2]. To address this problem, researchers in the United States

Formatted: Right: 0.25"

developed a comprehensive bicycle route safety rating model with a focus on injury severity [4]. This model evaluates multiple operational and physical aspects such as traffic volume, population density, highway classification, lane width, and the presence of one-way streets. In addition, it is capable of predicting the severity of injuries due to motor vehicle–related crashes at specific locations [4]. Another finding was that a route is considered adequately safe if it includes geometric factors that enhance safety [4]. This model can aid urban planners and public officials in creating infrastructure such as bike lanes and implementing strict lane policies to improve cyclist safety [4]. Implementing bike lanes has been demonstrated to reduce crash rates by up to 40% among adult cyclists [5]. One study regarding roundabouts indicated that roundabouts with cycle tracks significantly reduced injury risk for cyclists compared with those lacking bicycle infrastructure [6]. Furthermore, adequate night-time lighting on rural roads has the potential to prevent over half of all cyclist injuries [7]. Bicycle crashes can also impose a significant burden on healthcare expenses. Elvik and Sundfør [8] have discussed the economic implications and healthcare expenditures associated with bicycle accidents. For instance, in Belgium, the average cost of bicycle accidents per case is estimated at 841 euros [9]. In the Netherlands, the total annual cost has been reported as €410.7 million [10].

Although intersectional crashes are generally more frequent than nonintersectional ones, in 2020, 64% of fatal crashes involving cyclists occurred on road segments, defined as areas 20 m away from intersections, whereas only 26% of such fatalities occurred at intersections [11]. Bil et al. demonstrated that car drivers, when at fault for crashes, often cause more serious consequences for cyclists on straight road sections [12]. In crashes occurring on road segments, several factors contribute to high injury severity, including being in a rural region with an elevated

speed limit, male gender, and cyclist age of >55 years [13]. Another identified risk factor is bicycling on roads against oncoming traffic [14].

Although relevant research has shed light on risk factors for bicycle crashes at intersections, few studies have explicitly investigated crashes on road segments. Bicycle crashes on road segments remain a substantial issue for public health concern. ~~Existing research primarily emphasizes intersection-related crashes.~~ This study aims to fill a critical gap by conducting a thorough examination of the risk factors associated with three distinct bicycle crash types: overtaking, rear-end, and door crashes that occur on road segments. Studies that have examined bicycle crashes relatively broadly, without distinguishing crash types, have identified several key factors—including vehicle volume [15], traffic density [16], number of lanes [16], access points along road segments [15], shoulder and median widths [15], parking space availability [15, 16] , length of continuous two-way left-turn lanes [15], and pavement type [17]—all of which contribute to bicycle crashes on road segments. ~~Several studies have specifically explored overtaking, rear-end, and door crashes involving bicycles. The primary objective of this study, building on our previous research~~Two exceptional work have examined ~~into~~ risk factors ~~related to~~for overtaking, rear-end, and door crashes~~, is to conduct a more comprehensive investigation.~~ [18, 19]. Specifically, Pai identified buses and coaches as common crash partners in overtaking crashes~~;~~, poor visibility, traversing manoeuvres, and teenage cyclists as risk factors for rear-end crashes~~;~~, and built-up areas as a risk factor for door crashes [18]. In addition, another study linked the speed of a passing vehicle to increased severity of cyclist injury in overtaking crashes [19].

The primary objective of the present study, an extension of our previous study [18], was to analyse police-reported crash data from additional years to determine whether the risk factors

Formatted: Font color: Text 1

Formatted: Font color: Text 1

Formatted: Font color: Text 1

Formatted: Font color: Text 1

Formatted: Font color: Text 1

Formatted: Font color: Text 1

Formatted: Font color: Text 1

Formatted: Right: 0.25"

for these three crash types remained unchanged. The study addresses a critical gap in current research, focusing on crashes specifically occurring on road segments. Existing literature offers limited insights into ~~this specific type of crash~~these crash types, highlighting a crucial need for targeted investigations. These crashes have the potential for severe impacts, involving complex dynamics that demand a nuanced understanding for effective mitigation strategies. By exploring these factors, our research aims to significantly enhance cyclist safety within this particular context. Furthermore, we aimed to untangle the joint associations of several factors—including light conditions, urban versus rural settings, vehicle types, and rider and driver characteristics—with these three crash types.

Formatted: Font color: Text 1

Material and Methods

Crash data source

The present investigation utilised data from 01/01/1991 to 31/12/2020, obtained from the United Kingdom's official road traffic casualty database, STATS19. Police record such data either at crash scenes or within 30 days of each crash. The UK's Department for Transport compiles the data, which the United Kingdom Data Archive then maintains and distributes. The dataset encompasses a variety of variables, including crash circumstances (e.g., time and date, weather conditions, road and light conditions, posted speed limit, road type), vehicle and driver characteristics, demographic details of the drivers, precrash manoeuvres of the vehicles, and the initial impact point of the vehicle. Additionally, the dataset contains demographic information and details regarding injury severity for each casualty. This study adhered to the STROBE (strengthening the reporting of observational studies in epidemiology) reporting guidelines.[20]

Formatted: Right: 0.25"

Injury severity in the aforementioned dataset is divided into three categories, namely slight, serious, and fatal. Fatal injuries refer to those leading to death within 30 days of the accident. Serious injuries include conditions such as fractures, internal injuries, severe cuts and lacerations, concussions, and any injury requiring hospitalisation. Slight injuries include sprains, bruises, and minor cuts, as well as mild shock requiring roadside attention. The exclusive focus of this study was crashes leading to cyclist casualties.

As shown in Figure 1, this study analysed 1,366,196 crashes involving bicycles and other vehicles. Initially, 1,235,032 junction cases were excluded. From the remaining 131,164 bicycle segment crashes, 3,527 were further excluded because of incomplete demographic data for the cyclist and missing speed limit information, leaving a valid cohort of 127,637 bicycle segment crashes for analysis. Within this cohort, this study identified 18,350 overtaking crashes, 44,962 rear-end crashes, 6,363 door crashes, and 57,962 other types of crashes.

Formatted: Right: 0.25"

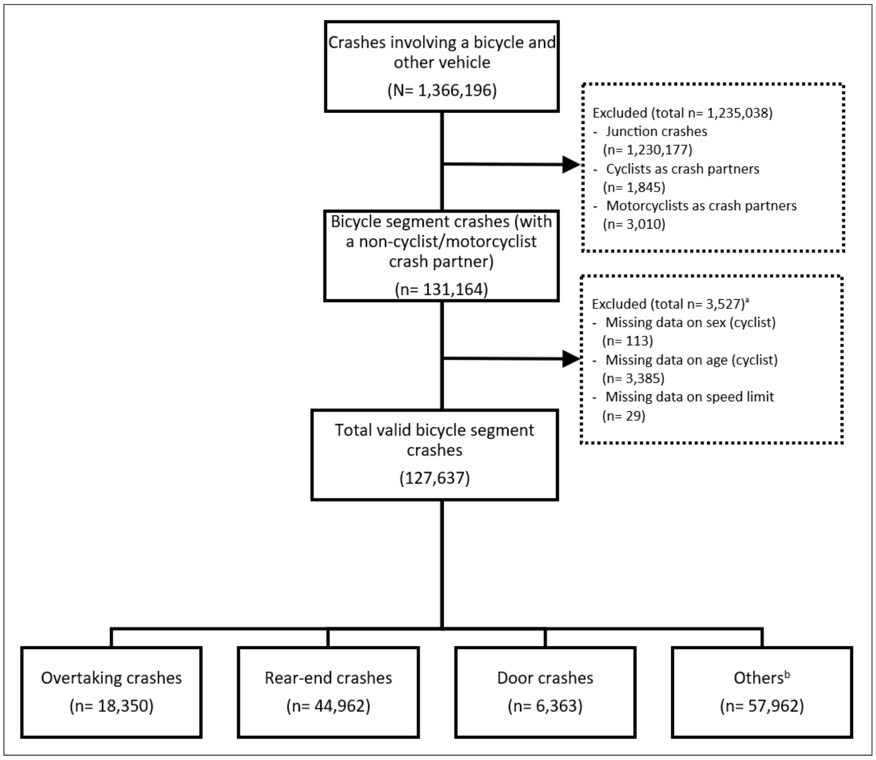

Figure. 1. Flowchart of the study sample selection process. [a]Listed excluded criteria are nonexclusive; thus, the sum of the total may exceed 3,527. [b]Other crashes include reversing crashes and head-on crashes.

Classification of crash types

As shown in Ffigure 2, an overtaking crash is defined as a crash where a motorised vehicle overtakes and impacts with a bicycle, which may be travelling straight, overtaking another vehicle, changing lanes, or turning. A rear-end crash occurs when a following vehicle impacts with the rear of a bicycle. A door crash involves a bicycle either being struck by or striking the opening door of an automobile. These three crash types were described using schematics in our previous study [18].

Formatted: Right: 0.25"

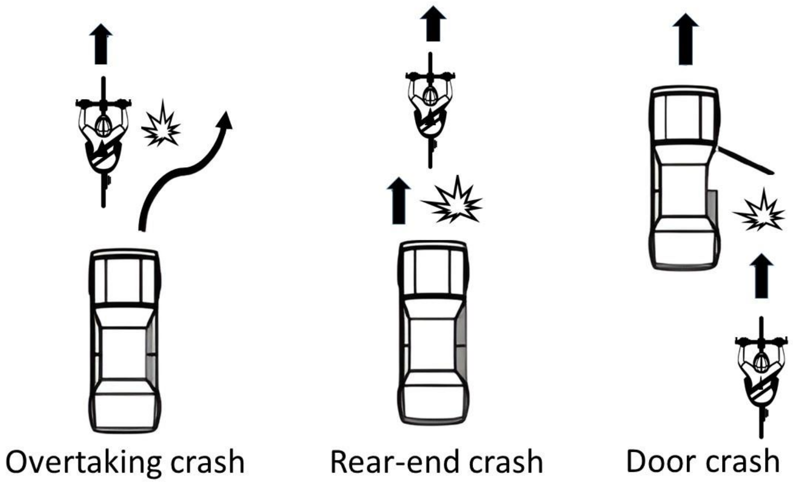

Figure 2. Illustrative diagram of the three crash types

Data collection

For the present study, the three crash types of focus (overtaking, rear-end, and door crashes) were the binary-dependent variables. The collected data encompassed the following factors: lighting conditions on the roadway at the time of the crash (daylight, darkness-lit, darkness-unlit), the speed limit at the crash scene (rural: ≥40 miles per hour [mph]; urban: 20–30 mph), the time of day categorised into four periods according to traffic volume (midnight: 00:00–06:00; rush hours: 07:00–08:00 and 17:00–18:00; nonrush hours: 09:00–16:00; and evening: 19:00–23:00), and the day of the week (weekday or weekend day). The demographic details of cyclist casualties encompassed age (≤18, 19–40, 41–64, or ≥65 years) and sex (male or female). Finally, the demographic details of the crash partner included the type of vehicle (identified as a taxi, private hire car, car, bus, or heavy goods vehicle [HGV]), age (≤18, 19–40, 41–64, or ≥65 years) and sex (male or female). On a cautionary note, we removed junction cases to avoid the variability

introduced when exogenous factors, such as junction geometry and control measures, are present at junctions. Furthermore, the cases involving other cyclists and motorcyclists were removed as we focused on vehicle-cycle crashes only. Missing data on sex, age, or speed limits were also excluded in the analysis. Excluding these data may impact our results in a marginal scale, as these data are likely to be single-bicycle crashes that in nature be underreported in police crash dataset [21].

Statistical analysis

This study employed the chi-squared test to examine the associations between crash type and other factors, including cyclist or motorist characteristics, vehicle features, roadway conditions, and temporal variables. Initially, we examined the distribution of three crash types across various variables to explore their relationships with a binary outcome. These variables included lighting conditions, speed limit, time of day, and day of the week. Demographic details concerning cyclist casualties encompassed age and sex, while information about the crash partner included vehicle type, age, and sex. We set a significance level of $p < 0.2$ to include risk factors in our multivariate analysis [23]. Adjusted odds ratios (AORs) were computed using multivariate logistic regression with backward selection.[22, 23]

The multivariate logistic regression model equation was specified as:

$$log\left(\frac{P(Y=1)}{1-P(Y=1)}\right)=\beta_0+\beta_1X_1+\beta_2X_2$$

where $P(Y=1)$ denotes the probability of the outcome, $\beta_0,\beta_1,\beta_2,\dots,\beta_p$ are the coefficients to be

Formatted: Font color: Text 1
Formatted: Font color: Text 1
Formatted: Justified
Formatted: Font color: Text 1
Formatted: Font color: Text 1
Formatted: Font color: Text 1
Formatted: Font color: Text 1
Formatted: Font color: Text 1
Formatted: Font color: Text 1
Formatted: Font color: Text 1
Formatted: Font color: Text 1
Formatted: Font color: Text 1
Formatted: Font color: Text 1
Formatted: Font color: Text 1
Formatted: Font color: Text 1
Formatted: Font color: Text 1
Formatted: Font color: Text 1
Formatted: Font color: Text 1
Formatted: Font color: Text 1
Formatted: Font color: Text 1
Formatted: Font color: Text 1
Formatted: Font color: Text 1
Formatted: Font color: Text 1
Formatted: Font color: Text 1
Formatted: Font color: Text 1
Formatted: Font color: Text 1
Formatted: Right: 0.25"

estimated, and $X_1, X_2, \ldots, X_p$ represent the predictor variables.

Before estimating the model, assumptions of logistic regression, such as linearity of the logit, absence of multicollinearity, and independence of observations, were evaluated. An odds ratio (OR) greater than 1 indicated a positive association between the independent variable and the occurrence rate, while an OR less than 1 indicated a negative association. An OR of 1 suggested no association between the variables of interest and the outcomes. All statistical analyses were conducted using SPSS Statistics version 25 for Windows (IBM Corp., Armonk, New York, USA). A p value lower than 0.05 in two-tailed tests was considered statistically significant.

Results

Population characteristics

Tables 1, 2, and 3 present the distributions of overtaking, rear-end, and door crashes, respectively, in relation to multiple independent variables. These data revealed that a significant proportion of bicycle crashes occurred in daylight (82.3%), occurred in urban settings (78.5%), occurred during nonrush hours (48.3%), occurred on weekdays (77.5%), involved cyclists aged under 18 years (40.1%), and involved male cyclists (81.3%). Additionally, most crashes involved cars as crash partners (83.6%), and crash partners were predominately aged 19–40 years (38.5%) and were male (76.4%). Table 1 highlights an overrepresentation in bicycle overtaking crashes for certain variables, namely unlit darkness (19.5%), rural areas (24.8%), midnight hours (17.7%), buses or HGVs as crash partners (24.7%), and elderly crash partners (21.5%) and male crash partners (16.0%). These results were revealed to be statistically significant by the chi-squared

Formatted: Right: 0.25"

test ($p < 0.01$).

Table 1. Distribution of overtaking crashes according to a set of independent variables

Variable	Total (n=127,637)	Overtaking crashes (n=18,350)	Non-overtaking crashes (n=109,287)	***χ2 test p value***
**Light conditions**, n (%)				<0.001
Daylight	105,053 (82.3%)	15,283 (14.55%)	89,770 (85.5%)	
Darkness-lit	16,543 (13.0%)	1,889 (11,42%)	14,654 (88.6%)	
Darkness-unlit	6,041 (4.7%)	1,178 (19.50%)	4,863 (80.5%)	
**Speed limit**, n (%)				<0.001
Rural (≥ 40 mph)	27,395 (21.5%)	6,805 (24.8%)	20,590 (75.6%)	
Urban (20–30 mph)	100,242 (78.5%)	11,545 (11.5%)	88,697 (88.5%)	
**Crash time (h)**, n (%)				<0.001
Midnight (00:00–06:00)	4,810 (3.8%)	852 (17.7%)	3,958 (82.3%)	
Rush hours (07:00–08:00/17:00–18:00)	41,619 (32.6%)	5,685 (13.7%)	35,934 (86.3%)	
Nonrush hours (09:00–16:00)	61,696 (48.3%)	9,386 (15.2%)	52,310 (84.8%)	
Evening (19:00–23:00)	19,512 (15.3%)	2,427 (12.4%)	17,085 (87.6%)	
**Crash day**, n (%)				0.094
Weekend	28,730 (22.5%)	4,218 (14.7%)	24,512 (85.2%)	
Weekday	98,907 (77.5%)	14,132 (14.3%)	84,775 (85.7%)	
**Cyclist's age (years)**, n (%)				<0.001
≤18	51,193 (40.1%)	5,220 (10.2%)	45,973 (89.8%)	
19–40	45,760 (35.9%)	7,108 (15.5%)	38,652 (84.5%)	
41–64	26,052 (20.4%)	5,012 (19.2%)	21,040 (80.8%)	
≥65	4,632 (3.6%)	1,010 (21.8%)	3,622 (78.2%)	
**Cyclist's sex**, n (%)				<0.001
Male	103,766 (81.3%)	14,746 (14.2%)	89,020 (85.8%)	
Female	23,871 (18.7%)	3,604 (15.1%)	20,267 (84.9%)	
**Crash partner**, n (%)				<0.001
Taxi/Private hire car	2,588 (2.0%)	208 (8.0%)	2,380 (92.0%)	
Car	106,668 (83.6%)	13,599 (12.8%)	93,069 (87.3%)	
Bus/Heavy goods vehicle	18,381 (14.4%)	4,543 (24.7%)	13,838 (75.3%)	
**Crash partner's age (years)**, n (%)				<0.001
≤18	2,415 (1.9%)	281 (11.6%)	2,134 (88.4%)	
19–40	49,103 (38.5%)	5,398 (11.0%)	43,705 (89.0%)	
41–64	35,598 (27.9%)	3,973 (11.2%)	31,625 (88.8%)	
≥65	40,521 (31.8%)	8,698 (21.5%)	31,823 (78.5%)	
**Crash partner's sex**, n (%)				<0.001
Male	97,447 (76.4%)	15,584 (16.0%)	81,863 (84.0%)	
Female	30,190 (23.8%)	2,766 (9.2%)	27,424 (90.8%)	

Several variables in Table 2 reveal significant differences between rear-end crashes and non-rear-end crashes. Specifically, a higher proportion of rear-end crashes occurred under darkness-unlit conditions (50.2%) compared to darkness-lit conditions (37.5%). Additionally, rear-end crashes were more prevalent in rural areas with speed limits of ≥ 40 mph (43.0%) compared to

Formatted: Font color: Text 1

Formatted: Right: 0.25"

urban areas with speed limits of 20–30 mph (33.1%). Crashes involving crash partners aged ≥ 65 accounted for 39.7% of rear-end crashes, which was higher compared to other age groups (age 41–64: 33.0% and ≤18: 36.0%). Furthermore, rear-end crashes were more likely to occur during midnight (47.6%) compared to rush hours (36.3%). Taxis were frequently involved in rear-end crashes (42.4%), as were male crash partners (36.8%). These findings highlight the significant influence of various factors on the likelihood of rear-end crashes. Variables such as darkness-unlit conditions, higher speed limits in rural areas, crash time, and characteristics of the crash partner all emerged as significant determinants. Specifically, rear-end crashes were notably more prevalent under darkness-unlit conditions, in rural areas with higher speed limits, during midnight hours, and involving certain characteristics of crash partners. Importantly, these associations were statistically significant, as indicated by the Chi-squared test ($p < 0.001$).

Table 2. Distribution of rear-end crashes according to a set of independent variables

Variable	Total (n=127,637)	Rear-end crashes (n=44,962)	Non-rear-end crashes (n=82,675)	*χ2 test p value*
**Light conditions**, n (%)				<0.001
Daylight	105,053 (82.3%)	35,726 (34.1%)	69,333 (66.0%)	
Darkness-lit	16,543 (13.0%)	6,204 (37.5%)	10,339 (63.5%)	
Darkness-unlit	6,041 (4.73%)	3,032 (50.19%)	3,003 (49.71%)	
**Speed limit**, n (%)				<0.001
Rural (≥ 40 mph)	27,395 (21.5%)	11,788 (43.0%)	15,607 (57.0%)	
Urban (20–30 mph)	100,242 (78.5%)	33,174 (33.1%)	67,068 (66.9%)	
**Crash time (h)**, n (%)				<0.001
Midnight (00:00–06:00)	4,810 (3.8%)	2,289 (47.6%)	2,521 (52.4%)	
Rush hours (07:00–08:00/17:00–18:00)	41,619 (32.6%)	15,089 (36.3%)	26,530 (63.7%)	
Nonrush hours (09:00–16:00)	61,696 (48.3%)	20,723 (33.6%)	40,973 (66.4%)	
Evening (19:00–23:00)	19,512 (15.3%)	6,861 (36.2%)	12,651 (64.9%)	
**Crash day**, n (%)				<0.001
Weekend	28,730 (22.5%)	9,485 (33.0%)	19,245 (67.0%)	
Weekday	98,907 (77.5%)	35,477 (35.9%)	63,430 (64.1%)	
**Cyclist's age (years)**, n (%)				<0.001
≤18	51,193 (40.1%)	13,446 (26.3%)	37,747 (73.7%)	
19–40	45,760 (35.9%)	19,102 (41.7%)	26,658 (58.3%)	
41–64	26,052 (20.4%)	10,619 (40.8%)	15,433 (59.2%)	
≥65	4,632 (3.6%)	1,795 (38.8%)	2,837 (61.3%)	
**Cyclist's sex**, n (%)				<0.001
Male	103,766 (81.3%)	37,175 (35.8%)	66,591 (64.2%)	
Female	23,871 (18.7%)	7,787 (32.6%)	16,084 (67.4%)	
**Crash partner**, n (%)				<0.001
Taxi/Private hire car	2,588 (2.0%)	1,096 (42.4%)	1,492 (57.7%)	
Car	106,668 (83.6%)	37,202 (34.9%)	71,342 (66.9%)	
Bus/Heavy goods vehicle	18,381 (14.4%)	6,664 (36.3%)	9,841 (53.5%)	
**Crash partner's age (years)**, n (%)				<0.001
≤18	2,415 (1.9%)	870 (36.0%)	1,545 (64.0%)	
19–40	49,103 (38.5%)	16,282 (33.2%)	32,821 (66.8%)	
41–64	35,598 (27.9%)	11,736 (33.0%)	23,862 (67.0%)	
≥65	40,521 (31.8%)	16,074 (40.0%)	24,447 (60.3%)	
**Crash partner's sex**, n (%)				<0.001

Formatted: Right: 0.25"

Male	97,447 (76.6%)	35,828 (36.8%)	61,619 (63.2%)
Female	30,190 (23.7%)	9,134 (30.3%)	21,056 (69.7%)

As shown in Table 3, several variables can contribute to door crashes involving bicycles. Door crashes predominantly occurred in urban areas with speed limits of 20-30 mph (6.2%), while a significantly lower proportion occurred in rural areas with speed limits ≥ 40 mph (0.5%). These crashes were overrepresented during non-rush hours (5.5%) and rush hours (4.9%) compared to evening (4.3%) and midnight (2.4%). Cyclists were more frequently involved in door crashes on weekdays (5.4%) than weekends (3.7%). As many as 8.2% of all female cyclists were involved in door crashes, which is higher than the involvement rate among males (4.2%). Taxi and private hire cars were overinvolved in door crashes (10.6%) compared to cars (5.2%) and buses/heavy goods vehicles (3.1%). Crash partners aged ≤18 years (5.2%) and 19-40 years (5.3%) were disproportionately involved in door crashes compared to older age groups, and female crash partners were overrepresented in door crashes (7.4%) compared to males (4.2%). These results were statistically significant, as indicated by the Chi-squared test ($p < 0.001$). They suggest that various factors—including traffic conditions (rural areas, crash time), cyclist demographics (younger age, gender), and characteristics of the crash partner (taxi/private hire cars)—significantly contribute to the likelihood of door crashes involving cyclists.

Table 3. Distribution of door crashes according to a set of independent variables

Variable	Total (n=127,637)	Door crashes (n=6,363)	Non-door crashes (n=121,274)	***χ2 test p value***
**Light conditions**, n (%)				<0.001
Daylight	105,053 (82.3%)	5,192 (4.9%)	99,861 (95.1%)	
Darkness-lit	16,543 (13.0%)	1,031 (6.2%)	15,512 (93.8%)	
Darkness-unlit	6,041 (4.7%)	140 (2.3%)	5,901 (97.7%)	
**Speed limit**, n (%)				<0.001
Rural (≥ 40 mph)	27,395 (21.5%)	123 (0.5%)	27,272 (99.6%)	
Urban (20–30 mph)	100,242 (78.5%)	6,240 (6.2%)	94,002 (93.8%)	
**Crash time (h)**, n (%)				<0.001
Midnight (00:00–06:00)	4,810 (3.8%)	113 (2.4%)	4,697 (97.7%)	

Formatted: Right: 0.25"

Rush hours (07:00–08:00/17:00–18:00)	41,619 (32.6%)	2,056 (4.9%)	39,563 (95.1%)	
Nonrush hours (09:00–16:00)	61,696 (48.3%)	3,363 (5.5%)	58,333 (94.6%)	
Evening (19:00–23:00)	19,512 (15.3%)	831 (4.3%)	18,681 (95.7%)	
**Crash day**, n (%)				<0.001
Weekend	28,730 (22.5%)	1,072 (3.7%)	27,658 (96.3%)	
Weekday	98,907 (77.5%)	5,291 (5.4%)	93,616 (94.7%)	
**Cyclist's age (years)**, n (%)				<0.001
≤18	51,193 (40.1%)	802 (1.6%)	50,391 (98.4%)	
19–40	45,760 (35.9%)	3,474 (7.6%)	42,286 (93.4%)	
41–64	26,052 (20.4%)	1,773 (6.8%)	24,279 (93.2%)	
≥65	4,632 (3.6%)	314 (6.8%)	4,318 (93.2%)	
**Cyclist's sex**, n (%)				<0.001
Male	103,766 (81.3%)	4,404 (4.2%)	99,362 (95.8%)	
Female	23,871 (18.7%)	1,959 (8.2%)	21,912 (91.8%)	
**Crash partner**, n (%)				<0.001
Taxi/Private hire car	2,588 (2.0%)	273 (10.6%)	2,315 (89.5%)	
Car	106,668 (83.6%)	5,514 (5.2%)	101,154 (94.8%)	
Bus/Heavy goods vehicle	18,381 (14.4%)	576 (3.1%)	17,805 (96.9%)	
**Crash partner's age (years)**, n (%)				<0.001
≤18	2,415 (1.9%)	1,62 (5.2%)	2,253 (93.3%)	
19–40	49,103 (38.5%)	2,585 (5.3%)	46,518 (94.7%)	
41–64	35,598 (27.9%)	1,887 (5.3%)	33,711 (94.7%)	
≥65	40,521 (31.8%)	1,729 (4.3%)	38,792 (95.7%)	
**Crash partner's sex**, n (%)				<0.001
Male	97,447 (76.6%)	4,123 (4.2%)	93,324 (95.8%)	
Female	30,190 (23.7%)	2,240 (7.4%)	27,950 (92.6%)	

Risk factors for the three crash types

Table 4 presents the logistic regression model results. Regarding overtaking crashes, the identified risk factors included daylight conditions (adjusted odds ratio [AOR] = 1.233, 95% confidence interval [CI] = 1.162–1.309; *p*<0.001), speed limits of ≥40 mph (AOR = 2.238, 95% CI = 2.159–2.320; *p*<0.001), nonrush hours (AOR = 1.091, 95% CI 1.031–1.154; *p*=0.003), cyclists aged ≥65 years (AOR = 1.785, 95% CI = 1.649–1.931; *p*<0.001), female cyclists (AOR = 1.106, 95% CI = 1.062–1.153), HGVs as crash partners (AOR = 2.867, 95% CI = 2.473–3.323; *p*<0.001), elderly crash partners (AOR = 2.013, 95% CI = 1.937–2.092; *p*<0.001), and male crash partners (AOR = 1.353, 95% CI = 1.292–1.416; *p*<0.001).

For rear-end crashes, noteworthy risk factors included unlit darkness (AOR = 1.486, 95% CI

Formatted: Font color: Text 1

Formatted: Right: 0.25"

= 1.404–1.573; p<0.001), speed limits of ≥40 mph (AOR = 1.315, 95% CI = 1.277–1.354; p<0.001), weekdays (AOR = 1.090, 95% CI = 1.059–1.122; p<0.001), midnight hours (AOR = 1.269, 95% CI = 1.190–1.354; p<0.001), and taxis as crash partners (AOR = 1.286, 95% CI = 1.186–1.394; p<0.001).

Formatted: Font color: Text 1

Regarding door crashes, significant risk factors included lit darkness (AOR = 1.373, 95% CI = 1.141–1.651; p<0.001), speed limits of 20–30 mph (AOR = 16.185, 95% CI = 13.514–19.382; p<0.001), weekdays (AOR = 1.246, 95% CI = 1.162–1.336; p<0.001), and nonrush hours (AOR = 2.912, 95% CI = 2.384–3.556; p<0.001). Additionally, female cyclists (AOR = 1.675, 95% CI = 1.582–1.774; p<0.001), taxis or private hire cars as crash partners (AOR = 2.695, 95% CI = 2.310–3.145; p<0.001), male crash partners (AOR = 1.373, 95% CI = 1.296–1.455; p<0.001), and crash partners aged 41–64 years (AOR = 1.855, 95% CI = 1.625–2.117; p<0.001) were associated with door crashes.

Formatted: Justified

Table 4. Multivariate logistic regression results

Variable	Overtaking crashes		Rear-end crashes		Door crashes	
	**AOR (95% CI)**	***p* value**	**AOR (95% CI)**	***p* value**	**AOR (95% CI)**	***p* value**
**Light condition**						
Daylight	1.233 (1.162, 1.309)	<0.001	Ref		1.146 (0.958, 1.370)	0.137
Darkness-lit	Ref		1.042 (1.002, 1.085)	0.041	1.373 (1.141, 1.651)	0.001
Darkness-unlit	1.152 (1.059, 1.253)	0.001	1.486 (1.404, 1.573)	<0.001	Ref	
**Speed limit**						
Rural (≥40 mph)	2.238 (2.159, 2.320)	<0.001	1.315 (1.277, 1.354)	<0.001	Ref	
Urban (20–30 mph)	Ref		Ref		16.185 (13.514, 19.382)	<0.001
**Crash time**						
Midnight	1.073 (0.982, 1.173)	0.119	1.269 (1.190, 1.354)	<0.001	Ref	
Rush hours	1.059 (1.002, 1.120)	0.043	1.108 (1.078, 1.139)	<0.001	2.502 (2.051, 3.052)	<0.001
Nonrush hours	1.091 (1.031, 1.154)	0.003	Ref		2.912 (2.384, 3.556)	<0.001
Evening	Ref		0.992 (0.953, 1.032)	0.686	2.014 (1.646, 2.465)	<0.001

Formatted: Right: 0.25"

Table 4. Multivariate logistic regression results *(continued)*

Variable	Overtaking crashes		Rear-end crashes		Door crashes	
	AOR (95% CI)	*p* value	AOR (95% CI)	*p* value	AOR (95% CI)	*p* value
**Crash day**						
Weekend	1.031 (0.991, 1.072)	0.132	Ref		Ref	
Weekday	Ref		1.090 (1.059, 1.122)	<0.001	1.246 (1.162, 1.336)	<0.001
**Cyclist's age (years)**						
≤18	Ref		Ref		Ref	
19–40	1.292 (1.242, 1.345)	<0.001	1.839 (1.788, 1.891)	<0.001	5.943 (5.489, 6.435)	<0.001
41–64	1.509 (1.444, 1.578)	<0.001	1.731 (1.676, 1.789)	<0.001	6.129 (5.621, 6.684)	<0.001
≥65	1.785 (1.649, 1.931)	<0.001	1.671 (1.568, 1.780)	<0.001	5.988 (5.217, 6.874)	<0.001
**Cyclist's sex**						
Male	Ref		1.172 (1.137, 1.208)	<0.001	Ref	
Female	1.106 (1.062, 1.153)	<0.001	Ref		1.675 (1.582, 1.774)	<0.001
**Crash partner**						
Taxi/Private hire car	Ref		1.286 (1.186, 1.394)	<0.001	2.695 (2.310, 3.145)	<0.001
Car	1.571 (1.359, 1.816)	<0.001	Ref		2.089 (1.908, 2.286)	<0.001
Bus/Heavy goods vehicle	2.867 (2.473, 3.323)	<0.001	1.099 (1.061, 1.139)	<0.001	Ref	
**Crash partner's age (years)**						
≤18	1.097 (0.963, 1.249)	0.162	1.225 (1.188, 1.263)	<0.001	1.507 (1.313, 1.731)	<0.001
19–40	Ref		1.038 (1.008, 1.069)	0.013	1.855 (1.625, 2.117)	<0.001
41–64	0.950 (0.909, 0.994)	0.025	Ref		1.801 (1.574, 2.060)	<0.001
≥65	2.013 (1.937, 2.092)	<0.001	1.241 (1.137, 1.355)	<0.001	Ref	
**Crash partner's sex**						
Male	1.353 (1.292, 1.416)	<0.001	1.150 (1.117, 1.185)	<0.001	1.373 (1.296, 1.455)	<0.001
Female	Ref		Ref		Ref	

Figure 2 presents a forest plot demonstrating the joint effects of several variables on the three crash types when other variables were controlled for. The results identified several key risk factors for both overtaking and rear-end crashes. The risk of overtaking crashes showed a significant increase of 193% in rural areas when elderly drivers were involved (AOR = 2.93, 95% CI = 2.79–3.08), and similarly when heavy goods vehicles (HGVs) were the crash partner (AOR = 2.62, 95% CI = 2.46–2.78). Elderly cyclists also faced a higher risk of overtaking crashes on weekends (AOR = 1.56, 95% CI = 1.34–1.81).

Regarding rear-end crashes, the risk increased notably with unlit darkness during midnight (AOR = 1.68, 95% CI = 1.48–1.90) and was significantly higher in rural areas (AOR = 2.15,

Formatted: Font color: Text 1

Formatted: Right: 0.25"

95% CI = 2.01–2.31). Furthermore, bicycling at midnight in rural areas was associated with an increased risk of rear-end crashes (AOR = 1.68; 95% CI = 1.51–1.86). In urban settings, the risk of door crashes was higher for female cyclists (AOR = 2.29; 95% CI = 2.17–2.43) and for elderly cyclists (AOR = 2.06; 95% CI = 1.82–2.34). Finally, female cyclists exhibited a 112% higher likelihood of door crashes when the crash partner was a taxi (AOR = 2.12; 95% CI = 1.68–2.69).

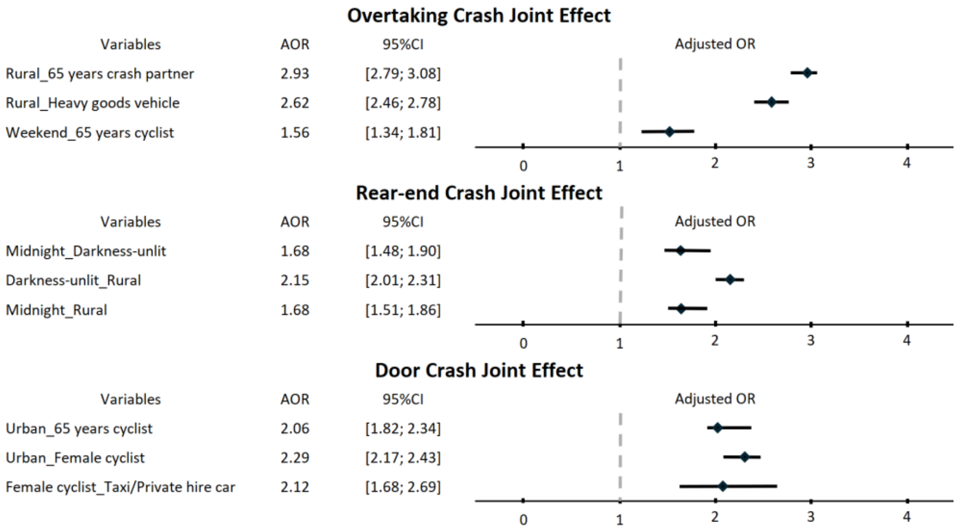

Figure. 3. Joint effects of several variables on the three crash types.

Discussion

This study explored the relationships among individual and environmental factors in relation to three common bicycle crash types (overtaking, rear-end, and door crashes) on roads in the United Kingdom from 1991 to 2020. The findings revealed several significant factors. First, for overtaking crashes, HGVs as crash partners, rural areas, and the involvement of elderly crash partners

Formatted: Right: 0.25"

emerged as key contributing factors. Second, unlit darkness, midnight hours, and rural areas were the factors most closely associated with rear-end crashes. Third, urban areas and taxis as crash partners significantly increased the likelihood of door crashes. Moreover, male crash partners were found to be a consistent risk factor across all three crash types.

Our research findings identified specific risk factors for overtaking crashes, namely rural areas, HGVs as crash partners, and elderly crash partners. These findings align with previous research that identified elderly drivers [24], speeds exceeding 10 mph, and the presence of pick-up trucks as factors contributing to increased risk for overtaking crash. Specifically, HGVs possess several characteristics that amplify this danger. Their large blind spots make it difficult for drivers to see cyclists, increasing the likelihood of crashes during overtaking. Additionally, HGVs are less ~~maneuverable~~manoeuvrable compared to passenger cars, which reduces their ability to avoid crashes if cyclists suddenly enter their path. The speed and distance perception issues between HGVs and cyclists further complicate the judgment of safe overtaking gaps. Furthermore, HGVs require longer stopping distances due to their size and weight, which can lead to severe consequences if a sudden need to brake arises. A behavioural study suggested that compared with cars, HGVs tended to maintain a narrower clearance zone when overtaking bicycles [25]. Regarding the association with buses or HGVs, Pai et al. [18] ~~suggests~~ suggested that time pressures on HGV drivers for timely loading and unloading might lead to more reckless driving. Specifically, our results align with the observations made by Pai et al., who also mentioned higher crash rates involving buses or HGVs, supporting the idea that these time pressures contribute to increased crash risks. Our findings underscore the necessity of implementing measures such as 'Share the Road' warning signs [26], particularly in rural settings, where HGVs are likely to execute

Formatted: Font color: Text 1

Formatted: Font color: Text 1

Formatted: Font color: Text 1

Formatted: Font color: Text 1

Formatted: Font color: Text 1

Formatted: Font color: Text 1

Formatted: Right: 0.25"

overtaking manoeuvres at high speed. Such measures could prompt motor vehicles to maintain safer distances from the edges of travel lanes, especially in areas with a notable presence of both HGVs and bicycles.

We also identified elderly drivers as a factor contributing to overtaking crashes—a finding consistent with relevant research [24]. We found that as individuals age, their risk of being involved in road accidents increases, primarily due to declines in cognitive capabilities. Our study corroborates these findings by showing that older cyclists are more susceptible to accidents during overtaking ~~maneuvers~~manoeuvres, which can be attributed to diminished reaction times and impaired decision-making abilities [27], their health [28], and their driving performance [29]. Notably, crashes involving elderly individuals often occur in scenarios with challenging conditions, including at intersections without traffic control measures, on high-speed roads, during adverse weather conditions, in poorly lit areas, and in head-on accidents [30-32]. The heightened level of risk under such conditions may be attributed to cognitive and perceptual decline in older drivers, which could affect their capacity to execute actions such as overtaking manoeuvres safely. Accordingly, developing specialised cognitive training programmes as interventions to enhance road safety for elderly drivers is evidently necessary [33]. Based on our study's findings, we recommend the development of ~~specialized~~ specialised interventions to improve road safety for elderly cyclists. Our analysis reveals that older cyclists are at a higher risk of being involved in overtaking crashes, with this increased risk being strongly linked to declines in cognitive capabilities associated with aging. To address this issue, we advocate for the implementation of targeted cognitive training programs specifically designed for elderly cyclists. These programs should focus on enhancing critical skills such as reaction time, situational awareness, and

Formatted: Font color: Text 1

Formatted: Font color: Text 1

Formatted: Font color: Text 1

Formatted: Font color: Text 1

Formatted: Right: 0.25"

decision-making abilities, which are crucial for reducing crash risk and improving overall road safety.

In the present study, several factors were found to increase the risk of rear-end crashes on road segments, including darkness with unlit surroundings, midnight hours, and rural settings (speed limit > 40 mph). Although few studies have specifically addressed rear-end crashes involving bicycles on road segments, available data suggest that the low conspicuity of bicycles, especially at night, is a recurrent factor in rear-end crashes [18, 34]. Moreover, a lack of adequate street lighting, which is common in rural settings, predisposes cyclists to rear-end crashes [18]. Our joint-effects analysis further indicated that the detrimental effect of unlit darkness is more pronounced in rural areas and during midnight hours. Potential intervention strategies to mitigate rear-end crashes include enhancing illumination and executing speed control management on rural road segments with heavy bicycle traffic.

Next, our analysis successfully identified associations of urban areas and taxis and private hire cars as crash partners with door crashes on road segments. Although research specifically focusing on door crashes on road segments is limited, similar findings were documented by Pai, indicating that urban roadways and taxis contributed to door crashes [18]. However, determining the factors influencing this trend poses a challenge. One possible explanation could be the increased presence of taxis or private hire cars in such areas, where passengers often disembark. Additionally, our analysis further revealed an elevated risk of door crashes involving crashes with taxis in urban areas. To reduce door crashes on road segments, educating taxi drivers, as well as passengers, about the importance of vigilance when opening doors near traffic is essential [18]. In addition, cyclists should be advised to maintain at least a door’s width distance from all parked

Formatted: Right: 0.25"

cars to improve the sight triangles of drivers and increase the visibility of cyclists [35]. Implementing a two-stage door opening mechanism for vehicles, which would enable drivers to verify the presence of bicycles to the rear, could also be beneficial [36].

The strengths of this study include the use of STATS19 datasets spanning from 1991 to 2020, which provides a robust statistical foundation and a broad perspective on trends in bicycle crashes. By focusing specifically on three crash types on road segments—overtaking, rear-end, and door crashes—the study provides a comprehensive and focused analysis, which can yield more actionable insights and more effective recommendations. The UK-based dataset ensures that the findings are particularly relevant for local policy and safety interventions. Additionally, the application of statistical techniques and the consideration of various factors, such as crash partner and time of day, enhance the validity and depth of the analysis.

Formatted: Font color: Text 1

This study had several limitations that warrant acknowledgement. First, the substantial underreporting of nonfatal casualties to the police, particularly casualties involving cyclists not obligated to report accidents, is a critical factor to consider. Such underreporting, as highlighted by the U.K. Government's Department for Transport [11], likely results in the incomplete representation of nonfatal and 'slight' casualties in road casualty data. Second, the STATS19 data utilised in this study lack critical variables, including precrash speeds, specific geometric characteristics of roadways, data regarding alcohol and illicit substance use, and cyclist speed at the time of an accident. Moreover, critical exposure data—such as those related to traffic flow, rider or driver experience, and other elements of risk exposure—are absent, and the absence of such details limits our ability to fully account for potential variations resulting from unobserved factors in the analyses. Finally, this study did not explore annual trends in each type of bicycle

Formatted: Right: 0.25"

crash over the 30-year study period; investigating such trends could provide insights regarding changing behaviours among cyclists and motor vehicle drivers as well as the effects of legislative changes for road speed limits.

One inherent problem with police-reported crash data is the variables not readily available, hereby causing unobserved heterogeneity across the observations. To overcome such a limitation, we estimated separate regression models, as suggested by Kim et al. [37], for the three crash types; such an approach provides greater explanatory power compared to single overall models. Further, we conducted joint-effect analyses of several variables of interest that capture heterogeneity. In our previous studies, we adopted the above-mentioned approaches to overcome the inherent problem with a success [38, 39].

Future research directions could involve integrating GPS (Global Positioning System) data and weather conditions to analyse both ~~the~~ injury frequency and fatalities of bicycle crashes on road segments. Additionally, exploring the potential of autonomous vehicles for detecting approaching bicycles for door-crashes and implementing AI-controlled lighting systems in rural areas for cyclist detection could be promising areas for further study.

Recommendations

For overtaking crashes, we recommend implementing 'Share the Road' warning signs, especially in rural areas, and developing specialized cognitive training programs for elderly drivers. Regarding rear-end crashes, our suggestions include improving illumination during night time and implementing speed control measures on rural road segments. For door crashes involving parked cars, we propose enhancing driver sight triangles and increasing cyclist visibility.

Formatted: Font color: Text 1

Formatted: Font color: Text 1

Formatted: Font color: Text 1

Formatted: Font color: Text 1

Formatted: Font color: Text 1

Formatted: Font color: Text 1

Formatted: Right: 0.25"

Moreover, implementing a two-stage door opening mechanism and an automatic detection device in vehicles to alert drivers of bicycles approaching from behind could potentially be beneficial.

Conclusions

This study identified several significant risk factors for the three predominate types of crashes involving cyclists on road segments: HGVs as crash partners, elderly crash partners, and rural areas for overtaking crashes; unlit darkness, midnight hours, and rural areas for rear-end crashes; and urban areas and taxis as crash partners for door crashes. These risk factors remained unchanged since our previous study conducted in 2011 [15]. The present research enhances the field of bicycle safety research by concluding that the detrimental effects of certain variables become more pronounced under certain conditions. For example, first, cyclists in rural settings exhibited an elevated risk of overtaking crashes involving HGVs. Second, the rear-end crash risk increases in the combined presence of unlit darkness, midnight hours, and rural areas. Finally, in urban settings, the likelihood of door crashes increases when a taxi is the crash partner.

Abbreviations

WHO: World Health Organization; HGVs: heavy goods vehicles; AOR: adjusted odds ratio; CI: confidence interval.

Acknowledgments

This manuscript was edited by Wallace Academic Editing.

Formatted: Right: 0.25"

Author contributions

Literature review: Chun-Chieh Chao.

Methodology: Chun-Chieh Chao, Chih-Wei Pai.

Data merging and analysis: Akhmad Fajri Widodo-, Wafaa Saleh, Bayu Satria Wiratama.

Writing - original draft: Chun-Chieh Chao.

Writing – review and editing: Hui-An Lin, Chenyi Chen, Hon-Ping Ma, Akhmad Fajri Widodo.

Validation: Chun-Chieh Chao, Hui-An Lin, Chenyi Chen, Hon-Ping Ma, Shih Yu Ko.

Supervision: Li Wei, Yen-Nung Lin, Shou-Chien Hsu, Chih-Wei Pai.

Funding: Cheng-Wei Chan, Chih-Wei Pai.

Funding

This study received financial support from the Ministry of Science and Technology, Taiwan (MOST 110–2410-H-038-016-MY2 and MOST 109–2314-B-038-066-); New Taipei City Hospital (NTPC113–002); and the National Science and Technology Council, Taiwan (NSTC 112-2410-H-038-016-MY2). The funders played no role in the design of the study, data collection and analysis, interpretation of data, or preparation of the manuscript.

Availability of data and materials

This study utilised the British STATS19 database, which contains data on all road traffic accidents in the United Kingdom. The data that support the findings of this study are openly available at

Formatted: Right: 0.25"

https://figshare.com/ndownloader/files/48173452~~https://www.data.gov.uk/dataset/cb7ae6f0-4be6-4935-9277-47e5ce24a11f/road-safety-data~~.

Declarations

Ethical approval and consent to participate

This study was conducted in accordance with the Declaration of Helsinki and approved by the Joint Institutional Review Board of Taipei Medical University (N202011030).

Consent for publication

This study was approved by the Joint Institutional Review Board of Taipei Medical University (N202011030). The Joint Institutional Review Board of Taipei Medical University has waived the requirement of informed consent. All methods were performed in accordance with the relevant guidelines and regulations of the Declaration of Helsinki.

Competing interests

The authors declare that they have no competing interests in relation to this work.

Author information

[1]Graduate Institute of Injury Prevention and Control, College of Public Health, Taipei Medical University, Taipei City, Taiwan. [2]Department of Emergency Medicine, Taipei Medical University Hospital, Taipei City, Taiwan. [3]Department of Emergency Medicine, School of Medicine, College of Medicine, Taipei Medical University, Taipei City, Taiwan. [4]Department of Emergency Medicine,

Formatted: Right: 0.25"

New Taipei City Hospital, New Taipei City, Taiwan. [5]College of Medicine, Chang Gung University, Taoyuan City, Taiwan. [6]Department of Emergency Medicine, Chang Gung Memorial Hospital, Linkou branch, Taoyuan, Taiwan.[7] Department of Emergency Medicine, Taipei Medical University-Shuang Ho Hospital, New Taipei City, Taiwan. [8]Taipei Neuroscience Institute, Taipei Medical University, Taipei City, Taiwan. [9]Division of Neurosurgery, Department of Surgery, Wan Fang Hospital, Taipei Medical University, Taipei City, Taiwan. [10]Department of Physical Medicine and Rehabilitation, Wan Fang Hospital, Taipei Medical University, Taipei City, Taiwan. [11]Transport Research Institute, Edinburgh Napier University, Edinburgh, Scotland. [12]Department of Epidemiology, Biostatistics, and Population Health, Faculty of Medicine, Public Health and Nursing, Universitas Gadjah Mada, Yogyakarta City, Indonesia, [13]Department of Occupational Medicine, Chang Gung Memorial Hospital, Linkou branch, Taoyuan, Taiwan.

Formatted: Right: 0.25"

References:

1. Kjeldgard L, Ohlin M, Elrud R, Stigson H, Alexanderson K, Friberg E. Bicycle crashes and sickness absence - a population-based Swedish register study of all individuals of working ages. BMC Public Health. 2019;19(1):943.
2. World Health Organization. Regional Office for E. Walking and cycling: latest evidence to support policy-making and practice. Copenhagen: World Health Organization. Regional Office for Europe; 2022.
3. Venkatraman V, Richard CM, Magee K, Johnson K. Countermeasures that work: A highway safety countermeasure guide for state highway safety offices, 10th Edition, 2020. (DOT HS 813 097).
4. Allen-Munley C, Daniel J, Dhar S. Logistic model for rating urban bicycle route safety. Transportation Research Record. 2004;1878(1):107-15.
5. Kaplan JA. Characteristics of the Regular Adult Bicycle User. Final Report 1975.
6. Rivara FP, Thompson DC, Thompson RS. Epidemiology of bicycle injuries and risk factors for serious injury. Inj Prev. 1997;3(2):110-4.
7. Wanvik PO. Effects of road lighting: an analysis based on Dutch accident statistics 1987-2006. Accid Anal Prev. 2009;41(1):123-8.
8. Elvik R, Sundfør HB. How can cyclist injuries be included in health impact economic assessments? Journal of Transport & Health. 2017;6:29-39.
9. Aertsens J, de Geus B, Vandenbulcke G, Degraeuwe B, Broekx S, De Nocker L, et al. Commuting by bike in Belgium, the costs of minor accidents. Accident Analysis & Prevention. 2010;42(6):2149-57.
10. Scholten AC, Polinder S, Panneman MJ, Van Beeck EF, Haagsma JA. Incidence and costs of bicycle-related traumatic brain injuries in the Netherlands. Accident Analysis & Prevention. 2015;81:51-60.
11. Traffic Safety Facts - Bicyclists and Other Cyclists. https://crashstats.nhtsa.dot.gov/Api/Public/ViewPublication/813322: National Highway Traffic Safety Administration; 2020.
12. Bil M, Bilova M, Muller I. Critical factors in fatal collisions of adult cyclists with automobiles. Accid Anal Prev. 2010;42(6):1632-6.
13. Moore DN, Schneider WHt, Savolainen PT, Farzaneh M. Mixed logit analysis of bicyclist injury severity resulting from motor vehicle crashes at intersection and non-intersection locations. Accid Anal Prev. 2011;43(3):621-30.
14. Wachtel A, Lewiston D. Risk factors for bicycle-motor vehicle collisions at intersections. ITE journal. 1994;64(9):30-5.
15. Ugan J, Abdel-Aty M, Cai Q, Mahmoud N, Al-Omari Me. Effect of various speed management strategies on bicycle crashes for urban roads in Central Florida. Transportation Research Record: Journal of the Transportation Research Board. 2021;2676:036119812110366.
16. Meuleners LB, Fraser M, Johnson M, Stevenson M, Rose G, Oxley J. Characteristics of the road infrastructure and injurious cyclist crashes resulting in a hospitalisation. Accident Analysis & Prevention. 2020;136:105407.
17. Robartes E, Chen T-D. The effect of crash characteristics on cyclist injuries: An analysis of Virginia automobile-bicycle crash data. Accid Anal Prev. 2017;104:165-73.

Formatted: Right: 0.25"

18. Pai C-W. Overtaking, rear-end, and door crashes involving bicycles: an empirical investigation. Accid Anal Prev. 2011;43(3):1228-35.
19. Debnath AK, Haworth N, Schramm A, Heesch KC, Somoray K. Factors influencing noncompliance with bicycle passing distance laws. Accid Anal Prev. 2018;115:137-42.
20. Vandenbroucke JP, von Elm E, Altman DG, Gotzsche PC, Mulrow CD, Pocock SJ, et al. Strengthening the Reporting of Observational Studies in Epidemiology (STROBE): explanation and elaboration. PLoS Medicine. 2007;4(10):1628-55.
21. Watson A, Watson B, Vallmuur K. Estimating under-reporting of road crash injuries to police using multiple linked data collections. Accident Analysis & Prevention. 2015;83:18-25.
22. Maldonado G, Greenland S. Simulation study of confounder-selection strategies. American ~~journal~~ Journal of ~~epidemiology~~Epidemiology. 1993;138(11):923-36.
23. Chen P-L, Pai C-W. Evaluation of injuries sustained by motorcyclists in approach-turn crashes in Taiwan. Accident Analysis & Prevention. 2019;124:33-9.
24. Liu J, Jones S, Adanu EK, Li X. Behavioral pathways in bicycle-motor vehicle crashes: From contributing factors, pre-crash actions, to injury severities. J Safety Res. 2021;77:229-40.
25. Walker I. Signals are informative but slow down responses when drivers meet bicyclists at road junctions. Accid Anal Prev. 2005;37(6):1074-85.
26. Kay JJ, Savolainen PT, Gates TJ, Datta TK. Driver behavior during bicycle passing maneuvers in response to a Share the Road sign treatment. Accid Anal Prev. 2014;70:92-9.
27. Anstey KJ, Horswill MS, Wood JM, Hatherly C. The role of cognitive and visual abilities as predictors in the Multifactorial Model of Driving Safety. Accid Anal Prev. 2012;45:766-74.
28. Kandasamy D, Betz ME, DiGuiseppi C, Mielenz TJ, Eby DW, Molnar LJ, et al. Self-reported health conditions and related driving reduction in older drivers. Occup Ther Health Care. 2018;32(4):363-79.
29. Laosee O, Rattanapan C, Somrongthong R. Physical and cognitive functions affecting road traffic injuries among senior drivers. Arch Gerontol Geriatr. 2018;78:160-4.
30. Cicchino JB, Wells JK, McCartt AT. Survey about pedestrian safety and attitudes toward automated traffic enforcement in Washington, D.C. Traffic Inj Prev. 2014;15(4):414-23.
31. Kostyniuk LP, Molnar LJ. Self-regulatory driving practices among older adults: health, age and sex effects. Accid Anal Prev. 2008;40(4):1576-80.
32. Zhang J, Lindsay J, Clarke K, Robbins G, Mao Y. Factors affecting the severity of motor vehicle traffic crashes involving elderly drivers in Ontario. Accid Anal Prev. 2000;32(1):117-25.
33. Edwards JD, Ross LA, Wadley VG, Clay OJ, Crowe M, Roenker DL, et al. The useful field of view test: normative data for older adults. Arch Clin Neuropsychol. 2006;21(4):275-86.
34. Wood JM, Lacherez PF, Marszalek RP, King MJ. Drivers' and cyclists' experiences of sharing the road: incidents, attitudes and perceptions of visibility. Accid Anal Prev. 2009;41(4):772-6.
35. Hunter W, Stewart JR. An evaluation of bike lanes adjacent to motor vehicle parking. 1999.
36. Huang C-Y. Observations of drivers' behavior when opening car doors. Procedia Manufacturing. 2015;3:2753-60.
37. Kim D-G, Washington S, Oh J. Modeling crash types: New insights into the effects of covariates on crashes at rural intersections. Journal of Transportation Engineering. 2006;132(4):282-92.
38. Pai C-W, Saleh W. Modelling motorcyclist injury severity by various crash types at T-junctions in the UK. Safety Science. 2008; 13:89-98.

Formatted: Right: 0.25"

39. Pai C-W, Jou R-C. Cyclists' red-light running behaviours: An examination of risk-taking, opportunistic, and law-obeying behaviours. Accident Analysis & Prevention. 2014;62:191-8.

Formatted: Right: 0.25"

Dear Editors and Reviewers,

We greatly appreciate the valuable comments and suggestions raised by reviewers. Please very kindly see our responses below, as well as the revised manuscript. We would be glad if you could have our manuscript reviewed again.

Best regards,

Chih-Wei Pai (Prof)

Graduate Institute of Injury Prevention and Control College of Public Health

Taipei Medical University

Reviewer comments:

Reviewer 1: Regarding the statistical analysis, I would like to ask the authors to explain:
1. the reason(s) for ignoring any probable interaction between independent variables in the multivariate logistic regression.

Author's response: We appreciate the reviewer's comment and question. By examining variables independently, we gain a clearer understanding of their individual impacts on the outcome (specifically, crash type in this study). This approach allows us to assess each variable's direct influence without the added complexity of interactions or modifications between variables. It provides insights into which variables independently affect the outcome, directly addressing our research questions. Initially, we used the chi-squared test to explore associations between a set of independent variables and the three crash types. To minimize type II errors in variable selection and ensure unbiased inferences, we included variables with a p-value less than 0.2 from the univariate analysis into the multivariate logistic regression models, a common practice in past studies of traffic injuries (e.g., a, b) and methodology (c). Subsequently, we examined interaction effects among several variables of interest, as depicted in Figure 2 of the manuscript. While acknowledging the potential for other interactions among variables, our study focused on assessing the joint effects of specific variables of interest. To take overtaking crashes as an example, these variables included rural areas, crash partners aged 65 years or older, heavy goods vehicles, weekends, and cyclists aged 65 years or older. Future research could delve into untangling the complexities of additional interaction effects among variables, as suggested by the reviewer.

References:

a: Chen, P-L, Pai, C-W. Evaluation of injuries sustained by motorcyclists in approach-turn crashes in Taiwan. Accident Analysis and Prevention, 2019, 124, 33-39.

b: Chien, D-K., Hwang, HF, Lin, MR. Injury severity measures for predicting return-to-work after a traumatic brain injury. Accident Analysis and Prevention, 2017, 98, 101-107.

c: Maldonado G, Greenland S. Simulation study of confounder-selection strategies. Am J Epidemiol 1993, 138, 11, 923-936.

2. Why did they consider different reference categories for the same individual variables among different outcomes in logistic regression modeling? This will make it difficult to interpret the comparison of the effect of an independent variable on different types of crashes. for example, in table 4, the ref category for Light condition is

Darkness-lit, Daylight and Darkness-unlit for Overtaking, Rear-end and Door crashes respectively.

Author's response: We appreciate the reviewer's comment and question. In our analysis, we chose various reference categories for variables based on the lowest Adjusted Odds Ratios (AORs) observed. This approach allowed us to highlight different risk factors associated with higher AORs for specific types of crashes. For example, urban roads with speed limits of 20-30 mph were identified as protective factors for overtaking and rear-end crashes. However, for door crashes, these urban roads appeared to pose a higher risk compared to rural roads, as indicated by their higher AOR. It is important to note that selecting a reference category does not change the estimation results of our models. Instead, assigning reference case with the lowest AOR helps readers identify risk factors with higher AORs among the three crash types.

3. I suggest authors provide identical indicators for figures both in the main text and in the figure's caption. Reading "Fig. 1" below a figure, one will look for the same word in the main text while it is recalled as "Figure 1".

Author's response: We appreciate this reviewer's comments, and we have revised the manuscript in the main text and figure's caption (please refer to lines 145 to 146; page 8 in the manuscript).

Reviewer 2:
1 General comments:
1.1 None of the authors was from the UK???

Author's response: We appreciate this reviewer's comments. One of our authors, Prof. Wafaa Saleh, is from Edinburgh Napier University, UK.

1.2 The authors should emphasize the significance of including these three types of crashes????

Author's response: We appreciate the reviewer's comments. We have incorporated the following statements into the introduction to underscore the significance of including the three crash types (please refer to lines 110 to 115; pages 5-6 in the manuscript):

> "The study addresses a critical gap in current research, focusing on crashes specifically occurring on road segments. Existing literature offers limited insights into this specific type of crash, highlighting a crucial need for targeted investigation. These crashes have the potential for severe impact, involving complex dynamics that demand a nuanced understanding for effective mitigation strategies. By exploring these factors, our research aims to significantly enhance cyclist safety within this particular context."

1.3 What novelty this study adds compared to the previous one in 2011???

Author's response:

We appreciate this reviewer's comment. One inherent problem with police-reported crash data is the variables not readily available, hereby causing unobserved heterogeneity across the observations. To overcome such a limitation, we estimated separate regression models, as suggested by Kim et al. (e.g., d), for the three crash types; such an approach provides greater explanatory power compared to single overall models. Further, we conducted joint-effect analyses of several variables of interest that capture heterogeneity. In our previous studies, we adopted the above-mentioned approaches to overcome the inherent problem with a success (e.g., e, f).

To clarify this, the following statements have been added to the Discussion section of the manuscript (please refer to lines 391 to 397; page 23 in the manuscript):

> "One inherent problem with police-reported crash data is the variables not readily available, hereby causing unobserved heterogeneity across the observations. To overcome such a limitation, we estimated separate regression models, as

suggested by Kim et al. (e.g., d), for the three crash types; such an approach provides greater explanatory power compared to single overall models. Further, we conducted joint-effect analyses of several variables of interest that capture heterogeneity. In our previous studies, we adopted the above-mentioned approaches to overcome the inherent problem with a success (e.g., e, f)."

d: Kim, D., Washington, S., Oh, J., 2006. Modelling crash outcomes: new insights into the effects of covariates on crashes at rural intersections. Journal of Transportation Engineering. 132 (4), 282-292.

e: Pai CW, Jou RC, 2014. Cyclists' red-light running behaviours: An examination of risk-taking, opportunistic, and law-obeying behaviours. Accident Analysis and Prevention. 62,191-198.

f: Pai CW, Saleh W., 2008. Modelling motorcyclist injury severity by various crash types at T-junctions in the UK. Safety Science. 13, 98-98.

1.4 The rationale for conducting the current study as well as the practical implications should be emphasized??

<u>Author's response:</u> We appreciate this reviewer's comments. First, regarding the rationale for conducting the current study, we have added the following statements (please kindly refer to lines 91-95 on page 5 of the manuscript):

> "Bicycle crashes on road segments remain a substantial issue for public health concern. Existing research primarily emphasizes intersection-related crashes. This study aims to fill a critical gap by conducting a thorough examination of the risk factors associated with three distinct bicycle crash types: overtaking, rear-end, and door crashes that occur on road segments."

Secondly, to highlight the practical implications, we have included the following statements in the Discussion section (please refer to lines 404-412 on pages 23-24 of the manuscript):

> "Recommendations
>
> For overtaking crashes, we recommend implementing 'Share the Road' warning signs, especially in rural areas, and developing specialized cognitive training programs for elderly drivers. Regarding rear-end crashes, our suggestions include improving illumination during night time and implementing speed control measures on rural road segments. For door crashes involving parked cars, we propose enhancing driver sight triangles and increasing cyclist visibility. Moreover, implementing a two-stage door opening mechanism and an automatic

detection device in vehicles to alert drivers of bicycles approaching from behind could potentially be beneficial."

1.5 For the introduction section, burden in terms of mortality, morbidity, and DALYs should be mentioned as well the economic and health care costs should be mentioned (globally and UK)

Author's response: We appreciate the reviewer's comments. Our original literature review has included several past studies that have reported the accident/injury outcomes resulting from these three crash types. For example, road segments with elevated speed limits, male cyclists, and cyclists aged over 55 years contribute significantly to high injury severity crashes. Additionally, built-up areas increase the risk of door crashes involving cyclists and parked cars.

It is important to note that there is limited research specifically examining the impact of overtaking, rear-end, and door crashes on Disability-Adjusted Life Years DALYs, economic costs, and healthcare expenses. Notable exceptions include studies by Elvik and Sundfør (e.g., d), who examined the inclusion of cyclist injuries in health impact economic assessments. Aertsens et al. (e.g., h) and Scholten et al. (e.g., i) also provided comprehensive analyses of the total and average costs associated with bicycle injuries. Although the three crash types were not explicitly examined in the above-mentioned studies, we have followed this reviewer's suggestion by incorporating these studies into the 'Introduction' section (please refer to lines 77-81; page 4 of the manuscript):

> "Bicycle crashes can also impose a significant burden on healthcare expenses. Elvik and Sundfør (e.g., g) have discussed the economic implications and healthcare expenditures associated with bicycle accidents. For instance, in Belgium, the average cost of bicycle accidents per case is estimated at 841 euros (e.g., h). In the Netherlands, the total annual cost has been reported as €410.7 million (e.g., i)."

References:

g: Elvik, R., & Sundfør, H. B. (2017). How can cyclist injuries be included in health impact economic assessments? Journal of Transport & Health, 6, 29-39.
h: Aertsens, J., de Geus, B., Vandenbulcke, G., Degraeuwe, B., Broekx, S., De Nocker, L., ... & Panis, L. I. (2010). Commuting by bike in Belgium, the costs of minor accidents. Accident Analysis & Prevention, 42(6), 2149-2157.

i: Scholten, A. C., Polinder, S., Panneman, M. J., Van Beeck, E. F., & Haagsma, J. A. (2015). Incidence and costs of bicycle-related traumatic brain injuries in the Netherlands. Accident Analysis & Prevention, 81, 51-60.

1.6 The number of cyclists in UK or those using bicycles for their mobility??

<u>Author's response:</u> We appreciate the reviewer's comment. In our study, we analyzed national police-reported crash data involving cyclists. Unfortunately, exposure data, such as the number of cyclists and miles traveled, were not available in the STATS19 dataset. While such data may be available from the UK National Travel Survey, it often reflects outdated information and may not be fully representative of the entire population.

2. Specific comments:
2.1 Instead of data collection, data used for analysis is appropriate??

<u>Author's response:</u> We appreciate the reviewer's comment. The dataset, UK Stats19 covering all traffic accidents in the UK, should be appropriate, as numerous studies in the field of traffic injury and medicine have analysed such data (e.g., references j, k, l).

j: Haghpanahan, Houra, et al. "An evaluation of the effects of lowering blood alcohol concentration limits for drivers on the rates of road traffic accidents and alcohol consumption: a natural experiment." *The Lancet* 393.10169 (2019): 321-329.

k: Pai, C. W., Hwang, K. P., & Saleh, W. (2009). A mixed logit analysis of motorists' right-of-way violation in motorcycle accidents at priority T-junctions. Accident Analysis & Prevention, 41(3), 565-573.

l: Fountas, G., Fonzone, A., Gharavi, N., & Rye, T. (2020). The joint effect of weather and lighting conditions on injury severities of single-vehicle accidents. *Analytic methods in accident research*, *27*, 100124.

2.2 Of the used crashes data, how many were fatal???

<u>Author's response:</u> We appreciate the reviewer's comment. As reported in the table below, as many as 0.8% of those in overtaking crashes sustained fatal injuries, which was the highest compared to those in the other two crash types.

	Slight	Serious	Fatal	Total
Overtaking crashes	14240(77.6%)	3,964(21.6%)	147(0.8%)	18350

Rear-end crashes	39821(89.1%)	4782(10.7%)	89(0.2%)	44692
Door crashes	5561(87.4%)	770(12.1%)	32(0.5%)	6363

2.3 For analysis of data, use the Odds ratios and 95% confidence intervals (univariate and bivariate)

Author's response: We appreciate this reviewer's comment. We analyzed the distribution of crash types across a set of independent variables. Chi-square tests were used to explore relationships between these variables and crash types. Variables with a significance level below 0.2 were identified to minimize type II errors and were considered significantly associated with the outcome variables ($p < 0.05$). Subsequently, these variables were included in multiple logistic regression models. Stepwise logistic regression was then employed to estimate the odds of various variables after controlling for specific factors. This methodology has been widely used in past studies of traffic injuries (e.g., a, b) and methodology (e.g., c).

a: Chen, P-L, Pai, C-W. Evaluation of injuries sustained by motorcyclists in approach-turn crashes in Taiwan. Accident Analysis and Prevention, 2019, 124, 33-39;

b: Chien, D-K., Hwang, HF, Lin, MR. Injury severity measures for predicting return-to-work after a traumatic brain injury. Accident Analysis and Prevention, 2017, 98, 101-107;

c: Maldonado G, Greenland S. Simulation study of confounder-selection strategies. Am J Epidemiol 1993, 138, 11, 923-936).

2.4 Details about the multivariate logistic regression model should be mentioned???
Use the Odds ratios for interpreting and displaying the results in tables 1, 2, and 3???

Author's response: We appreciate the reviewer's comment. Firstly, if we understand this reviewer correctly, we have incorporated additional details (such as formulation and derivation) of the multivariate logistic regression model into the "Methods" section (please refer to lines 179-194 on pages 10-11 of the manuscript):

> "Initially, we examined the distribution of three crash types across various variables to explore their relationships with a binary outcome. These variables included lighting conditions, speed limit, time of day, and day of the week. Demographic details concerning cyclist casualties encompassed age and sex, while information about the crash partner included vehicle type, age, and sex. We set a significance level of p <

0.2 to include risk factors in our multivariate analysis. Adjusted odds ratios (AORs) were computed using multivariate logistic regression with backward selection.

The multivariate logistic regression model equation was specified as:

$$log\left(\frac{P(Y=1)}{1-P(Y=1)}\right) = \beta_0 + \beta_1 X_1 + \beta_2 X_2$$

where $P(Y=1)$ denotes the probability of the outcome, $\beta_0, \beta_1, \beta_2, \ldots, \beta_p$ are the coefficients to be estimated, and $X_1, X_2, \ldots, X_p$ represent the predictor variables.

Before estimating the model, assumptions of logistic regression, such as linearity of the logit, absence of multicollinearity, and independence of observations, were evaluated.

An odds ratio (OR) greater than 1 indicated a positive association between the independent variable and the occurrence rate, while an OR less than 1 indicated a negative association. An OR of 1 suggested no association between the variables of interest and the outcomes."

Secondly, this reviewer suggested that we should use the Odds ratios for interpreting and displaying the results in tables 1, 2, and 3. While we acknowledge this suggestion, we would like to clarify here that we adopted the commonly-used Chi-square tests to identify the distribution of three crash types across several independent variables. Instead of the univariate logistic regression, such a method has been proved as an efficient way to minimize type II errors, and has been widely employed in past studies of traffic injuries (e.g., a, b) and methodology (e.g., c).

a: Chen, P-L, Pai, C-W. Evaluation of injuries sustained by motorcyclists in approach-turn crashes in Taiwan. Accident Analysis and Prevention, 2019, 124, 33-39;

b: Chien, D-K., Hwang, HF, Lin, MR. Injury severity measures for predicting return-to-work after a traumatic brain injury. Accident Analysis and Prevention, 2017, 98, 101-107;

c: Maldonado G, Greenland S. Simulation study of confounder-selection strategies. Am J Epidemiol 1993, 138, 11, 923-936).

2.5 Chi square is not enough test to identify the direction and which segment of the given variable is significantly different???

Author's response: We appreciate this reviewer's comment. The reviewer is correct. Chi-square tests can be used for ascertaining the association of the dependent and independent variables. However, the direction of the independent variables can be untangled in the subsequent multivariate logistic regression models.

2.6 What was the adjustment made for??? And how???

Author's response: We appreciate this reviewer's comment. Each variable was adjusted for in the multivariate analysis. For instance, in Table 4, adjustments were made for crash day after accounting for other variables such as cyclist's sex, crash partner, and crash partner's age and sex.

2.7 The joint-crash effect: how it was measured statistically???

Author's response: Thank you for your valuable comment. We do apologize for not making our analysis clear. To clarify how joint-effect analysis was structured, we drew several figures below that help us respond to this reviewer.
As Figure A1 (X axis: speed limit; Y axis: percentage) and A2 report (X axis: Crash partner's age; Y axis: percentage), the joint effects of speed limit (two categories: rural (≥ 40 mph) /urban (20–30 mph)] and crash partner's age (four categories: ≤18, 19–40, 41–64, and ≥65) on overtaking crashes were examined, yielding eight combinations of interaction effects (i.e., 1. Rural x ≤18; 2. Rural x 19-40; 3. Rural x 41-64; 4. Rural x ≥65; 5. Urban x ≤18; 6. Urban x 19-40; 7. Urban x 41-64; 8. Urban x ≥65). All percentages of overtaking crashes among these eight combinations were compared, and the combination with the highest percentages for overtaking crashes is taken as the indicator variable. In this joint-effect analysis, the indicator variable "rural areas x crash partner's ≥65 years old" has the highest percentage of overtaking crashes. These results elucidated that overtaking crashes were more likely to occur when the cyclists were in rural areas and when involving ≥65-year-old crash partners.

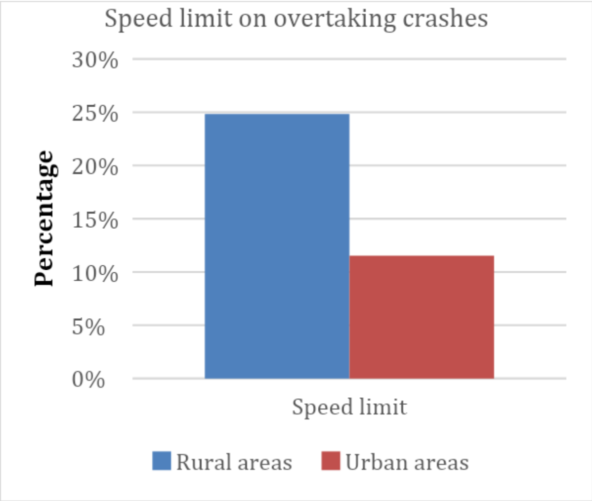

Figure A1

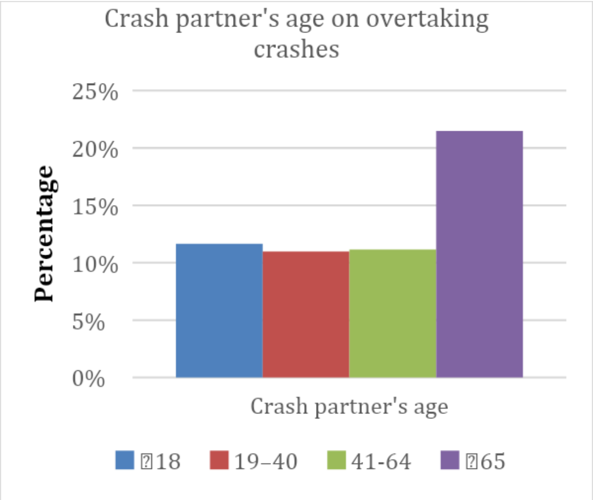

Figure A2

In practice, such a joint-effect analysis has been widely employed in medicine or traffic injury literature. One well-known paper by Weinstein et al. (i.e., m) was published in JAMA which examined the joint effect of physical activity and body mass index on diabetes in women. In this paper, Weinstein et al. pointed out that the beneficial effect of active lifestyle on type 2 diabetes was consistent across women with three BMI levels.

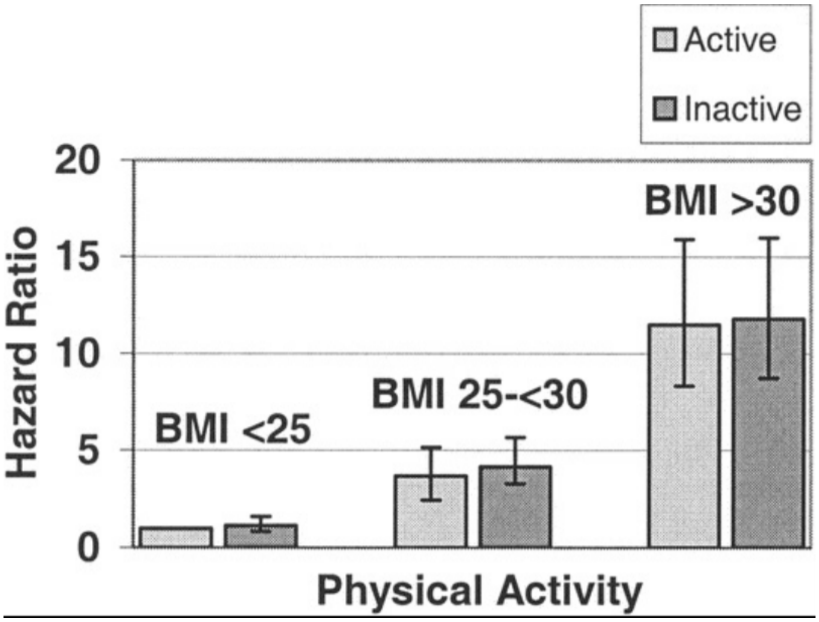

Another example is our previous paper published in Accident Analysis and Prevention in 2020 titled: Evaluating the combined effect of alcohol-involved and un-helmeted riding on motorcyclist fatalities in Taiwan. In this study, we specifically analysed the joint effect of alcohol use and helmet use on motorcyclist fatalities (i.e., n).

Interaction results for motorcyclist's alcohol use with motorcyclist's helmet use.

Interaction term	Odds ratio	p value	95 % CI
Blood alcohol level with helmet use[a]			
1 Blood alcohol positive and not using helmet	18.1	< 0.001	15.9 – 20.4
2 Blood alcohol positive and using helmet	10.1	< 0.001	9.3 – 11.1
3 Blood alcohol negative and not using helmet	2.3	< 0.001	2.1 – 2.5
4 Blood alcohol negative and using helmet	1	–	1

In addition, our previous paper published in BMC Public Health in 2023 titled: Walking against traffic and pedestrian injuries in the United Kingdom: new insights (i.e., o). In this study, we specifically analysed the joint effect to examine whether the beneficial effect of walking against traffic on injury severity may apply to different situations. By doing so, we were able to compare injury outcomes in walking against-traffic crashes against those in walking with-traffic crashes.

Variables	AOR	95% CI
With traffic_Darkness unlit	1.48	1.29-1.70
With traffic_Midnight	1.60	1.37-1.87
With traffic_Rural area	2.20	1.92-2.51
With traffic_Elderly pedestrian	2.65	2.16-3.26
With traffic_HGVs	1.51	1.28-1.78

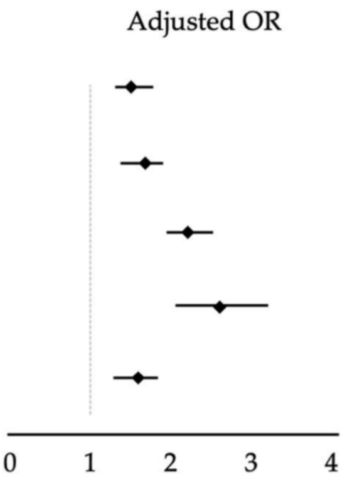

Fig. 2 Joint effects of walking with traffic and other variables on pedestrian fatalities

We believe this detailed explanation clarifies our methodology.

Reference:

m: Weinstein A., Sesso, H., Lee, I., Cook, N., Manson, J., Buring, J., Gaziano, J., 2004. The relationship of physical activity vs body mass index with type 2 diabetes in women. JAMA 290: 1188-1194.

n: Wiratama, B., Chen, P., Ma, S., Chen, Y., Saleh, W., Lin, H., Pai, C., 2020. Evaluating the combined effect of alcohol-involved and un-helmeted riding on motorcyclist fatalities in Taiwan. Accident Analysis and Prevention, 143, 105594.

o: Widodo, A. F., Chen, C., Chan, C. W., Saleh, W., Wiratama, B. S., & Pai, C. W. (2023). Walking against traffic and pedestrian injuries in the United Kingdom: new insights. BMC public health, 23(1), 2205.

Reviewer #3: Areas for Improvement:
3.1 Clarity and Conciseness:

Some sections of the text are verbose and could benefit from more concise language. For instance, the detailed descriptions of statistical methods and results could be streamlined without losing essential information.
Simplifying the language and structure would enhance readability and accessibility, particularly for readers who are not specialists in the field.

Author's response: We appreciate the reviewer's valuable suggestions. Concerning two reviewers who recommended extending several sections (i.e., reviewer #2 asked us to explain more on multivariate regression models and reviewer #4 requested for further discussions), we maintained a neutral stance for the time being. Nonetheless, we have revised the introduction to provide a clearer context and expanded our descriptions in the discussion section to provide broader insights into the implications of our findings. Additionally, detailed descriptions of the statistical methods have been included in the methods section, aimed at enhancing readability and accessibility for our readers.

3.2 Detailed Interpretation of Results:

While the results section provides extensive data, there is limited interpretation of what these results mean in practical terms. Adding more context about how these findings could influence policy or infrastructure design would be valuable. Discussing potential interventions based on the identified risk factors, such as specific infrastructure improvements or policy changes, would strengthen the practical implications of the study.
Author's response: We appreciate the reviewer's comment and suggestion. We have revised the discussion section of the manuscript and added one recommendation section to address findings that could potentially influence policy or infrastructure as follows (please refer to lines 404-412 on pages 23-24 of the manuscript):

> "Recommendations
>
> For overtaking crashes, we recommend implementing 'Share the Road' warning signs, especially in rural areas, and developing specialized cognitive training programs for elderly drivers. Regarding rear-end crashes, our suggestions include improving illumination during night time and implementing speed control measures on rural road segments. For door crashes involving parked cars, we propose enhancing driver sight triangles and increasing cyclist visibility. Moreover, implementing a two-stage door opening mechanism and an automatic

detection device in vehicles to alert drivers of bicycles approaching from behind could potentially be beneficial."

3.3 Comparative Analysis:
Including a comparative analysis with similar studies from other countries could provide a broader context for the findings and highlight whether these risk factors are unique to the UK or consistent globally.
Discussing how the UK's findings compare with those from the United States or other European countries, especially concerning the impact of infrastructure and vehicle types, could offer valuable insights:

Author's response: We appreciate the reviewer's comment. To our knowledge, no comparative analysis from other countries has been conducted for the three crash types (overtaking, rear-end, and door crashes). In addition, it is out of the scope of the current research to obtain crash data from other countries and conduct a large scale of comparative analysis. However, in our introduction sections, we have reviewed previous studies that focused on risk factors for these crash types individually or collectively on road segments (such as vehicle volume, traffic density, and number of lanes).

In the discussion section, we have discussed our findings with those of other studies in the US or elsewhere. For instance, previous analyses of overtaking crashes highlighted risk factors such as speeds exceeding 10 mph and the presence of pick-up trucks. Rear-end crashes were associated with conditions such as darkness, unlit surroundings, midnight hours, and reduced cognitive capabilities. Door crashes were found to be influenced by factors including urban roadways and the presence of taxis.

3.4 Providing more detailed information about the methodology, particularly the criteria for excluding certain data points, would enhance transparency. For example, explaining why specific demographic data were incomplete and how this might affect the results would be useful.
A discussion on the limitations of the data and the potential biases introduced by police reporting practices could provide a more nuanced understanding of the findings.

Author's response: We appreciate the reviewer's comment. To clarify the reasons for excluding junction cases and cyclists/motorcyclists as crash partners, we have added the following statements in the Methods section (please also kindly see lines 168 to 174; pages 9-10 in the manuscript):

“On a cautionary note, we removed junction cases to avoid the variability introduced when exogenous factors, such as junction geometry and control measures, are present at junctions. Furthermore, the cases involving other cyclists and motorcyclists were removed as we focused on vehicle-cycle crashes only. Missing data on sex, age, or speed limits were also excluded in the analysis. Excluding these data may impact our results in a marginal scale, as these data are likely to be single-bicycle crashes that in nature be underreported in police crash dataset [e.g., p]. “

Regarding the limitation of police reported crash data, the following statements have been added to the manuscript (please also kindly see lines 378 to 391; pages 22-23 in the manuscript):

“This study had several limitations that warrant acknowledgement. First, the substantial underreporting of nonfatal casualties to the police, particularly casualties involving cyclists not obligated to report accidents, is a critical factor to consider. Such underreporting, as highlighted by the U.K. Government’s Department for Transport, likely results in the incomplete representation of nonfatal and ‘slight’ casualties in road casualty data. Second, the STATS19 data utilised in this study lack critical variables, including precrash speeds, specific geometric characteristics of roadways, data regarding alcohol and illicit substance use, and cyclist speed at the time of an accident. Moreover, critical exposure data—such as those related to traffic flow, rider or driver experience, and other elements of risk exposure—are absent, and the absence of such details limits our ability to fully account for potential variations resulting from unobserved factors in the analyses. Finally, this study did not explore annual trends in each type of bicycle crash over the 30-year study period; investigating such trends could provide insights regarding changing behaviours among cyclists and motor vehicle drivers as well as the effects of legislative changes for road speed limits.”

p. Watson, Angela, Barry Watson, and Kirsten Vallmuur. "Estimating under-reporting of road crash injuries to police using multiple linked data collections." *Accident Analysis & Prevention* 83 (2015): 18-25.

3.4 Visual Aids:

Adding more visual aids, such as graphs or charts, could help in visualizing the key findings and making the data more accessible to readers.
A geographic distribution map showing where different types of crashes are more prevalent could add an interesting dimension to the analysis.

Author's response: We appreciate the reviewer's suggestions. We firstly reported our sampling by using a flowchart that helps readers understand what data were excluded and included in the analyses. Although we presented our statistical analyses in a traditional way (Tables 1 to 4), we illustrated a forest plot demonstrating the joint effects of several variables on the three crash types when other variables were controlled for (please refer to lines 213 to 276; pages 12-17 in the manuscript).

Regarding the geographic distribution map illustrating where these crash types were more prevalent, our research objective does not primarily emphasize the geographic effects of these three crash types. Rather, we focused on identifying risk factors for these crash types. While we appreciate this reviewer's valuable comment on this, we have identified this as an important research area as follows (please refer to lines 398 to 402; page 23 in the manuscript):

> "Future research directions could involve integrating GPS (Global Positioning System) data and weather conditions to analyse both the injury frequency and fatalities of bicycle crashes on road segments. Additionally, exploring the potential of autonomous vehicles for detecting approaching bicycles for door-crashes and implementing AI-controlled lighting systems in rural areas for cyclist detection could be promising areas for further study."

3.5 Future Directions:

Including a section on future research directions would be beneficial. Identifying gaps in the current research and suggesting areas for further investigation could guide subsequent studies.

Discussing the potential impact of emerging technologies, such as autonomous vehicles and advanced cyclist detection systems, on these crash types could provide a forward-looking perspective.

Author's response: We appreciate the reviewer's comment and suggestion. We have revised the discussion section of the manuscript and added one future research section (please refer to lines 398 to 402; page 23 in the manuscript). Furthermore, we have added one new section "Recommendation" that reports potential intervention points (please refer to lines 404-412 on pages 23-24 of the manuscript):

> "Recommendations
>
> For overtaking crashes, we recommend implementing 'Share the Road' warning signs, especially in rural areas, and developing specialized cognitive training programs for elderly drivers. Regarding rear-end crashes, our suggestions include improving illumination during night time and implementing speed control

measures on rural road segments. For door crashes involving parked cars, we propose enhancing driver sight triangles and increasing cyclist visibility. Moreover, implementing a two-stage door opening mechanism and an automatic detection device in vehicles to alert drivers of bicycles approaching from behind could potentially be beneficial."

Future research directions:

"Future research directions could involve integrating GPS (Global Positioning System) data and weather conditions to analyse both the injury frequency and fatalities of bicycle crashes on road segments. Additionally, exploring the potential of autonomous vehicles for detecting approaching bicycles for door-crashes and implementing AI-controlled lighting systems in rural areas for cyclist detection could be promising areas for further study."

Reviewer #4: This Study is technically sound and has potential to add to the body of knowledge involving bicycle riding safety in the UK and everywhere across the globe. It has adhered to the research and publication ethics, however, the study still need revision on some of the key identified areas which i have pointed out, starting from abstract, background, results and discussions.

4.1 Abstract

The abstract is lacking the background section, please see the comment on the pdf

This abstract is lacking the background section, which must start when presenting structured abstract. Also there is no objective put here, but rather the research problem investigated.

<u>Author's response:</u> We appreciate the reviewer's comment and suggestion. We have revised the abstract to add background and objects as follows (please refer to lines 23 to 27; page 2 in the manuscript):

> "Background: Relevant research has provided valuable insights into risk factors for bicycle crashes at intersections. However, few studies have focused explicitly on three common types of bicycle crashes on road segments: overtaking, rear-end, and door crashes.
>
> Objective: This study aims to identify risk factors for overtaking, rear-end, and door crashes that occur on road segment."

4.1.1 Abbreviations should be defined when they are first mentioned

<u>Author's response:</u> We appreciate the reviewer's suggestion. We have revised the abstract to include the full definitions of abbreviations upon their first appearance as follows (please refer to lines 31; page 2 in the manuscript).

> "Abstract: AOR (adjusted odds ratio)"

4.2 Introduction

4.2.1 The authors did not explain the context of the previous study, where this current study was based, but only cited it. For my comments also see the pdf with my comments on this section

4.2.2 See the comments above on the abstract to enhance this one

4.2.3 Highlight some key findings of this previous study here to avoid making the readers look for the findings on their own. The point of scientific writing is to make the work easy to understand

<u>Author's response:</u> We appreciate the reviewer's comment and suggestion. We have revised our introduction section to include the reviewer's suggestion, providing an explanation of the previous study and emphasizing our key findings accordingly as follows (please refer to lines 101 to 106; page 5 in the manuscript):

> "The primary objective of this study, building on our previous research into risk factors related to overtaking, rear-end, and door crashes, is to conduct a more comprehensive investigation. Specifically, Pai identified buses and coaches as common crash partners in overtaking crashes; poor visibility, traversing manoeuvres, and teenage cyclists as risk factors for rear-end crashes; and built-up areas as a risk factor for door crashes."

4.3 Methodology

The method section was described well and is adequate, although we need to know whether normality checks were conducted.

<u>Author's response:</u> Thank you for your positive feedback on the method section and for your valuable suggestion regarding normality checks. We employed multivariate logistic regression models in our investigation, which do not require assuming the normality of the predictor variables. Logistic regression is resilient to deviations from normality as it estimates the likelihood of a binary outcome instead of assuming a normal distribution of the variables. Consequently, we refrained from performing formal normality assessments for the predictor variables.

4.4 Results

This area still requires more work. The way the results were presented was hasty, and we need to redo some of the highlighted sections. For example, a separate Univariate table is needed as Table 1.

<u>4.4,1 I think you need a joint univariate table of all factors studied that combining it all in the Bivariate table. It is a lazy way of reporting that require a reader to tease out proportions on their own. address this</u>

<u>Author's response:</u> We appreciate the reviewer's comment. In response to the suggestion for a joint univariate table of all studied factors, we acknowledge the importance of presenting comprehensive data that is readily interpretable. Our analysis

included an examination of crash type distributions across multiple independent variables. To explore these relationships, we employed Chi-square tests. Variables with significance levels below 0.2 were identified to minimize type II errors and were considered significantly associated with the outcome variables ($p < 0.05$). These variables were subsequently included in multiple logistic regression models.

We utilized stepwise logistic regression to estimate odds ratios while controlling for specific factors, following a methodological approach well-established in traffic injury studies (e.g., references a and b) and detailed in previous research (e.g., reference c). This approach allows for a nuanced understanding of how various factors interact to influence crash types, ensuring our findings are robust and informative. This methodological approach is well-established in the study of traffic injuries (e.g., references a and b) and has been detailed in previous studies (e.g., reference c).

a: Chen, P-L, Pai, C-W. Evaluation of injuries sustained by motorcyclists in approach-turn crashes in Taiwan. Accident Analysis and Prevention, 2019, 124, 33-39;

b: Chien, D-K., Hwang, HF, Lin, MR. Injury severity measures for predicting return-to-work after a traumatic brain injury. Accident Analysis and Prevention, 2017, 98, 101-107;

c: Maldonado G, Greenland S. Simulation study of confounder-selection strategies. Am J Epidemiol 1993, 138, 11, 923-936).

4.4.2 Use one decimal place and not two

Author's response: We appreciate this reviewer's comment. We have updated our tables (Tables 1-4) to display data with one decimal place instead of two (please refer to lines 213 to 276; pages 12-17 in the manuscript).

4.4.3 After inserting a combined univariate table, please remove these percentages, as they are very misleading

Author's response: We appreciate this reviewer's comment. However, presenting percentages is crucial for demonstrating the distribution among each crash type and others. Therefore, we have decided to continue using percentages as presentation in our manuscript.

4.4.5 Tables: Here put frequencies/percenatgase and removed all the percentages from the table. the same applies to all other tables

<u>Author's response:</u> We appreciate this reviewer's comment. Nevertheless, it is essential to use percentages to clearly demonstrate the distribution of each crash type across a set of variables. By reporting these percentages, we are able to identify whether one certain variable was over-involved in one crash type. Therefore, we have opted to maintain the use of percentages in our presentation.

<u>4.4.6 All most all the bivariate table has not been interpreted. but summarize using phrases like serveral variables as shown in table 2.</u>

<u>Author's response:</u> We appreciate this reviewer's comment. We have revised our results section to incorporate the reviewer's suggestion and rephrase the sentence accordingly (please refer to lines 215 to 229; page 13 in the manuscript):

> "Several variables in Table 2 reveal significant differences between rear-end crashes and non-rear-end crashes. Specifically, a higher proportion of rear-end crashes occurred under darkness-unlit conditions (50.2%) compared to darkness-lit conditions (37.5%). Additionally, rear-end crashes were more prevalent in rural areas with speed limits of ≥ 40 mph (43.0%) compared to urban areas with speed limits of 20–30 mph(33.1%). Crashes involving crash partners aged ≥ 65 accounted for 39.7% of rear-end crashes, which was higher compared to other age groups (age 41–64: 33.0% and ≤18: 36.0%). Furthermore, rear-end crashes were more likely to occur during midnight (47.6%) compared to rush hours (36.3%). Taxis were frequently involved in rear-end crashes (42.4%), as were male crash partners (36.8%). These findings highlight the significant influence of various factors on the likelihood of rear-end crashes. Variables such as darkness-unlit conditions, higher speed limits in rural areas, crash time, and characteristics of the crash partner all emerged as significant determinants. Specifically, rear-end crashes were notably more prevalent under darkness-unlit conditions, in rural areas with higher speed limits, during midnight hours, and involving certain characteristics of crash partners. Importantly, these associations were statistically significant, as indicated by the Chi-squared test ($p < 0.001$)."

<u>4,4,7 Do inteprete the results individually for all the significant factors.</u>

<u>Author's response:</u> We appreciate this reviewer's comment. We have revised our discussion section to incorporate the reviewer's suggestion and rephrase the sentence accordingly (please refer to lines 232 to 248; pages 14-15 in the manuscript):

> "As shown in Table 3, several variables can contribute to door crashes involving bicycles. Door crashes predominantly occurred in urban areas with speed limits of 20-30 mph (6.22%), while a significantly lower proportion occurred in rural

areas with speed limits ≥ 40 mph (0.45%). These crashes were overrepresented during non-rush hours (5.54%) and rush hours (4.94%) compared to evening (4.26%) and midnight (2.35%). Cyclists were more frequently involved in door crashes on weekdays (5.35%) than weekends (3.73%). As many as 8.21% of all female cyclists were involved in door crashes, which is higher than the involvement rate among males (4.24%).Taxi and private hire cars were overinvolved in door crashes (10.55%) compared to cars (5.17%) and buses/heavy goods vehicles (3.13%). Crash partners aged ≤18 years (5.22%) and 19-40 years (5.26%) were disproportionately involved in door crashes compared to older age groups, and female crash partners were overrepresented in door crashes (7.42%) compared to males (4.23%). These results were statistically significant, as indicated by the Chi-squared test ($p < 0.001$). They suggest that various factors including traffic conditions (rural areas, crash time), cyclist demographics (younger age, gender), and characteristics of the crash partner (taxi/private hire cars)—significantly contribute to the likelihood of door crashes involving cyclists."

4.4.8 where are the corresponding p-values. include them for all the significant risk factors

Author's response: We appreciate this reviewer's comment. We have revised our Results section to include the reviewer's suggestion and have added the corresponding p-values accordingly (please refer to lines 253 to 272; page 16 in the manuscript):

For example: "(AOR = 2.912, 95% CI = 2.384–3.556; p<0.001)."

4.4.9 Here, present both the crude and adjusted odd ratios

Author's response: We appreciate the reviewer's comment. In response, we have focused on presenting the adjusted odds ratios (AOR) and their corresponding 95% confidence intervals in our manuscript.

To address the analysis of crash types across various independent variables, we conducted Chi-square tests to assess the association between dependent and independent variables. The direction of the independent variables will be clarified in the subsequent multivariate logistic regression models.

Significant variables identified through stepwise selection were included in the multiple logistic regression models. The adjusted odds ratios (AOR) and their 95% confidence intervals were then calculated from these final models. This approach, widely used in traffic injury studies (e.g., a, b), ensures robust methodology by controlling for other variables (e.g., c).

a: Chen, P-L, Pai, C-W. Evaluation of injuries sustained by motorcyclists in approach-turn crashes in Taiwan. Accident Analysis and Prevention, 2019, 124, 33-39;

b: Chien, D-K., Hwang, HF, Lin, MR. Injury severity measures for predicting return-to-work after a traumatic brain injury. Accident Analysis and Prevention, 2017, 98, 101-107;

c: Maldonado G, Greenland S. Simulation study of confounder-selection strategies. Am J Epidemiol 1993, 138, 11, 923-936).

<u>4.4.10 Also do interpret these results. For instance what does the odd ratio of 2.93 mean in this case?</u>

<u>Author's response:</u> We appreciate the reviewer's comment and suggestion. We have revised our results section to incorporate the reviewer's suggestion and have interpreted the meaning of odds ratios in our findings accordingly (please refer to lines 279 to 286; pages17-18 in the manuscript):

> "The results identified several key risk factors for both overtaking and rear-end crashes. The risk of overtaking crashes showed a significant increase of 193% in rural areas when elderly drivers were involved (AOR = 2.93, 95% CI = 2.79–3.08), and similarly when heavy goods vehicles (HGVs) were the crash partner (AOR = 2.62, 95% CI = 2.46–2.78). Elderly cyclists also faced a higher risk of overtaking crashes on weekends (AOR = 1.56, 95% CI = 1.34–1.81).
>
> Regarding rear-end crashes, the risk increased notably with unlit darkness during midnight (AOR = 1.68, 95% CI = 1.48–1.90) and was significantly higher in rural areas (AOR = 2.15, 95% CI = 2.01–2.31)."

<u>4.4.11 you look at risk factors and not only environment factors, what about factors like sex, age. are they from the environment too, and yet you included them.</u>

<u>Author's response:</u> We appreciate the reviewer's comment. In our multivariate logistic regression results in Table 4, we analyzed and presented such factors such as cyclist's sex and age for each crash type (please refer to lines 275 to 276; page 17 in the manuscript). Moreover, in our joint-effect analysis, cyclist's age (≥65-year-old cyclist) was combined and analyzed with other variables.

4.5 Discussion

The section also needs serious work, especially on the way the findings were discussed. The authors should consider discussing their own findings rather than those of other studies. There is also a need to have a section for recommendations rather than merging it within result

4.5.1 You dont need this type of writing, just discuss the findings

Author's response: We appreciate the reviewer's comment. We have revised our discussion section to delete the paragraph as the reviewer's suggestion as follows (please refer to lines 305; page 19 in the manuscript):

> "Delete: These findings warrant further discussion and thus are elaborated on in this section of this paper."

You have not discussed the findings. Yes you found HGVs a risk for overtaking crash, so tell us why you think that is a risk factor. in other word explain your findings and then place it in the context of other study

Author's response: We appreciate the reviewer's comment and suggestion. We have revised our discussion section to integrate the reviewer's suggestion and provide a discussion on how heavy goods vehicles (HGVs) pose a risk for overtaking crashes accordingly as follows (please refer to lines 308 to 317; page 19 in the manuscript):

> "These findings align with previous research that identified elderly drivers, speeds exceeding 10 mph, and the presence of pick-up trucks as factors contributing to increased risk for overtaking crash. Specifically, HGVs possess several characteristics that amplify this danger. Their large blind spots make it difficult for drivers to see cyclists, increasing the likelihood of crashes during overtaking. Additionally, HGVs are less maneuverable compared to passenger cars, which reduces their ability to avoid crashes if cyclists suddenly enter their path. The speed and distance perception issues between HGVs and cyclists further complicate the judgment of safe overtaking gaps. Furthermore, HGVs require longer stopping distances due to their size and weight, which can lead to severe consequences if a sudden need to brake arises."

4.5.3 Do not discuss other people's findings, just discuss your findings and only state whether it agrees of disagrees with what Pai et al found for example

Author's response: We appreciate the reviewer's comment and suggestion. We have revised our Discussion section to incorporate the reviewer's suggestion and provide a

discussion on our findings, comparing them with previous studies accordingly (please refer to lines 318 to 322; pages 19-20 in the manuscript):

> “Regarding the association with buses or HGVs, our findings are consistent with existing research suggesting that time pressures on HGV drivers for timely loading and unloading might lead to more reckless driving. Specifically, our results align with the observations made by Pai et al., who also mentioned higher crash rates involving buses or HGVs, supporting the idea that these time pressures contribute to increased crash risks.”

4.5.4 Take this to the recommendation section

Author’s response: We appreciate the reviewer’s comment and suggestion. We have added a recommendations section (please refer to lines 404-412; pages 23-24 of the manuscript):

> “Recommendations
>
> For overtaking crashes, we recommend implementing 'Share the Road' warning signs, especially in rural areas, and developing specialized cognitive training programs for elderly drivers. Regarding rear-end crashes, our suggestions include improving illumination during night time and implementing speed control measures on rural road segments. For door crashes involving parked cars, we propose enhancing driver sight triangles and increasing cyclist visibility. Moreover, implementing a two-stage door opening mechanism and an automatic detection device in vehicles to alert drivers of bicycles approaching from behind could potentially be beneficial.”

4.5.5 Good use of references but first tells why you found what you found. And again your study was looking at comparing the risk factors for overtaking crashes with what was previously found in your study and the findings of that study needed to be described well in this study too

Author’s response: We appreciate the reviewer’s comment and suggestion. We have revised our Discussion section to incorporate the reviewer's suggestion and provide a discussion on our findings, comparing them with previous studies accordingly (please refer to lines 328 to 332; page 20 in the manuscript):

> “We found that as individuals age, their risk of being involved in road accidents increases, primarily due to declines in cognitive capabilities. Our study corroborates these findings by showing that older cyclists are more susceptible to accidents during overtaking maneuvers, which can be attributed to diminished reaction times and impaired decision-making abilities, their health, and their driving performance.”

4.5.6 I think you need to also link this to delays in reacting as compared to the younger cyclist or driver.

Author's response: We appreciate this reviewer's comment. We have revised our Discussion section to integrate the reviewer's suggestion and provide a discussion on delays in reaction among elderly cyclists or drivers accordingly (please refer to lines 328 to 332; page 20 in the manuscript):

> "We found that as individuals age, their risk of being involved in road accidents increases, primarily due to declines in cognitive capabilities. Our study corroborates these findings by showing that older cyclists are more susceptible to accidents during overtaking maneuvers, which can be attributed to diminished reaction times and impaired decision-making abilities, their health, and their driving performance."

4.5.7 This is supposed to be a recommendation but first of all it is not right. it is not what you found but what you think is making more elder drivers to get into overspreading crashes. So recommend only based on what you found and not based on what you think.

Author's response: We appreciate the reviewer's comment and suggestion. We have revised our discussion section to integrate the reviewer's suggestion as follows (please refer to lines 339 to 346; pages 20-21 in the manuscript):

> "Based on our study's findings, we recommend the development of specialized interventions to improve road safety for elderly cyclists. Our analysis reveals that older cyclists are at a higher risk of being involved in overtaking crashes, with this increased risk being strongly linked to declines in cognitive capabilities associated with aging. To address this issue, we advocate for the implementation of targeted cognitive training programs specifically designed for elderly cyclists. These programs should focus on enhancing critical skills such as reaction time, situational awareness, and decision-making abilities, which are crucial for reducing crash risk and improving overall road safety. "

4.5.7 Now this is a good statement that should have followed your first sentence, starting from the full stop after segments. then you can now show us how similar it is with what Pai and others found.

Author's response: We appreciate this reviewer's comment. If we understand this reviewer correctly, this reviewer makes a valid argument that Advanced Stop Lines (ASLs), also called bike boxes that had been implemented in the UK for decades, would be beneficial in reducing conflicts between cars and cyclists. However, our study focuses on cyclist crashes that occurred on road segments only (i.e., 20 metres away from junctions); as a result, we remain reserved with discussing this finding with this engineering measure (i.e., ASLs).

In addition to this, we routinely discussed our current findings with those of Pai; for instance, HGVs, unlit streets and midnight hours, and taxis have been similarly identified as a risk factor for overtaking crashes, rear-end crashes, and door crashes, respectively.

4.6 discussions.

4.6.1 They need to tell us how they tried to minimize the biases that could have been introduced by the many study limitations identified for this study.

Author's response: We appreciate this reviewer's comment. One inherent problem with police-reported crash data is the variables not readily available, hereby causing unobserved heterogeneity across the observations. To overcome such a limitation, we estimated separate regression models, as suggested by Kim et al. (2006), for the three crash types; such an approach provides greater explanatory power compared to single overall models. Further, we conducted joint-effect analyses of several variables of interest that capture heterogeneity. In our previous studies, we adopted the above-mentioned approaches to overcome the inherent problem with a success (see, for example, Pai and Saleh, 2008; Pai and Jou, 2014).

To clarify this, the following statements have been added to the Discussion section of the manuscript (please refer to lines 391 to 397; page 23 in the manuscript):

> "One inherent problem with police-reported crash data is the variables not readily available, hereby causing unobserved heterogeneity across the observations. To overcome such a limitation, we estimated separate regression models, as suggested by Kim et al. (e.g., d), for the three crash types; such an approach provides greater explanatory power compared to single overall models. Further, we conducted joint-effect analyses of several variables of interest that capture heterogeneity. In our previous studies, we adopted the above-mentioned approaches to overcome the inherent problem with a success (e.g., e, f)."

d: Kim, D., Washington, S., Oh, J., 2006. Modelling crash outcomes: new insights into the effects of covariates on crashes at rural intersections. Journal of Transportation Engineering. 132 (4), 282-292.

e: Pai CW, Jou RC, 2014. Cyclists' red-light running behaviours: An examination of risk-taking, opportunistic, and law-obeying behaviours. Accident Analysis and Prevention. 62,191-198.

f: Pai CW, Saleh W., 2008. Modelling motorcyclist injury severity by various crash types at T-junctions in the UK. Safety Science. 13, 98-98.

4.6.2 Present both the strength and limitations of the study. And you have really brought the limitation well, but my question would be, despite knowing all these why did you decided to carry on to utilised this dataset as opposed to others. Please tell us how you catered for these limitations as away of reducing bias that might have been introduced by them

Author's response: We appreciate this reviewer's comment. We have added a section discussing the strengths of our study before addressing its limitations as follows (please refer to lines 370 to 377; page 22 in the manuscript):

> "The strengths of this study include the use of STATS19 datasets spanning from 1991 to 2020, which provides a robust statistical foundation and a broad perspective on trends in bicycle crashes. By focusing specifically on three crash types on road segments—overtaking, rear-end, and door crashes—the study provides a comprehensive and focused analysis, which can yield more actionable insights and more effective recommendations. The UK-based dataset ensures that the findings are particularly relevant for local policy and safety interventions. Additionally, the application of statistical techniques and the consideration of various factors, such as crash partner and time of day, enhance the validity and depth of the analysis."
