# Supplementary material for: Risk factors for overtaking, rear-end, and door crashes involving bicycles in the United Kingdom: Revisited and reanalysed
Source: PLoS One. 2025 Jan 3;20(1):e0315692. doi: 10.1371/journal.pone.0315692 (PMC11698528; doi:10.1371/journal.pone.0315692)
Supplement: Attachment — Submitted filename: 10-18 PLOS Response.pdf [file pone.0315692.s005.pdf]

Dear Editors and Reviewers,

We greatly appreciate the valuable comments and suggestions raised by reviewers. Please very kindly see our responses below, as well as the revised manuscript. We would be glad if you could have our manuscript reviewed again.

Best regards,

Chih-Wei Pai (Prof)

Graduate Institute of Injury Prevention and Control College of Public Health

Taipei Medical University

Reviewer 2:

1.1 In the Abstract as well as in the results (main text) AOR sometimes expressed with three digits (decimals) and other places two decimals (please consider and use effective digits “decimals”).
Author’s response: We appreciate the reviewer’s comment and suggestions. All AORs have been amended to two decimals (Please refer to lines 34 to 40 on page 2 in the manuscript).

1.2 In the abstract “results section”: the AOR are sometimes very narrow (please explain).
Author’s response: We appreciate the reviewer’s comment and question. The narrow confidence intervals (CIs) for the adjusted odds ratios (AORs) indicate high precision in our estimates. This precision is primarily due to our large sample size, which reduces variability and enhances reliability. For example, the AOR for "male as crash partner” in overtaking crashes is 1.28 with a CI of 1.25-1.33, reflecting a strong effect size and contributing to the narrow CI. Variability and heterogeneity in the data can affect CI width. Risk factors with more consistent effects across the dataset often show narrower CIs (e.g., a).

a. Katz, M. H. (2011). Multivariable Analysis: A Practical Guide for Clinicians and Public Health Researchers.

1.3 In the introduction: word roundabouts are repeated “study demonstrated that roundabout significantly reduces -----“
Author’s response: We appreciate the reviewer’s comment and suggestions. We have revised the manuscript. (Please refer to lines 74 to 76; page 4 in the manuscript):

> “One study found that roundabouts with dedicated cycle tracks significantly lower the risk of injury for cyclists compared to those without such bicycle infrastructure.”

1.4 In the rationale, the authors still need to emphasize the significance of the three types of crashes, this part of the introduction barely touched this point????
Author’s response: We appreciate the reviewer’s comment and suggestions. We have revised the manuscript. (Please refer to lines 104 to110; pages 5 -6 in the manuscript):

> “The high mortality rate from crashes on road segments underscores the significant risks linked to overtaking, rear-end, and door crashes. Overtaking, involving high-speed maneuvers, greatly increases the likelihood of severe accidents. Rear-end crashes, frequently triggered by sudden stops or aggressive tailgating, pose a persistent threat to cyclists. Furthermore, injuries sustained by cyclists striking an opening car door can be devastating due to the impacts from the door, ground, or vehicles behind. These critical issues highlight the urgent need for identifying risk factors for these crashes.”

Statistical analysis:
1.5 - Rationale for considering p value of 0.2 at the univariate (bivariate) level to be incorporated in the multiple Logistic regression models???
Author's response: We appreciate the reviewer's comment and question. In the first and second round of review, this reviewer expressed concerns over our use of Chi-square tests to examine the relationship between three crash types and the independent variables. We have now opted to estimate the crude odds ratio by univariate logistic regressions. Please kindly see Table 4 lines 259 to 260; page 15 in the manuscript.

1.6- How the data were handled statistically: descriptive and inferential methods should be mentioned in this section
Author's response: We appreciate the reviewer's comment and question. In response to your comment, we have revised the section on statistical handling to provide a more comprehensive explanation of both the descriptive and inferential methods employed. (Please refer to lines 182 to 191; page 9 in the manuscript).

> "We initially utilized descriptive statistics to examine the distribution of crash types across various variables such as lighting conditions, speed limit, time of day, and day of the week. Demographic details concerning cyclist casualties encompassed age and sex, while information about the crash partner included vehicle type, age, and sex. This preliminary analysis provided a general picture of basic characteristics of the data and identification of potential patterns. For inferential analysis, we applied the Chi-squared test to investigate associations between crash type and various factors, including cyclist and motorist characteristics, vehicle features, roadway conditions, and temporal variables. We then estimated crude odds ratios by estimating univariate logistic regression and adjusted odds ratios by multivariate logistic models, respectively."

1.8- What type of model was used (stepwise, or else), how the model was tested to be fit???
Author's response: We appreciate the reviewer's comment and question. We used multivariate logistic regression with backward selection to compute adjusted odds ratios (AORs). This method involves initially including all potential predictors and then iteratively removing the least significant variables based on their p-values.
In terms of model fit statistics, the final models were chosen based on the ρ^2 statistics (e.g., b). The ρ^2 statistics for the estimated models range from 0.327 to 0.398, indicating a reasonable model fit.

b. Ben-Akiva, M. E., & Lerman, S. R. (1985). *Discrete choice analysis: theory and application to travel demand* (Vol. 9). MIT press.

1.9- How the variables were categorized to be suitable for the inclusion of logistic regression analysis?

Author's response: We appreciate the reviewer's comment and question. Considering findings from past studies and selecting the model with the most parsimonious and robust statistical properties (e.g., goodness of fit, reasonable parameter magnitudes, and t-statistics), the variables were categorized and explained as follows:

First, age data were divided into four categories: ≤18 (not of legal driving age), 19–40, 41–64, and ≥65 (defined as older age by WHO standards). This classification highlights the different risk profiles associated with each age group.

The variable "time of crash" was classified into four periods—midnight (00:00–06:00), rush hours (07:00–08:00 and 17:00–18:00), non-rush hours (09:00–16:00), and evening (19:00–23:00)—to account for fluctuations in traffic patterns and accident likelihood throughout the day.

Speed limits were categorized by location into two types: nonbuilt-up areas (rural, ≥40 mph) and built-up areas (urban, 20–30 mph).

Day of the week was grouped as either weekday or weekend to evaluate variations in crash patterns.

These classifications have been commonly adopted in safety literature (e.g. , c; d).

c. Widodo, Akhmad Fajri, et al. "Walking against traffic and pedestrian injuries in the United Kingdom: new insights." *BMC public health* 23.1 (2023): 2205.

d. Wiratama, Bayu Satria, et al. "Joint effect of heavy vehicles and diminished light conditions on paediatric pedestrian injuries in backover crashes: a UK population-based study." *International journal of environmental research and public health* 19.18 (2022): 11689.

110- The reference group in the multivariate regression table is not consistent along the three types of crashes??? Please explain.

Author's response: We appreciate the reviewer's comment and question. The reference groups in the univariate and multivariate analysis have been assigned consistent. Please kindly see Table 4 lines 259 to 260; pages 14-15 and Table 5 lines 292 to 293; pages 16-17 in the manuscript.

1.11- Joint sensitivity analysis should be mentioned in this section "indication, methods and output"

Author's response: We appreciate the reviewer's insightful comments and suggestions. To illustrate the effectiveness of models with joint effects, we found that these models produced a

higher log-likelihood at convergence and demonstrated an improved overall fit, as indicated by a better ρ^2 statistic.

Moreover, we performed a likelihood ratio test (e.g., e) to confirm the superiority of the joint effects models over the general models. The test statistic is given by:

$$\chi^2 = -2[LL(\beta_G) - LL(\beta_J)]$$

Where $LL\ (\beta_G)$ represents the log-likelihood at convergence for the general model, and $LL(\beta_J)$ is for the joint effects model. This statistic follows a χ^2 distribution, with degrees of freedom equal to the difference in the number of parameters between the general and joint effects models.

e. Vuong, Q.H., 1989. Likelihood ratio tests for model selection and non-nested hypothesis. Econometrica 57, 307-333.

Results:

1.12- The previous comments on using the Chi-square test remained the same??? Non-specific, non-parametric test and can't' point out to the direction of significance???

Author's response: We appreciate this reviewer's comment. In addition to the multivariate logistic regression, we have now estimated the univariate logistic regression models. Please kindly see Table 4 lines 259 to 260; pages 14-15 and Table 5 lines 292 to 293; pages 16-17 in the manuscript.

1.13- What software used to produce figure 2???

Author's response: We appreciate the reviewer's comment and question. We recreated the figure from the previous article (e.g., f) using Photoshop and then edited it in PowerPoint.

f. Pai C-W. Overtaking, rear-end, and door crashes involving bicycles: an empirical investigation. Accid Anal Prev. 2011;43(3):1228-35.

Review 4

4.1 This has been addressed but in the main document start with background under the background sentences, conclude it with the objective, instead of presenting it as a separate paragraph.

Author's response: We appreciate the reviewer's comment and suggestions. We have revised the manuscript. (please refer to lines 23 to 27 ; page 2 in the manuscript):

> **"Background and Objective:** Relevant research has provided valuable insights into risk factors for bicycle crashes at intersections. However, few studies have focused explicitly on three common types of bicycle crashes on road segments: overtaking, rear-end, and door crashes. This study aims to identify risk factors for overtaking, rear-end, and door crashes that occur on road segments."

4.2 I understand this response; however, you need to conduct a normality check for all continuous variables like age and others like distance. This helps you to present either the mean age or the median age

Author's response: We appreciate the reviewer's comment and suggestions. Normality check for continuous variables is needed only while estimating a linear regression model. In our study, we estimated several logistic models in which testing for normality and homoscedasticity is not needed. For a comprehensive discussion on the derivation of logistic regression models, see Hosmer et al. (e.g., g).

g. Hosmer Jr, David W., Stanley Lemeshow, and Rodney X. Sturdivant. Applied logistic regression. John Wiley & Sons, 2013.

4.3 N(%) consider using this type of reforestation and removed the percentage signs from the table

Author's response: We appreciate the reviewer's comment and suggestions. We have removed the percentage signs and replaced them with "n (%)" in the tables 1, 2 and 3. (Please refer to lines 221-222 of page 11; lines 237 -238 of pages 12- 13; lines 254-255 of pages 13- 14 in the manuscript).

4.4 Data analysed should replace this, you didn't collect data

Author's response: We appreciate the reviewer's comment and suggestions. We have revised the manuscript. (Please refer to lines 160; page 8 in the manuscript):

> "Data analysis"

4.5 I insist this be removed, but keep the proportion there and take this up and say N(%) or read other publication to see how this is presented

Author's response: We appreciate the reviewer's comment and suggestions. We have removed the percentage signs and replaced them with "n (%)" in the table1, 2 and 3. Please refer to lines 221-222 of page 11; lines 237 -238 of pages 12- 13; lines 254-255 of pages 13- 14 in the manuscript.

4.6 This has not been fully addressed. What the authors did was just introduced the corresponding Odds Ratios and P-Values but no result interpretation. Consider doing something like this, "having a HGVs as crash partners had 2.9 times higher likelihood of being involved in overtaking crash", something like this for all the significant variables.

Author's response: We appreciate the reviewer's comment and suggestions. We have revised the manuscript. (Please refer to 293 to 295; page 17 in the manuscript):

> "In overtaking crashes, the presence of heavy goods vehicles (HGVs) as partners increases the likelihood by 1.3 times (AOR = 1.30, 95% CI = 1.27-1.33; $p < 0.001$)."

4.7 This has now been introduced, however, start with what you found, then bring the reason supporting those findings and lastly place it in the context of other study and cite it.

Author's response: We appreciate the reviewer's comment and suggestions. We have outlined the reasons supporting these findings and, finally, situated them within the context of existing research, providing appropriate citations. (Please refer to lines 344 to 347; pages 19-20 in the manuscript):

> "Their large blind spots make it difficult for drivers to see cyclists, increasing the likelihood of crashes during overtaking [e.g., c]. Additionally, HGVs are less manoeuvrable compared to passenger cars, which reduces their ability to avoid crashes if cyclists suddenly enter their path [e.g., d]. The speed and distance perception issues between HGVs and cyclists further complicate the judgment of safe overtaking gaps[e.g., e]."

c. Marshall, Russell, and Stephen Summerskill. "An objective methodology for blind spot analysis of HGVs using a DHM approach." *DS 87-8 Proceedings of the 21st International Conference on Engineering Design (ICED 17) Vol 8: Human Behaviour in Design, Vancouver, Canada, 21-25.08. 2017*. 2017.

d. Frings, Daniel, Andy Rose, and Anne M. Ridley. "Bicyclist fatalities involving heavy goods vehicles: Gender differences in risk perception, behavioral choices, and training." *Traffic injury prevention* 13.5 (2012): 493-498.

e. Chew, Esther Li-Wen, and Amanda Stephens. "Human Factors That Impact HGV Drivers From Being Aware of VRUs Through Direct and Indirect Vision Mechanisms."

4.8 I think you need to reference this in the method section also where you discussed the data source. Some readers don't reach here

Author’s response: We appreciate the reviewer’s comment and suggestions. We have revised the manuscript. (please refer to 135 to 137; page 7 in the manuscript):

> “The data that support the findings of this study are openly available at https://figshare.com/ndownloader/files/48173452.”
